# Supplementary material for: Rapid label-free identification of seven bacterial species using microfluidics, single-cell time-lapse phase-contrast microscopy, and deep learning-based image and video classification
Source: PLoS One. 2025 Sep 8;20(9):e0330265. doi: 10.1371/journal.pone.0330265 (PMC12416834; doi:10.1371/journal.pone.0330265)
Supplement: S1 Appendix — The document shows all traps that either had the label manually changed or were removed from the test set after processing of the labeling tool. (PDF) [file pone.0330265.s001.pdf]

Data cleaning and testset adjustments

The following are all adjustments that were made to the testset after the labeled assignment by the labeling tool.

Changed to *K. Pneumoniae*

The following traps had a very weak but present signal in the Cy3 channel, and were subsequently changed to *K. Pneumoniae*.

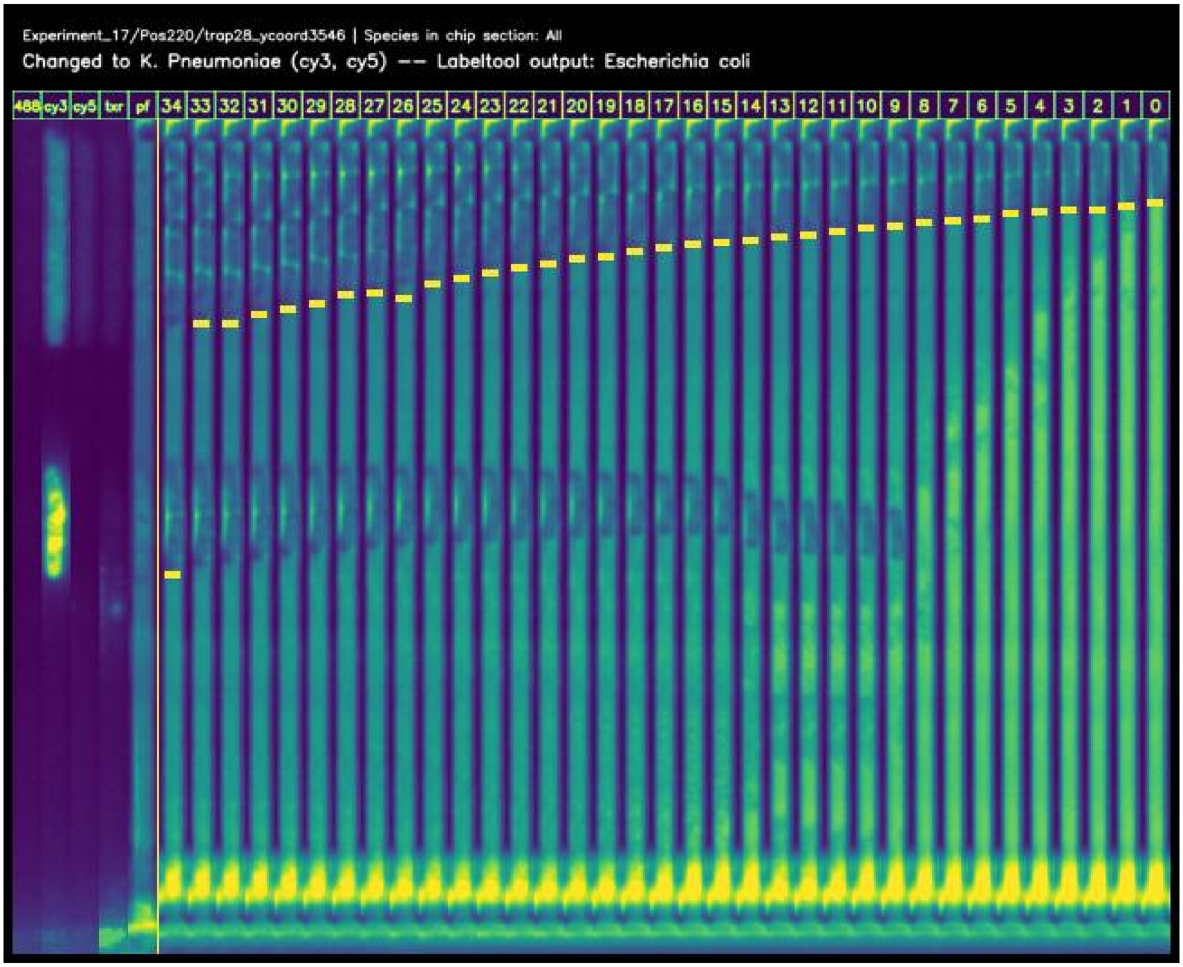

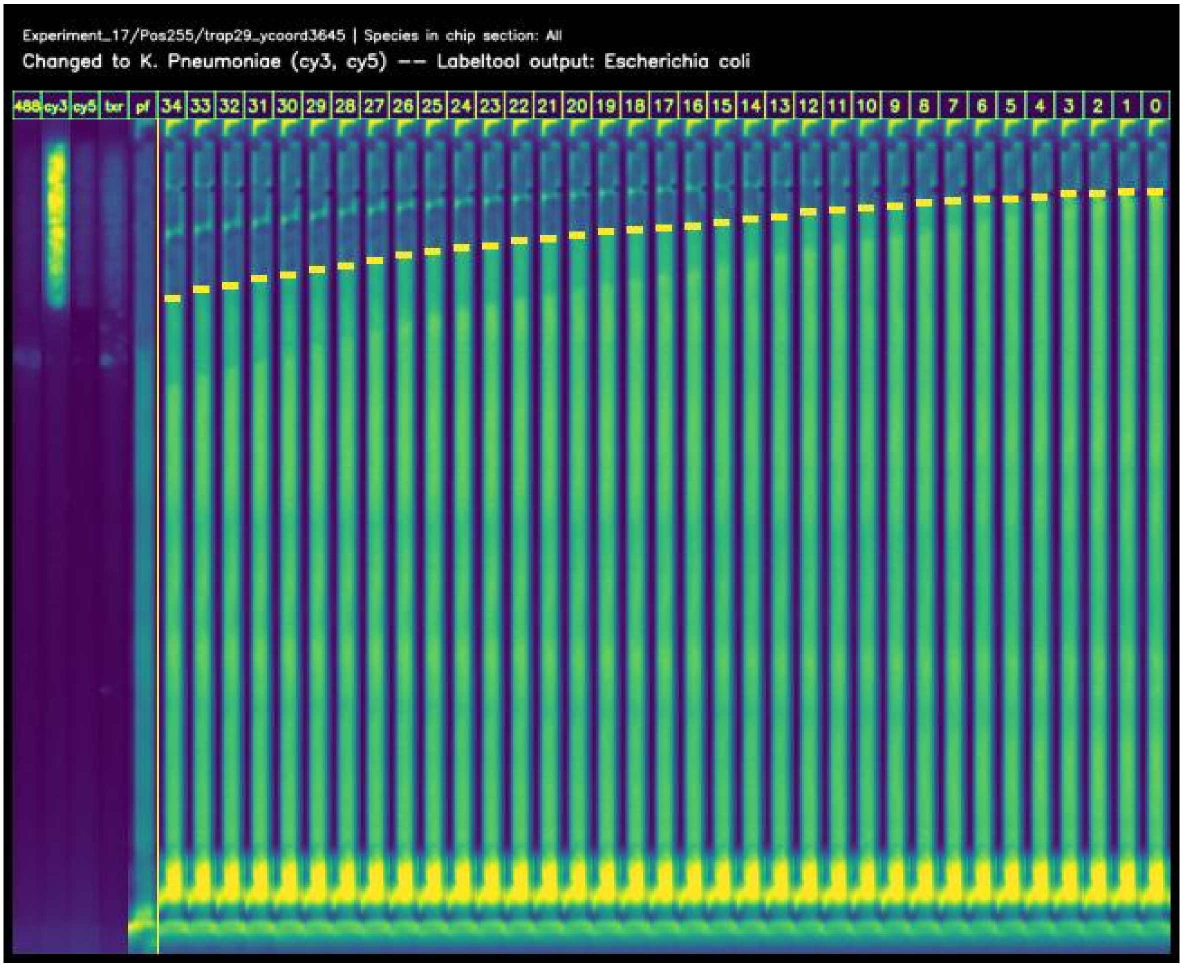
Experiment_17/Pos255/trap29_ycoord3645 | Species in chip section: All
Changed to K. Pneumoniae (cy3, cy5) -- Labeltool output: Escherichia coli
488 cy3 cy5 txr pf 34 33 32 31 30 29 28 27 26 25 24 23 22 21 20 19 18 17 16 15 14 13 12 11 10 9 8 7 6 5 4 3 2 1 0

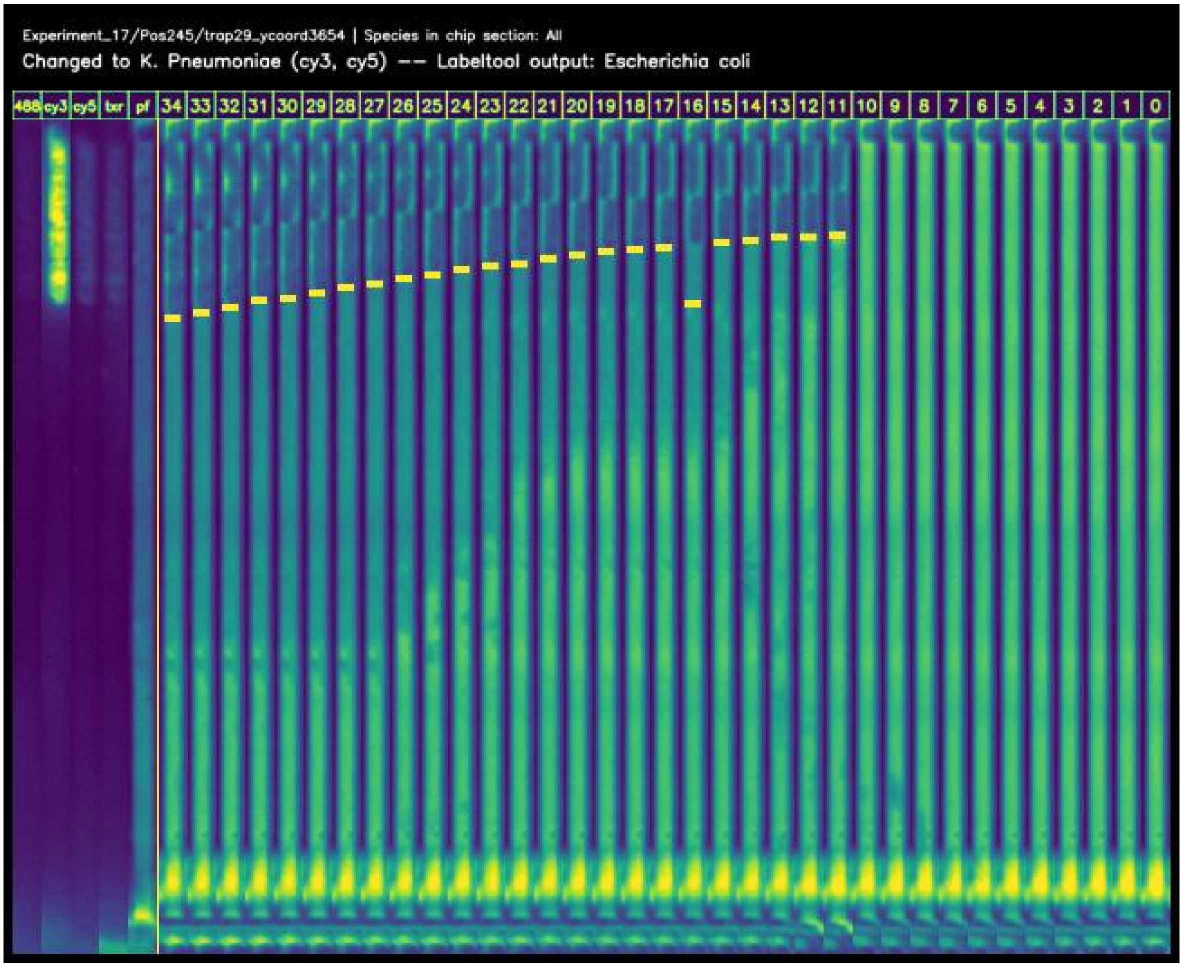
Experiment_17/Pos245/trap29_ycoord3654 | Species in chip section: All
Changed to K. Pneumoniae (cy3, cy5) -- Labeltool output: Escherichia coli
488 cy3 cy5 txr pf 34 33 32 31 30 29 28 27 26 25 24 23 22 21 20 19 18 17 16 15 14 13 12 11 10 9 8 7 6 5 4 3 2 1 0

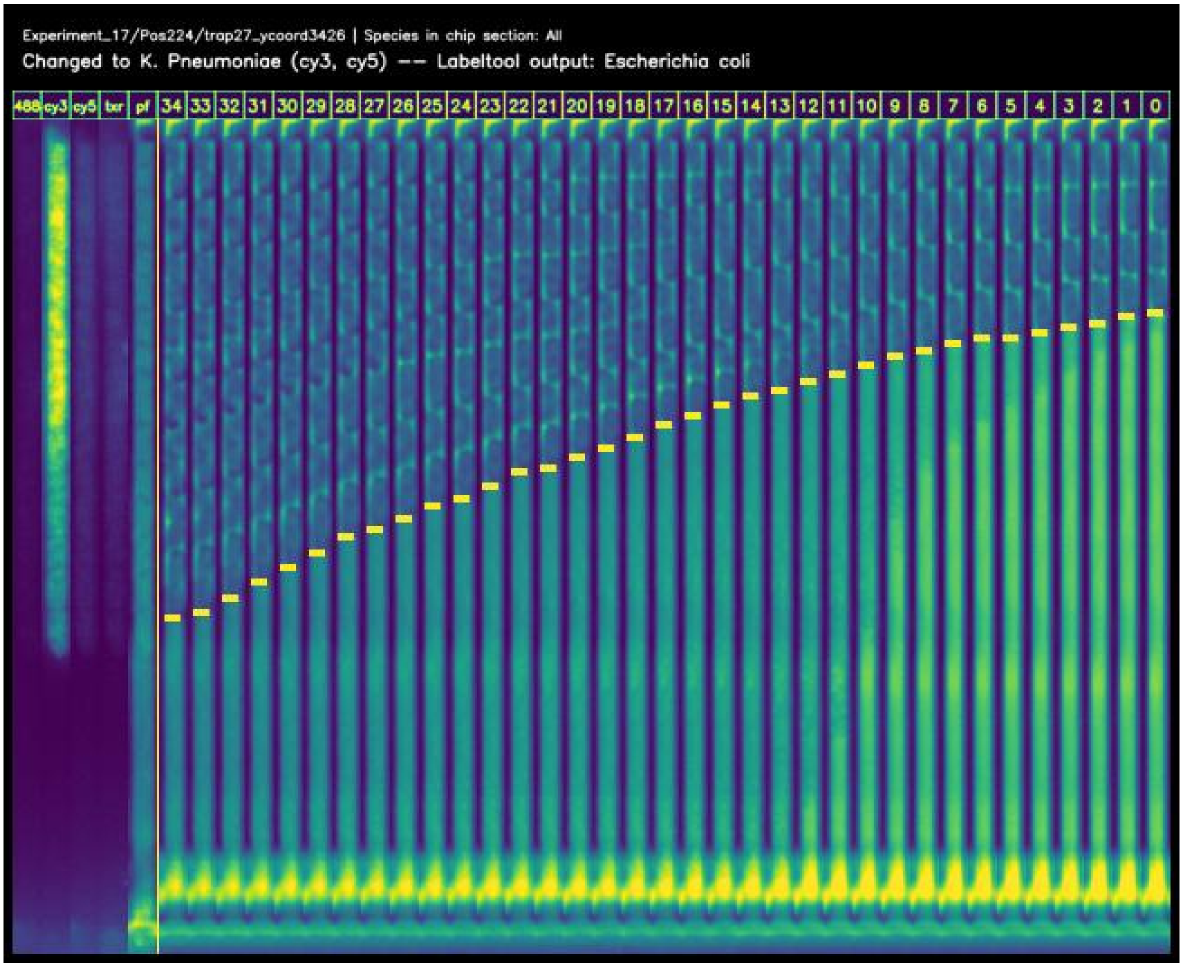
Experiment_17/Pos224/trap27_ycoord3426 | Species in chip section: All
Changed to K. Pneumoniae (cy3, cy5) -- Labeltool output: Escherichia coli
488 cy3 cy5 txr pf 34 33 32 31 30 29 28 27 26 25 24 23 22 21 20 19 18 17 16 15 14 13 12 11 10 9 8 7 6 5 4 3 2 1 0

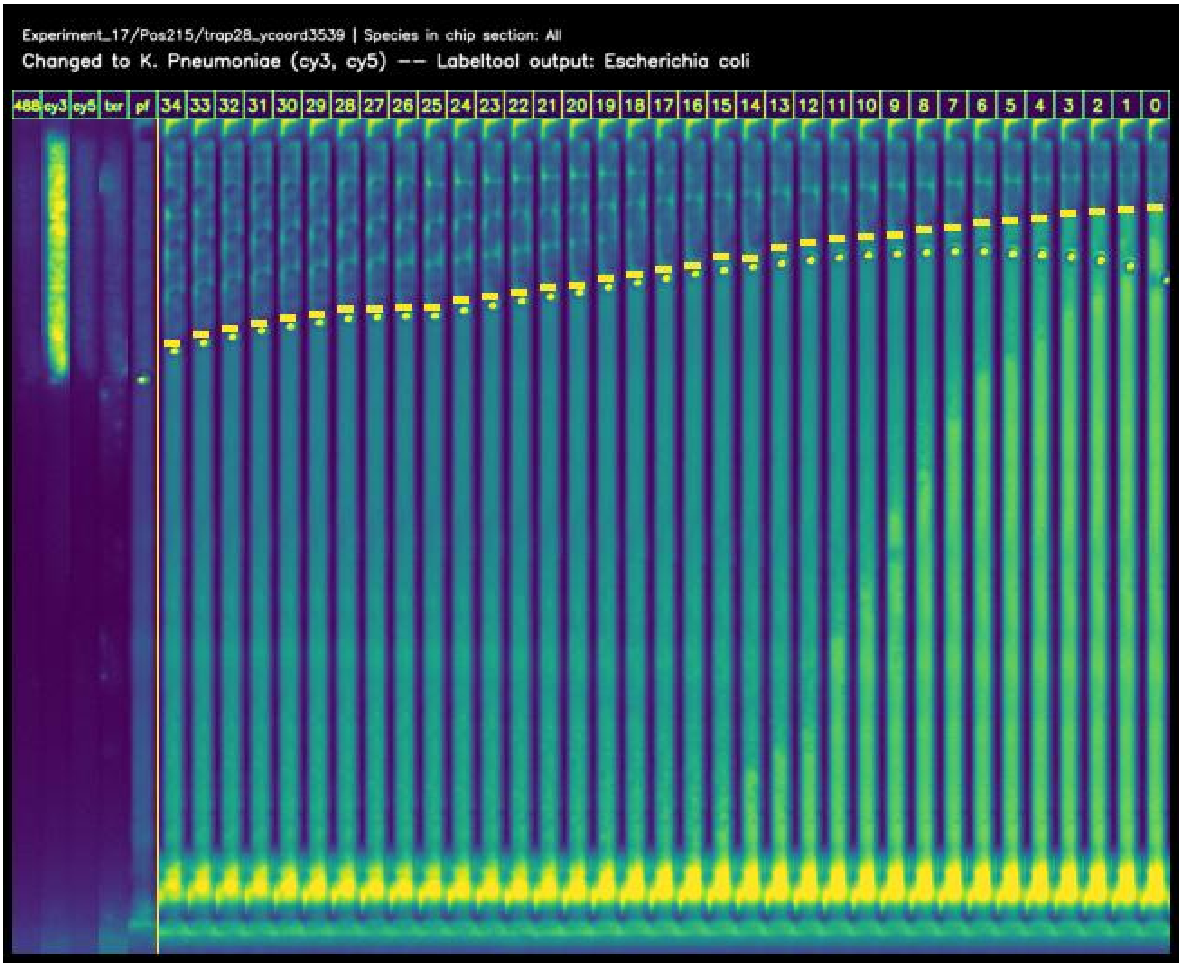
Experiment_17/Pos215/trap28_ycoord3539 | Species in chip section: All
Changed to K. Pneumoniae (cy3, cy5) -- Labeltool output: Escherichia coli
488 cy3 cy5 txr pf 34 33 32 31 30 29 28 27 26 25 24 23 22 21 20 19 18 17 16 15 14 13 12 11 10 9 8 7 6 5 4 3 2 1 0

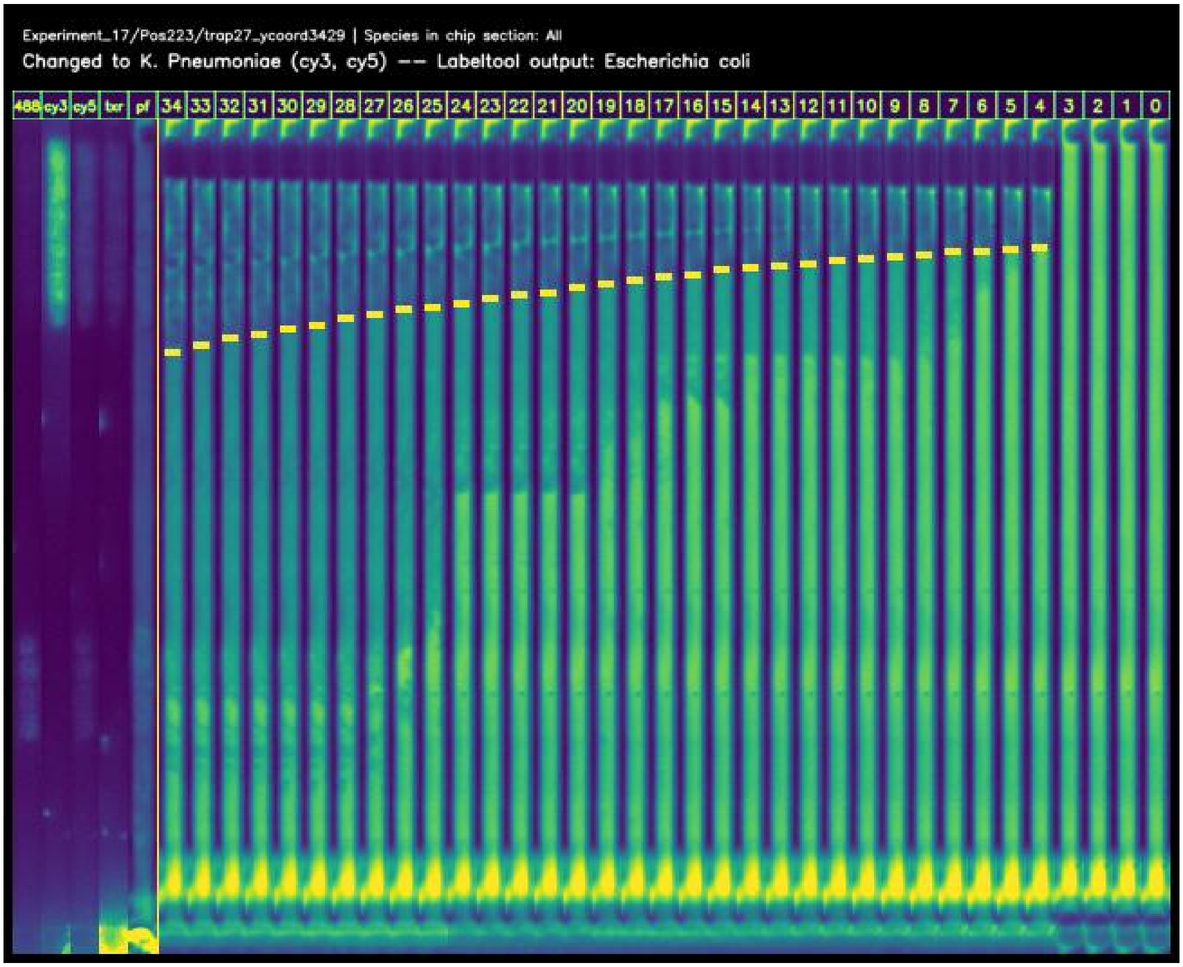
Experiment_17/Pos223/trap27_ycoord3429 | Species in chip section: All
Changed to K. Pneumoniae (cy3, cy5) -- Labeltool output: Escherichia coli
488 cy3 cy5 txr pf 34 33 32 31 30 29 28 27 26 25 24 23 22 21 20 19 18 17 16 15 14 13 12 11 10 9 8 7 6 5 4 3 2 1 0

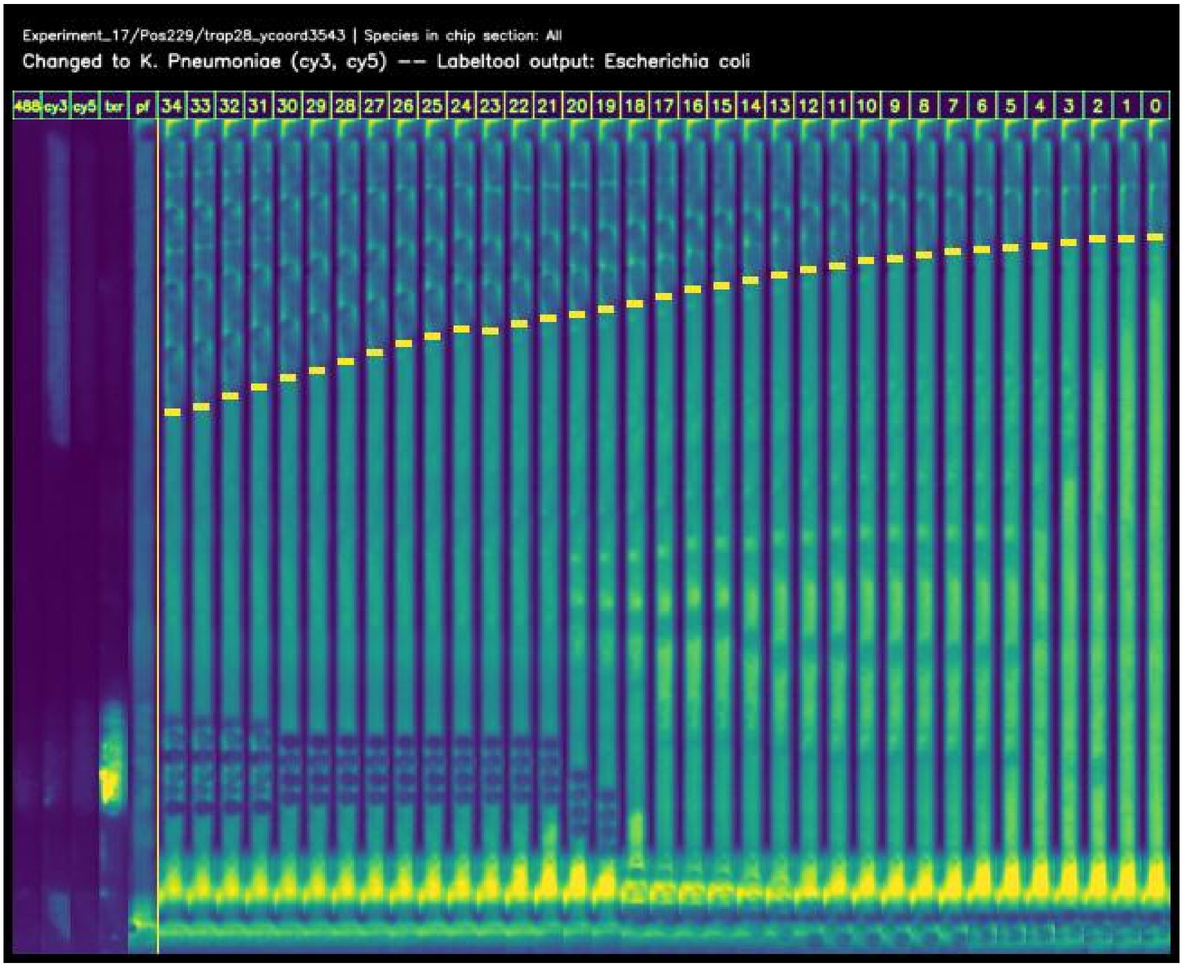
Experiment_17/Pos229/trap28_ycoord3543 | Species in chip section: All
Changed to K. Pneumoniae (cy3, cy5) -- Labeltool output: Escherichia coli
488 cy3 cy5 txr pf 34 33 32 31 30 29 28 27 26 25 24 23 22 21 20 19 18 17 16 15 14 13 12 11 10 9 8 7 6 5 4 3 2 1 0

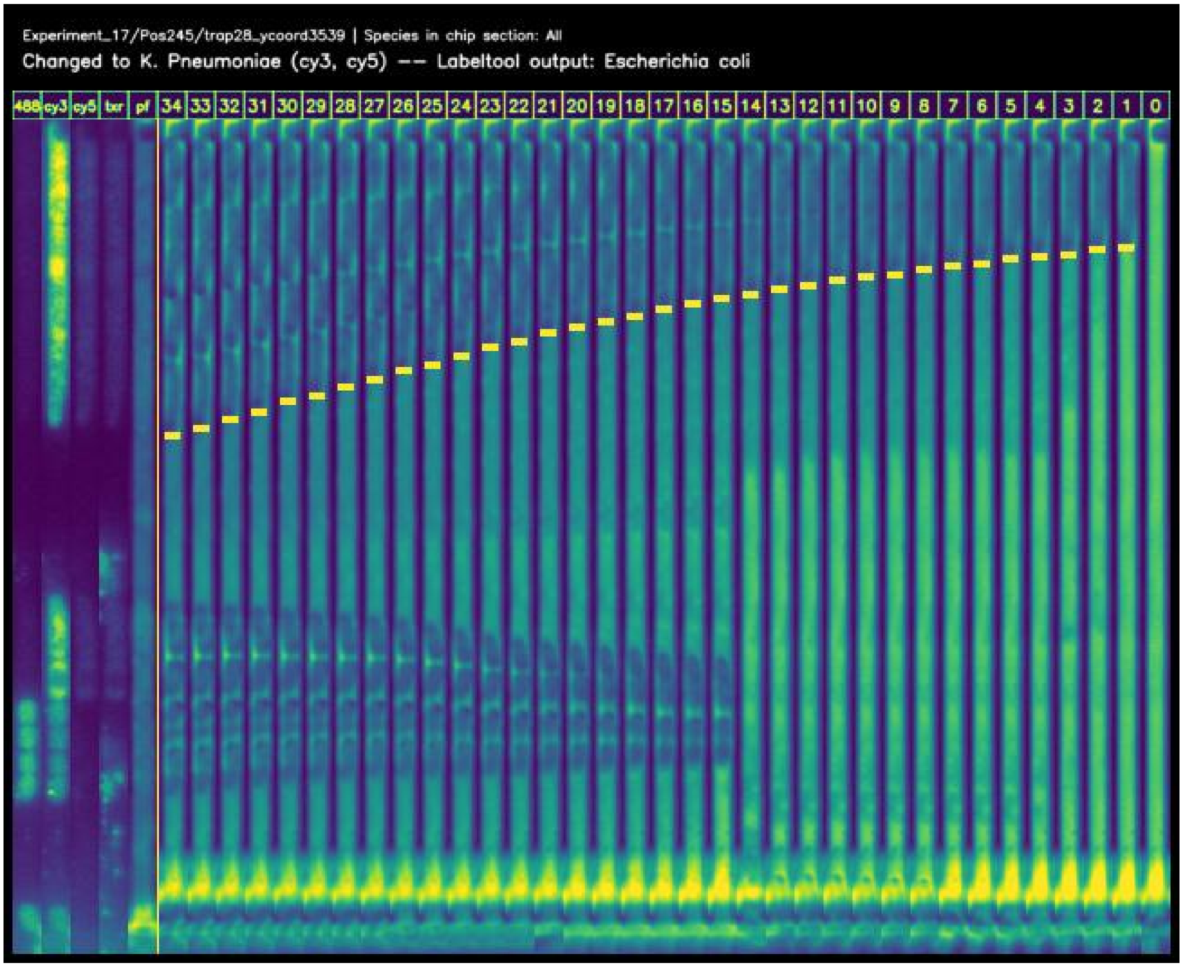
Experiment_17/Pos245/trap28_ycoord3539 | Species in chip section: All
Changed to K. Pneumoniae (cy3, cy5) -- Labeltool output: Escherichia coli
488 cy3 cy5 txr pf 34 33 32 31 30 29 28 27 26 25 24 23 22 21 20 19 18 17 16 15 14 13 12 11 10 9 8 7 6 5 4 3 2 1 0

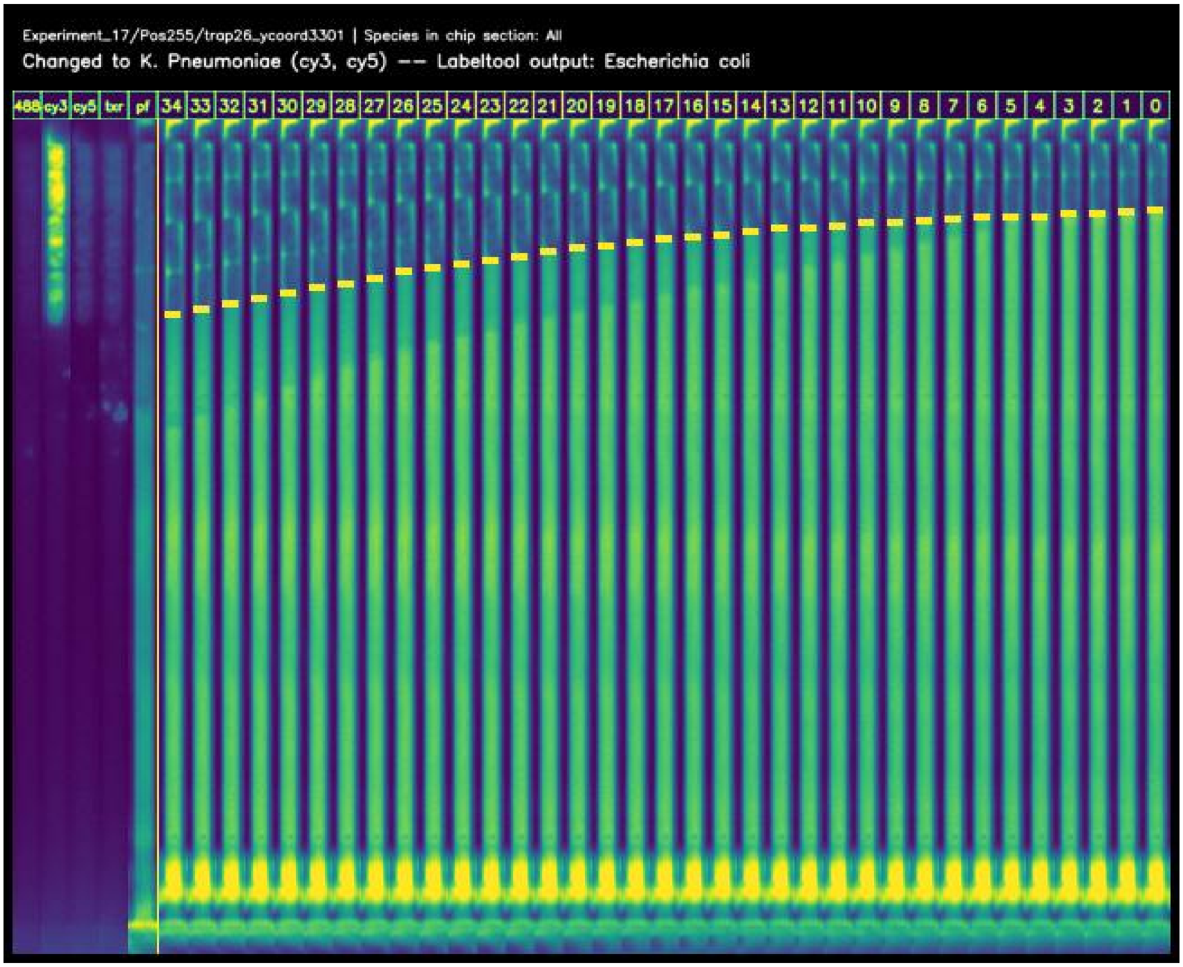
Experiment_17/Pos255/trap26_ycoord3301 | Species in chip section: All
Changed to K. Pneumoniae (cy3, cy5) -- Labeltool output: Escherichia coli
488 cy3 cy5 txr pf 34 33 32 31 30 29 28 27 26 25 24 23 22 21 20 19 18 17 16 15 14 13 12 11 10 9 8 7 6 5 4 3 2 1 0

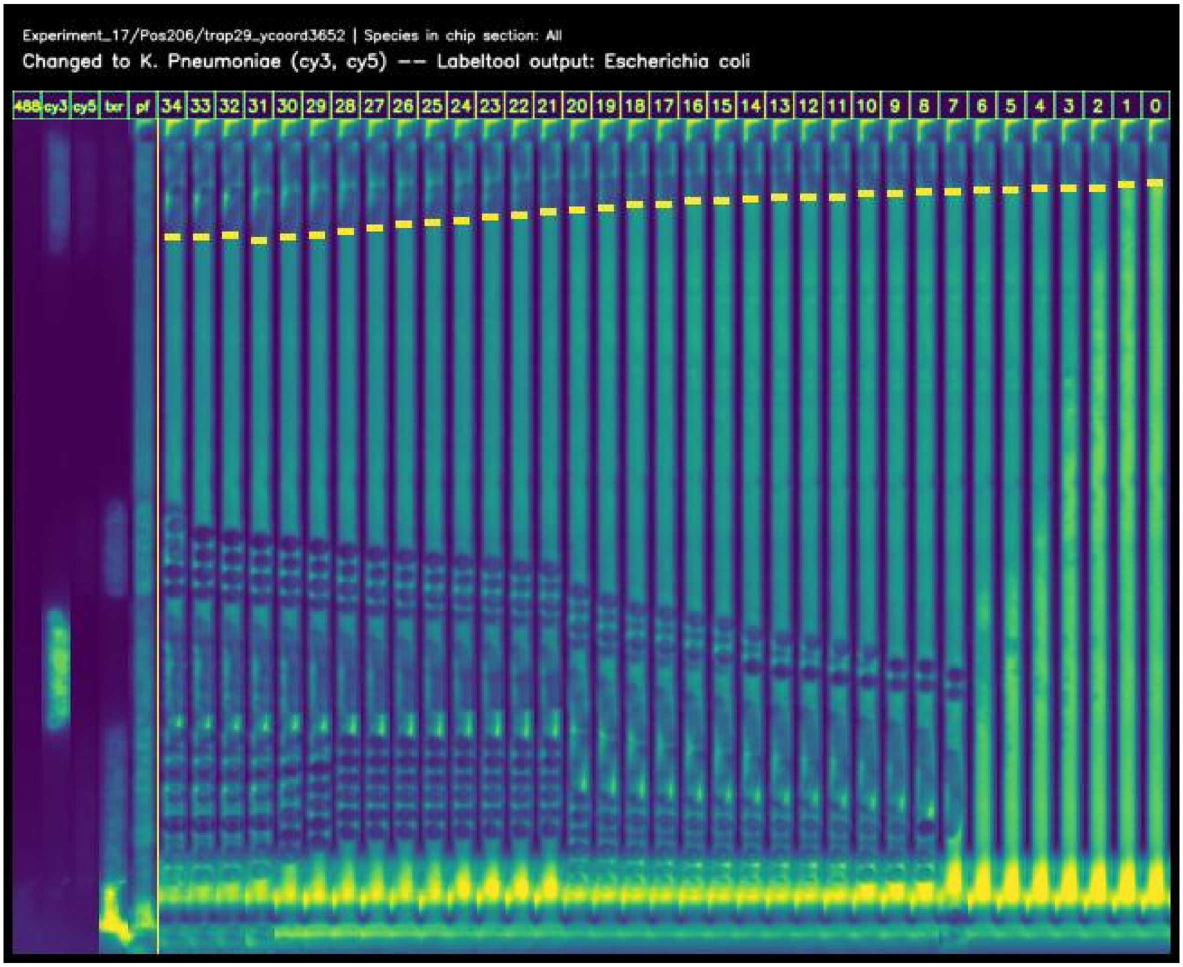

Experiment_17/Pos206/trap29_ycoord3652 | Species in chip section: All
Changed to K. Pneumoniae (cy3, cy5) -- Labeltool output: Escherichia coli
488 cy3 cy5 txr pf 34 33 32 31 30 29 28 27 26 25 24 23 22 21 20 19 18 17 16 15 14 13 12 11 10 9 8 7 6 5 4 3 2 1 0

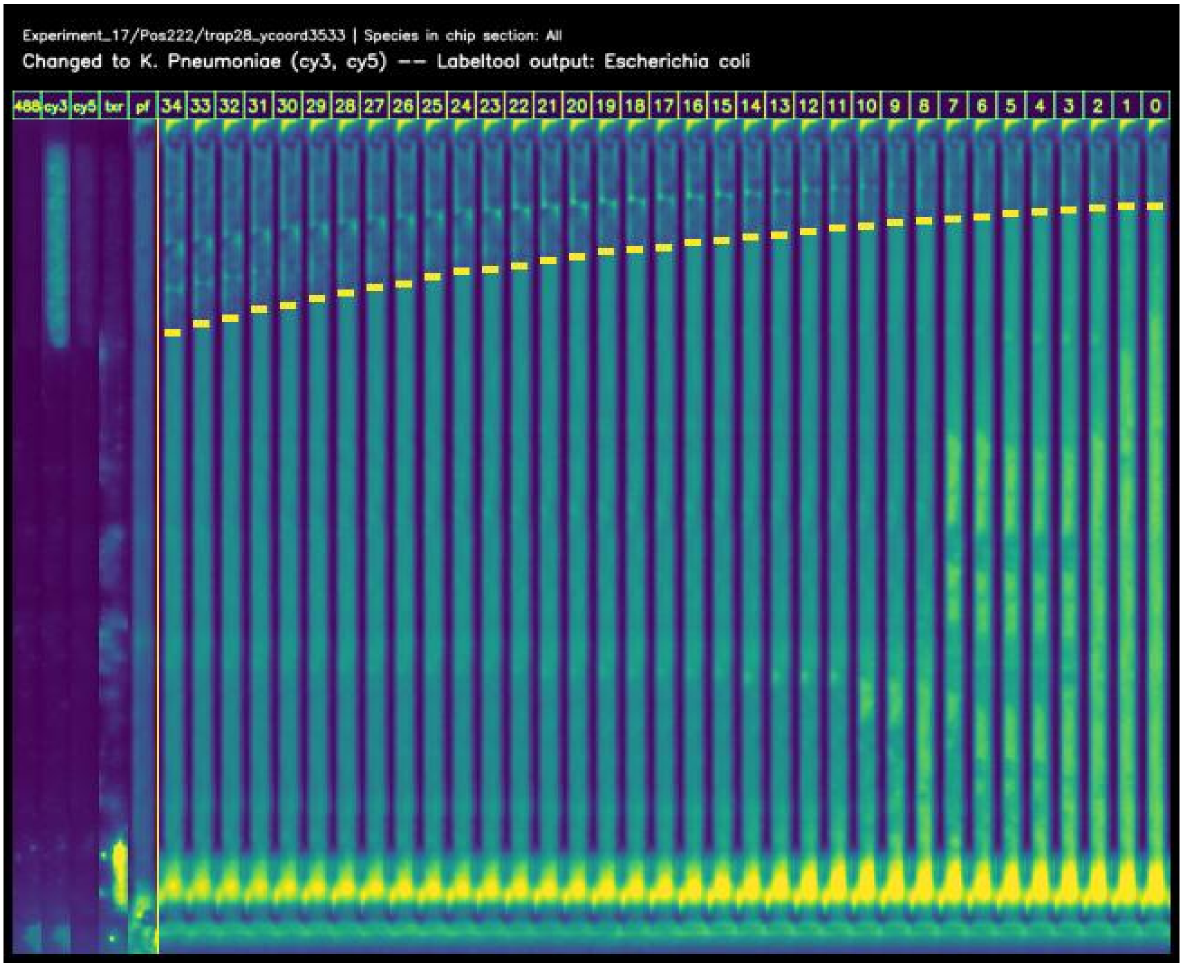

Experiment_17/Pos222/trap28_ycoord3533 | Species in chip section: All
Changed to K. Pneumoniae (cy3, cy5) -- Labeltool output: Escherichia coli
488 cy3 cy5 txr pf 34 33 32 31 30 29 28 27 26 25 24 23 22 21 20 19 18 17 16 15 14 13 12 11 10 9 8 7 6 5 4 3 2 1 0

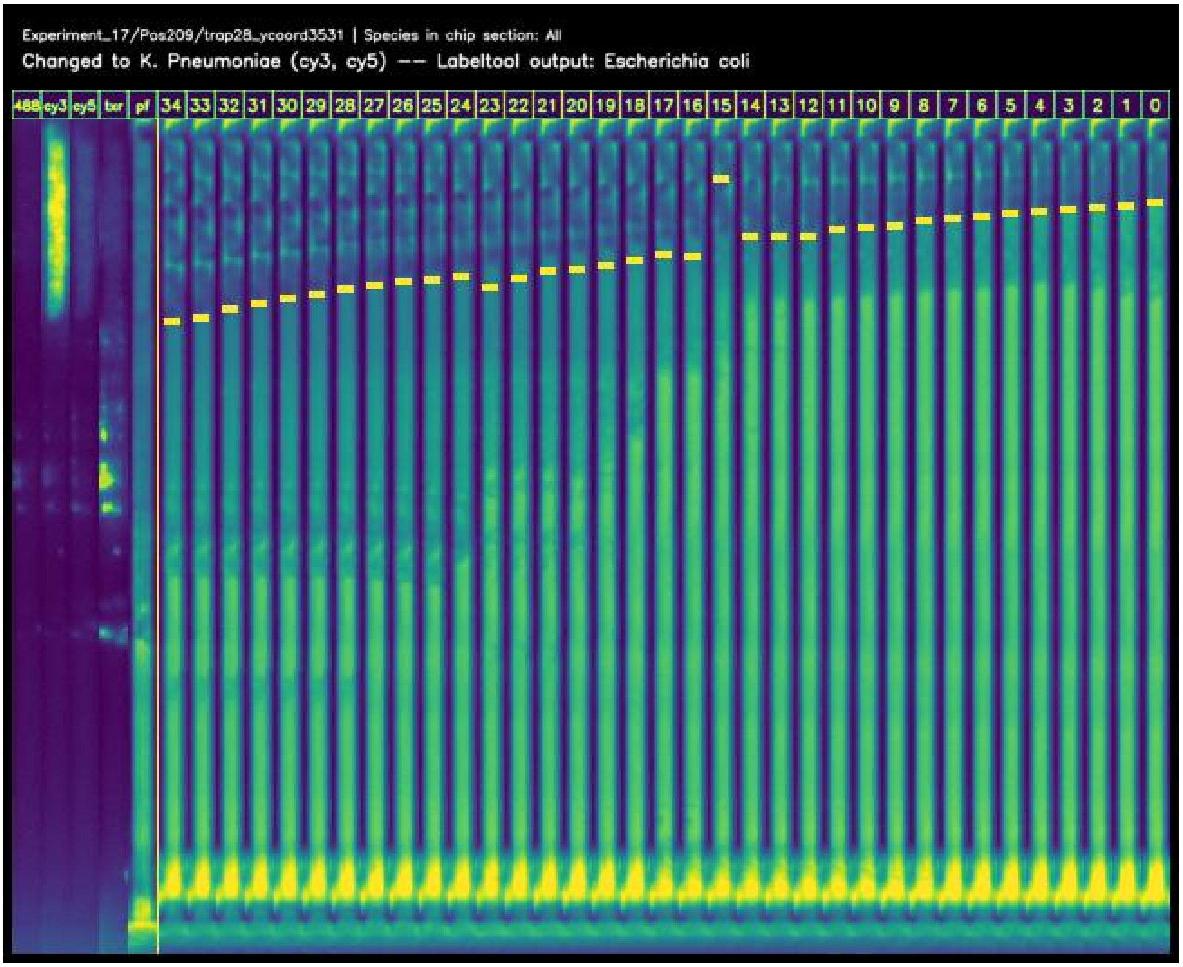
Experiment_17/Pos209/trap28_ycoord3531 | Species in chip section: All
Changed to K. Pneumoniae (cy3, cy5) -- Labeltool output: Escherichia coli
488 cy3 cy5 bxr pf 34 33 32 31 30 29 28 27 26 25 24 23 22 21 20 19 18 17 16 15 14 13 12 11 10 9 8 7 6 5 4 3 2 1 0

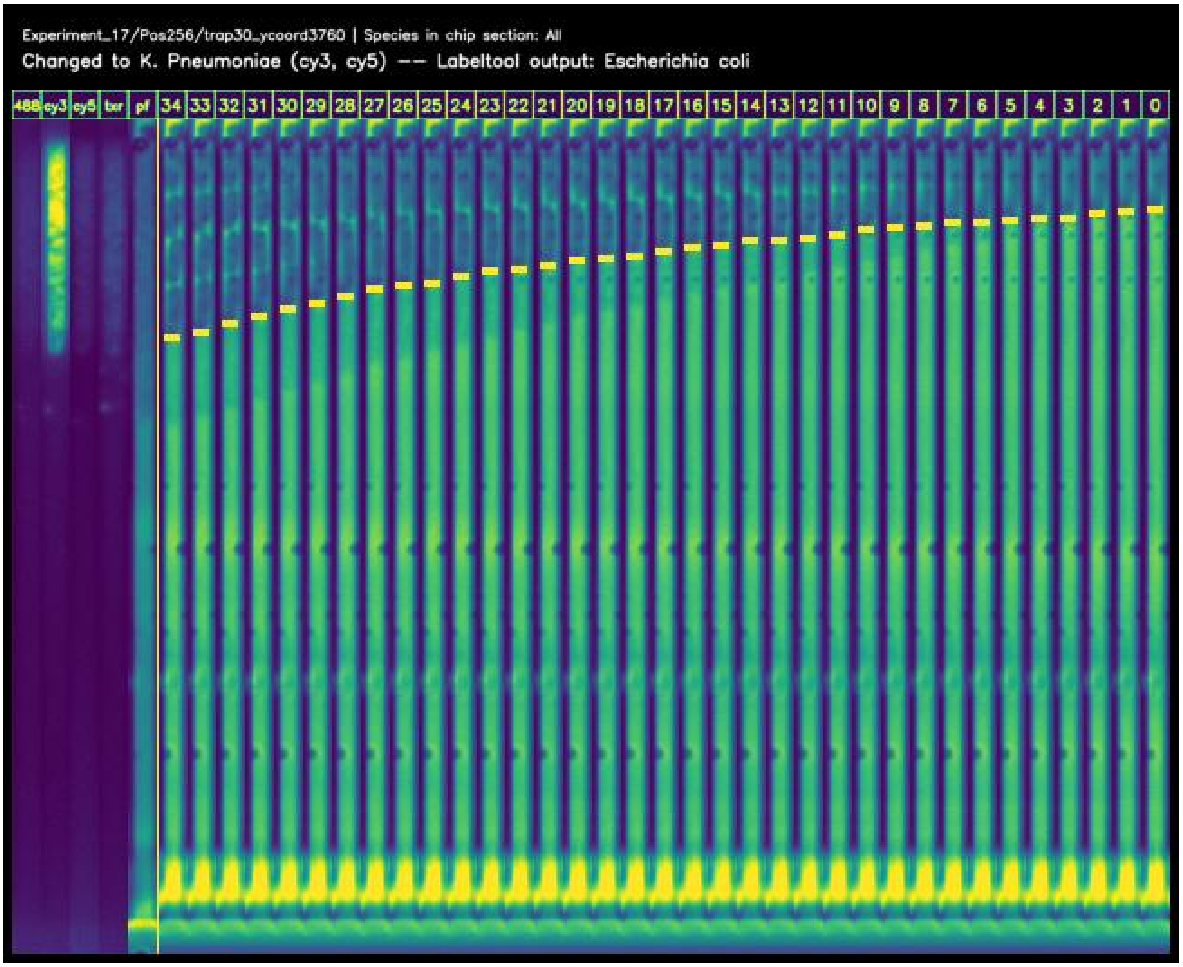
Experiment_17/Pos256/trap30_ycoord3760 | Species in chip section: All
Changed to K. Pneumoniae (cy3, cy5) -- Labeltool output: Escherichia coli
488 cy3 cy5 bxr pf 34 33 32 31 30 29 28 27 26 25 24 23 22 21 20 19 18 17 16 15 14 13 12 11 10 9 8 7 6 5 4 3 2 1 0

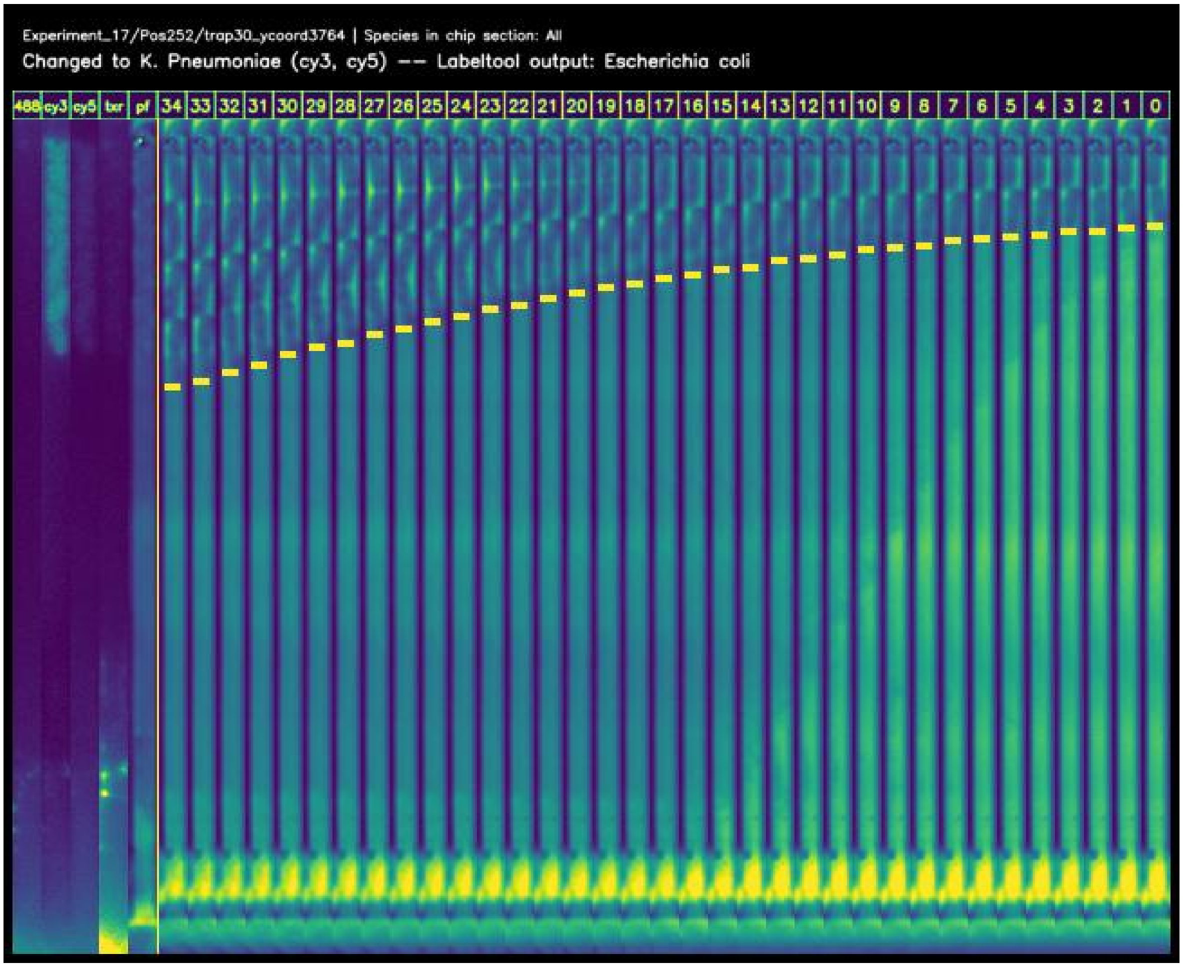
Experiment_17/Pos252/trap30_ycoord3764 | Species in chip section: All
Changed to K. Pneumoniae (cy3, cy5) -- Labeltool output: Escherichia coli
488 cy3 cy5 txr pf 34 33 32 31 30 29 28 27 26 25 24 23 22 21 20 19 18 17 16 15 14 13 12 11 10 9 8 7 6 5 4 3 2 1 0

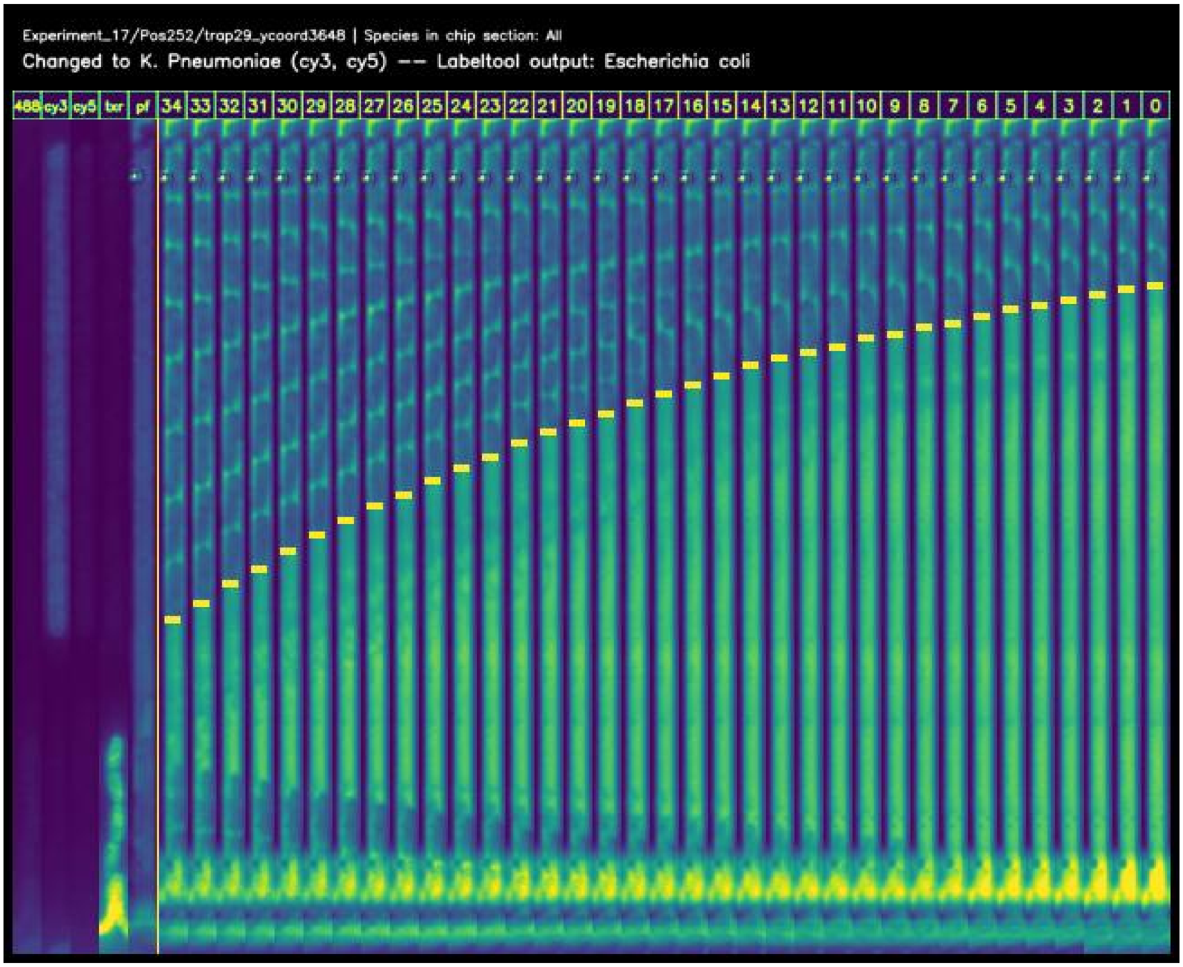
Experiment_17/Pos252/trap29_ycoord3648 | Species in chip section: All
Changed to K. Pneumoniae (cy3, cy5) -- Labeltool output: Escherichia coli
488 cy3 cy5 txr pf 34 33 32 31 30 29 28 27 26 25 24 23 22 21 20 19 18 17 16 15 14 13 12 11 10 9 8 7 6 5 4 3 2 1 0

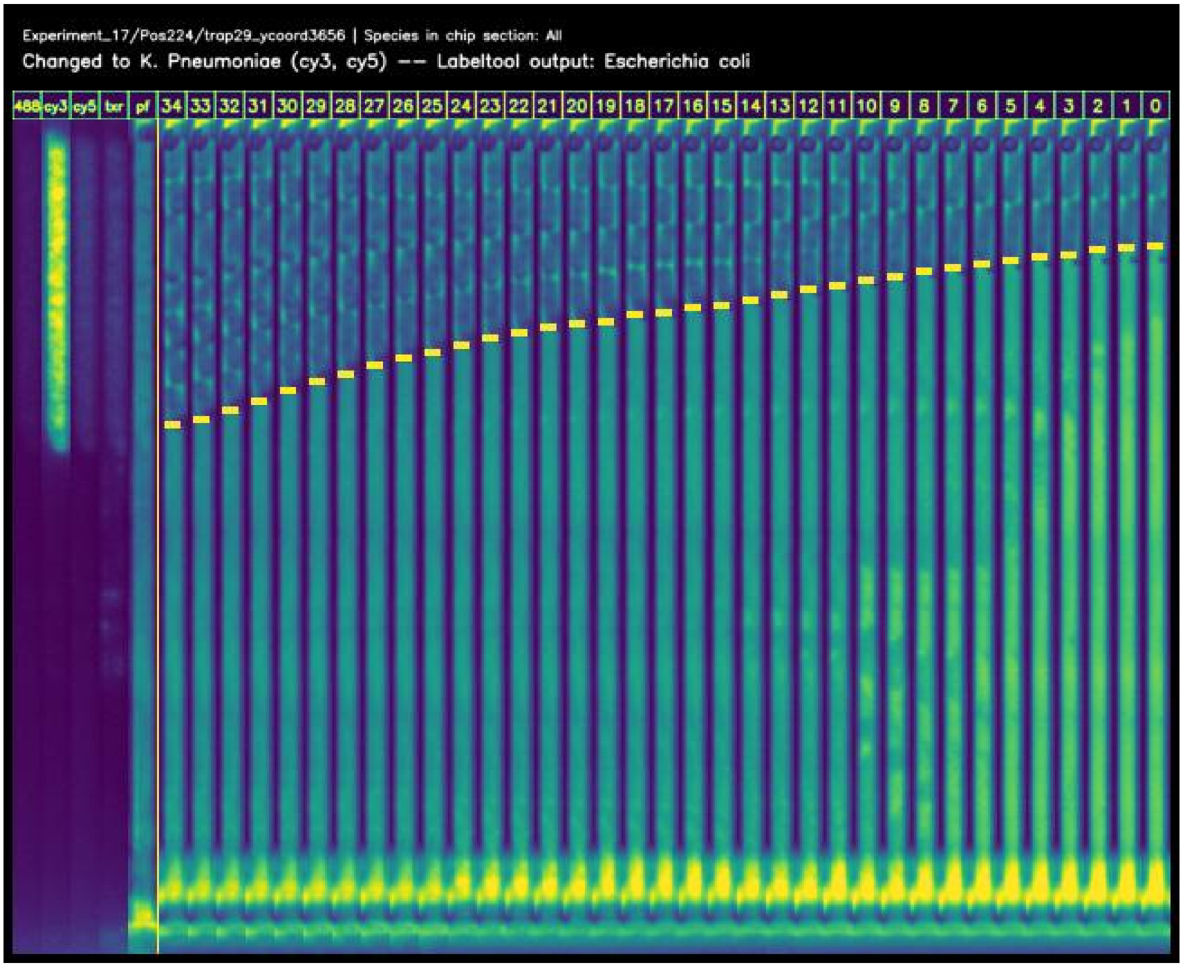
Experiment_17/Pos224/trap29_ycoord3656 | Species in chip section: All
Changed to K. Pneumoniae (cy3, cy5) -- Labeltool output: Escherichia coli
488 cy3 cy5 txr pf 34 33 32 31 30 29 28 27 26 25 24 23 22 21 20 19 18 17 16 15 14 13 12 11 10 9 8 7 6 5 4 3 2 1 0

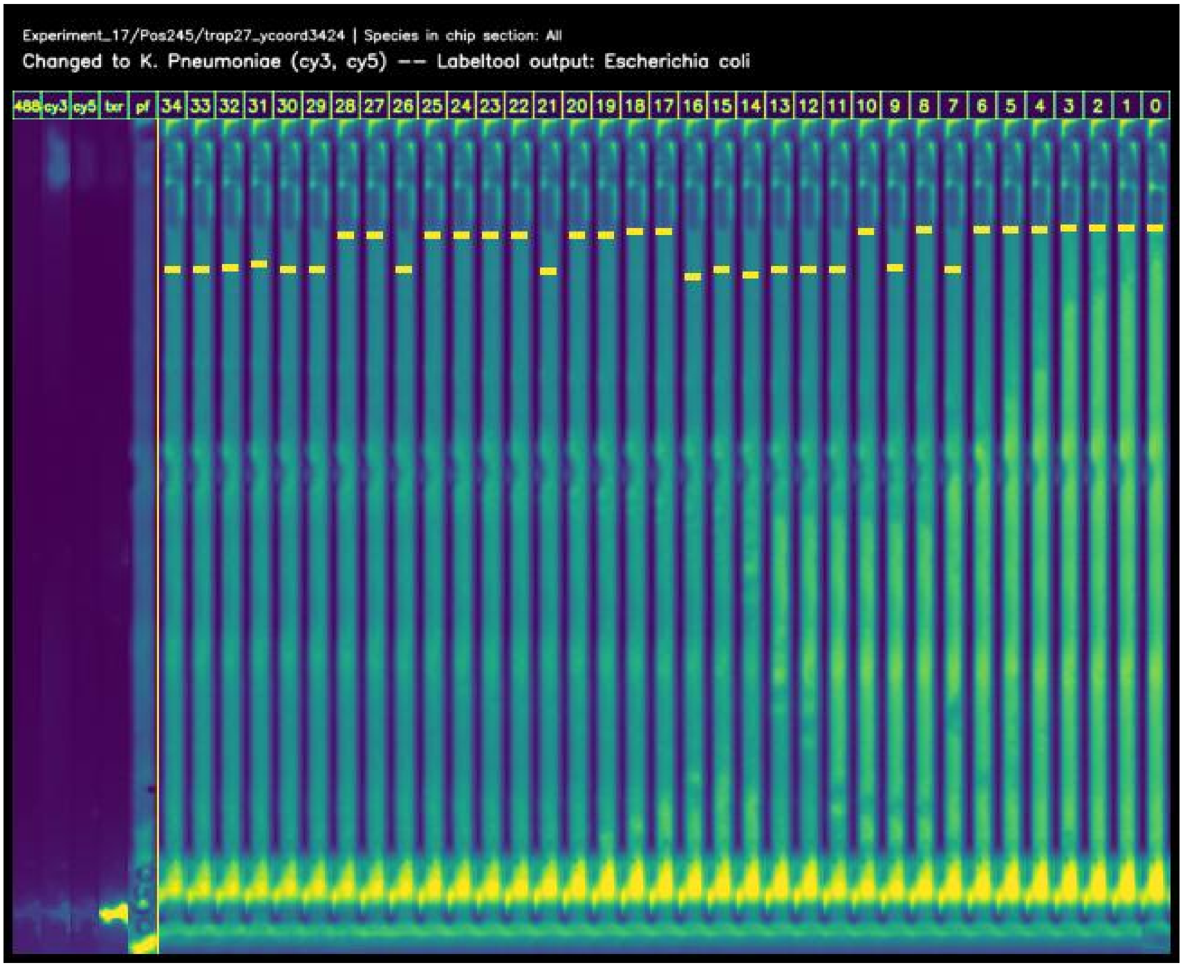
Experiment_17/Pos245/trap27_ycoord3424 | Species in chip section: All
Changed to K. Pneumoniae (cy3, cy5) -- Labeltool output: Escherichia coli
488 cy3 cy5 txr pf 34 33 32 31 30 29 28 27 26 25 24 23 22 21 20 19 18 17 16 15 14 13 12 11 10 9 8 7 6 5 4 3 2 1 0

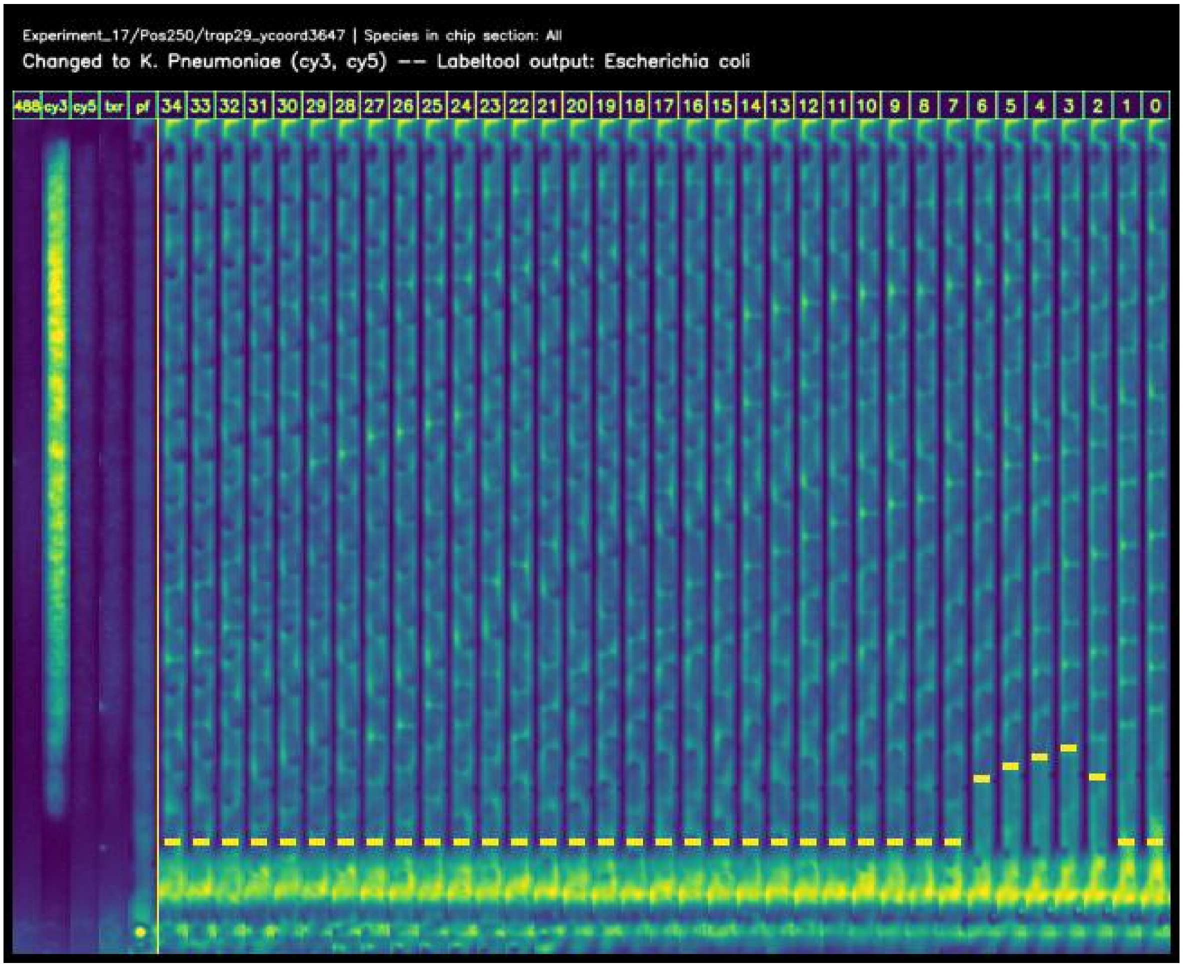
Experiment_17/Pos250/trap29_ycoord3647 | Species in chip section: All
Changed to K. Pneumoniae (cy3, cy5) -- Labeltool output: Escherichia coli
488 cy3 cy5 txr pf 34 33 32 31 30 29 28 27 26 25 24 23 22 21 20 19 18 17 16 15 14 13 12 11 10 9 8 7 6 5 4 3 2 1 0

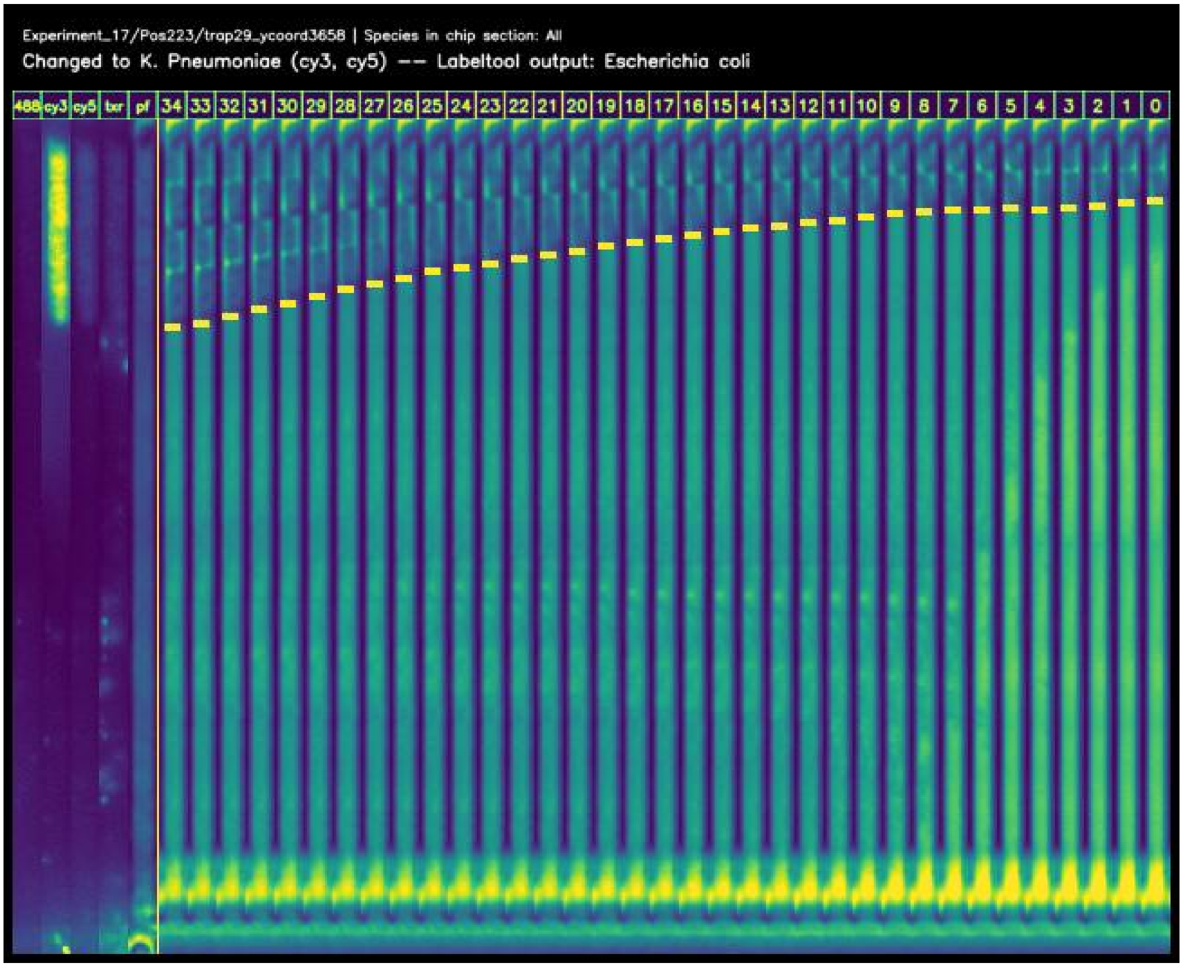
Experiment_17/Pos223/trap29_ycoord3658 | Species in chip section: All
Changed to K. Pneumoniae (cy3, cy5) -- Labeltool output: Escherichia coli
488 cy3 cy5 txr pf 34 33 32 31 30 29 28 27 26 25 24 23 22 21 20 19 18 17 16 15 14 13 12 11 10 9 8 7 6 5 4 3 2 1 0

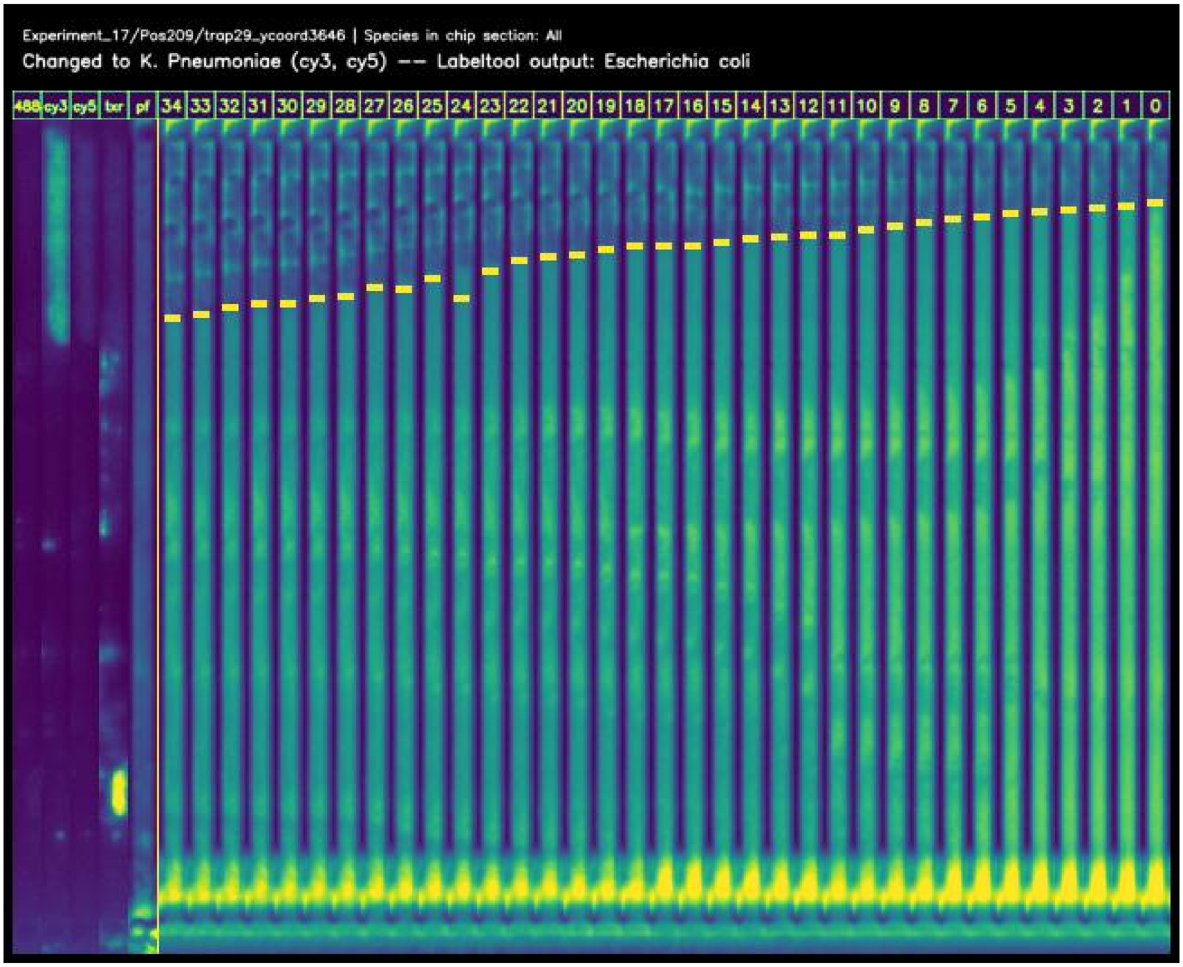
Experiment_17/Pos209/trap29_ycoord3646 | Species in chip section: All
Changed to K. Pneumoniae (cy3, cy5) -- Labeltool output: Escherichia coli
488
cy3
cy5
txr
pf
34 33 32 31 30 29 28 27 26 25 24 23 22 21 20 19 18 17 16 15 14 13 12 11 10 9 8 7 6 5 4 3 2 1 0

Changed to ***P. Mirabilis***

The following trap was changed to *P. Mirabilis*, the labeling tool misidentified the sample due to background noise in the Cy3 channel.

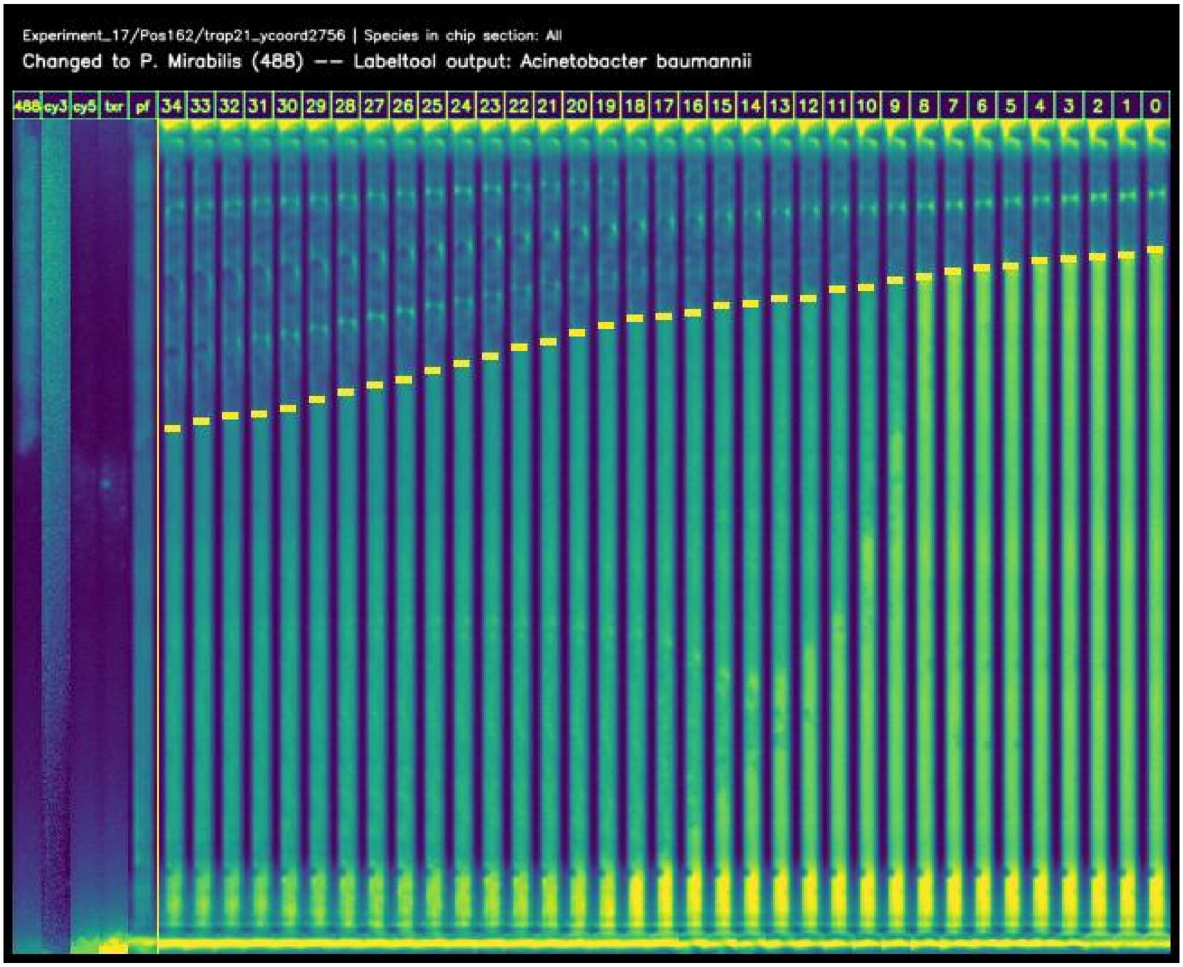

Changed to ***P. Aeruginosa***

The following traps were changed to *P. Aeruginosa*, the labeling tool made an error due to low absorption in the Alexa488 channel.

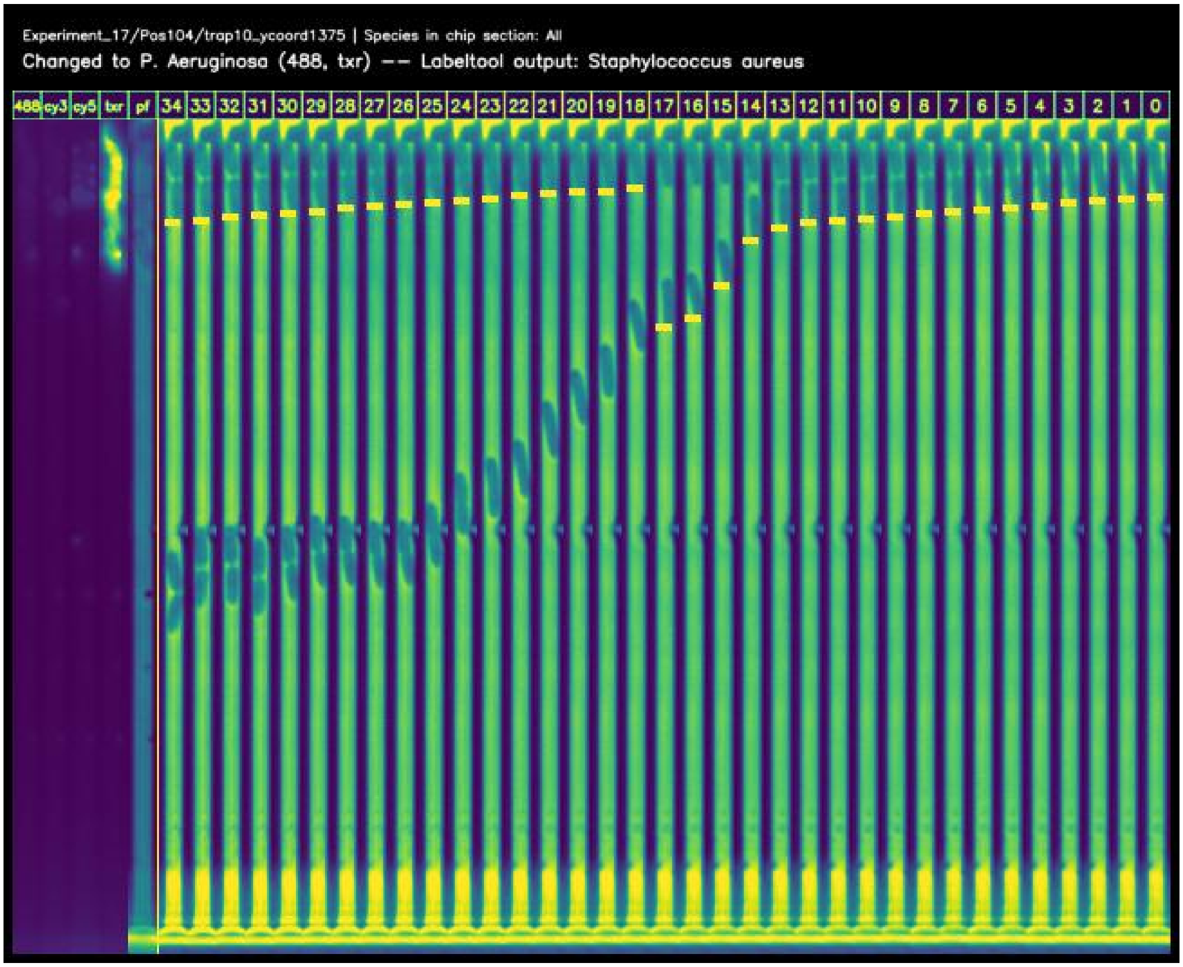

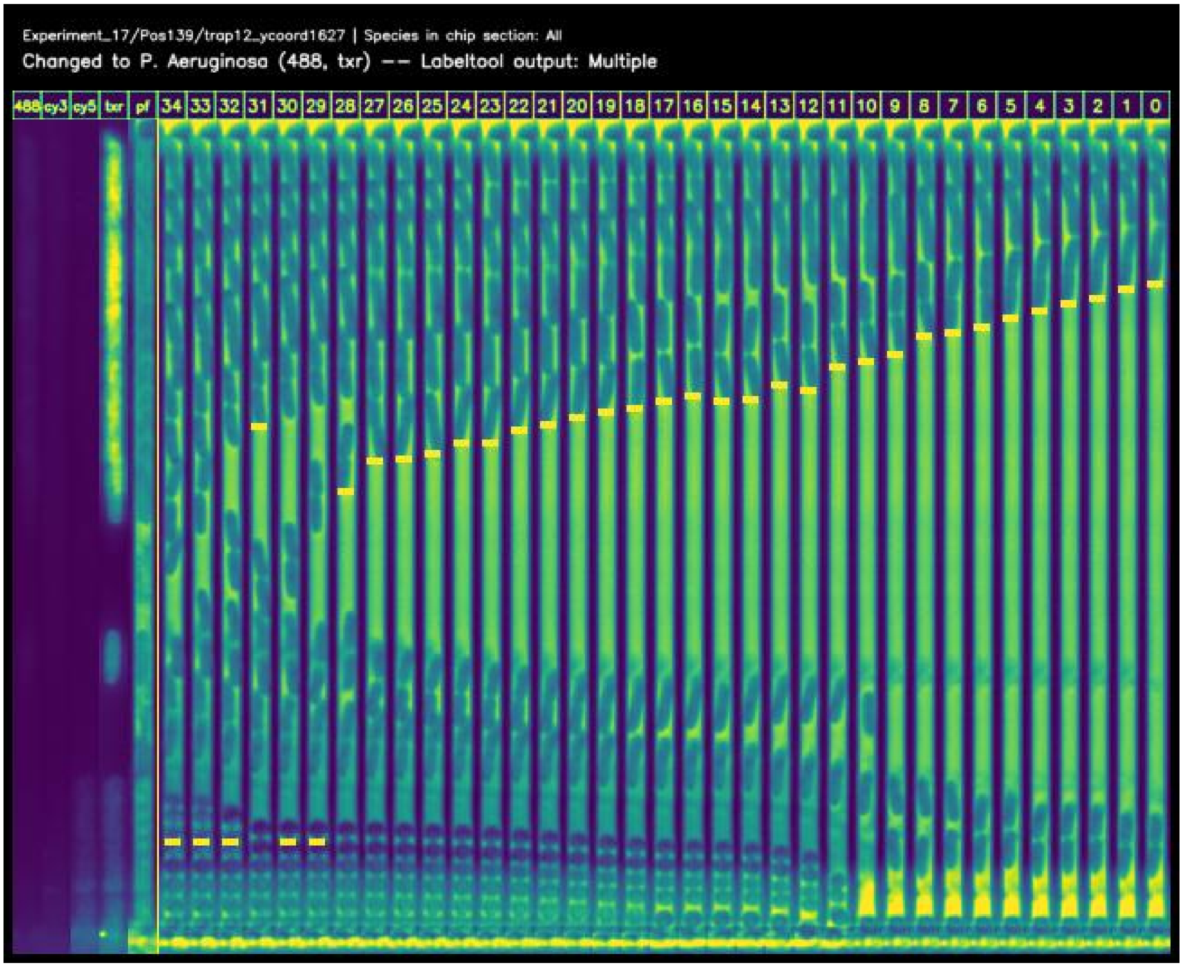
Experiment_17/Pos139/trap12_ycoord1627 | Species in chip section: All
Changed to P. Aeruginosa (488, txr) -- Labeltool output: Multiple
488 cy3 cy5 txr pf 34 33 32 31 30 29 28 27 26 25 24 23 22 21 20 19 18 18 17 16 15 14 13 12 11 10 9 8 7 6 5 4 3 2 1 0

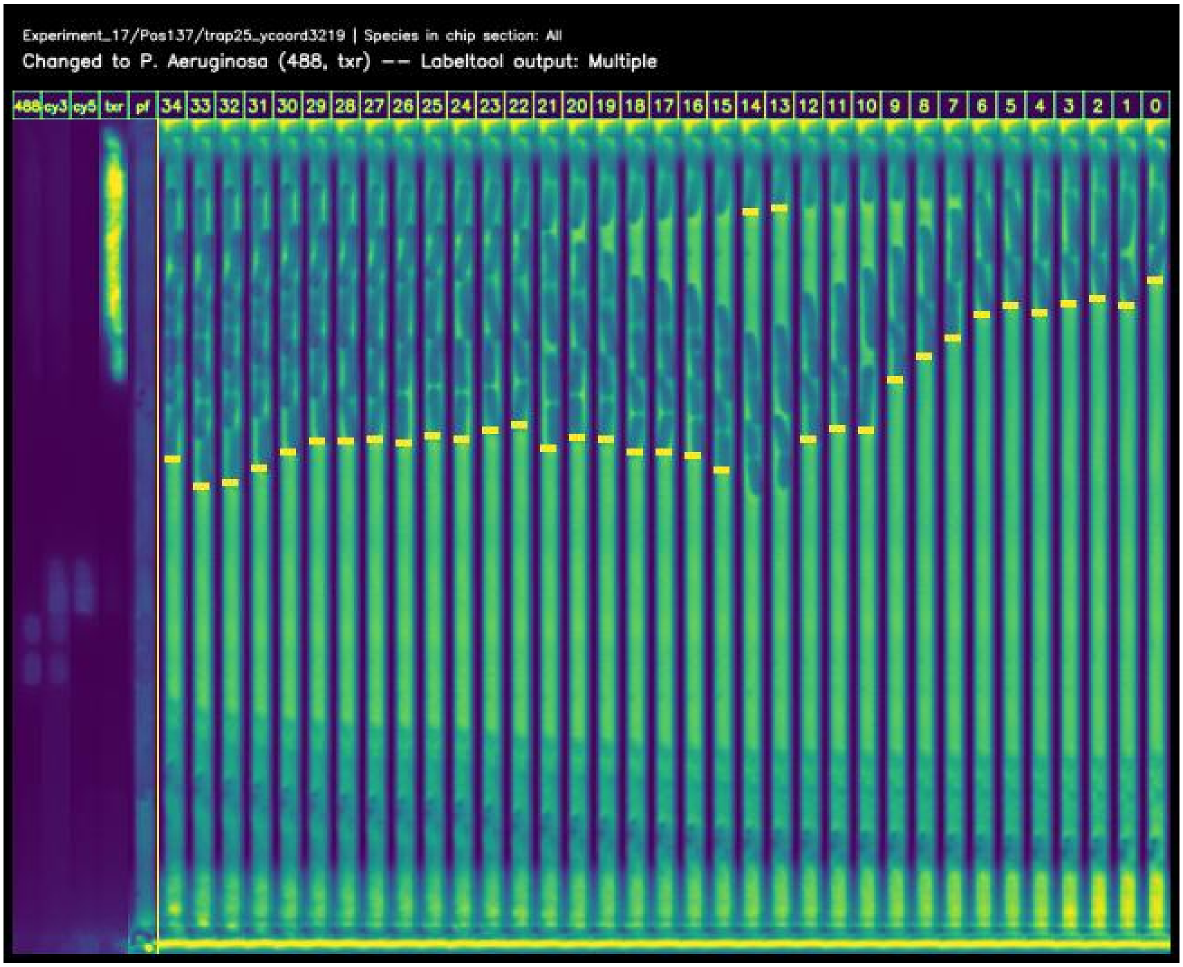
Experiment_17/Pos137/trap25_ycoord3219 | Species in chip section: All
Changed to P. Aeruginosa (488, txr) -- Labeltool output: Multiple
488 cy3 cy5 txr pf 34 33 32 31 30 29 28 27 26 25 24 23 22 21 20 19 18 18 17 16 15 14 13 12 11 10 9 8 7 6 5 4 3 2 1 0

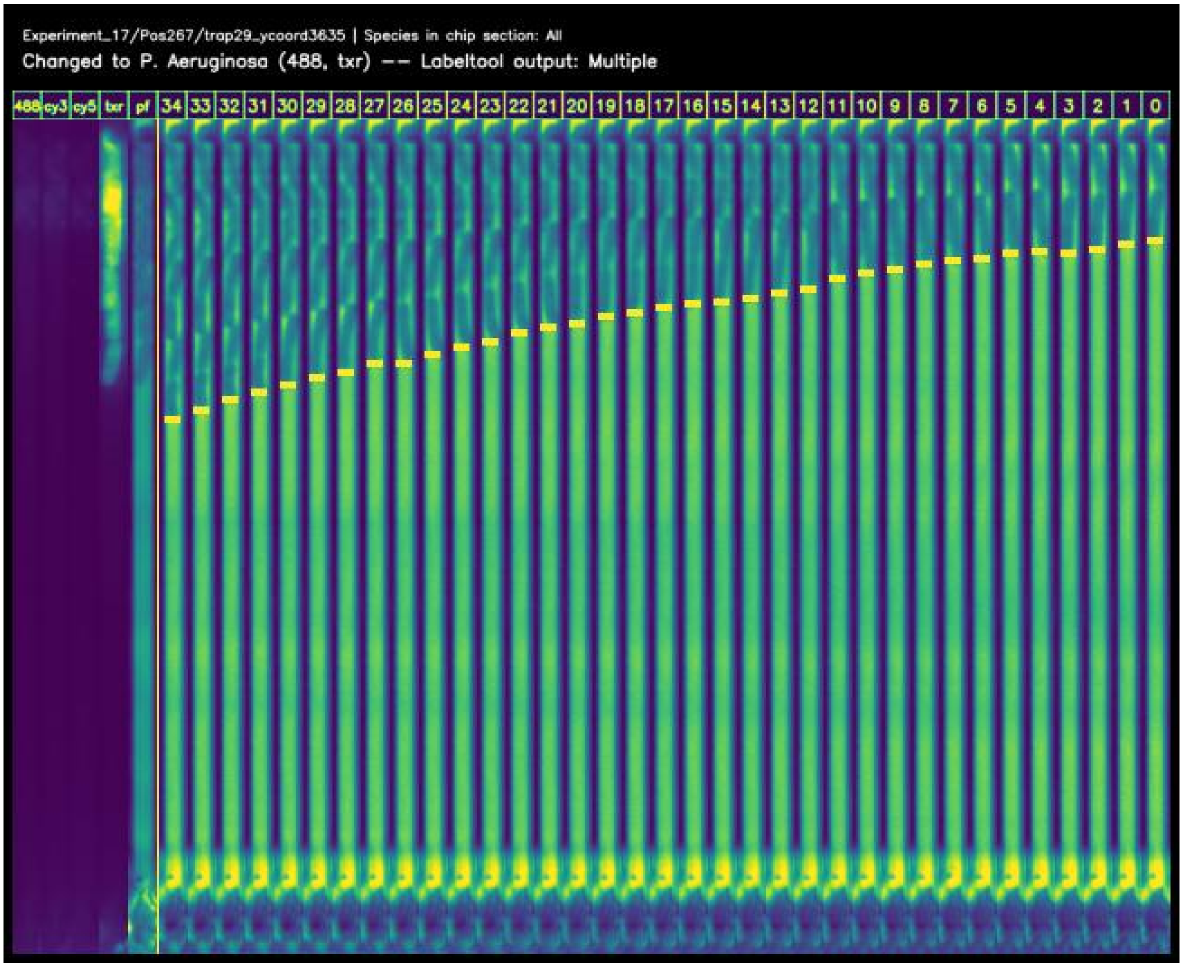
Experiment_17/Pos267/trap29_ycoord3635 | Species in chip section: All
Changed to P. Aeruginosa (488, txr) -- Labeltool output: Multiple
488 cy3 cy5 txr pf 34 33 32 31 30 29 28 27 26 25 24 23 22 21 20 19 18 17 16 15 14 13 12 11 10 9 8 7 6 5 4 3 2 1 0

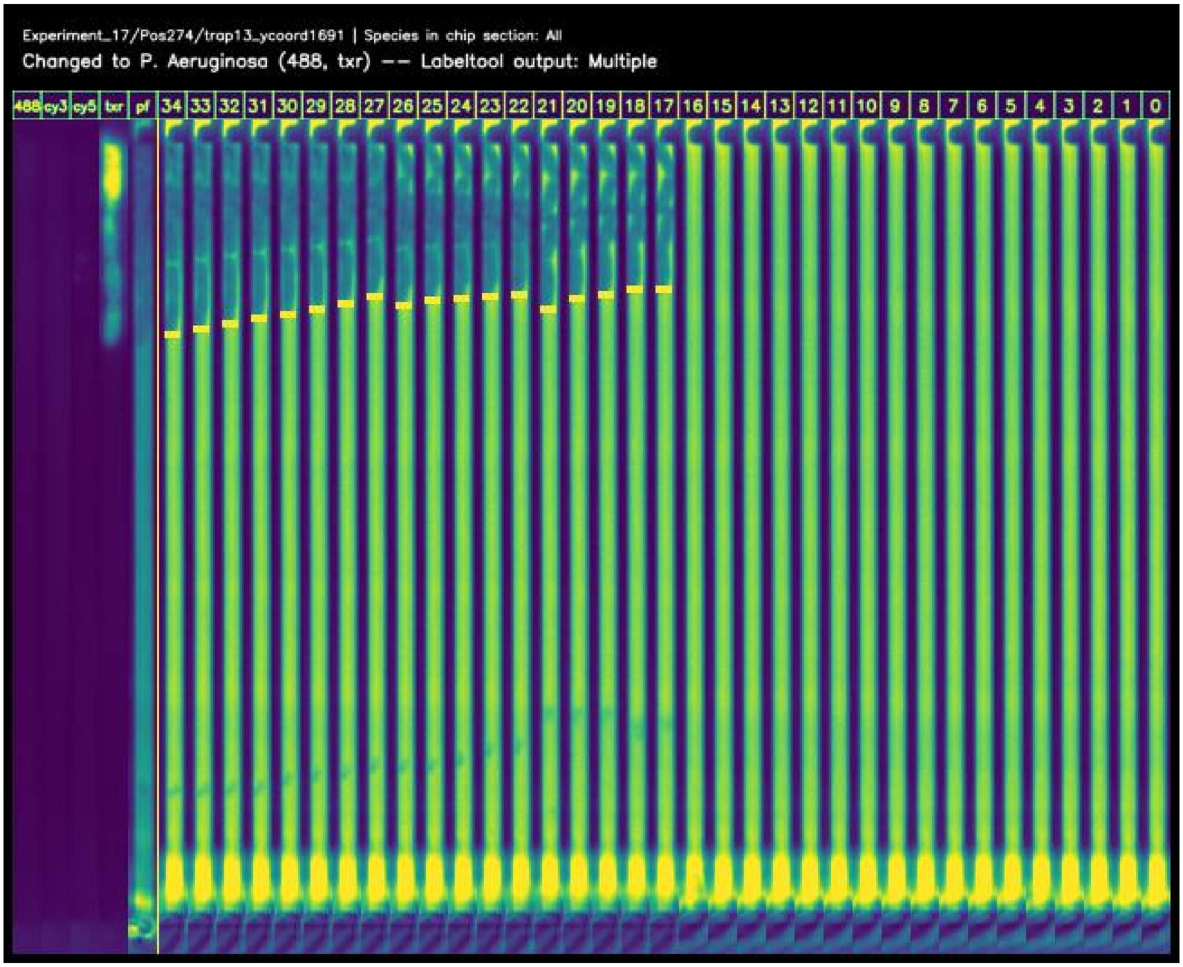
Experiment_17/Pos274/trap13_ycoord1691 | Species in chip section: All
Changed to P. Aeruginosa (488, txr) -- Labeltool output: Multiple
488 cy3 cy5 txr pf 34 33 32 31 30 29 28 27 26 25 24 23 22 21 20 19 18 17 16 15 14 13 12 11 10 9 8 7 6 5 4 3 2 1 0

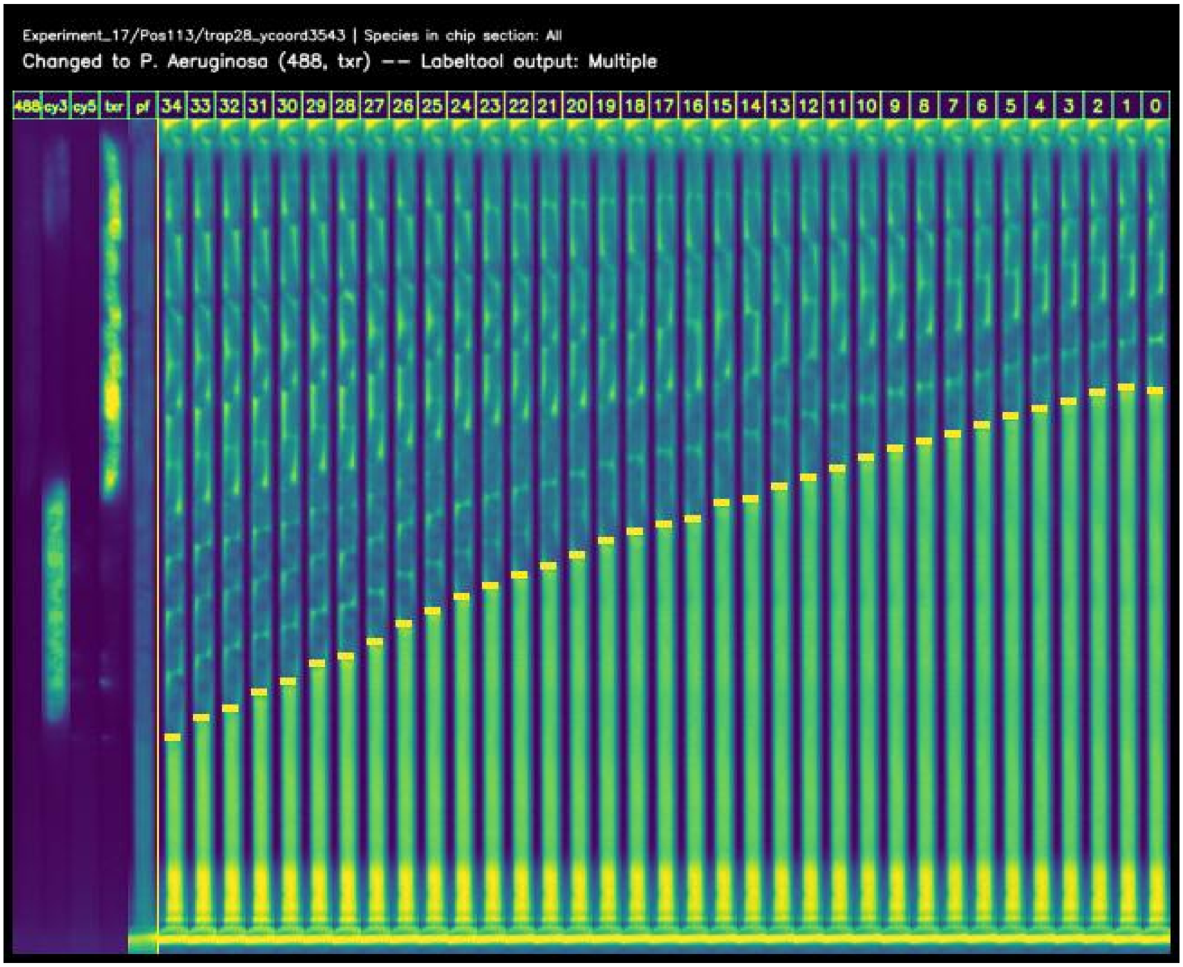
Experiment_17/Pos113/trap28_ycoord3543 | Species in chip section: All
Changed to P. Aeruginosa (488, txr) -- Labeltool output: Multiple
488 cy3 cy5 txr pf 34 33 32 31 30 29 28 27 26 25 24 23 22 21 20 19 18 17 16 15 14 13 12 11 10 9 8 7 6 5 4 3 2 1 0

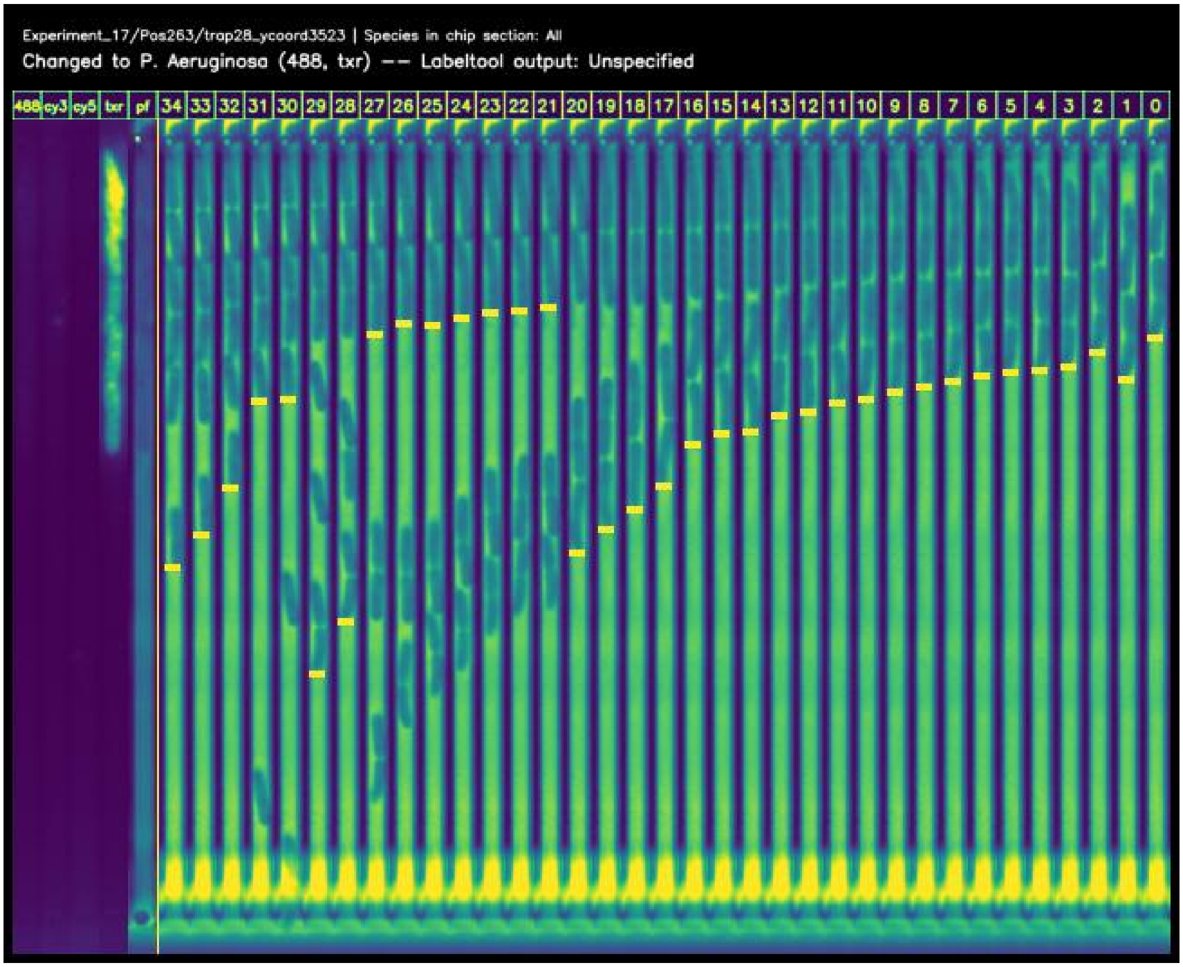
Experiment_17/Pos263/trap28_ycoord3523 | Species in chip section: All
Changed to P. Aeruginosa (488, txr) -- Labeltool output: Unspecified
488 cy3 cy5 txr pf 34 33 32 31 30 29 28 27 26 25 24 23 22 21 20 19 18 17 16 15 14 13 12 11 10 9 8 7 6 5 4 3 2 1 0

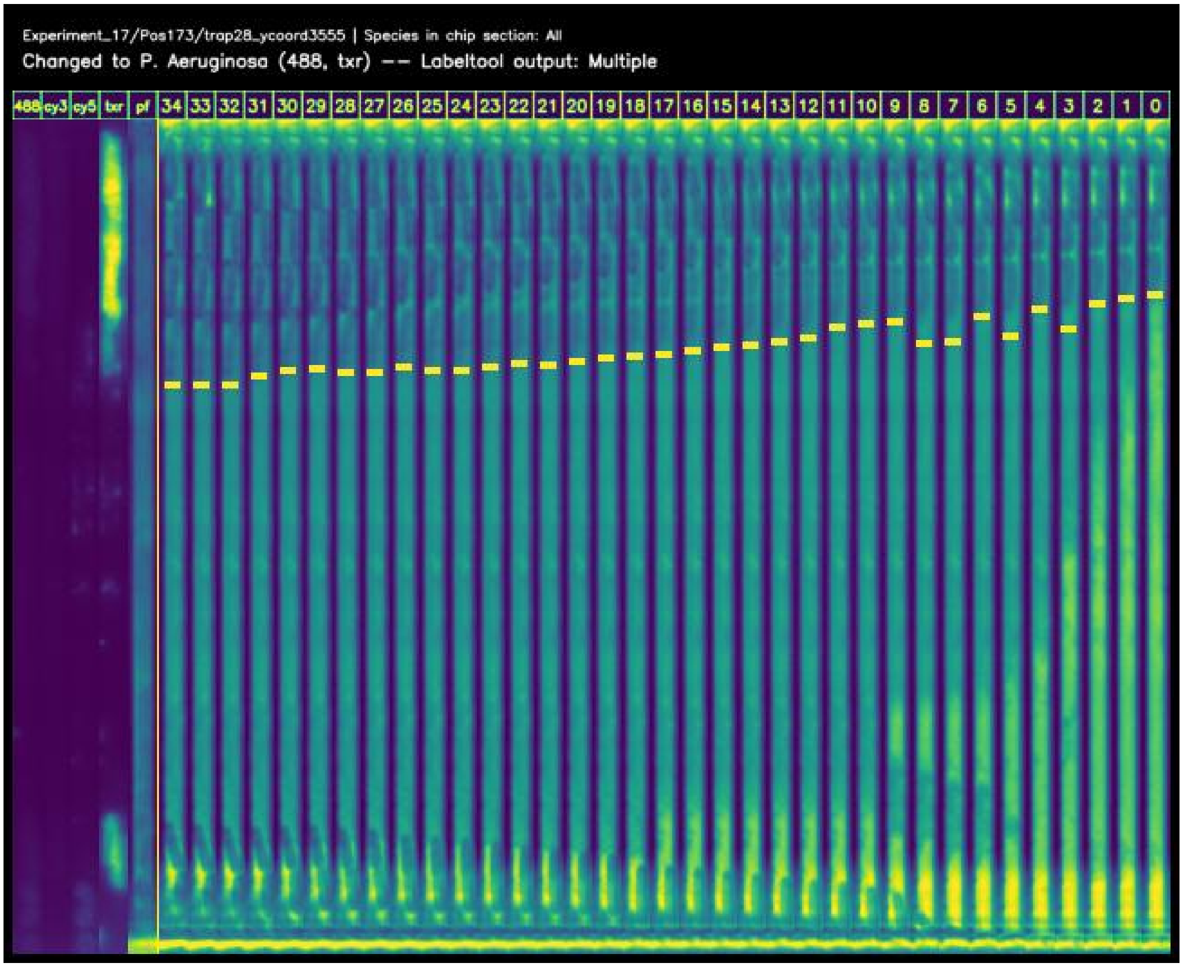
Experiment_17/Pos173/trap28_ycoord3555 | Species in chip section: All
Changed to P. Aeruginosa (488, txr) -- Labeltool output: Multiple
488 cy3 cy5 txr pf 34 33 32 31 30 29 28 27 26 25 24 23 22 21 20 19 18 17 16 15 14 13 12 11 10 9 8 7 6 5 4 3 2 1 0

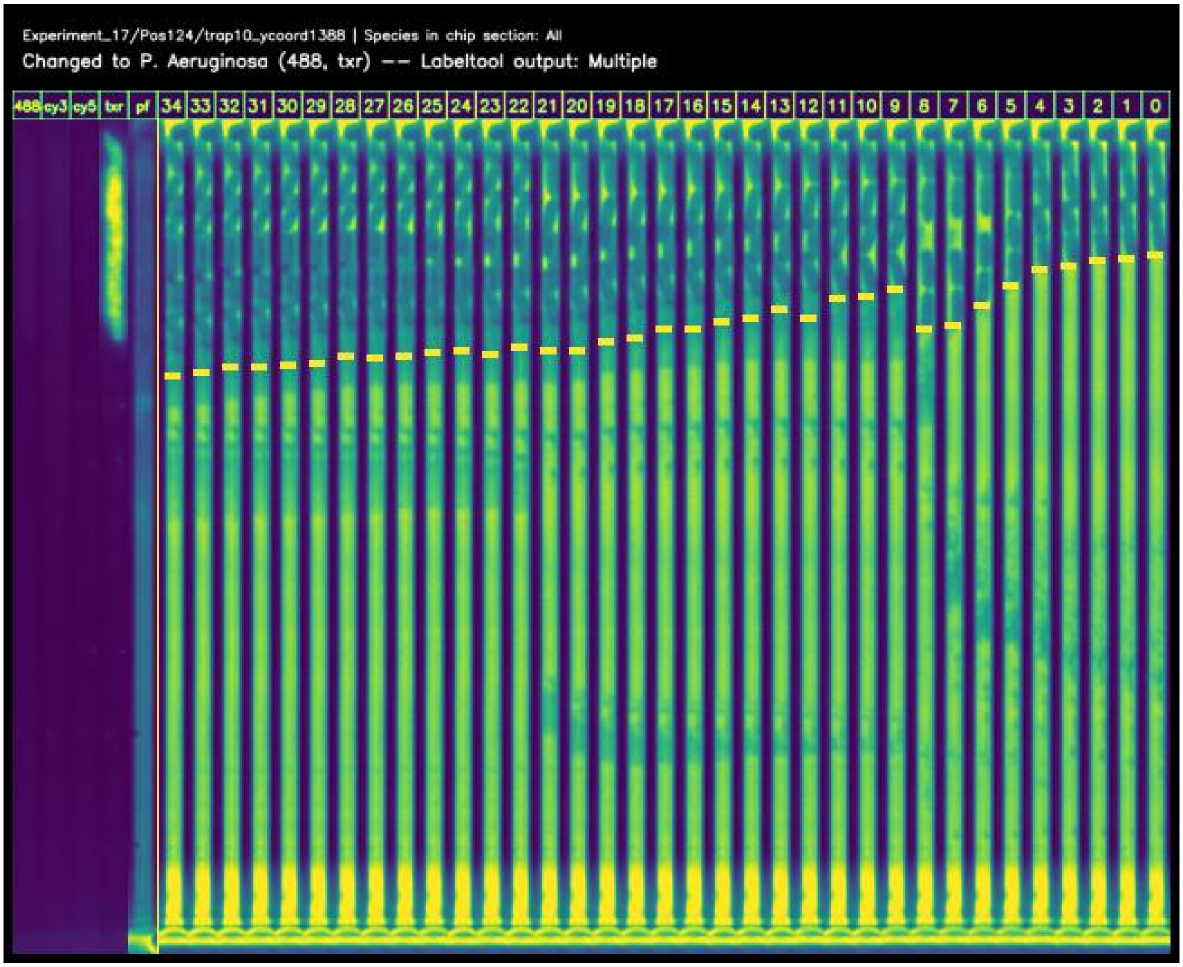
Experiment_17/Pos124/trap10_ycoord1388 | Species in chip section: All
Changed to P. Aeruginosa (488, txr) -- Labeltool output: Multiple
488 cy3 cy5 txr pf 34 33 32 31 30 29 28 27 26 25 24 23 22 21 20 19 18 17 16 15 14 13 12 11 10 9 8 7 6 5 4 3 2 1 0

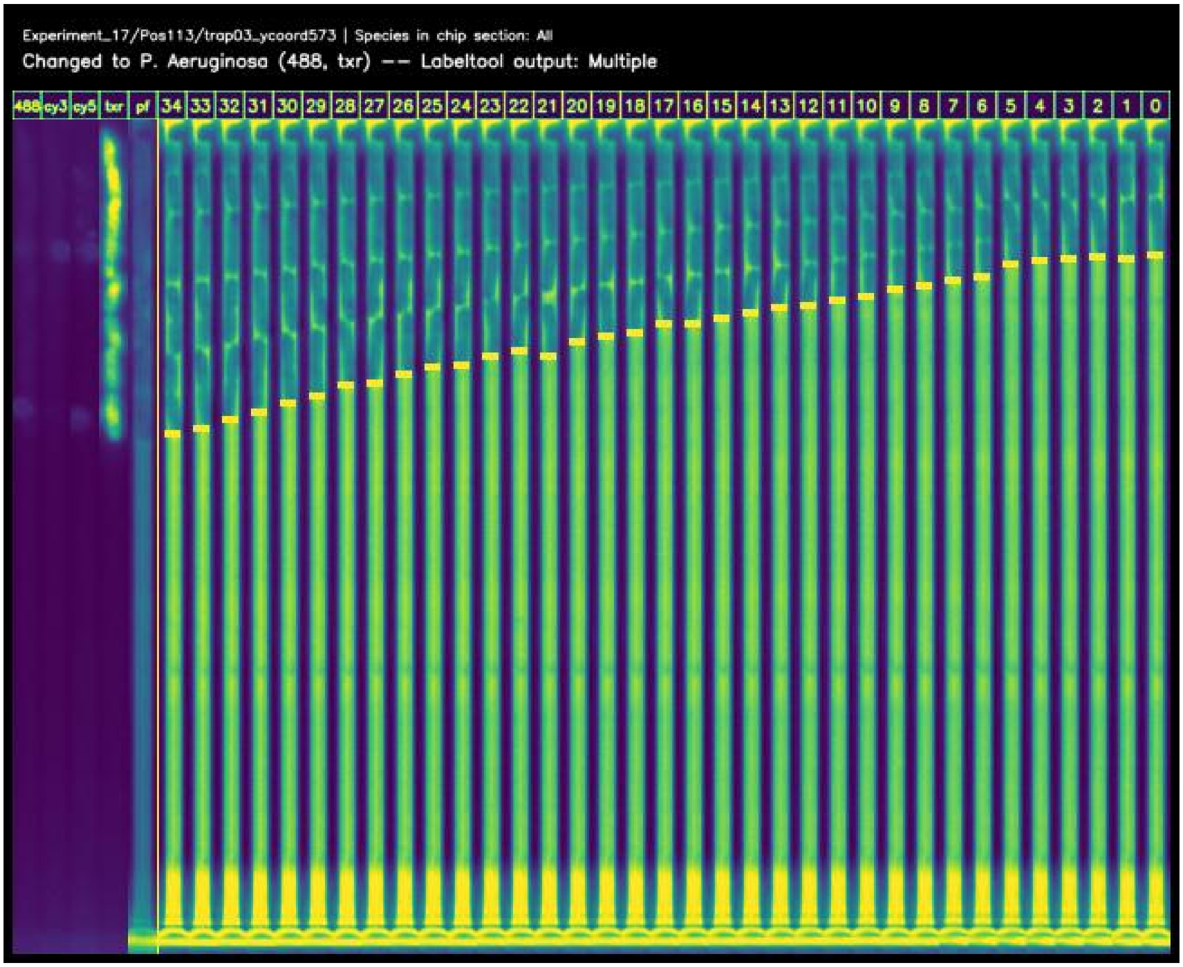
Experiment_17/Pos113/trap03_ycoord573 | Species in chip section: All
Changed to P. Aeruginosa (488, txr) -- Labeltool output: Multiple
488 cy3 cy5 txr pf 34 33 32 31 30 29 28 27 26 25 24 23 22 21 20 19 18 17 16 15 14 13 12 11 10 9 8 7 6 5 4 3 2 1 0

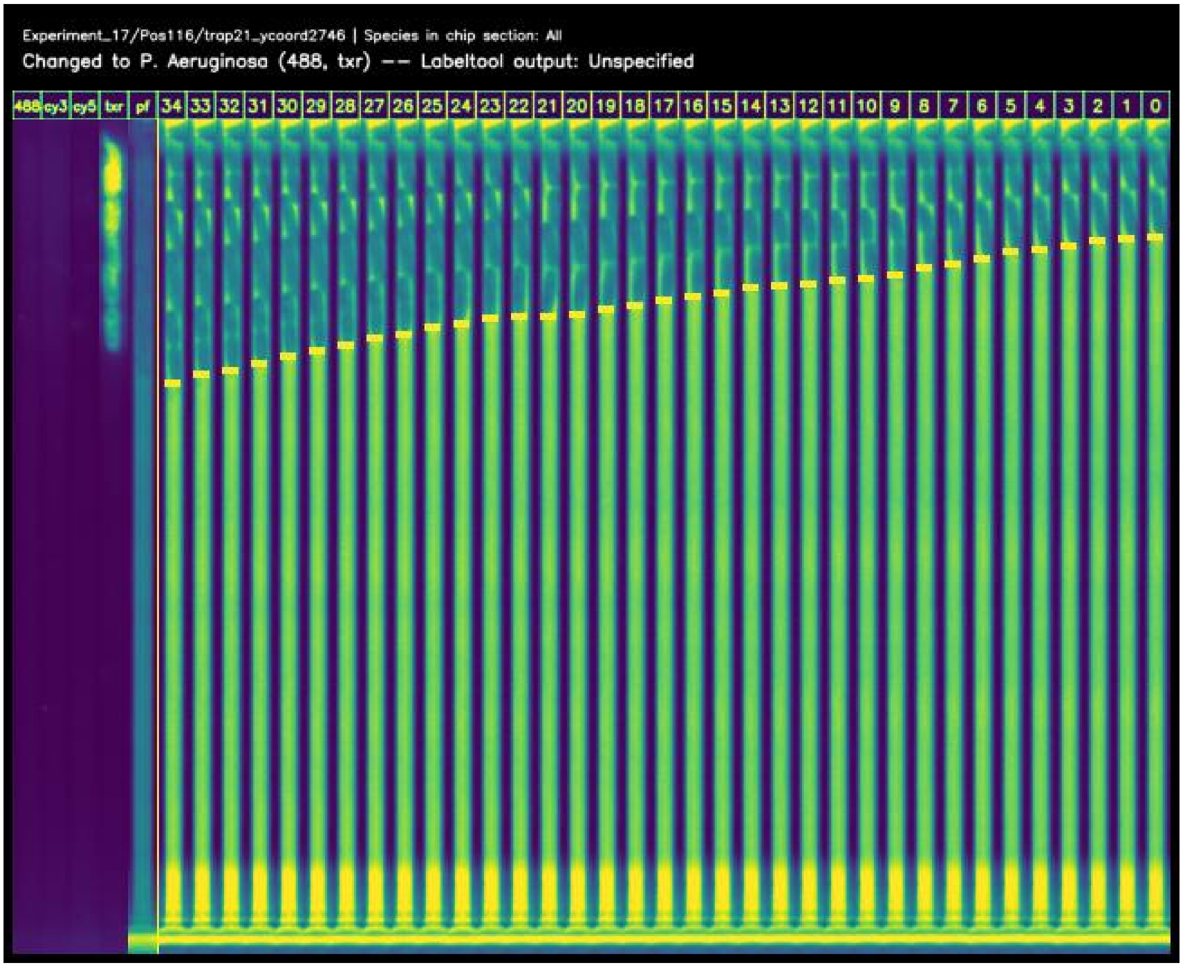
Experiment_17/Pos116/trap21_ycoord2746 | Species in chip section: All
Changed to P. Aeruginosa (488, txr) -- Labeltool output: Unspecified
488 cy3 cy5 txr pf 34 33 32 31 30 29 28 27 26 25 24 23 22 21 20 19 18 17 16 15 14 13 12 11 10 9 8 7 6 5 4 3 2 1 0

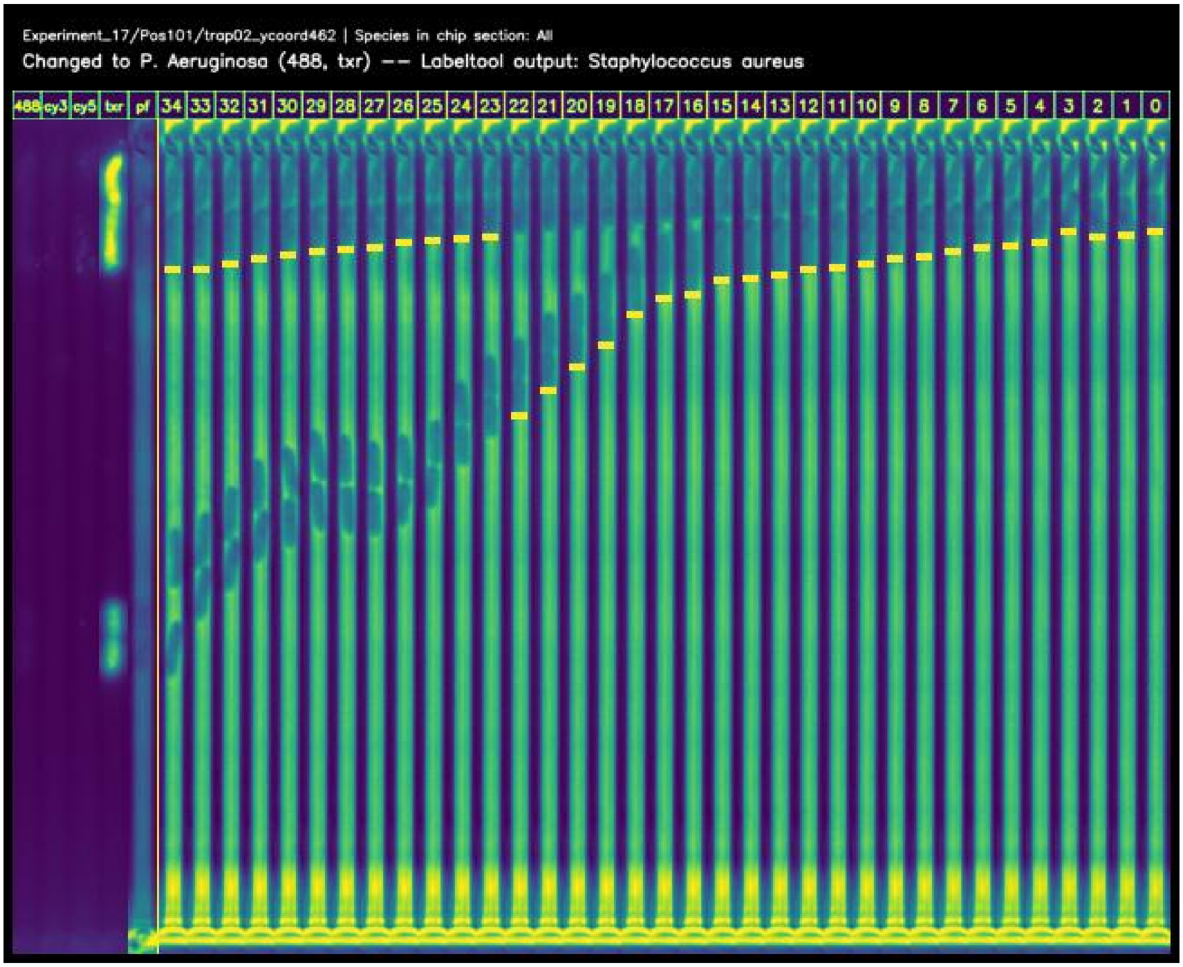
Experiment_17/Pos101/trap02_ycoord462 | Species in chip section: All
Changed to P. Aeruginosa (488, txr) -- Labeltool output: Staphylococcus aureus
488 cy3 cy5 txr pf 34 33 32 31 30 29 28 27 26 25 24 23 22 21 20 19 18 17 16 15 14 13 12 11 10 9 8 7 6 5 4 3 2 1 0

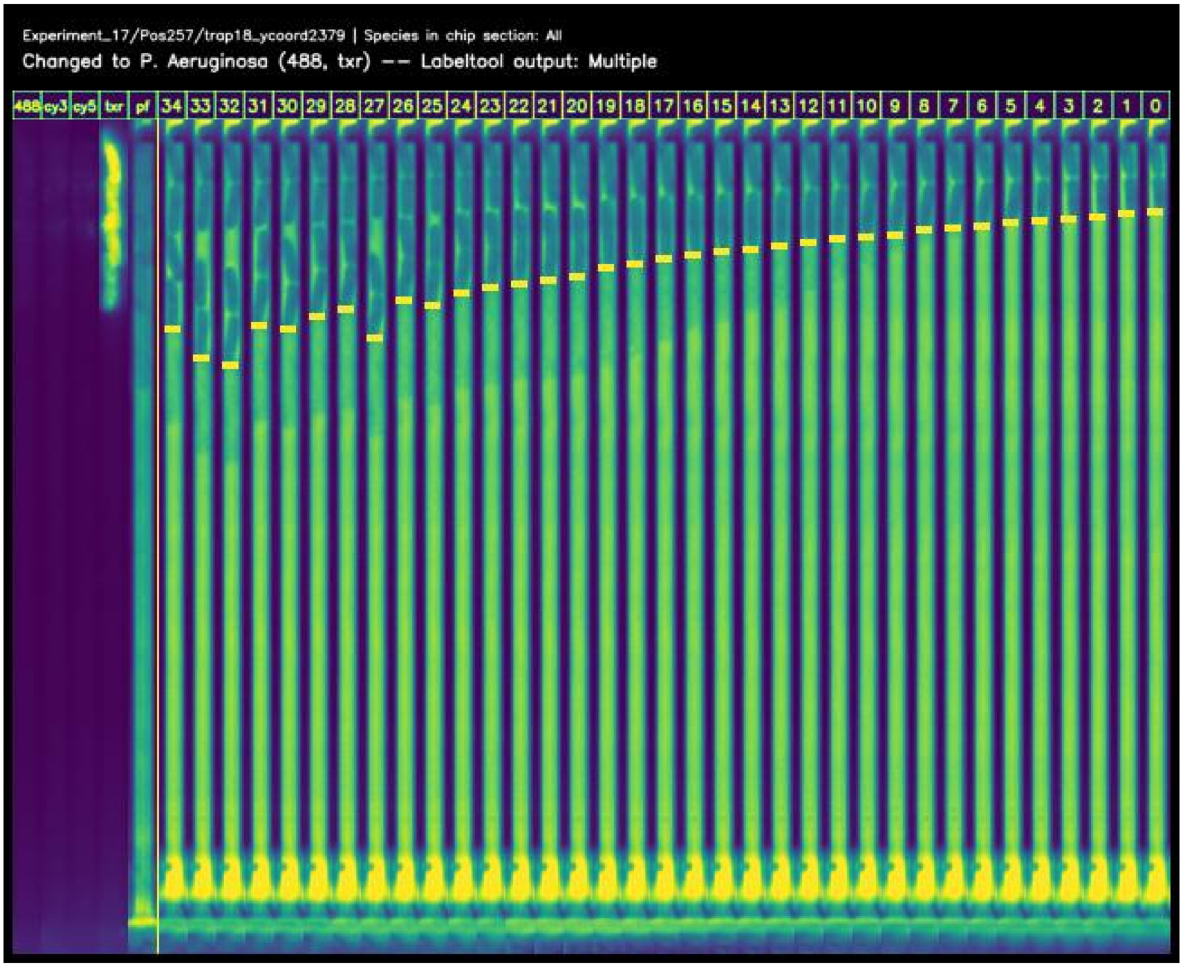
Experiment_17/Pos257/trap18_ycoord2379 | Species in chip section: All
Changed to P. Aeruginosa (488, txr) -- Labeltool output: Multiple
488 cy3 cy5 txr pf 34 33 32 31 30 29 28 27 26 25 24 23 22 21 20 19 18 17 16 15 14 13 12 11 10 9 8 7 6 5 4 3 2 1 0

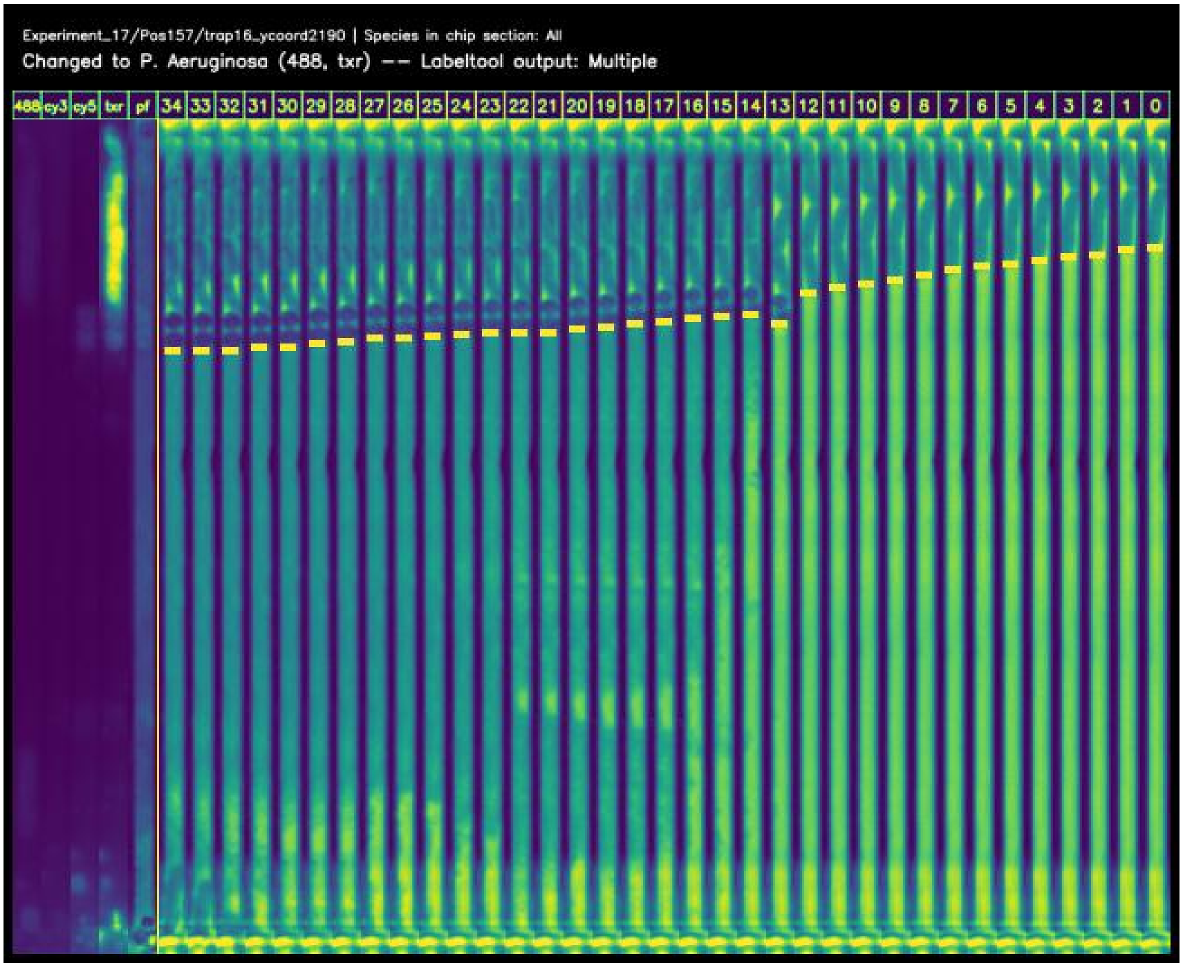

Experiment_17/Pos157/trap16_ycoord2190 | Species in chip section: All
Changed to P. Aeruginosa (488, txr) -- Labeltool output: Multiple
488 cy3 cy5 txr pf 34 33 32 31 30 29 28 27 26 25 24 23 22 21 20 19 18 17 16 15 14 13 12 11 10 9 8 7 6 5 4 3 2 1 0

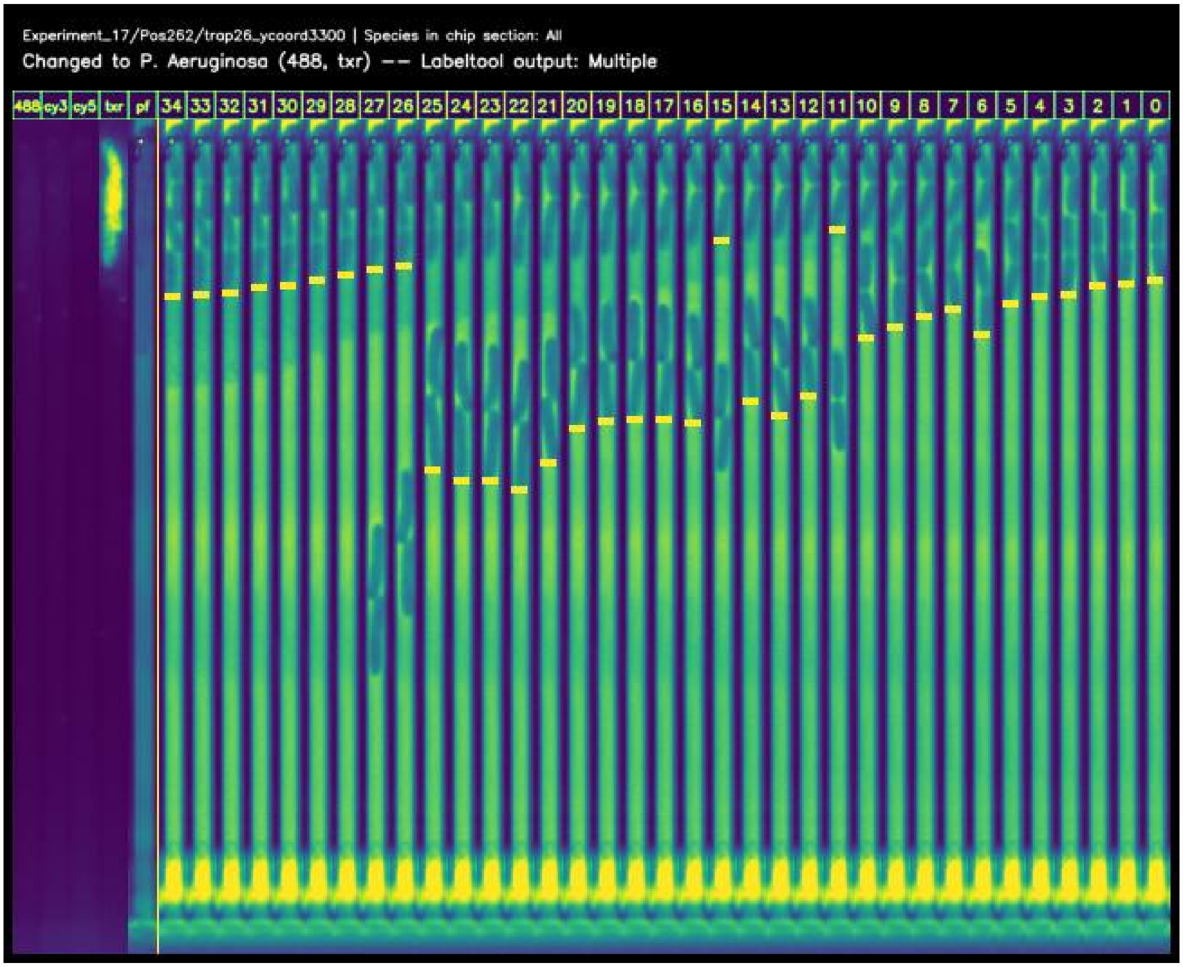

Experiment_17/Pos262/trap26_ycoord3300 | Species in chip section: All
Changed to P. Aeruginosa (488, txr) -- Labeltool output: Multiple
488 cy3 cy5 txr pf 34 33 32 31 30 29 28 27 26 25 24 23 22 21 20 19 18 17 16 15 14 13 12 11 10 9 8 7 6 5 4 3 2 1 0

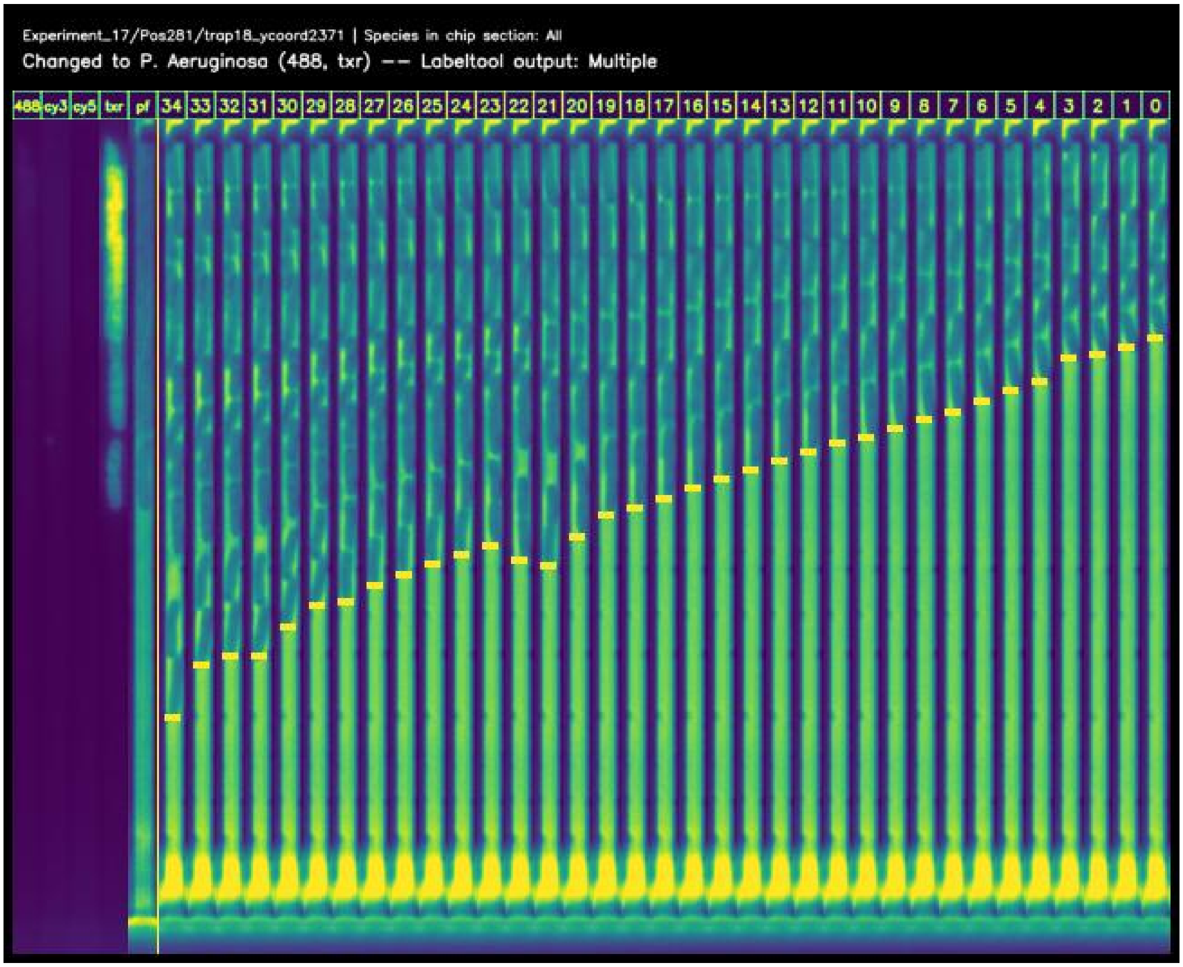
Experiment_17/Pos281/trap18_ycoord2371 | Species in chip section: All
Changed to P. Aeruginosa (488, txr) -- Labeltool output: Multiple
488 cy3 cy5 txr pf 34 33 32 31 30 29 28 27 26 25 24 23 22 21 20 19 18 17 16 15 14 13 12 11 10 9 8 7 6 5 4 3 2 1 0

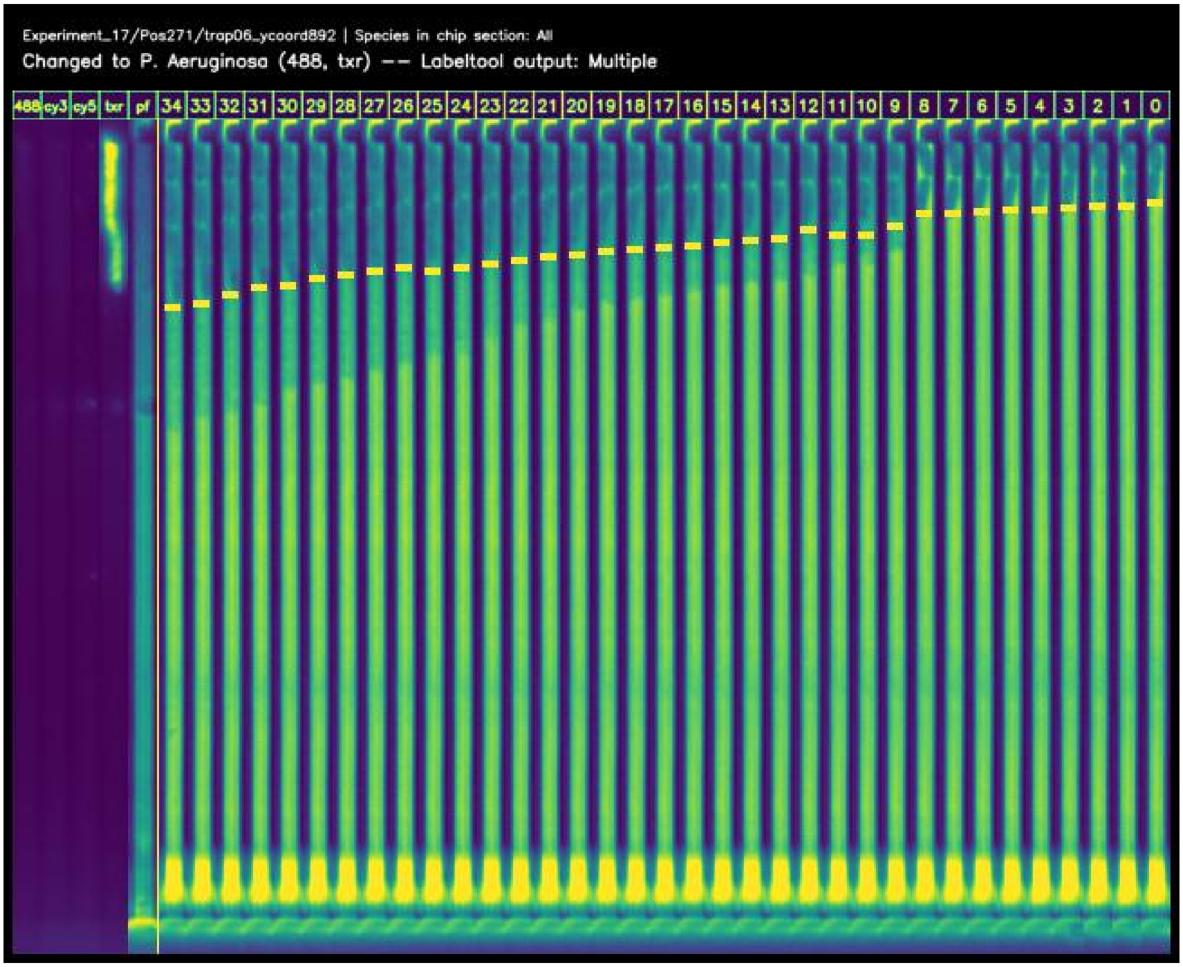
Experiment_17/Pos271/trap06_ycoord892 | Species in chip section: All
Changed to P. Aeruginosa (488, txr) -- Labeltool output: Multiple
488 cy3 cy5 txr pf 34 33 32 31 30 29 28 27 26 25 24 23 22 21 20 19 18 17 16 15 14 13 12 11 10 9 8 7 6 5 4 3 2 1 0

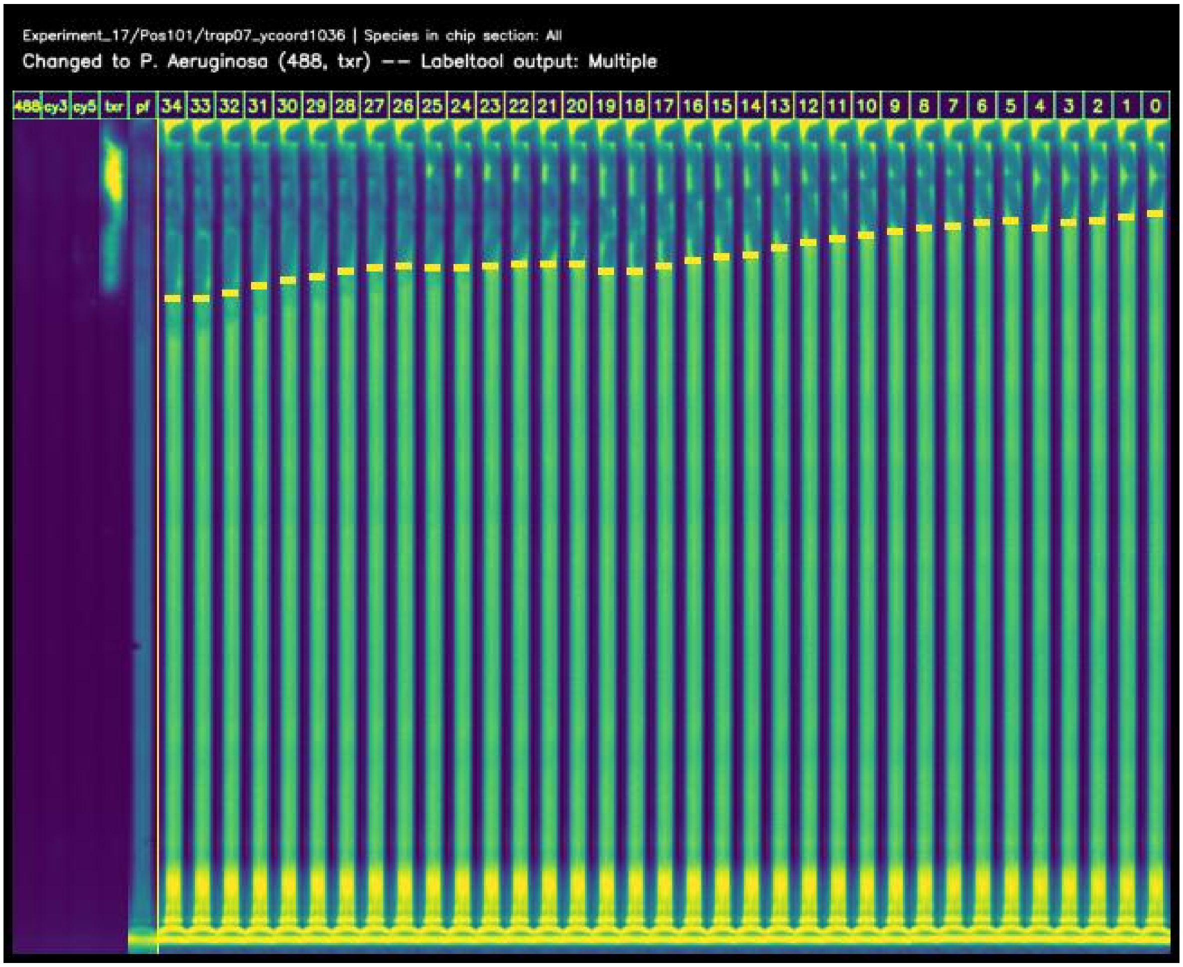
Experiment_17/Pos101/trap07_ycoord1036 | Species in chip section: All
Changed to P. Aeruginosa (488, txr) -- Labeltool output: Multiple
488 cy3 cy5 txr pf 34 33 32 31 30 29 28 27 26 25 24 23 22 21 20 19 18 17 16 15 14 13 12 11 10 9 8 7 6 5 4 3 2 1 0

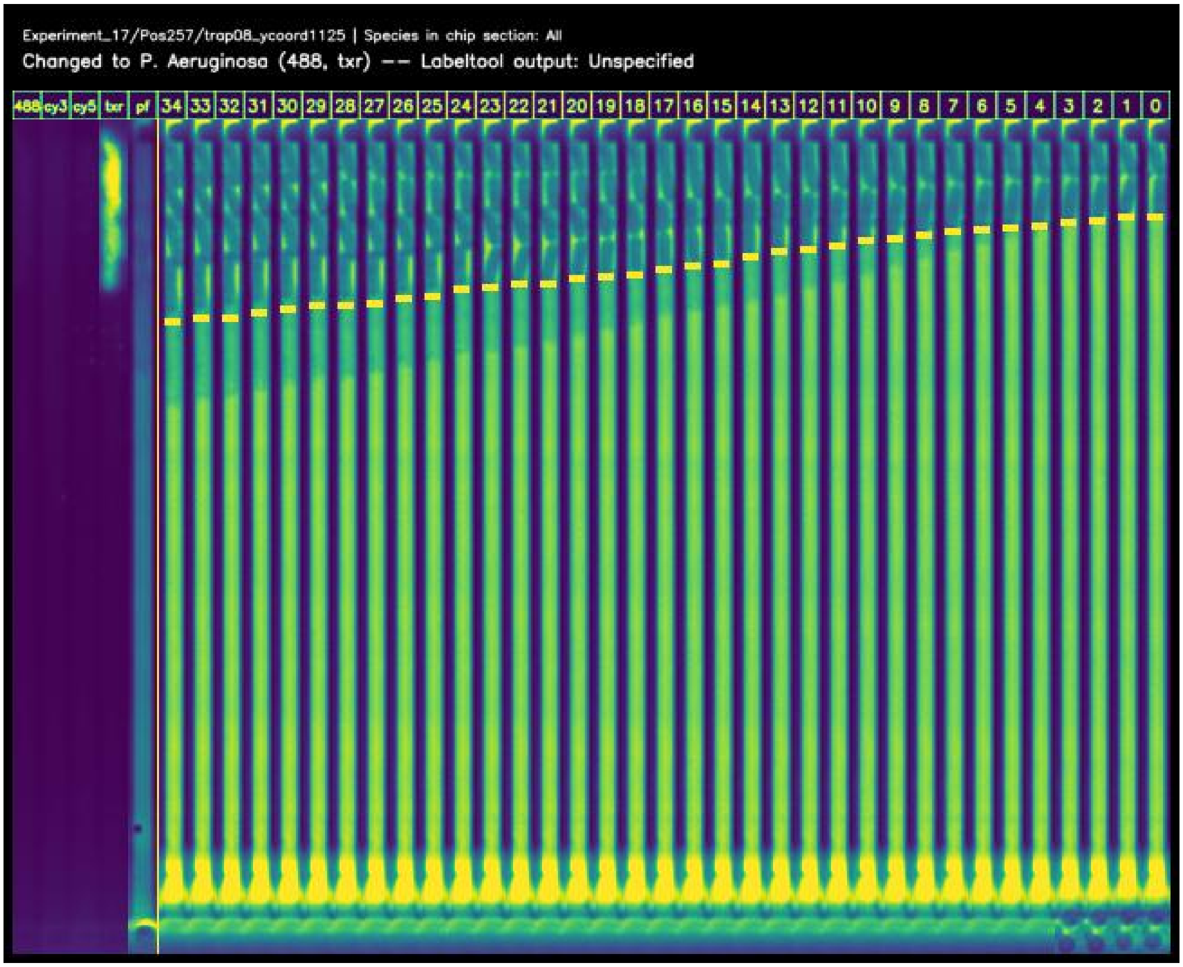
Experiment_17/Pos257/trap08_ycoord1125 | Species in chip section: All
Changed to P. Aeruginosa (488, txr) -- Labeltool output: Unspecified
488 cy3 cy5 txr pf 34 33 32 31 30 29 28 27 26 25 24 23 22 21 20 19 18 17 16 15 14 13 12 11 10 9 8 7 6 5 4 3 2 1 0

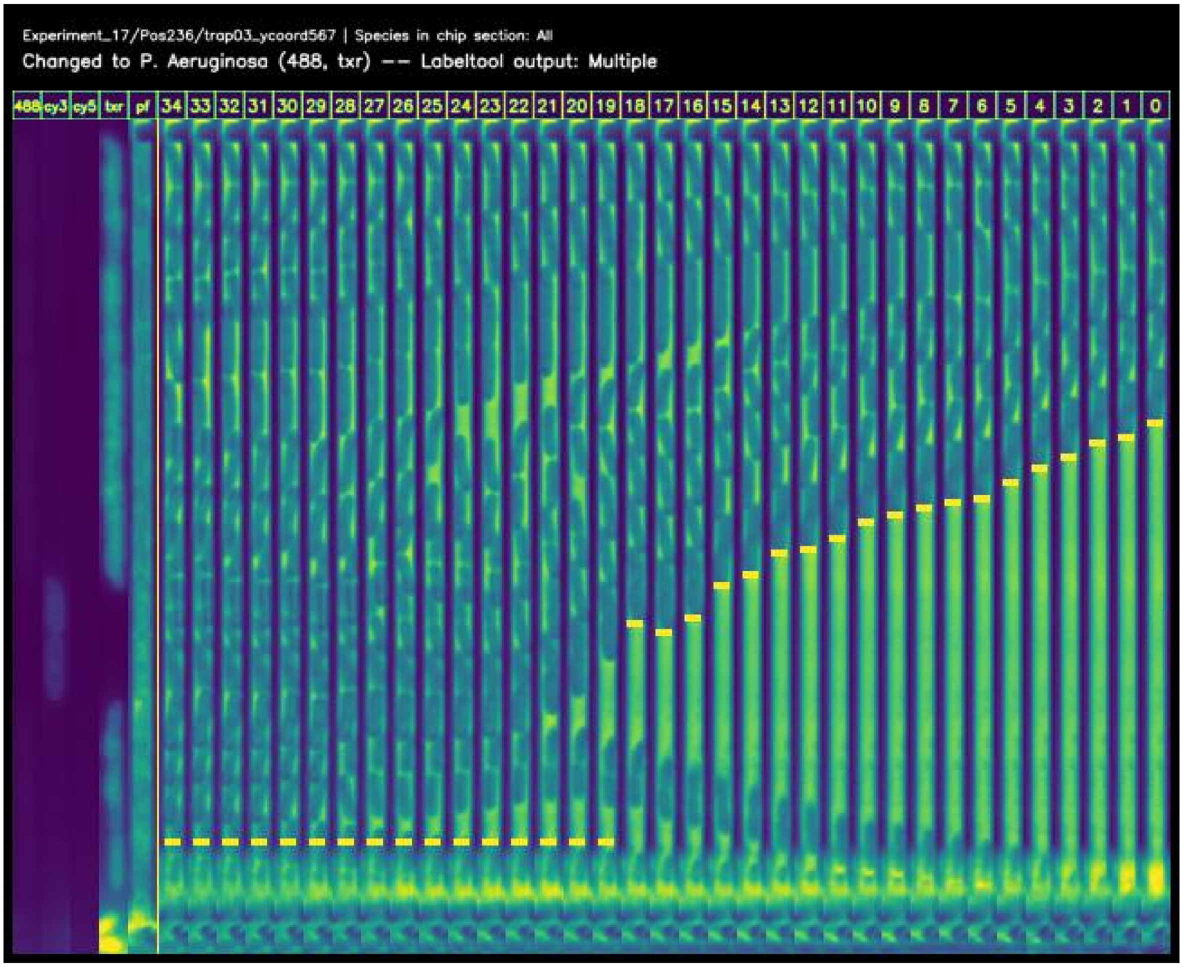
Experiment_17/Pos236/trap03_ycoord567 | Species in chip section: All
Changed to P. Aeruginosa (488, txr) -- Labeltool output: Multiple
488 cy3 cy5 txr pf 34 33 32 31 30 29 28 27 26 25 24 23 22 21 20 19 18 17 16 15 14 13 12 11 10 9 8 7 6 5 4 3 2 1 0

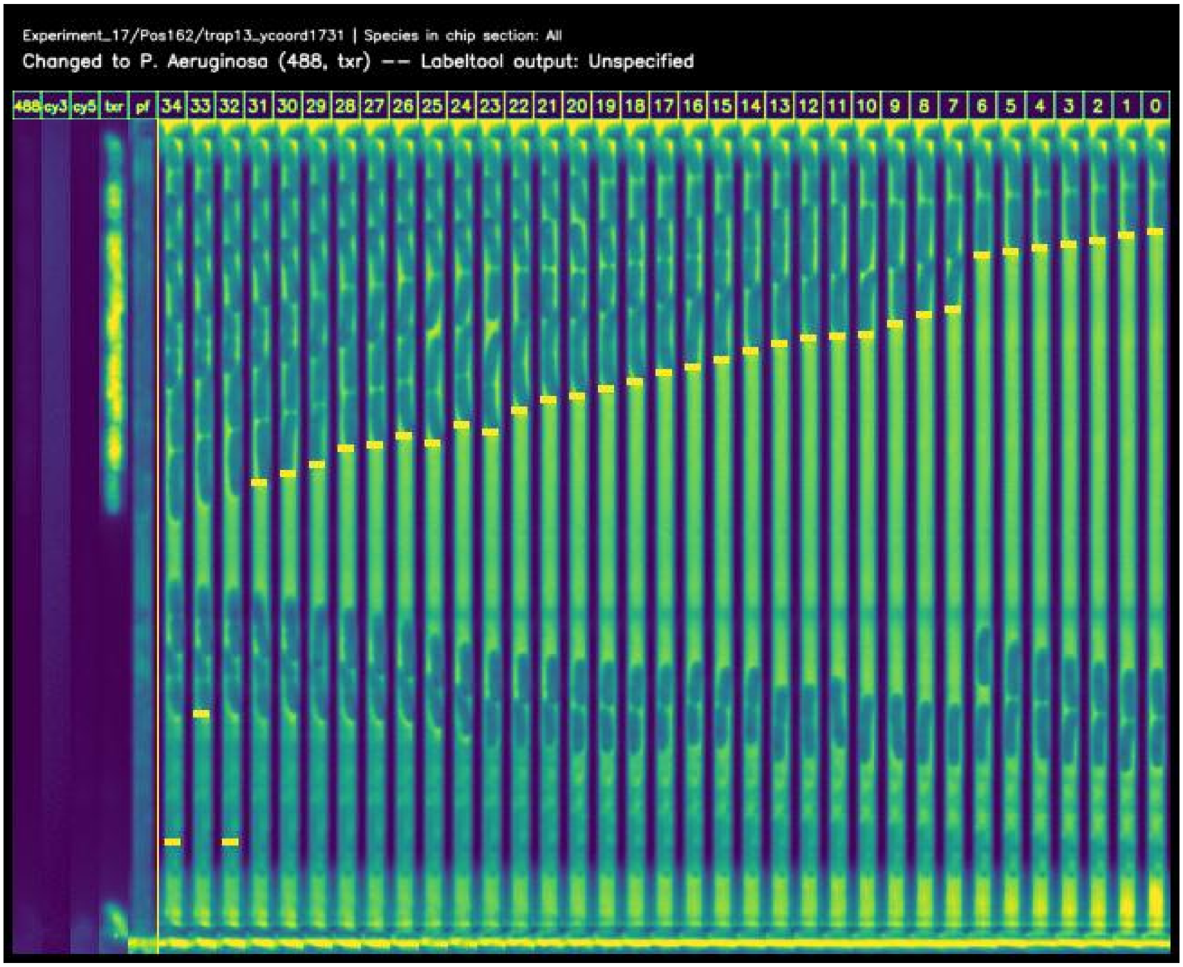
Experiment_17/Pos162/trap13_ycoord1731 | Species in chip section: All
Changed to P. Aeruginosa (488, txr) -- Labeltool output: Unspecified
488 cy3 cy5 txr pf 34 33 32 31 30 29 28 27 26 25 24 23 22 21 20 19 18 17 16 15 14 13 12 11 10 9 8 7 6 5 4 3 2 1 0

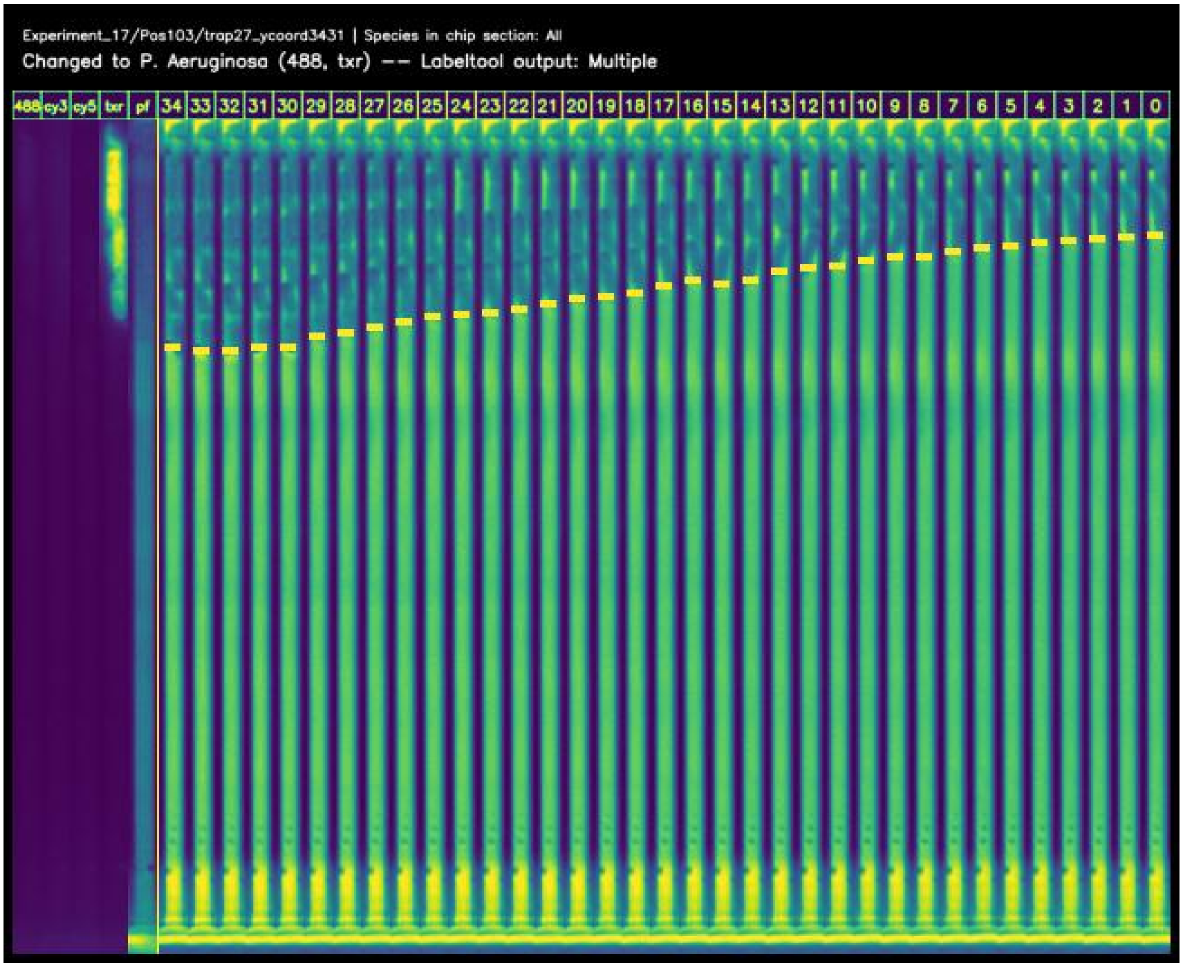
Experiment_17/Pos103/trap27_ycoord3431 | Species in chip section: All
Changed to P. Aeruginosa (488, txr) -- Labeltool output: Multiple
488 cy3 cy5 txr pf 34 33 32 31 30 29 28 27 26 25 24 23 22 21 20 19 18 17 16 15 14 13 12 11 10 9 8 7 6 5 4 3 2 1 0

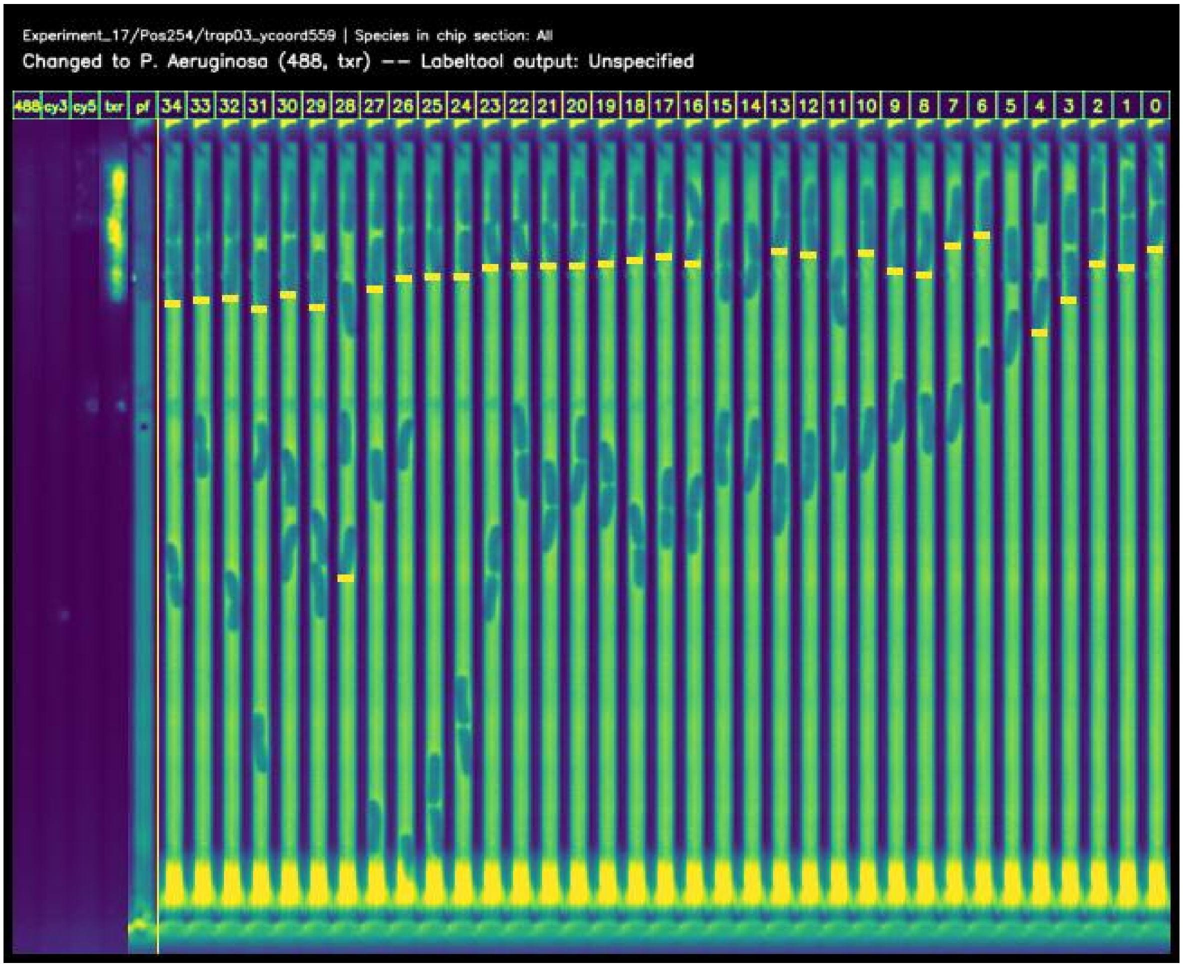
Experiment_17/Pos254/trap03_ycoord559 | Species in chip section: All
Changed to P. Aeruginosa (488, txr) -- Labeltool output: Unspecified
488 cy3 cy5 txr pf 34 33 32 31 30 29 28 27 26 25 24 23 22 21 20 19 18 17 16 15 14 13 12 11 10 9 8 7 6 5 4 3 2 1 0

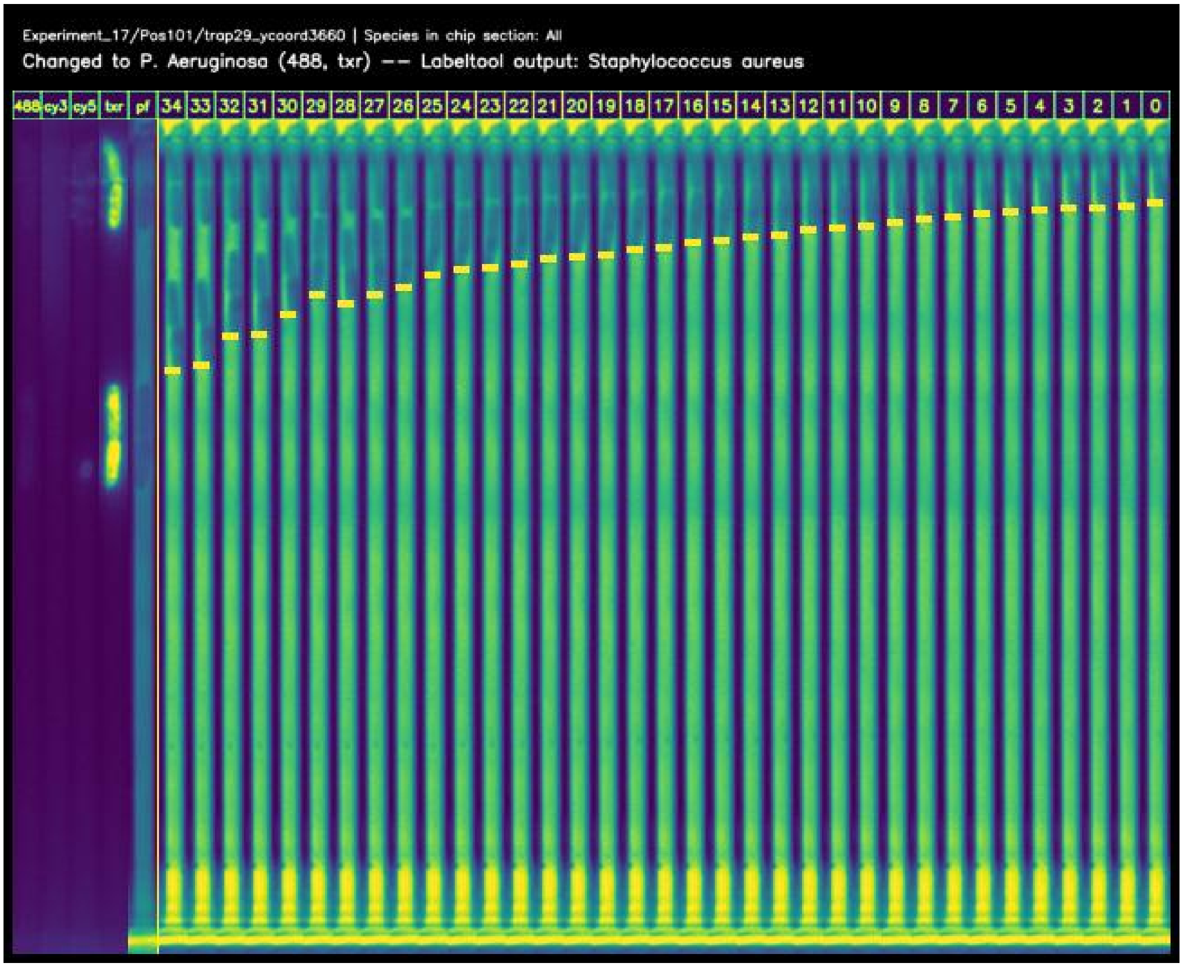
Experiment_17/Pos101/trap29_ycoord3660 | Species in chip section: All
Changed to P. Aeruginosa (488, txr) -- Labeltool output: Staphylococcus aureus
488 cy3 cy5 txr pf 34 33 32 31 30 29 28 27 26 25 24 23 22 21 20 19 18 17 16 15 14 13 12 11 10 9 8 7 6 5 4 3 2 1 0

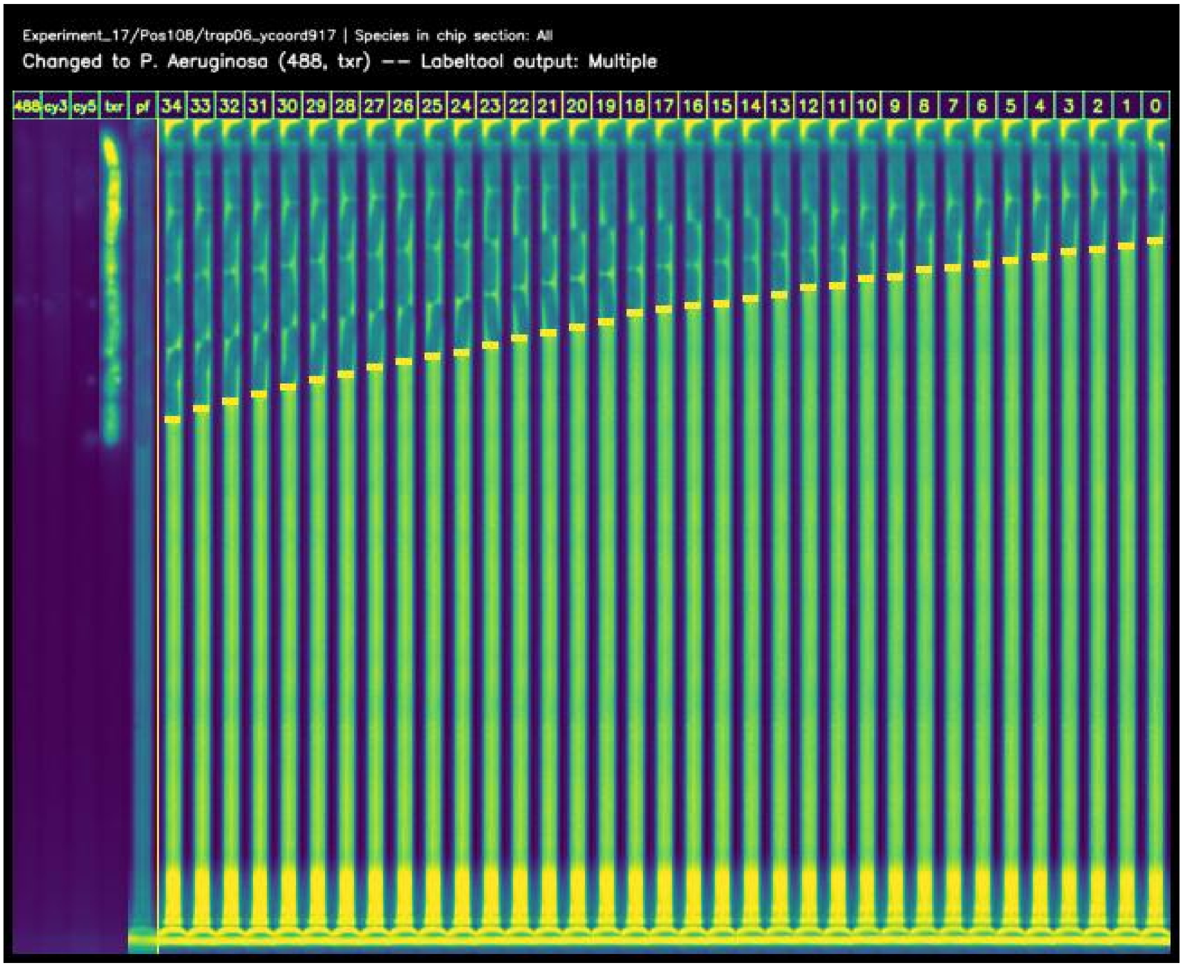
Experiment_17/Pos108/trap06_ycoord917 | Species in chip section: All
Changed to P. Aeruginosa (488, txr) -- Labeltool output: Multiple
488 cy3 cy5 txr pf 34 33 32 31 30 29 28 27 26 25 24 23 22 21 20 19 18 17 16 15 14 13 12 11 10 9 8 7 6 5 4 3 2 1 0

Discarded due to empty frames or dislocation

The following traps were discarded because they were either dislocated from the physical stop at the top of the trap or remained empty for 30 minutes (after frame 15).

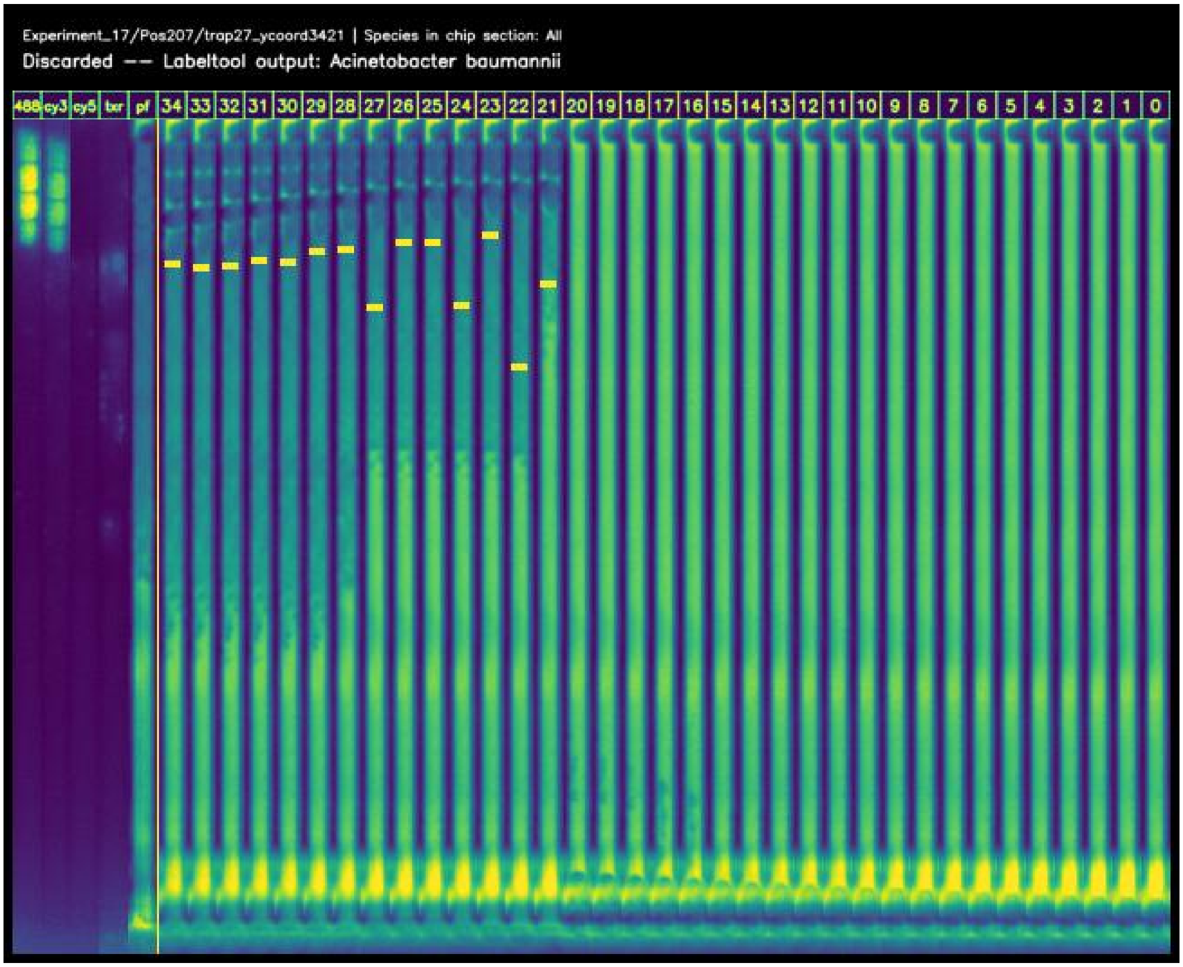

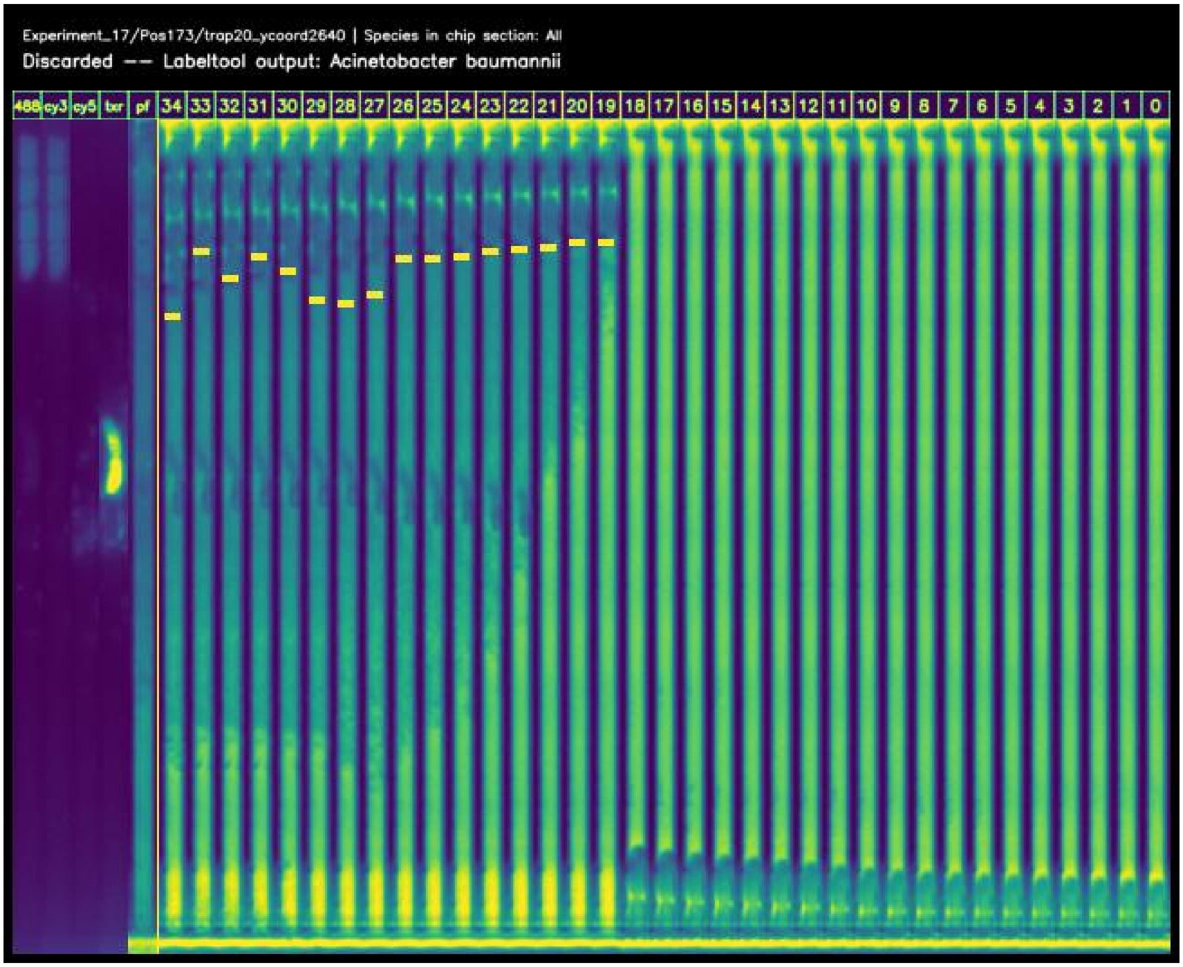
Experiment_17/Pos173/trap20_ycoord2640 | Species in chip section: All
Discarded -- Labeltool output: Acinetobacter baumannii
488 cy3 cy5 txr pf 34 33 32 31 30 29 28 27 26 25 24 23 22 21 20 19 18 17 16 15 14 13 12 11 10 9 8 7 6 5 4 3 2 1 0

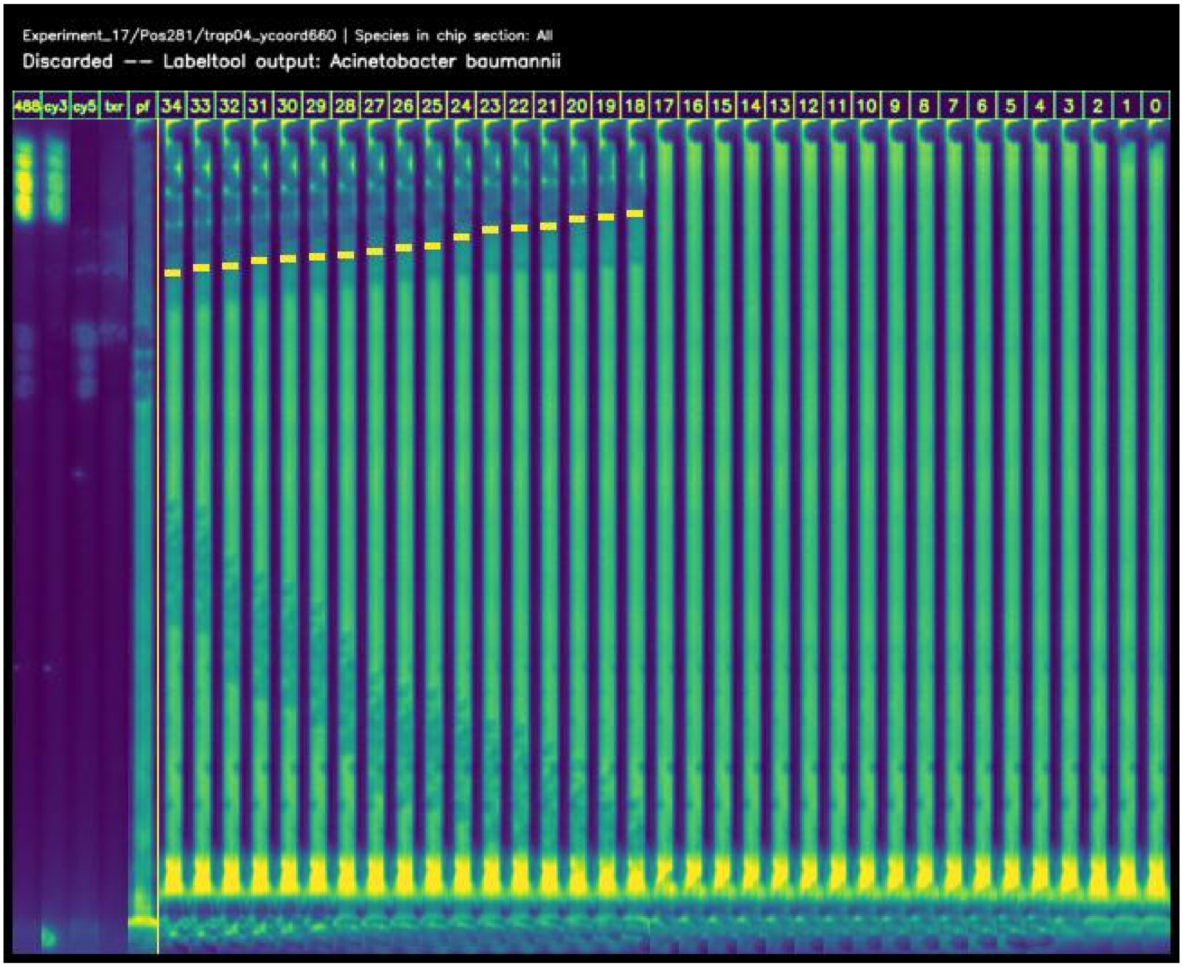
Experiment_17/Pos281/trap04_ycoord660 | Species in chip section: All
Discarded -- Labeltool output: Acinetobacter baumannii
488 cy3 cy5 txr pf 34 33 32 31 30 29 28 27 26 25 24 23 22 21 20 19 18 17 16 15 14 13 12 11 10 9 8 7 6 5 4 3 2 1 0

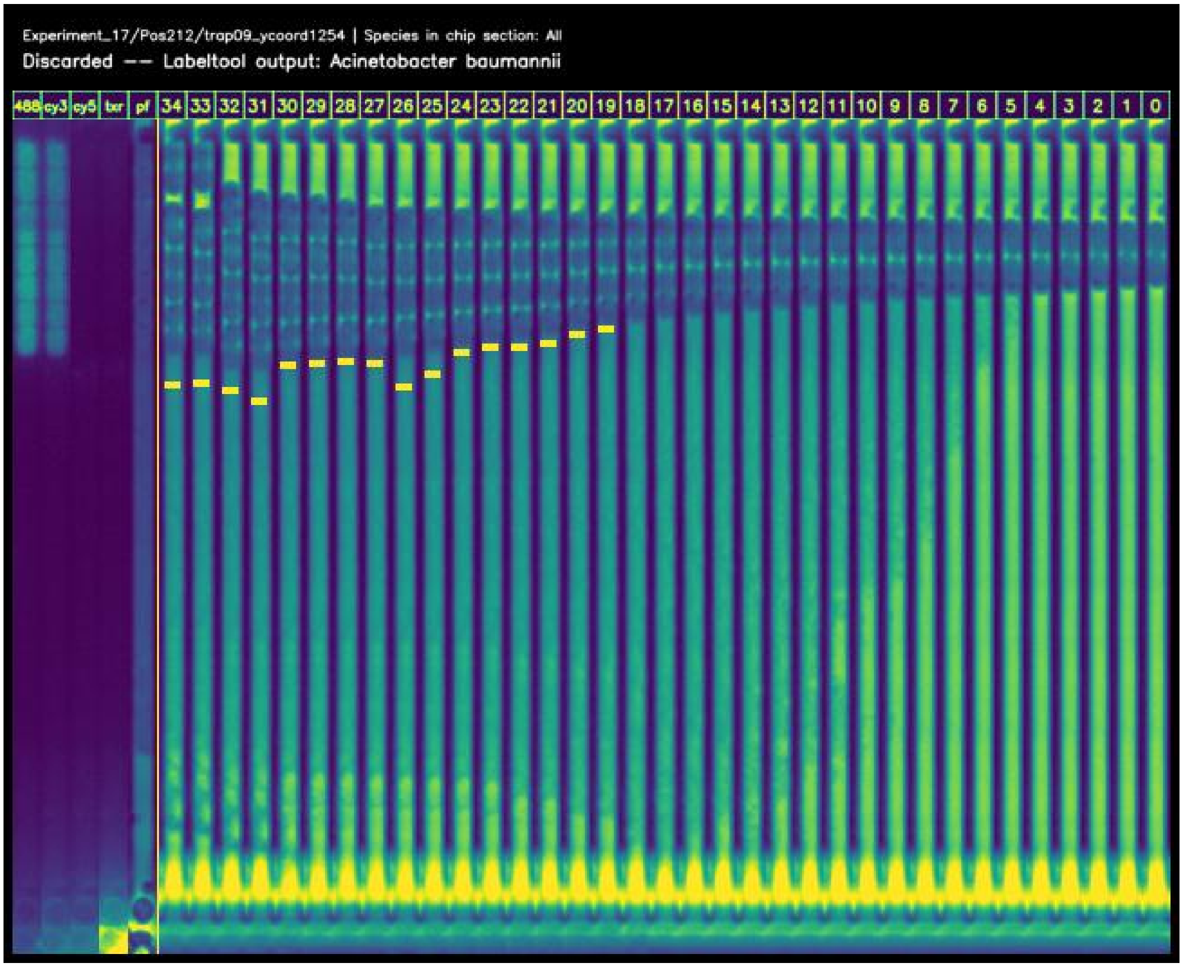
Experiment_17/Pos212/trap09_ycoord1254 | Species in chip section: All
Discarded -- Labeltool output: Acinetobacter baumannii
488 cy3 cy5 txr pf 34 33 32 31 30 29 28 27 26 25 24 23 22 21 20 19 18 17 16 15 14 13 12 11 10 9 8 7 6 5 4 3 2 1 0

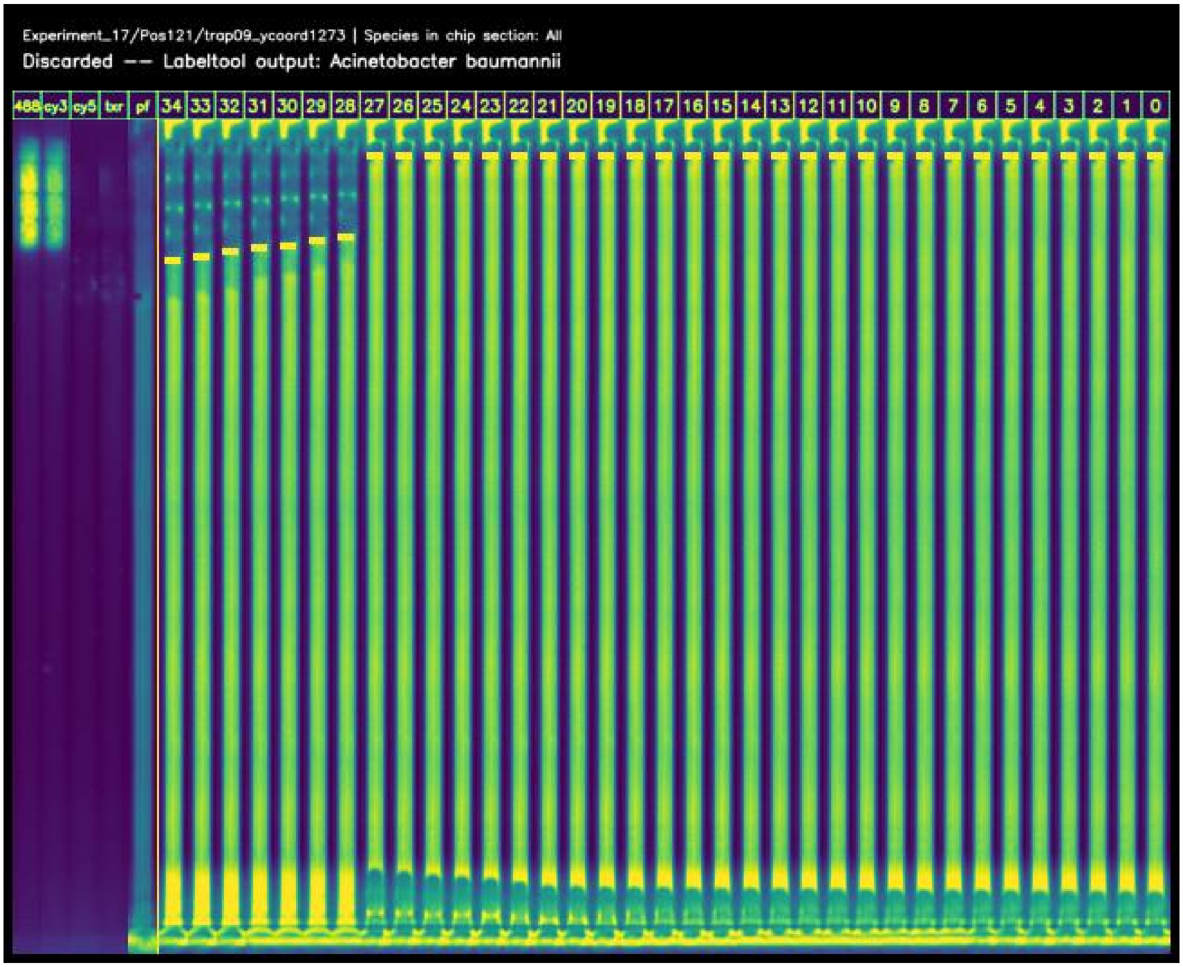
Experiment_17/Pos121/trap09_ycoord1273 | Species in chip section: All
Discarded -- Labeltool output: Acinetobacter baumannii
488 cy3 cy5 txr pf 34 33 32 31 30 29 28 27 26 25 24 23 22 21 20 19 18 17 16 15 14 13 12 11 10 9 8 7 6 5 4 3 2 1 0

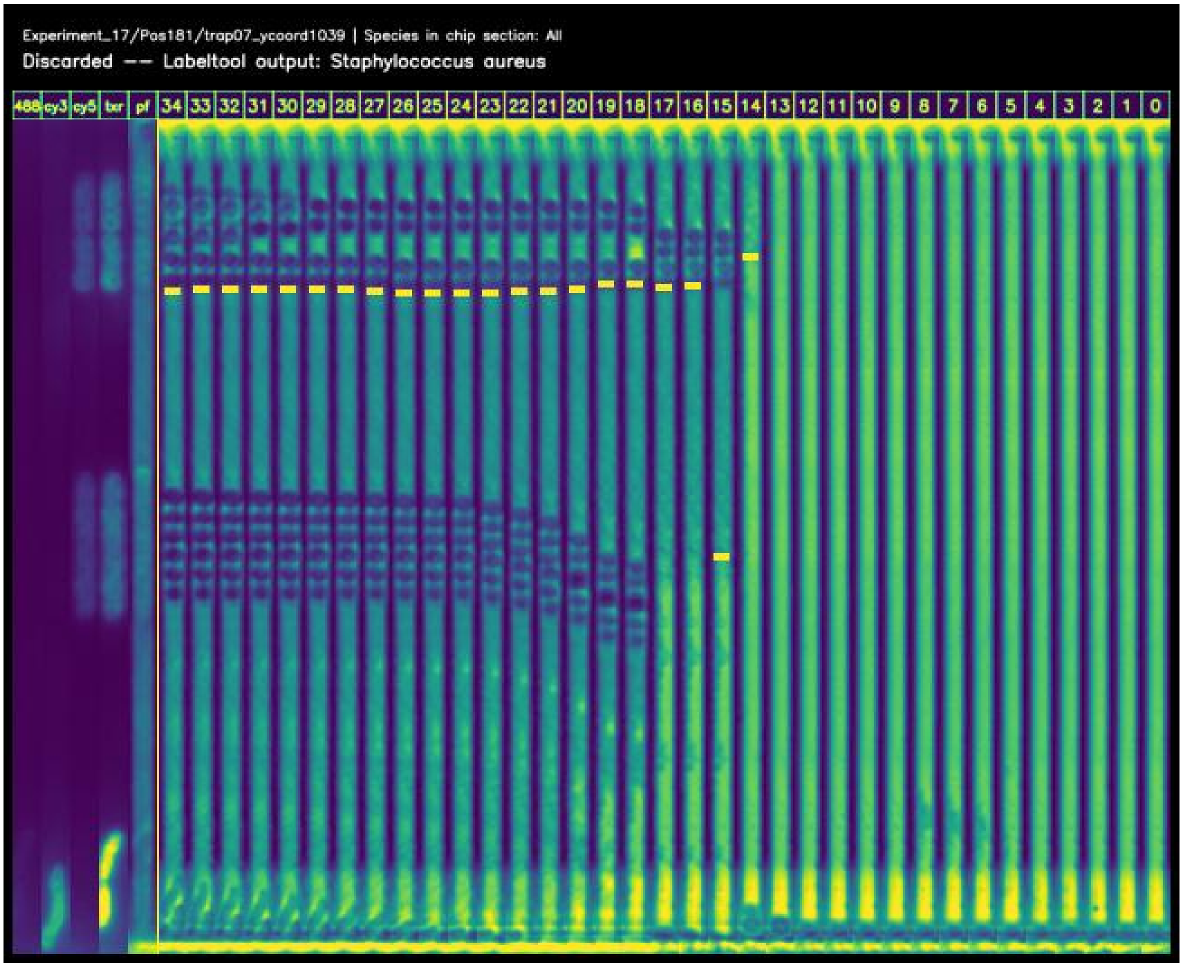
Experiment_17/Pos181/trap07_ycoord1039 | Species in chip section: All
Discarded -- Labeltool output: Staphylococcus aureus
488 cy3 cy5 txr pf 34 33 32 31 30 29 28 27 26 25 24 23 22 21 20 19 18 17 16 15 14 13 12 11 10 9 8 7 6 5 4 3 2 1 0

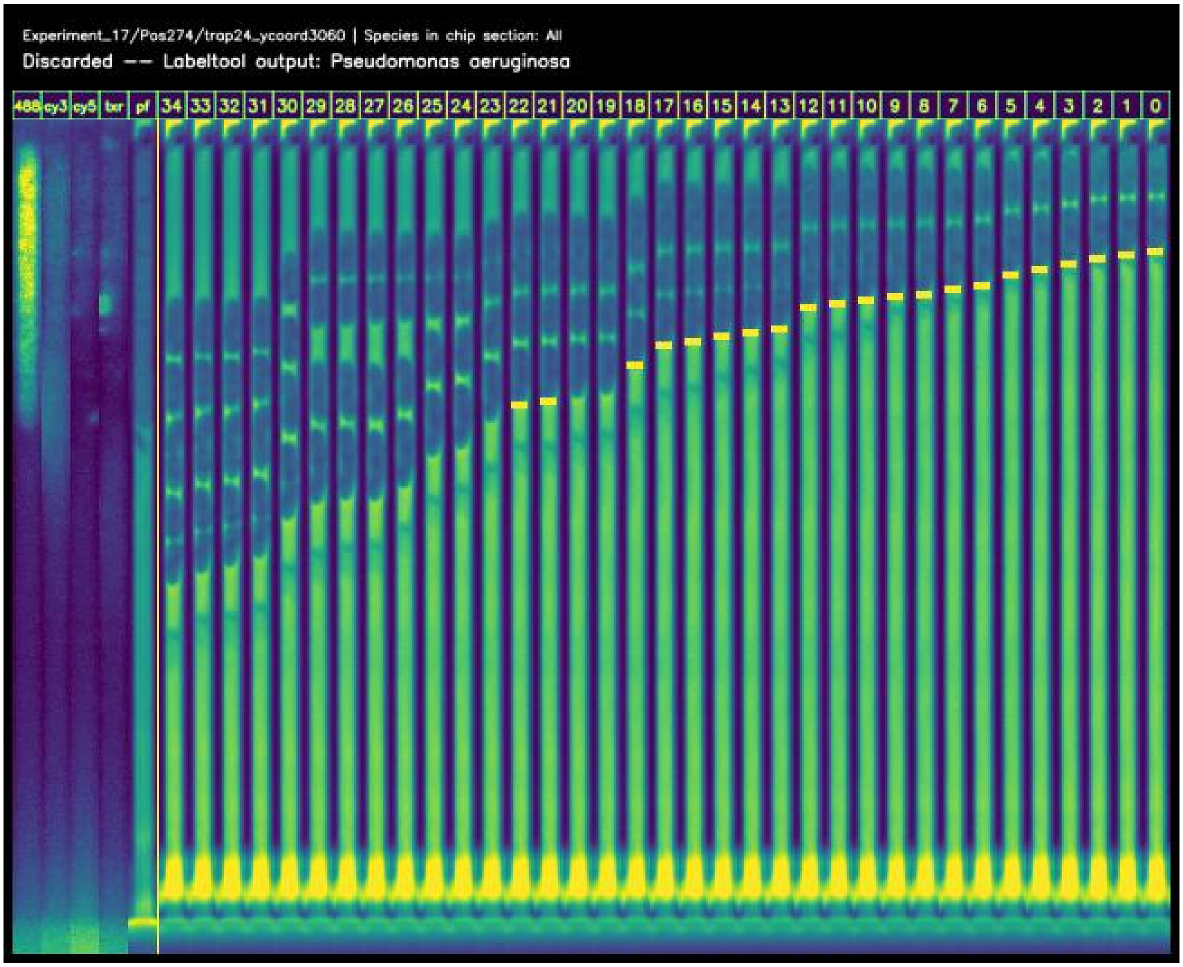
Experiment_17/Pos274/trap24_ycoord3060 | Species in chip section: All
Discarded -- Labeltool output: Pseudomonas aeruginosa
488 cy3 cy5 txr pf 34 33 32 31 30 29 28 27 26 25 24 23 22 21 20 19 18 17 16 15 14 13 12 11 10 9 8 7 6 5 4 3 2 1 0

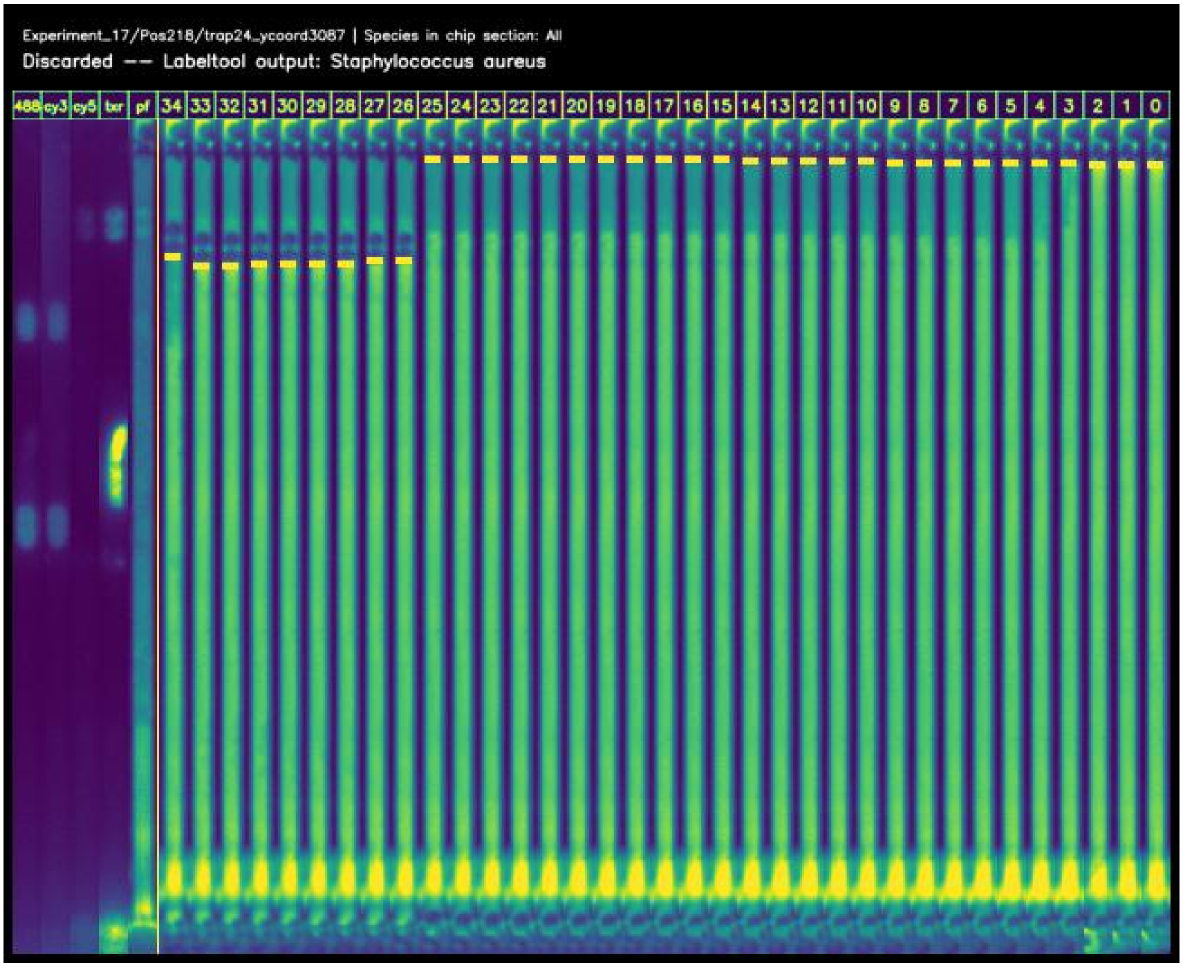
Experiment_17/Pos218/trap24_ycoord3087 | Species in chip section: All
Discarded -- Labeltool output: Staphylococcus aureus
488 cy3 cy5 txr pf 34 33 32 31 30 29 28 27 26 25 24 23 22 21 20 19 18 17 16 15 14 13 12 11 10 9 8 7 6 5 4 3 2 1 0

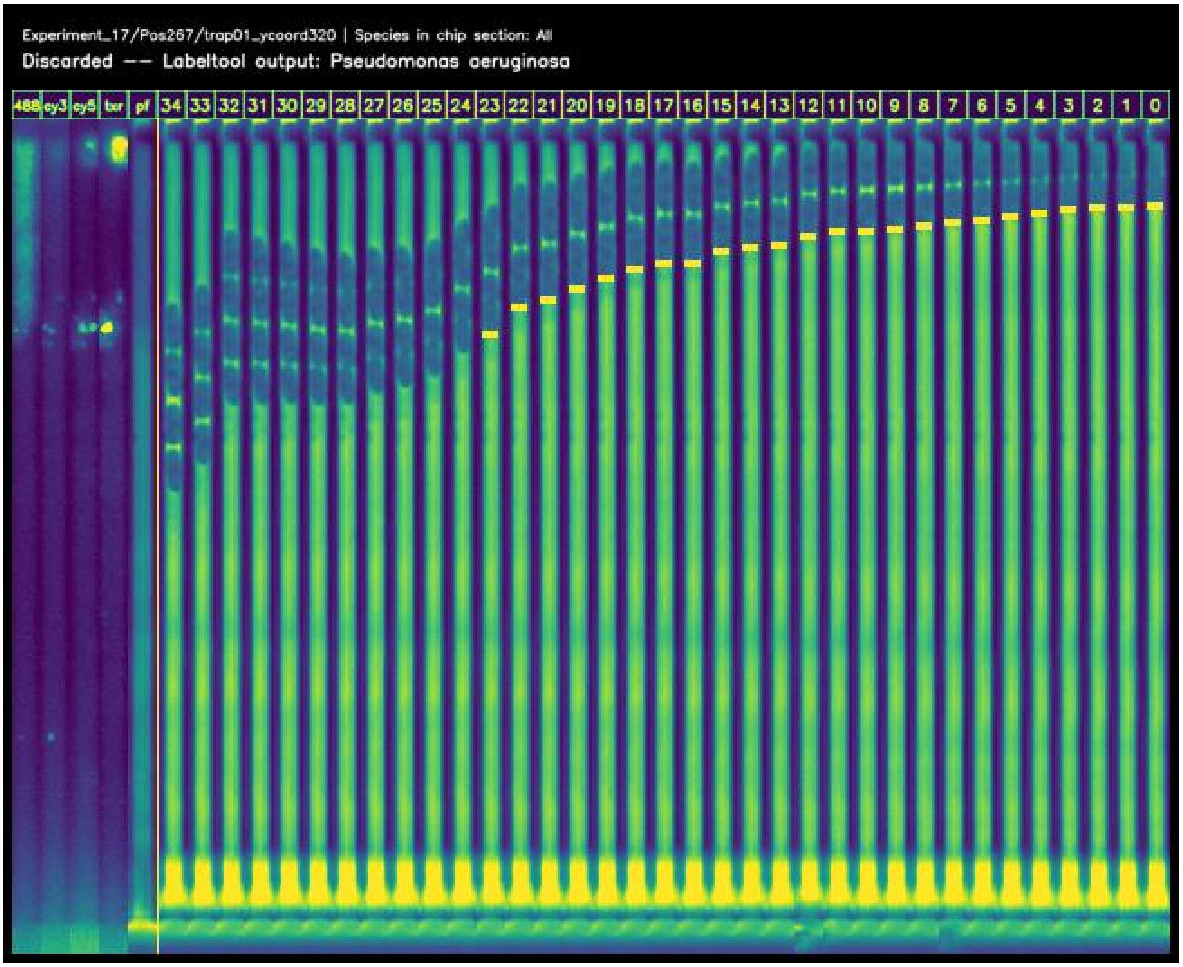
Experiment_17/Pos267/trap01_ycoord320 | Species in chip section: All
Discarded -- Labeltool output: Pseudomonas aeruginosa
488 cy3 cy5 txr pf 34 33 32 31 30 29 28 27 26 25 24 23 22 21 20 19 18 17 16 15 14 13 12 11 10 9 8 7 6 5 4 3 2 1 0

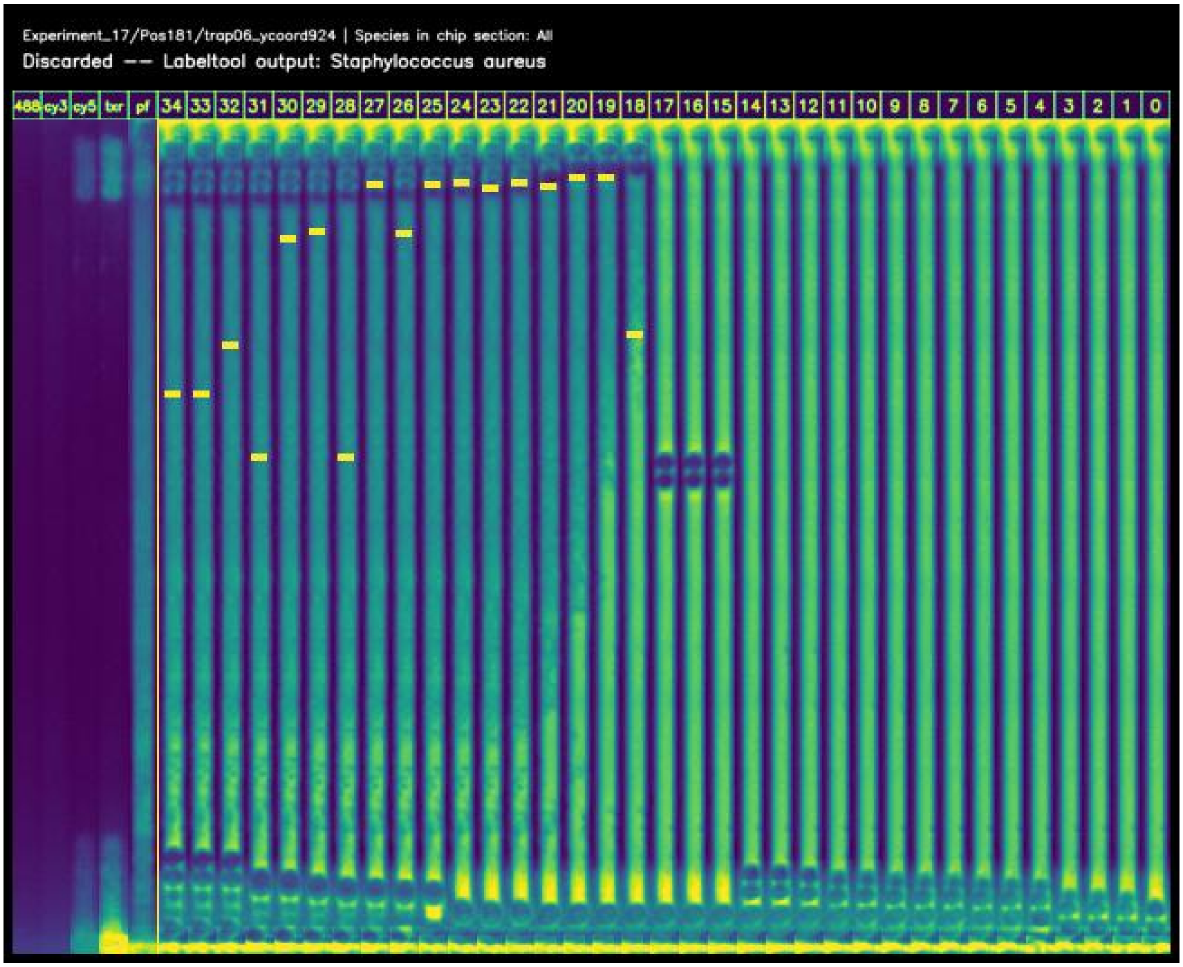
Experiment_17/Pos181/trap06_ycoord924 | Species in chip section: All
Discarded -- Labeltool output: Staphylococcus aureus
488 cy3 cy5 txr pf 34 33 32 31 30 29 28 27 26 25 24 23 22 21 20 19 18 17 16 15 14 13 12 11 10 9 8 7 6 5 4 3 2 1 0

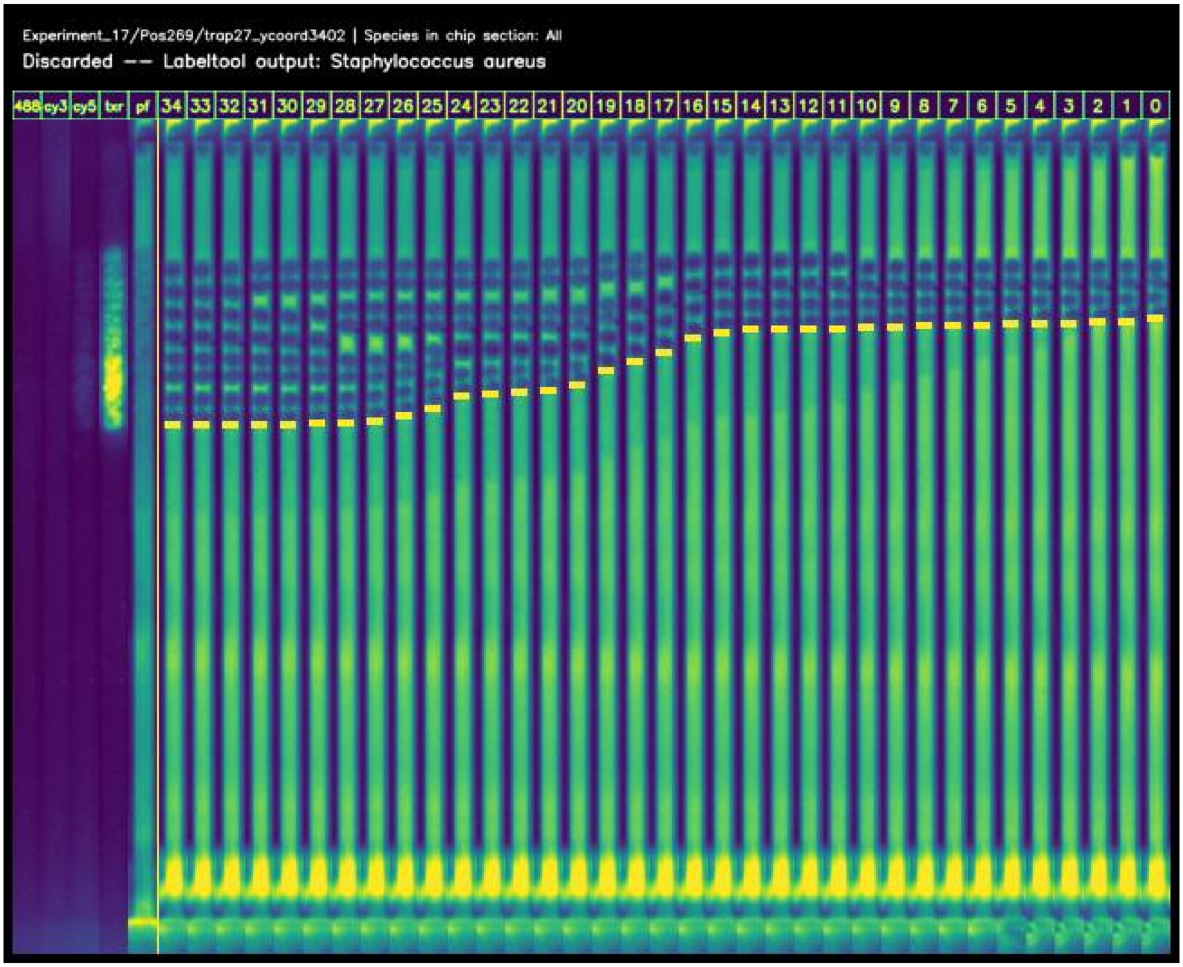
Experiment_17/Pos269/trap27_ycoord3402 | Species in chip section: All
Discarded -- Labeltool output: Staphylococcus aureus
488 cy3 cy5 txr pf 34 33 32 31 30 29 28 27 26 25 24 23 22 21 20 19 18 17 16 15 14 13 12 11 10 9 8 7 6 5 4 3 2 1 0

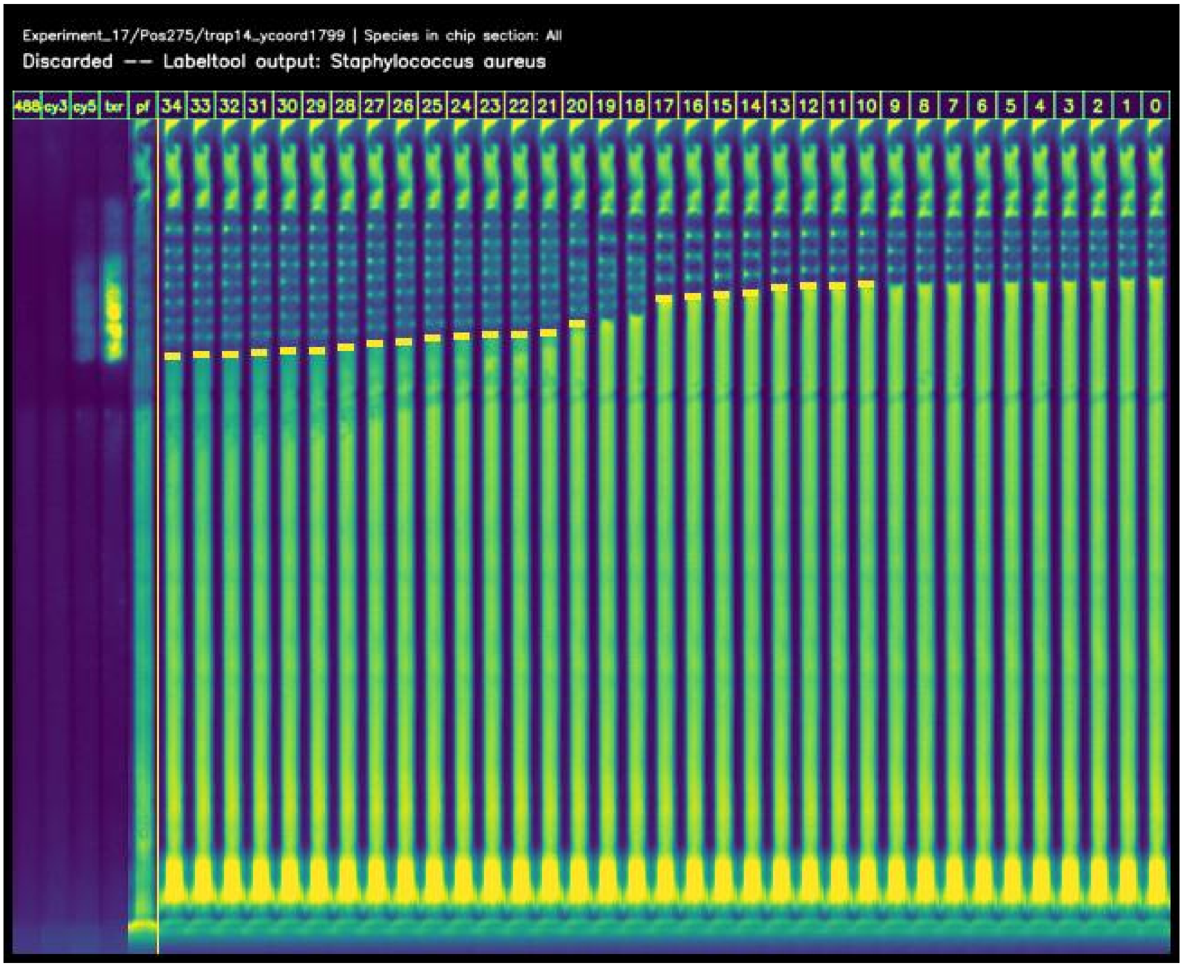
Experiment_17/Pos275/trap14_ycoord1799 | Species in chip section: All
Discarded -- Labeltool output: Staphylococcus aureus
488 cy3 cy5 txr pf 34 33 32 31 30 29 28 27 26 25 24 23 22 21 20 19 18 17 16 15 14 13 12 11 10 9 8 7 6 5 4 3 2 1 0

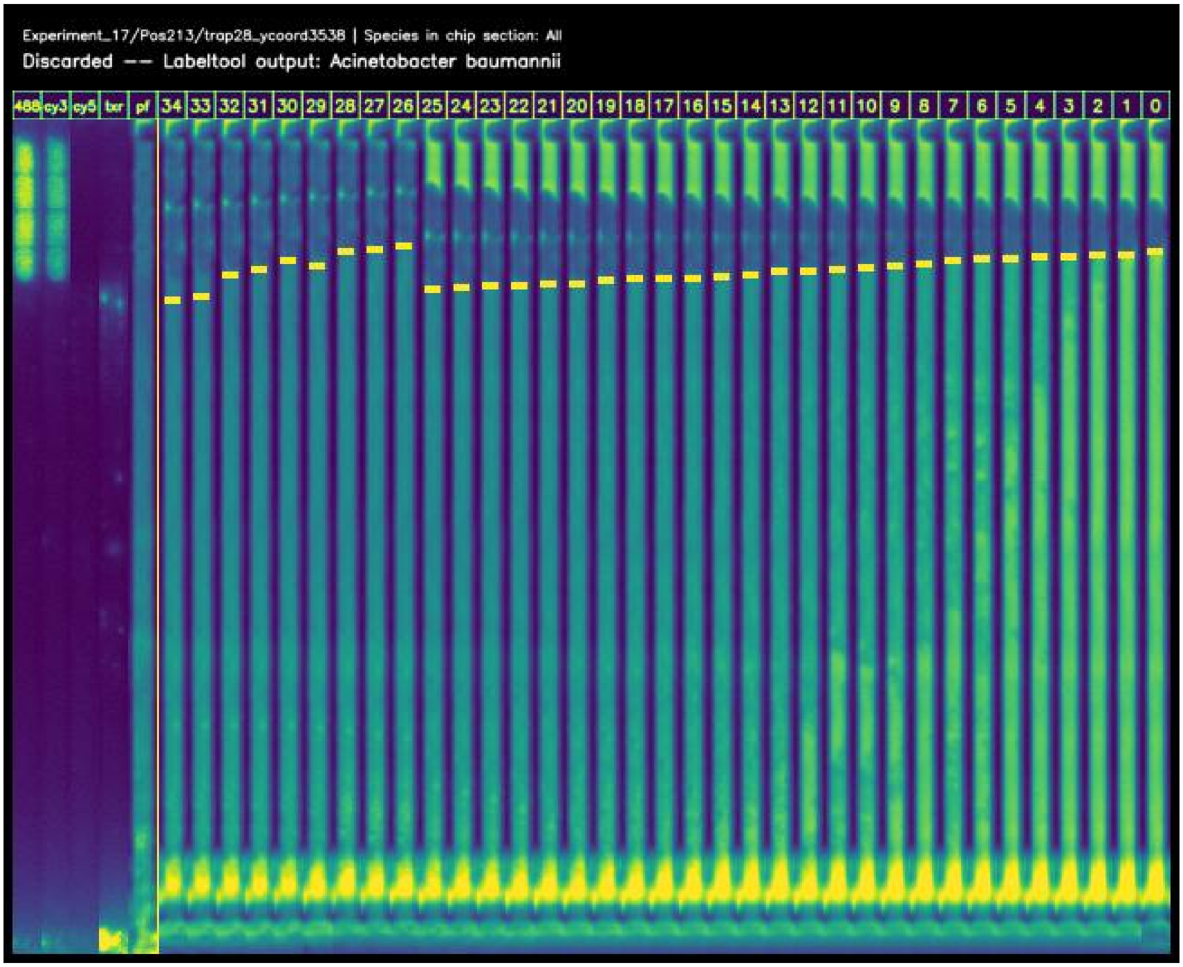
Experiment_17/Pos213/trap28_ycoord3538 | Species in chip section: All
Discarded -- Labeltool output: Acinetobacter baumannii
488 cy3 cy5 txr pf 34 33 32 31 30 29 28 27 26 25 24 23 22 21 20 19 18 17 16 15 14 13 12 11 10 9 8 7 6 5 4 3 2 1 0

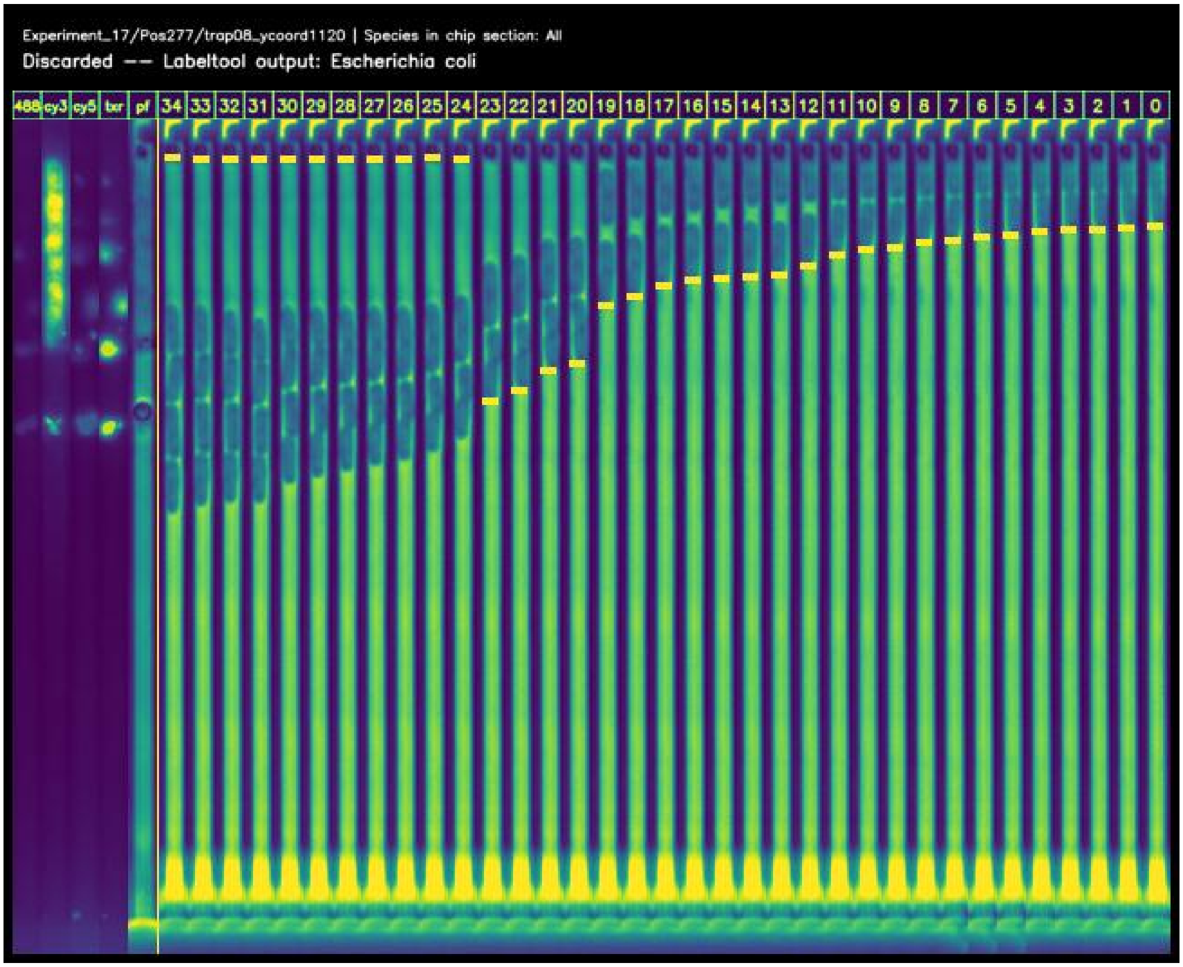
Experiment_17/Pos277/trap08_ycoord1120 | Species in chip section: All
Discarded -- Labeltool output: Escherichia coli
488 cy3 cy5 txr pf 34 33 32 31 30 29 28 27 26 25 24 23 22 21 20 19 18 17 16 15 14 13 12 11 10 9 8 7 6 5 4 3 2 1 0

Discarded due to containing multiple species

The following traps were discarded due to containing multiple species, visible through fluorescent staining, which were missed by the labeling tool. Notably, the Cy5 signal is often much weaker than the Cy3 signal. While some traps appear to contain only *E. coli* (Cy3), they actually contain both *E. coli* and *Klebsiella*, as indicated by a dim Cy5 signal.

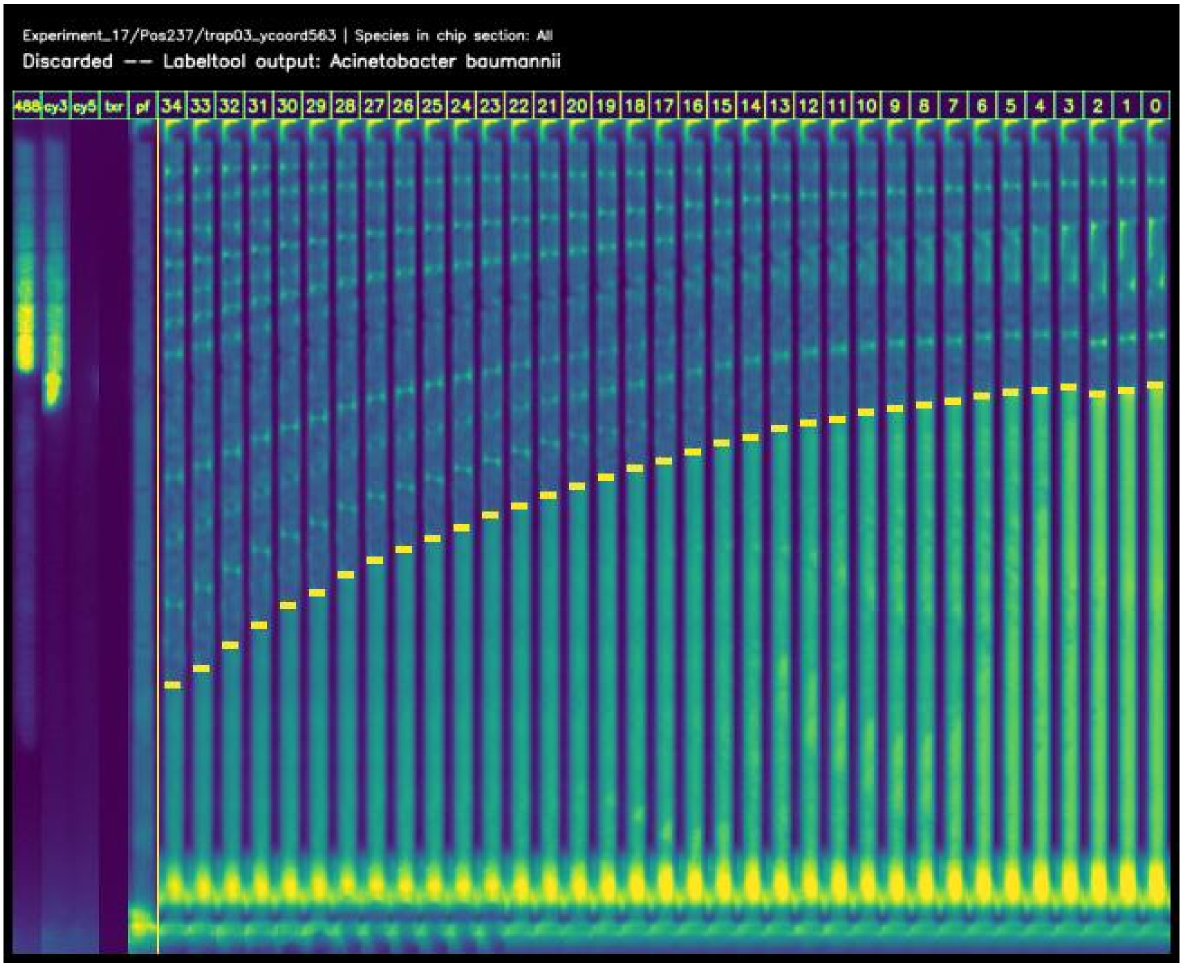

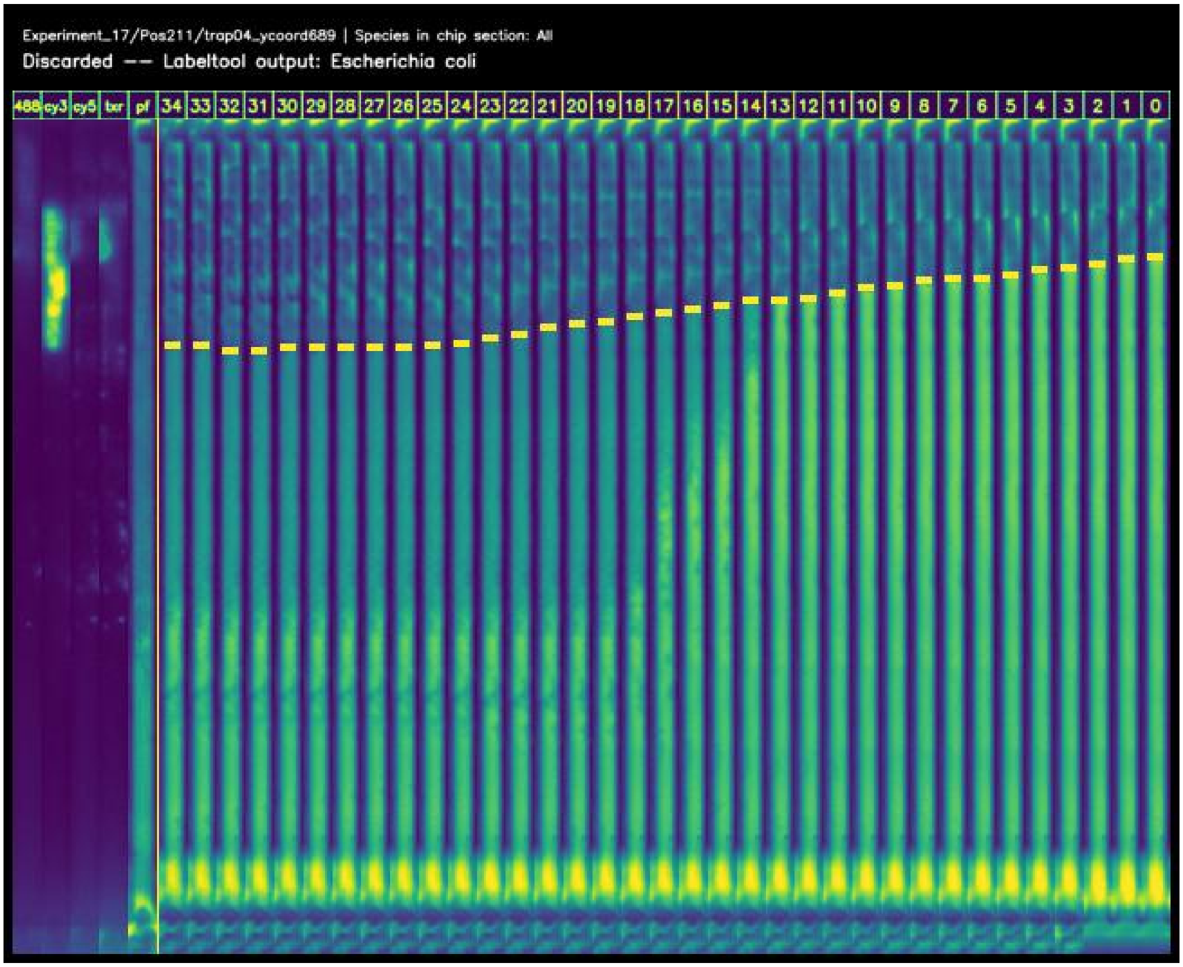
Experiment_17/Pos211/trap04_ycoord689 | Species in chip section: All
Discarded -- Labeltool output: Escherichia coli
488 cy3 cy5 txr pf 34 33 32 31 30 29 28 27 26 25 24 23 22 21 20 19 18 17 16 15 14 13 12 11 10 9 8 7 6 5 4 3 2 1 0

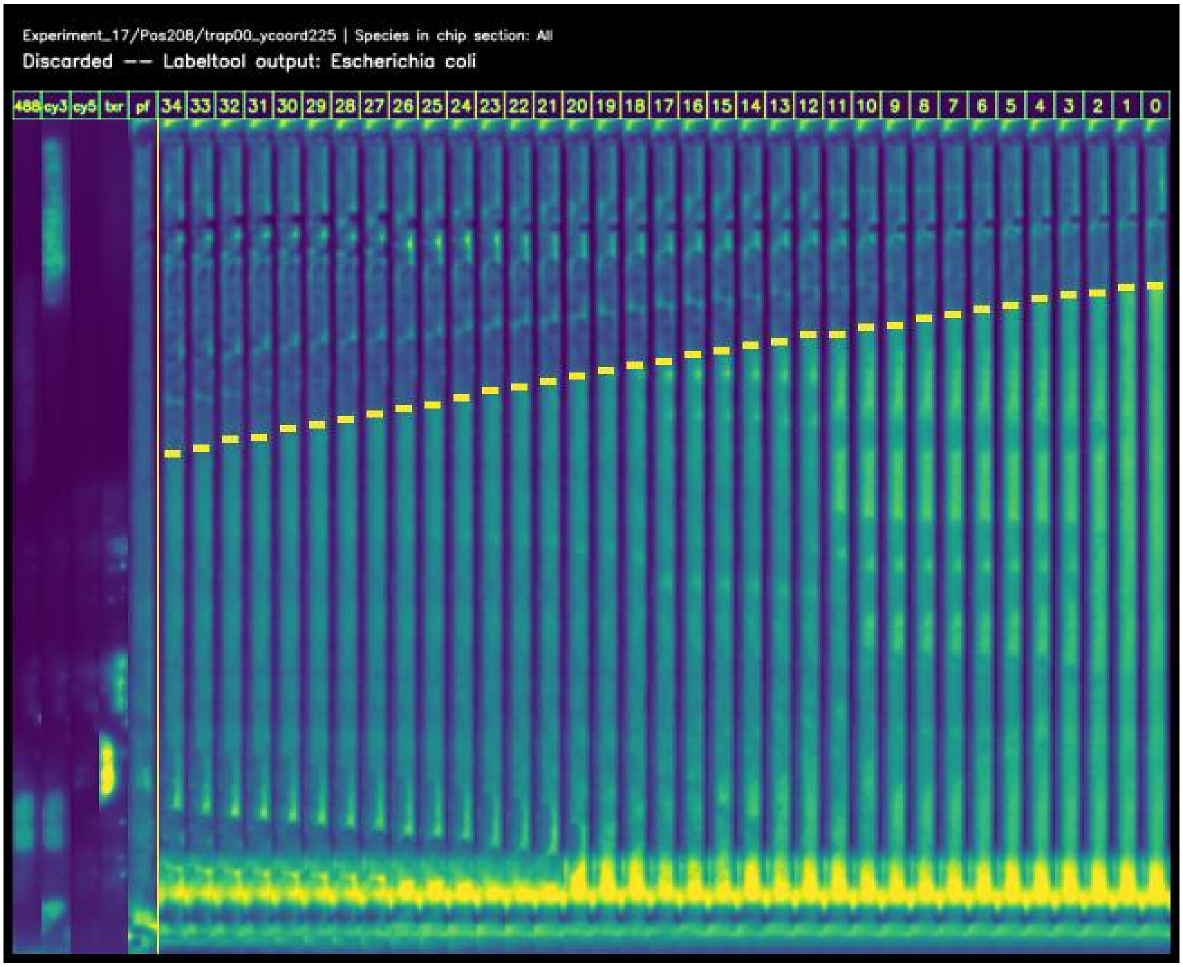
Experiment_17/Pos208/trap00_ycoord225 | Species in chip section: All
Discarded -- Labeltool output: Escherichia coli
488 cy3 cy5 txr pf 34 33 32 31 30 29 28 27 26 25 24 23 22 21 20 19 18 17 16 15 14 13 12 11 10 9 8 7 6 5 4 3 2 1 0

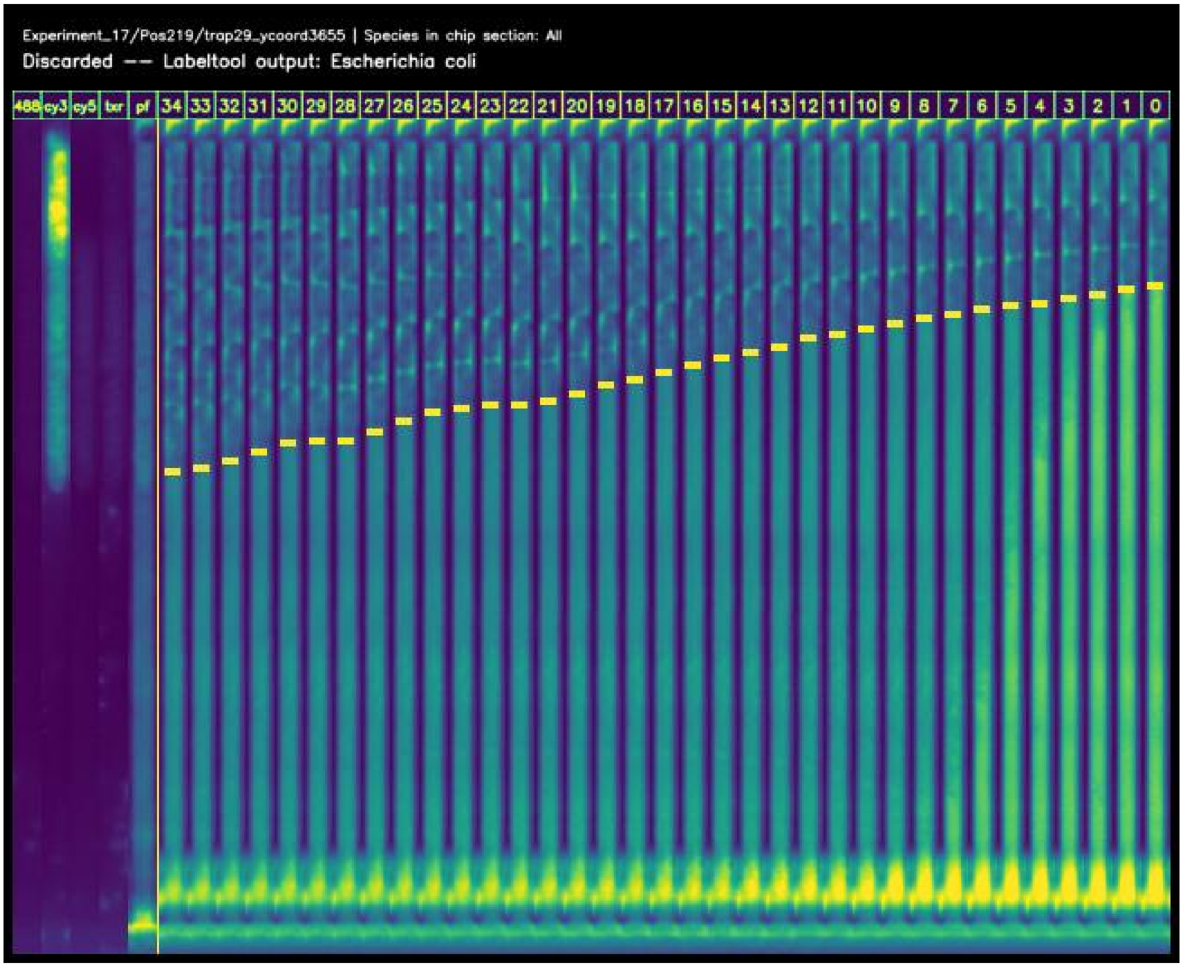
Experiment_17/Pos219/trap29_ycoord3655 | Species in chip section: All
Discarded -- Labeltool output: Escherichia coli
488 cy3 cy5 txr pf 34 33 32 31 30 29 28 27 26 25 24 23 22 21 20 19 18 17 16 15 14 13 12 11 10 9 8 7 6 5 4 3 2 1 0

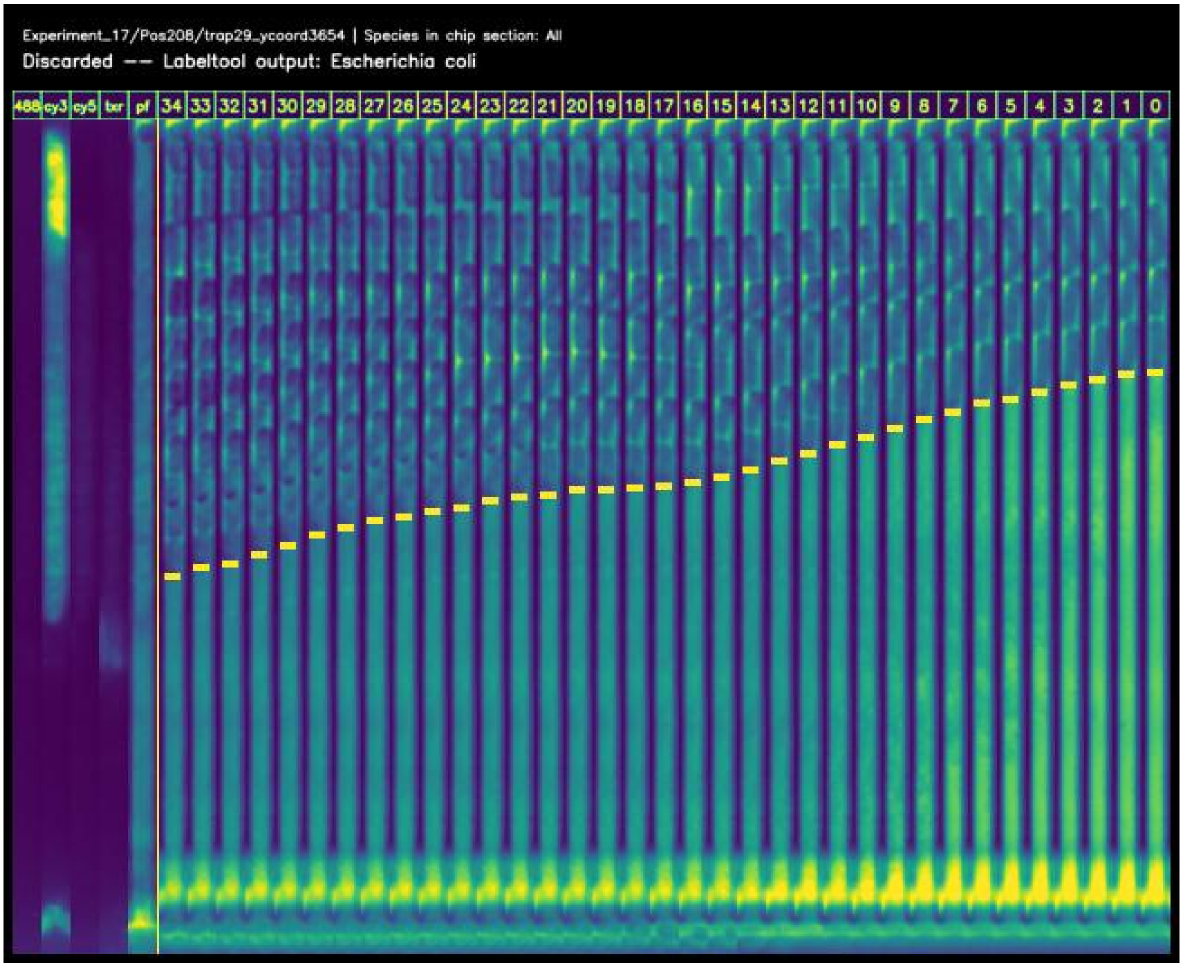
Experiment_17/Pos208/trap29_ycoord3654 | Species in chip section: All
Discarded -- Labeltool output: Escherichia coli
488 cy3 cy5 txr pf 34 33 32 31 30 29 28 27 26 25 24 23 22 21 20 19 18 17 16 15 14 13 12 11 10 9 8 7 6 5 4 3 2 1 0

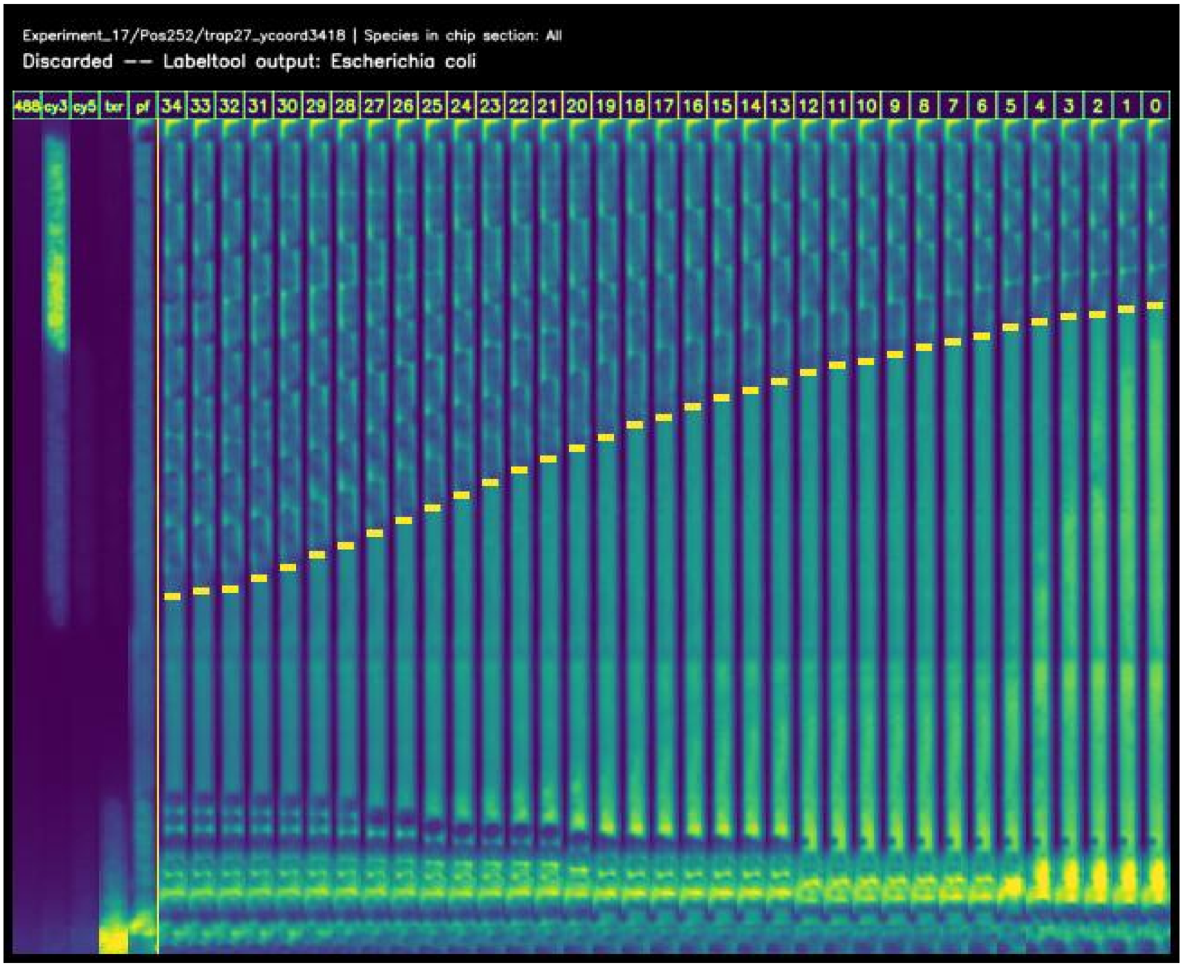
Experiment_17/Pos252/trap27_ycoord3418 | Species in chip section: All
Discarded -- Labeltool output: Escherichia coli
488 cy3 cy5 txr pf 34 33 32 31 30 29 28 27 26 25 24 23 22 21 20 19 18 17 16 15 14 13 12 11 10 9 8 7 6 5 4 3 2 1 0

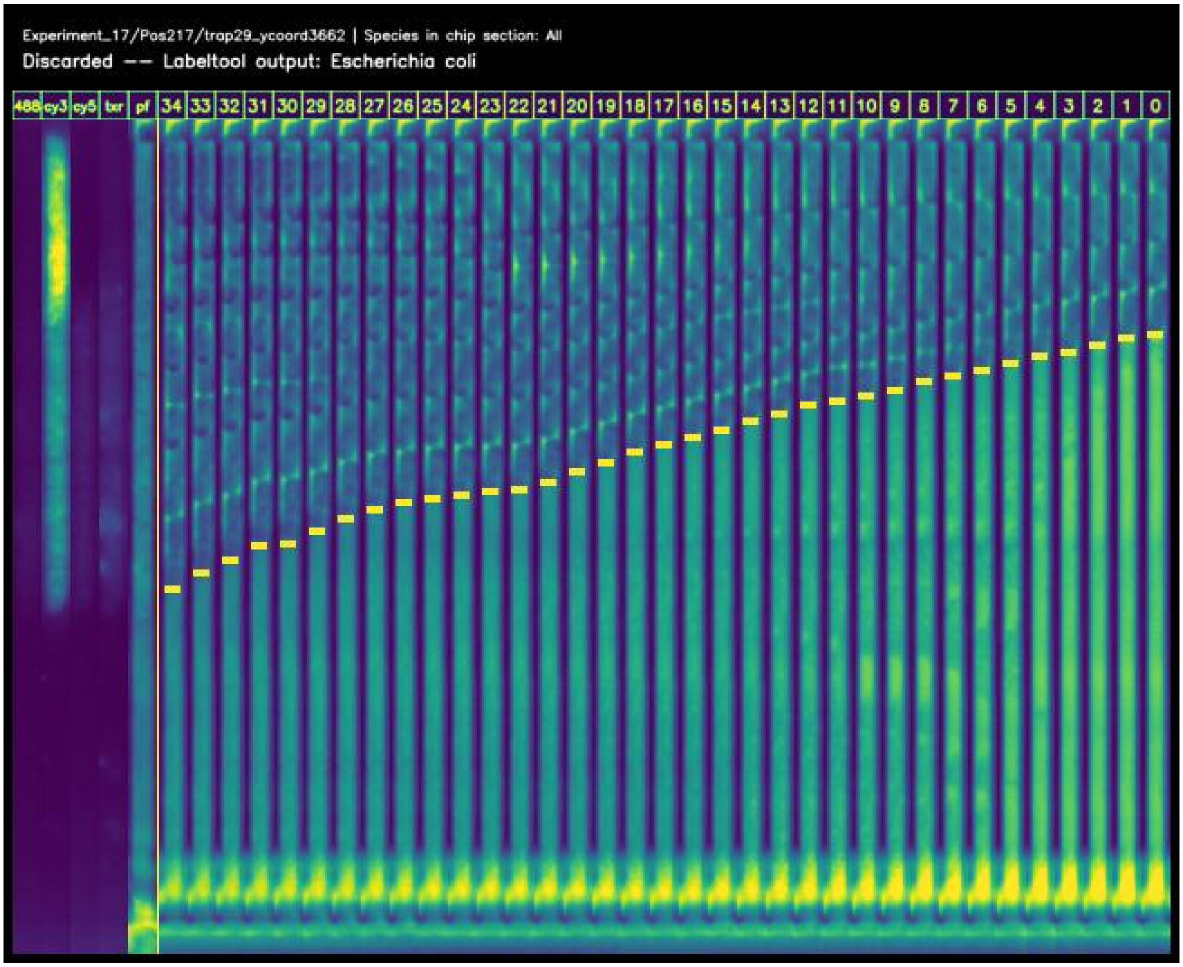
Experiment_17/Pos217/trap29_ycoord3662 | Species in chip section: All
Discarded -- Labeltool output: Escherichia coli
488 cy3 cy5 txr pf 34 33 32 31 30 29 28 27 26 25 24 23 22 21 20 19 18 17 16 15 14 13 12 11 10 9 8 7 6 5 4 3 2 1 0

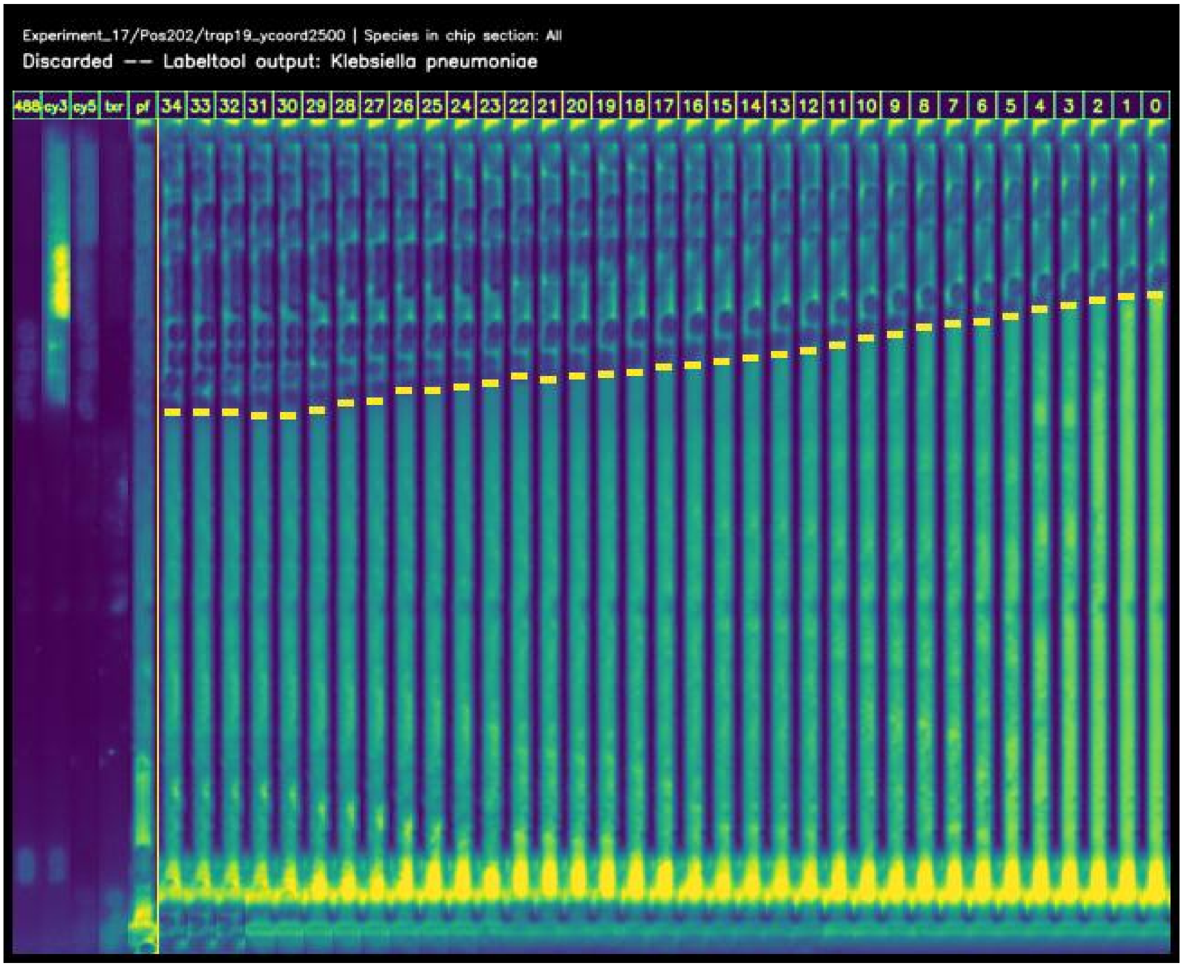
Experiment_17/Pos202/trap19_ycoord2500 | Species in chip section: All
Discarded -- Labeltool output: Klebsiella pneumoniae
488 cy3 cy5 txr pf 34 33 32 31 30 29 28 27 26 25 24 23 22 21 20 19 18 17 16 15 14 13 12 11 10 9 8 7 6 5 4 3 2 1 0

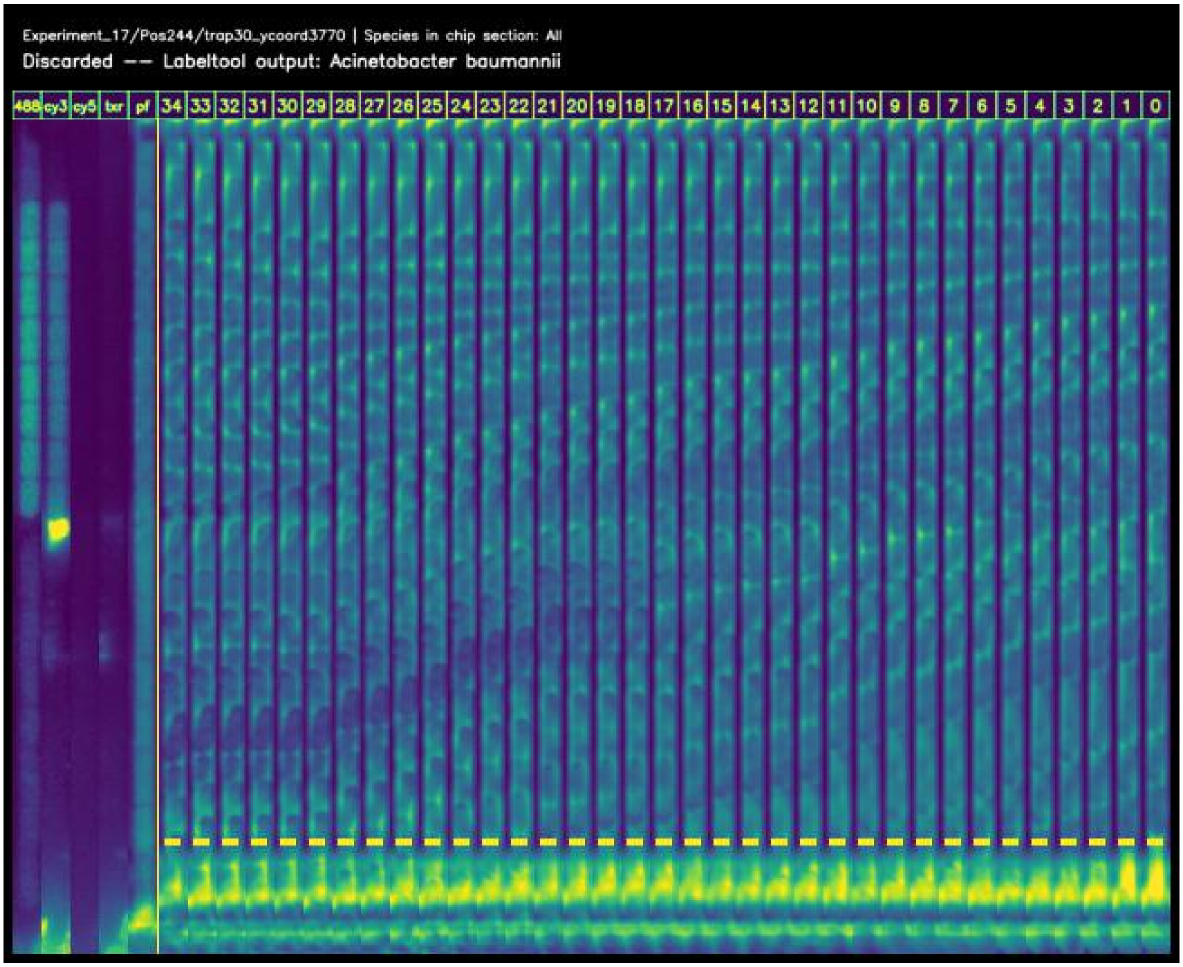
Experiment_17/Pos244/trap30_ycoord3770 | Species in chip section: All
Discarded -- Labeltool output: Acinetobacter baumannii
488 cy3 cy5 txr pf 34 33 32 31 30 29 28 27 26 25 24 23 22 21 20 19 18 17 16 15 14 13 12 11 10 9 8 7 6 5 4 3 2 1 0

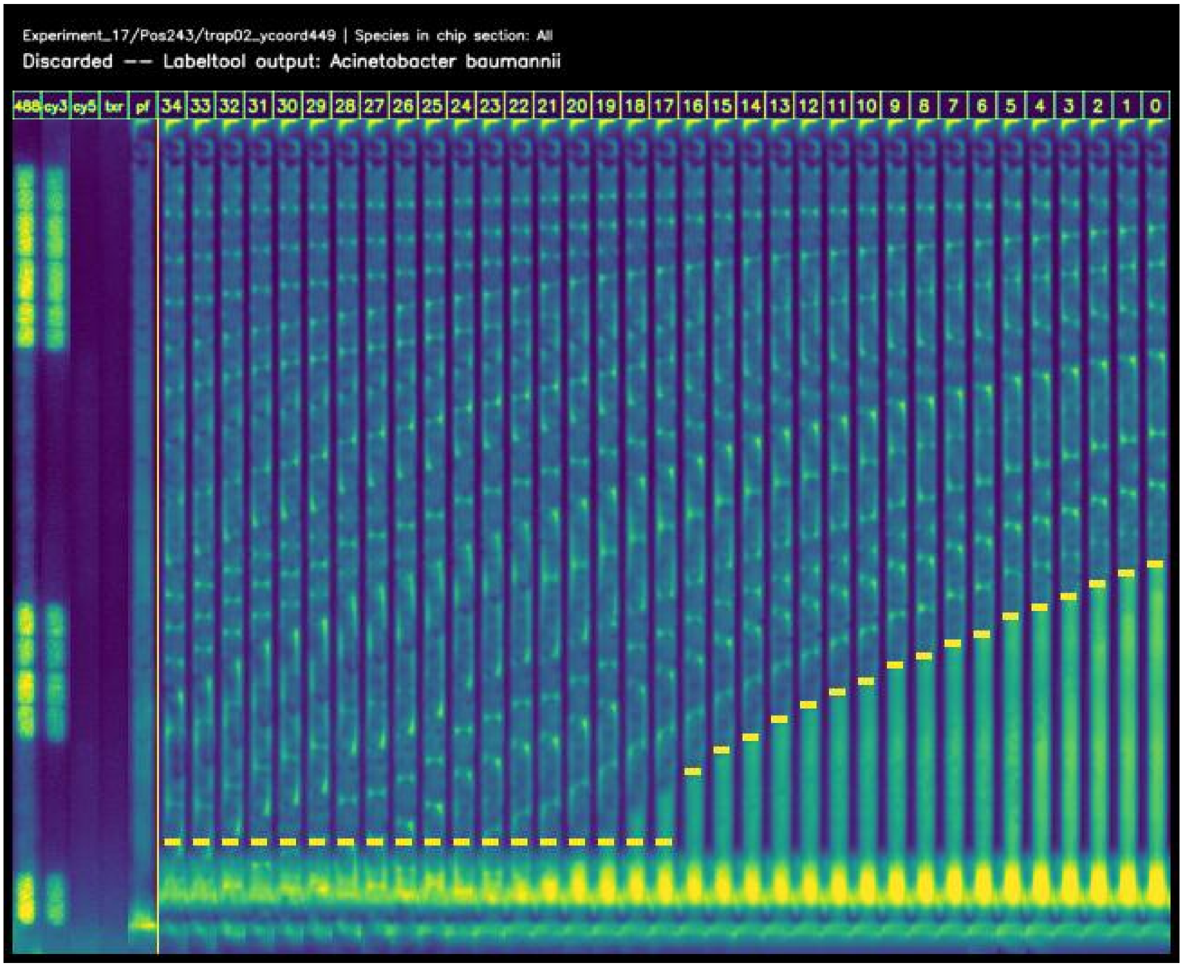
Experiment_17/Pos243/trap02_ycoord449 | Species in chip section: All
Discarded -- Labeltool output: Acinetobacter baumannii
488 cy3 cy5 txr pf 34 33 32 31 30 29 28 27 26 25 24 23 22 21 20 19 18 17 16 15 14 13 12 11 10 9 8 7 6 5 4 3 2 1 0

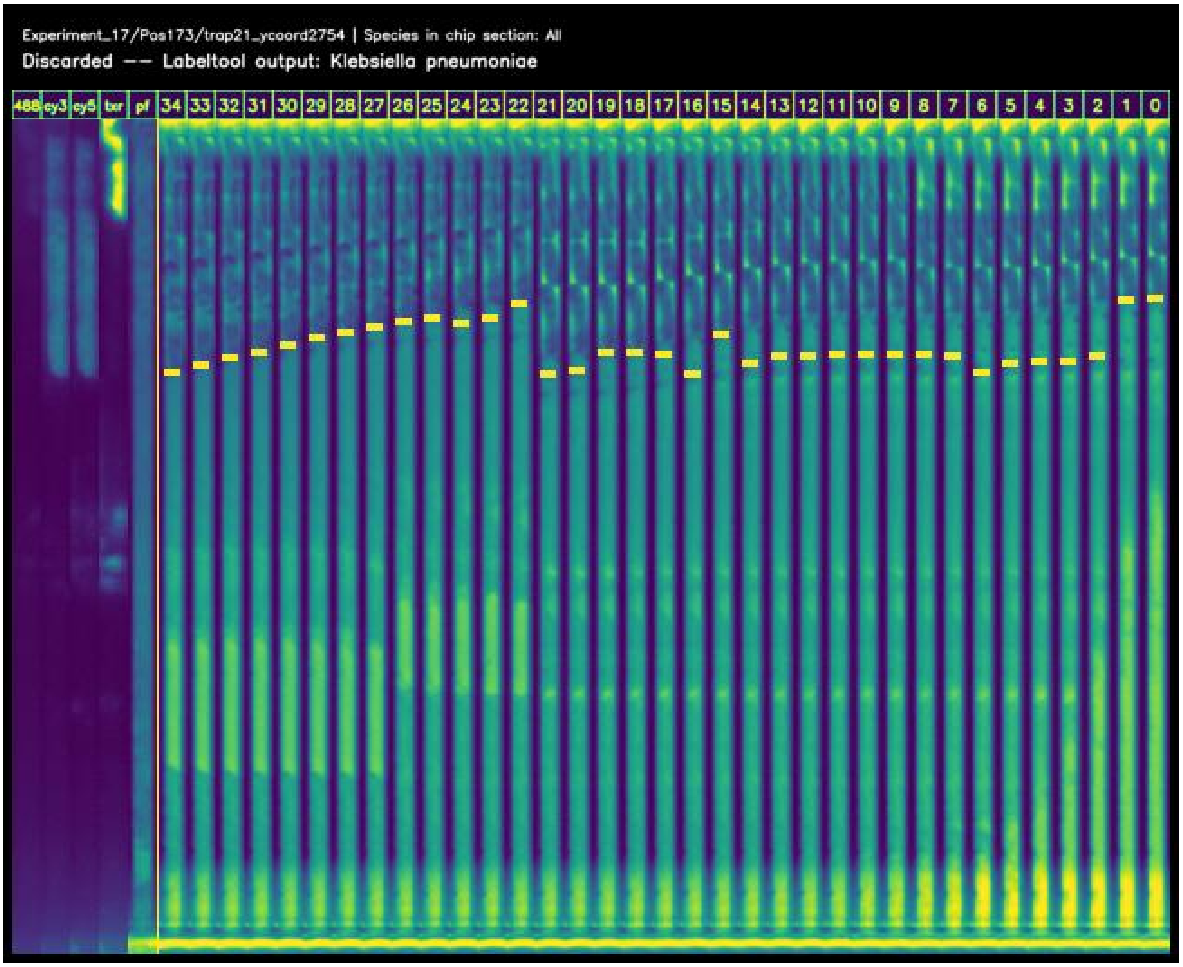
Experiment_17/Pos173/trap21_ycoord2754 | Species in chip section: All
Discarded -- Labeltool output: Klebsiella pneumoniae
488 cy3 cy5 txr pf 34 33 32 31 30 29 28 27 26 25 24 23 22 21 20 19 18 17 16 15 14 13 12 11 10 9 8 7 6 5 4 3 2 1 0

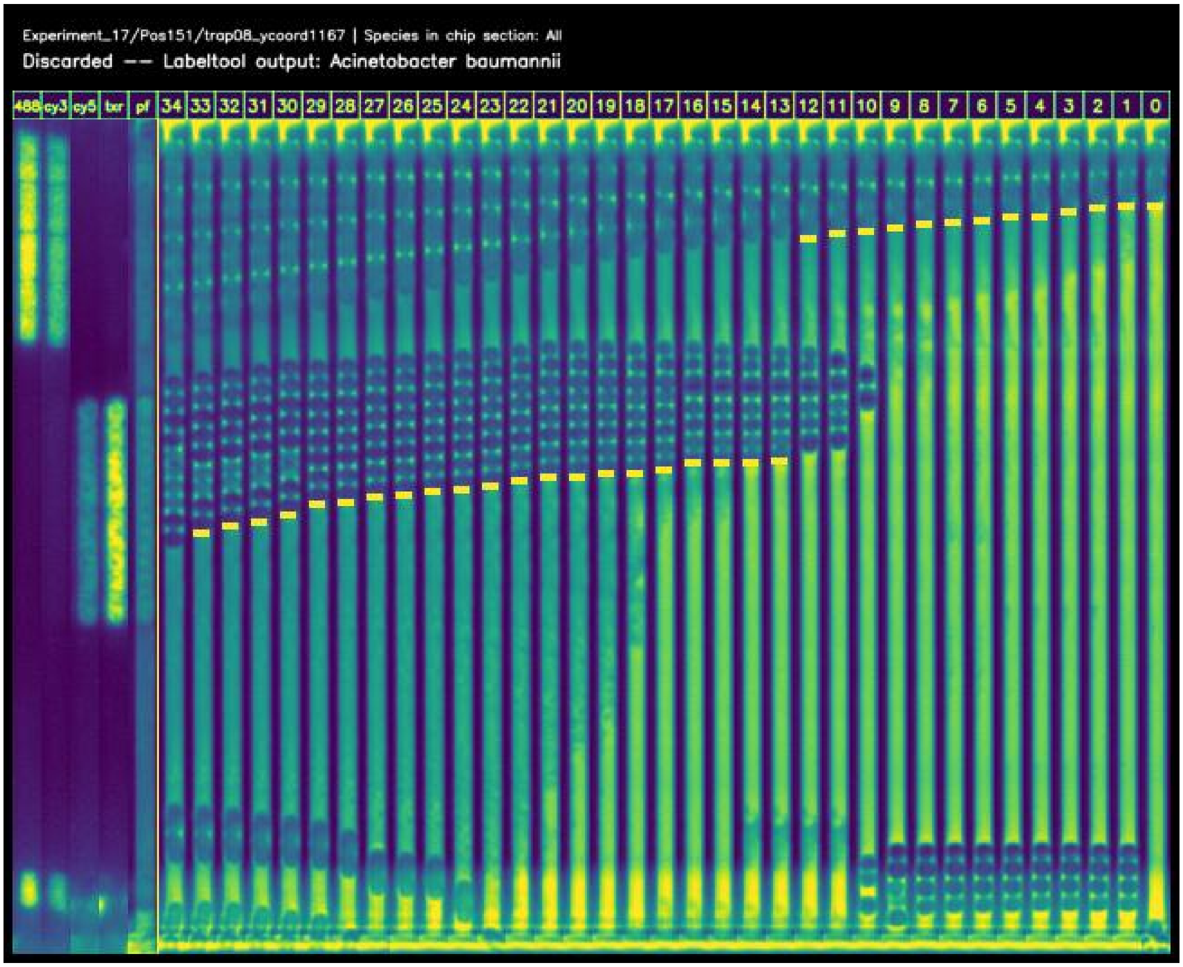
Experiment_17/Pos151/trap08_ycoord1167 | Species in chip section: All
Discarded -- Labeltool output: Acinetobacter baumannii
488 cy3 cy5 txr pf 34 33 32 31 30 29 28 27 26 25 24 23 22 21 20 19 18 17 16 15 14 13 12 11 10 9 8 7 6 5 4 3 2 1 0

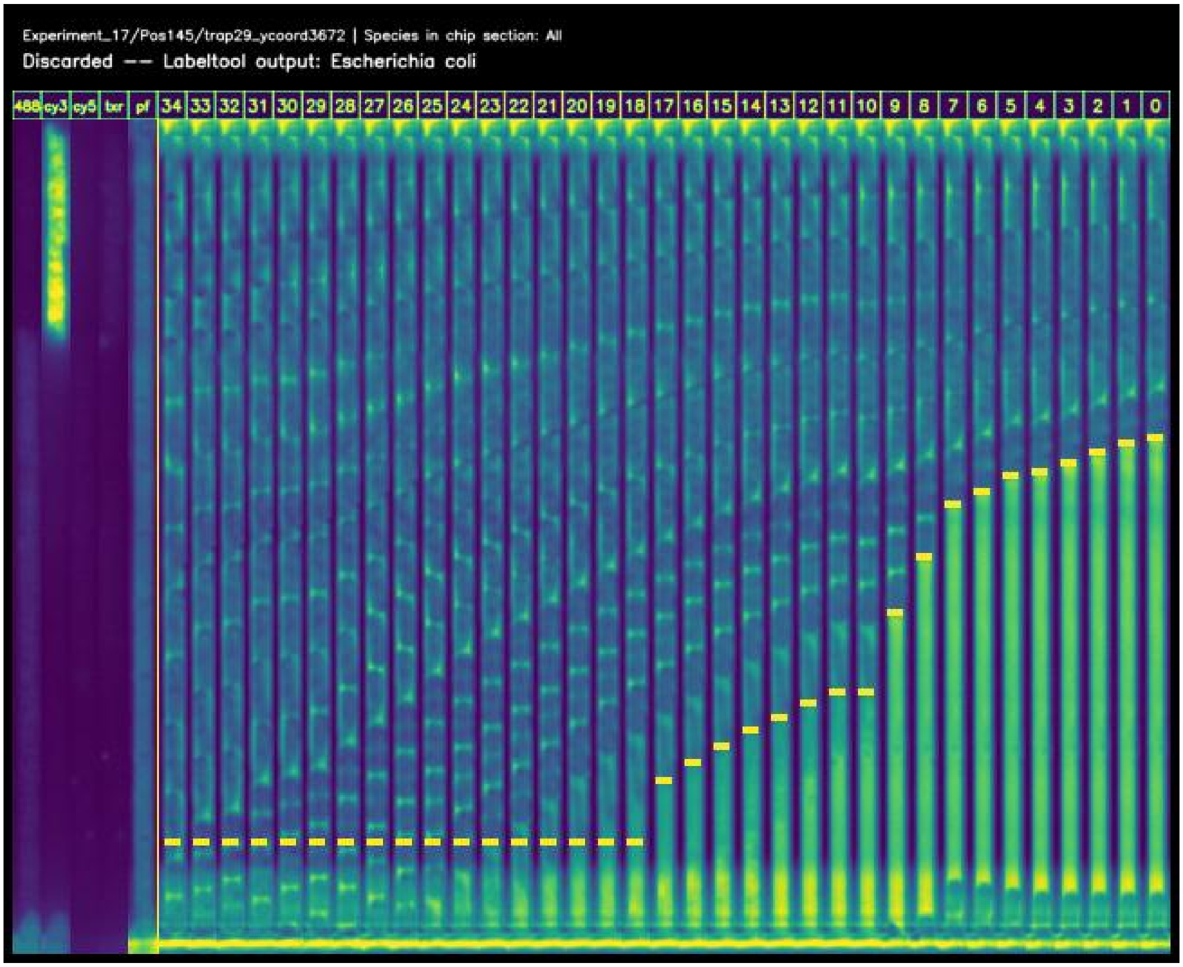
Experiment_17/Pos145/trap29_ycoord3672 | Species in chip section: All
Discarded -- Labeltool output: Escherichia coli
488 cy3 cy5 txr pf 34 33 32 31 30 29 28 27 26 25 24 23 22 21 20 19 18 17 16 15 14 13 12 11 10 9 8 7 6 5 4 3 2 1 0

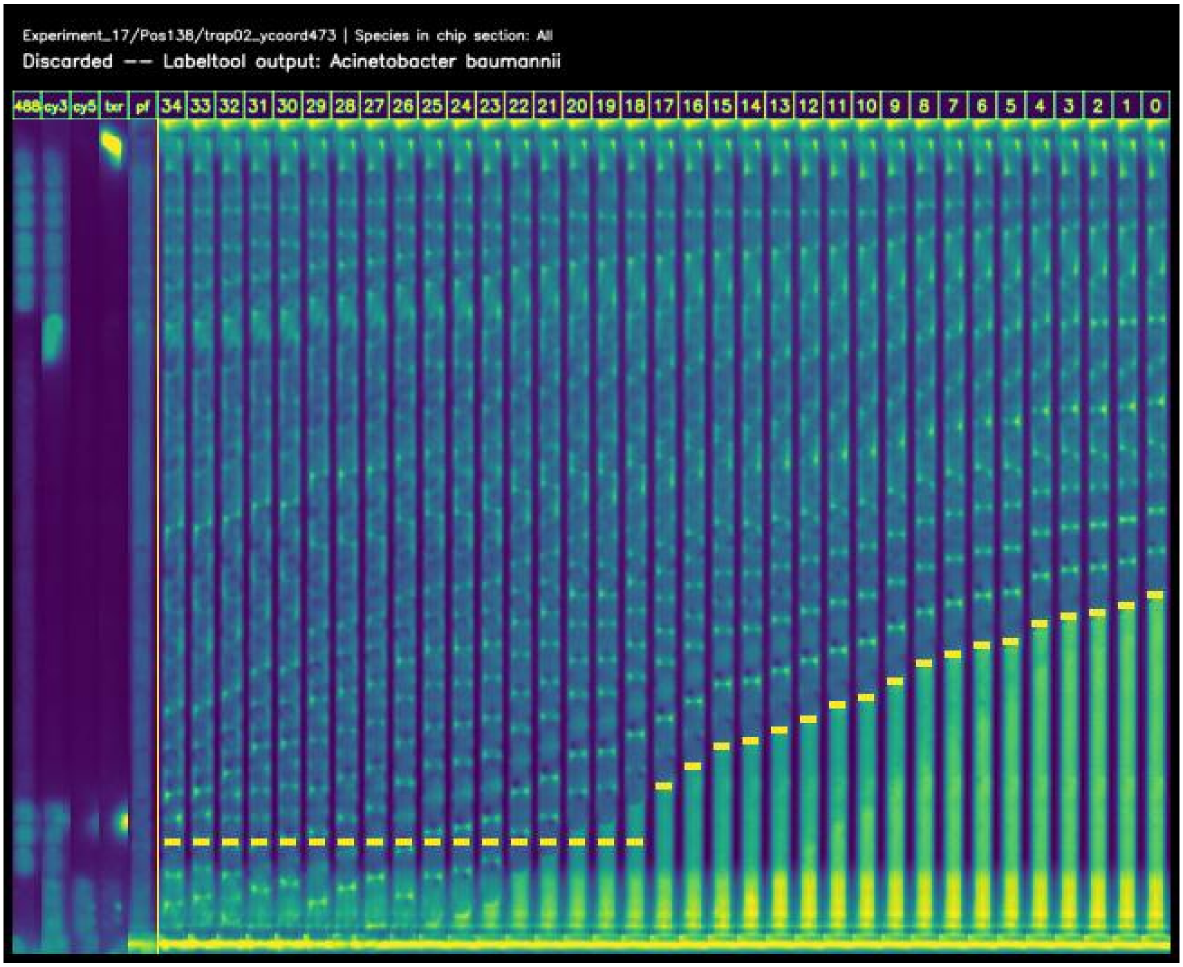
Experiment_17/Pos138/trap02_ycoord473 | Species in chip section: All
Discarded -- Labeltool output: Acinetobacter baumannii
488 cy3 cy5 txr pf 34 33 32 31 30 29 28 27 26 25 24 23 22 21 20 19 18 17 16 15 14 13 12 11 10 9 8 7 6 5 4 3 2 1 0

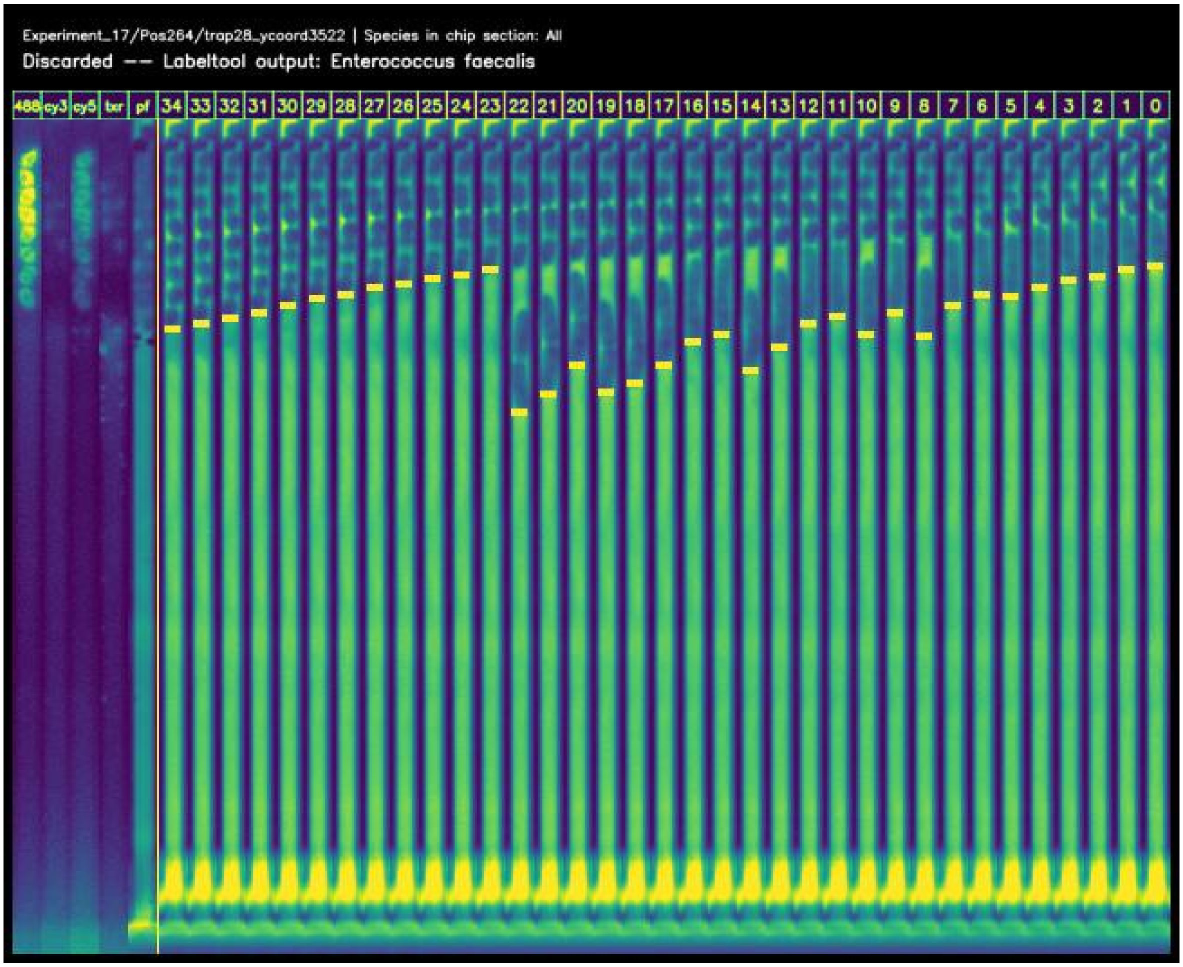
Experiment_17/Pos264/trap28_ycoord3522 | Species in chip section: All
Discarded -- Labeltool output: Enterococcus faecalis
488 cy3 cy5 txr pf 34 33 32 31 30 29 28 27 26 25 24 23 22 21 20 19 18 17 16 15 14 13 12 11 10 9 8 7 6 5 4 3 2 1 0

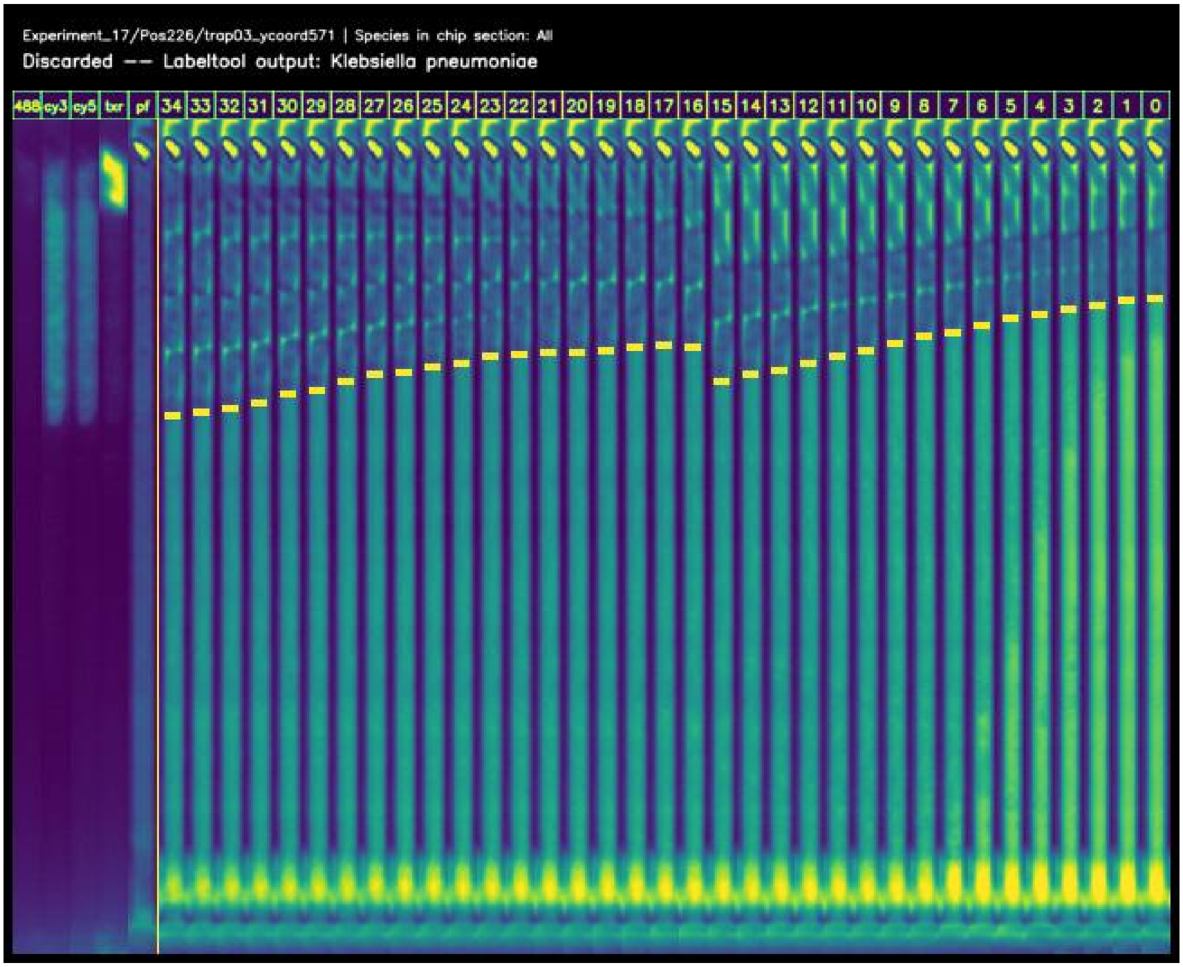
Experiment_17/Pos226/trap03_ycoord571 | Species in chip section: All
Discarded -- Labeltool output: Klebsiella pneumoniae
488 cy3 cy5 txr pf 34 33 32 31 30 29 28 27 26 25 24 23 22 21 20 19 18 17 16 15 14 13 12 11 10 9 8 7 6 5 4 3 2 1 0

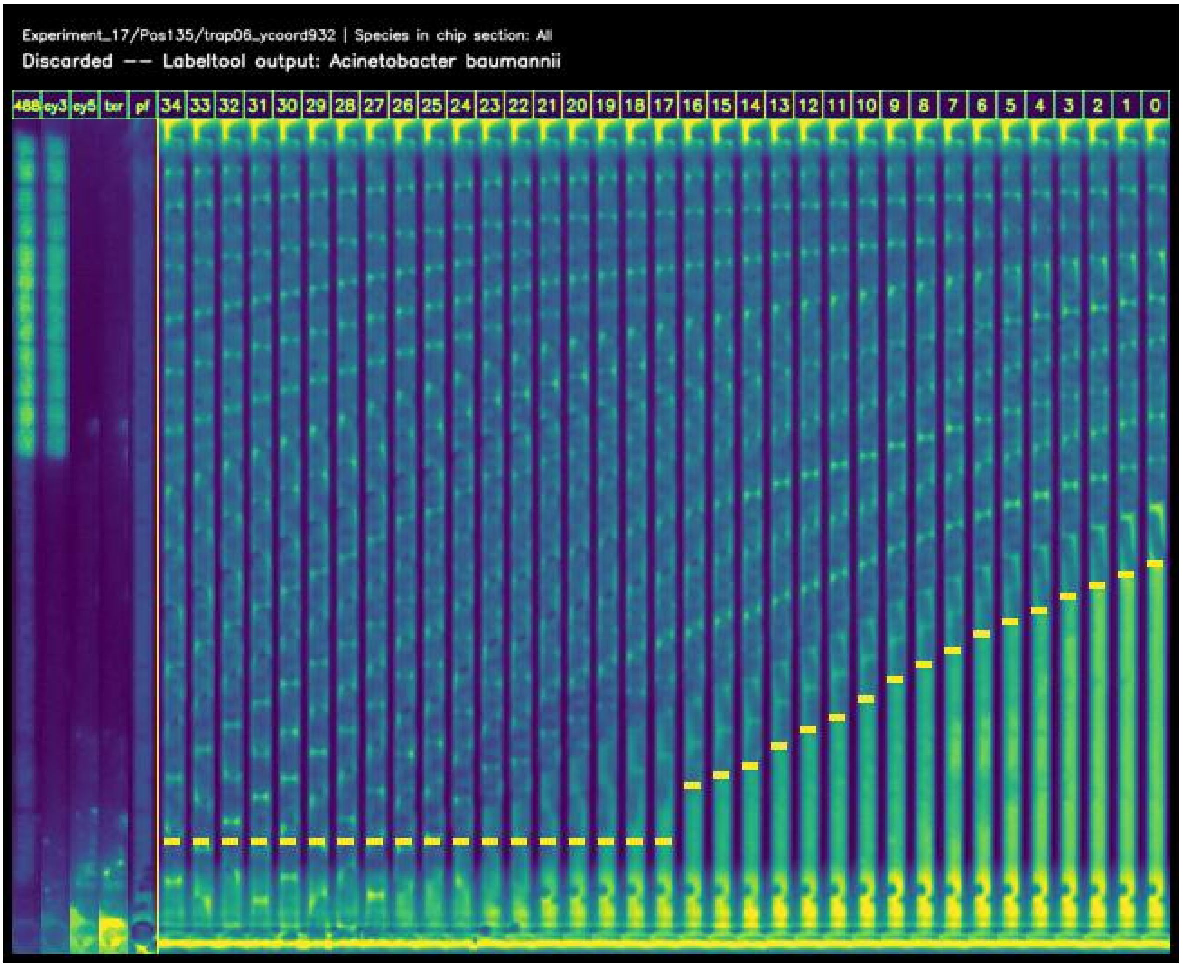
Experiment_17/Pos135/trap06_ycoord932 | Species in chip section: All
Discarded -- Labeltool output: Acinetobacter baumannii
488 cy3 cy5 txr pf 34 33 32 31 30 29 28 27 26 25 24 23 22 21 20 19 18 17 16 15 14 13 12 11 10 9 8 7 6 5 4 3 2 1 0

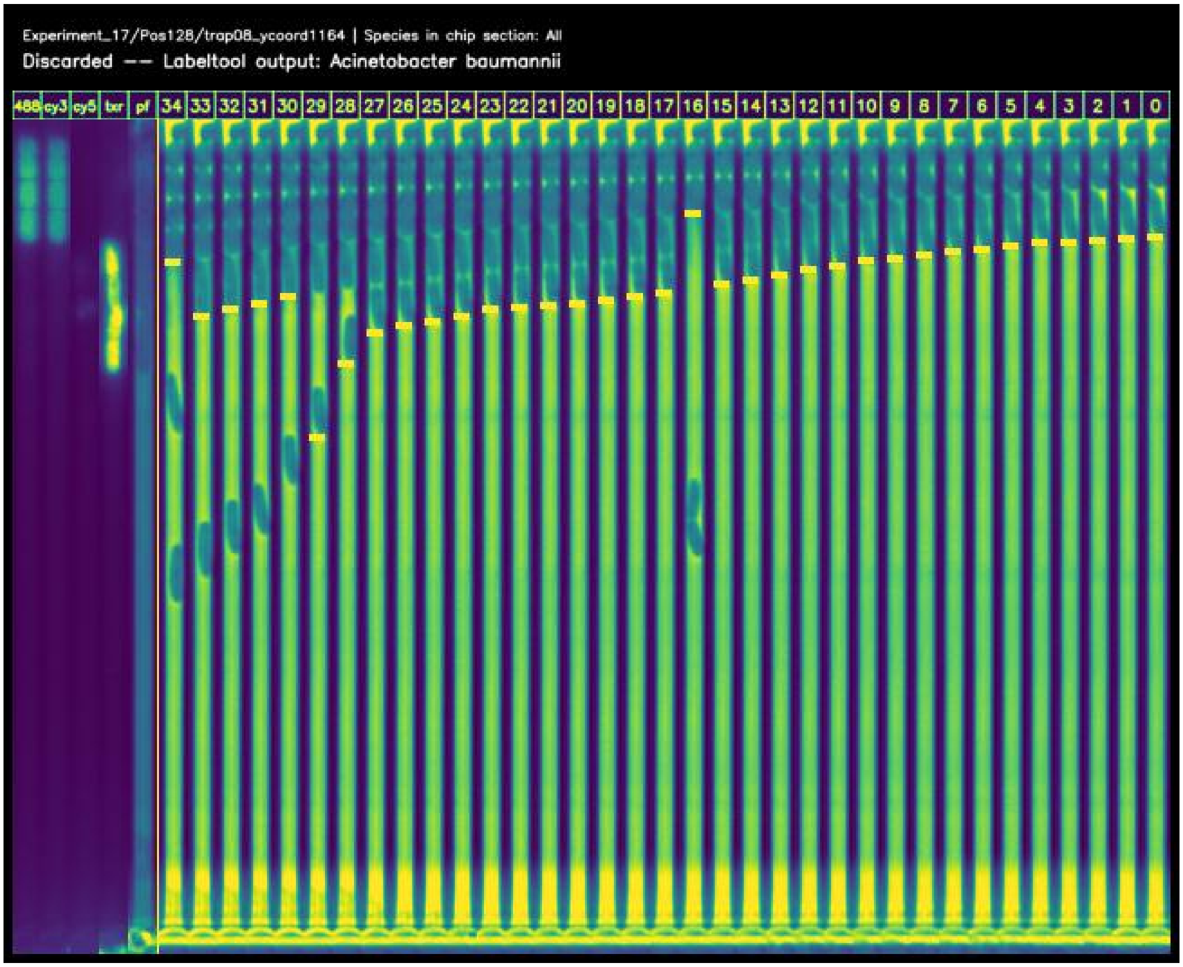

Experiment_17/Pos128/trap08_ycoord1164 | Species in chip section: All
Discarded -- Labeltool output: Acinetobacter baumannii
488 cy3 cy5 txr pf 34 33 32 31 30 29 28 27 26 25 24 23 22 21 20 19 18 17 16 15 14 13 12 11 10 9 8 7 6 5 4 3 2 1 0

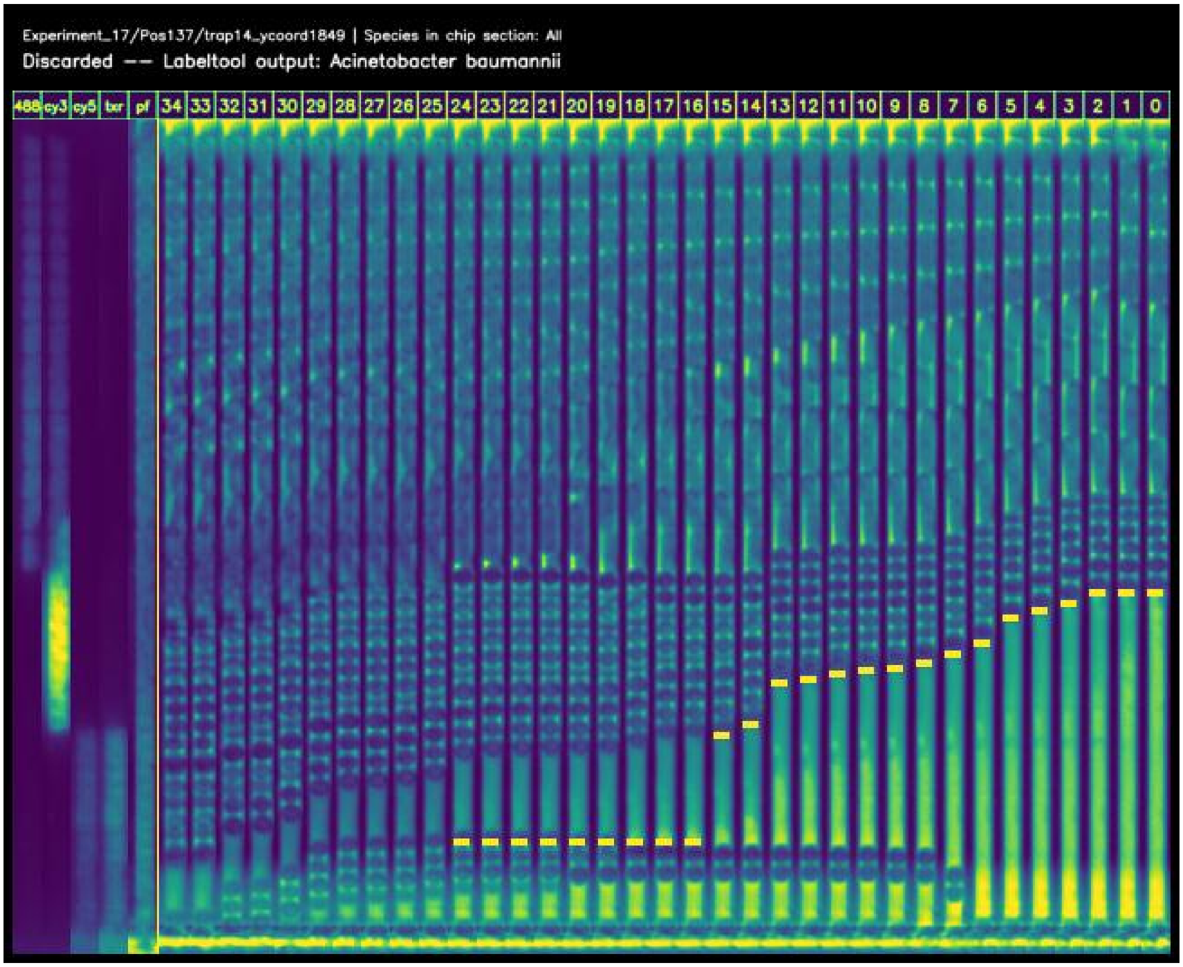

Experiment_17/Pos137/trap14_ycoord1849 | Species in chip section: All
Discarded -- Labeltool output: Acinetobacter baumannii
488 cy3 cy5 txr pf 34 33 32 31 30 29 28 27 26 25 24 23 22 21 20 19 18 17 16 15 14 13 12 11 10 9 8 7 6 5 4 3 2 1 0

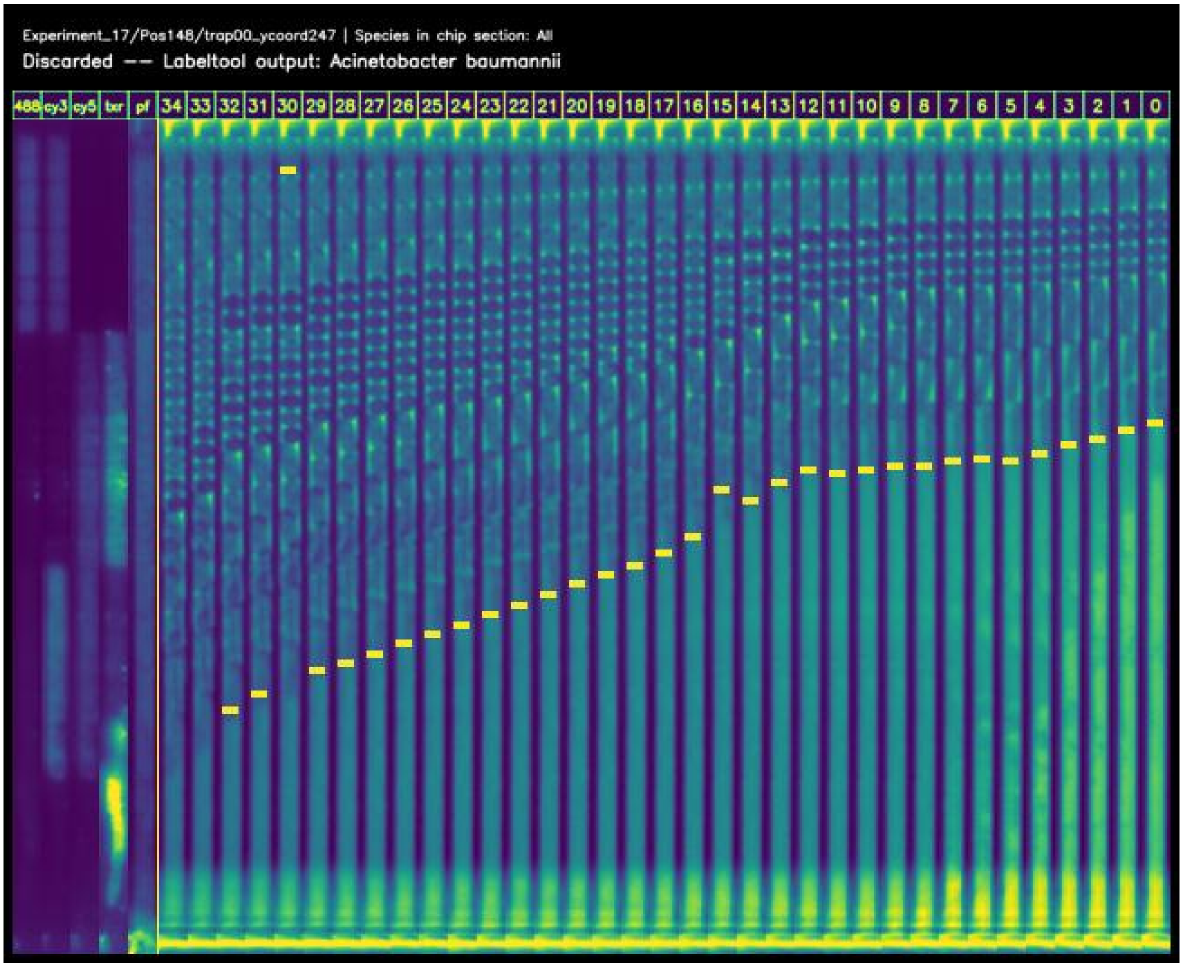

Experiment_17/Pos148/trap00_ycoord247 | Species in chip section: All
Discarded -- Labeltool output: Acinetobacter baumannii
488 cy3 cy5 txr pf 34 33 32 31 30 29 28 27 26 25 24 23 22 21 20 19 18 17 16 15 14 13 12 11 10 9 8 7 6 5 4 3 2 1 0

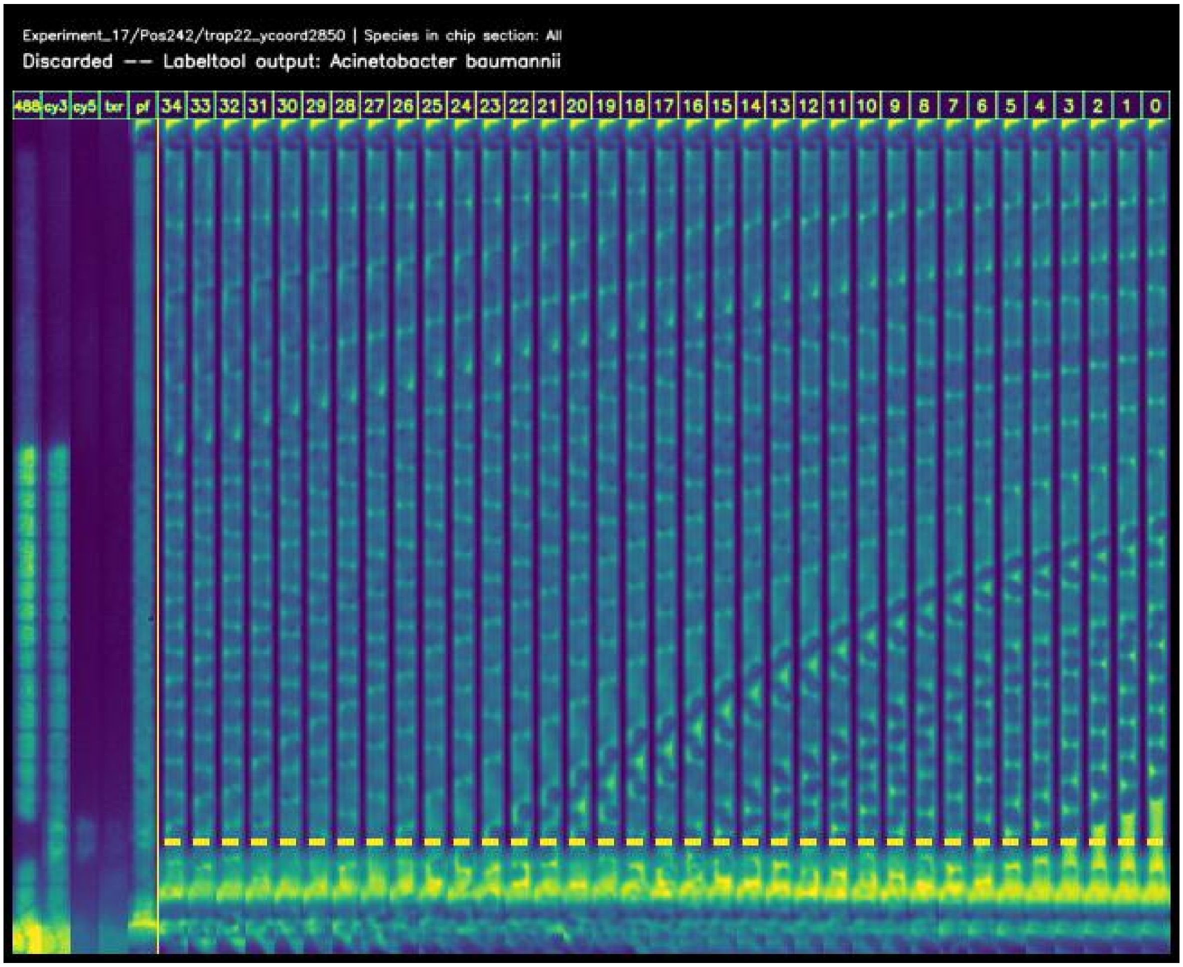

Experiment_17/Pos242/trap22_ycoord2850 | Species in chip section: All
Discarded -- Labeltool output: Acinetobacter baumannii
488 cy3 cy5 txr pf 34 33 32 31 30 29 28 27 26 25 24 23 22 21 20 19 18 17 16 15 14 13 12 11 10 9 8 7 6 5 4 3 2 1 0

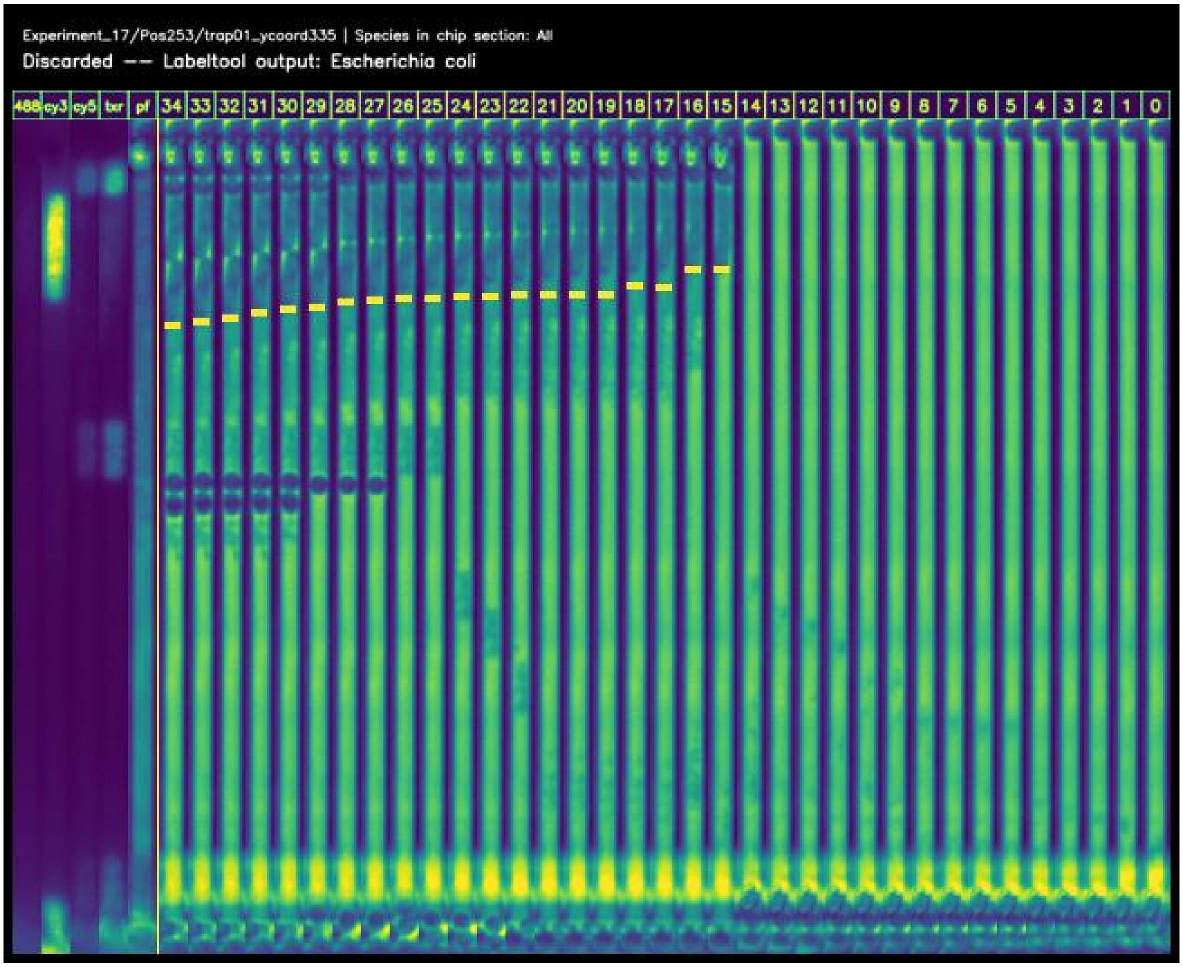
Experiment_17/Pos253/trap01_ycoord335 | Species in chip section: All
Discarded -- Labeltool output: Escherichia coli
488 cy3 cy5 txr pf 34 33 32 31 30 29 28 27 26 25 24 23 22 21 20 19 18 17 16 15 14 13 12 11 10 9 8 7 6 5 4 3 2 1 0

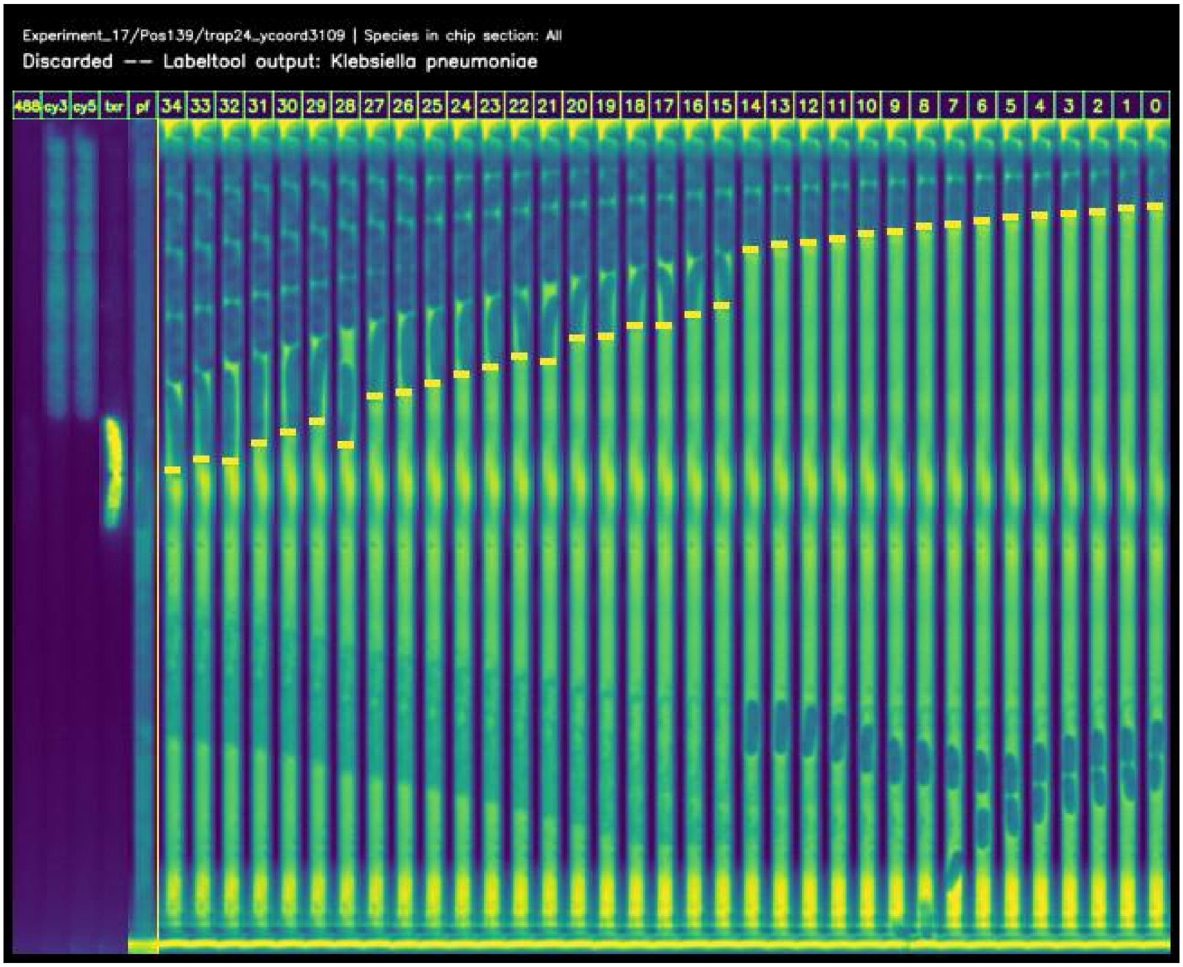
Experiment_17/Pos139/trap24_ycoord3109 | Species in chip section: All
Discarded -- Labeltool output: Klebsiella pneumoniae
488 cy3 cy5 txr pf 34 33 32 31 30 29 28 27 26 25 24 23 22 21 20 19 18 17 16 15 14 13 12 11 10 9 8 7 6 5 4 3 2 1 0

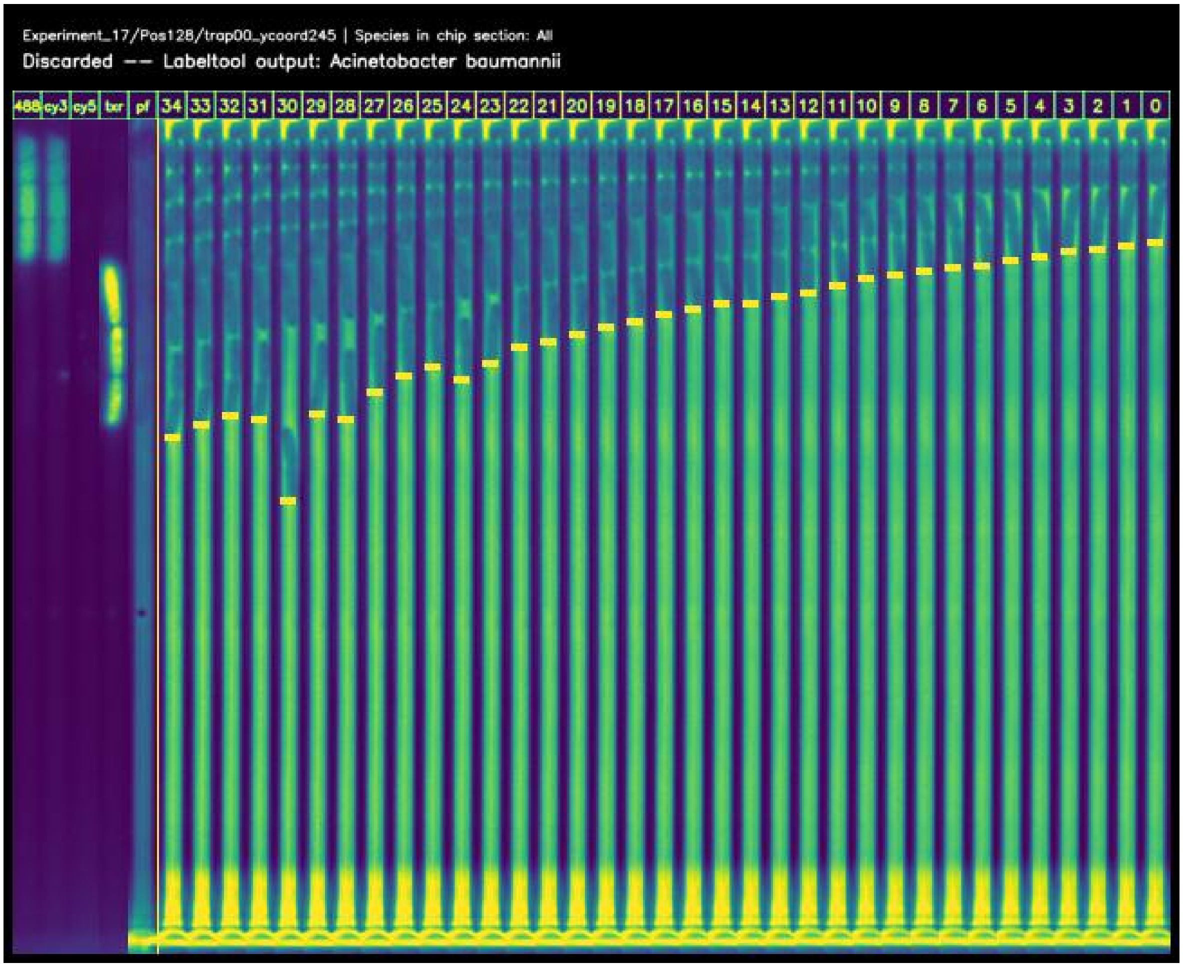
Experiment_17/Pos128/trap00_ycoord245 | Species in chip section: All
Discarded -- Labeltool output: Acinetobacter baumannii
488 cy3 cy5 txr pf 34 33 32 31 30 29 28 27 26 25 24 23 22 21 20 19 18 17 16 15 14 13 12 11 10 9 8 7 6 5 4 3 2 1 0

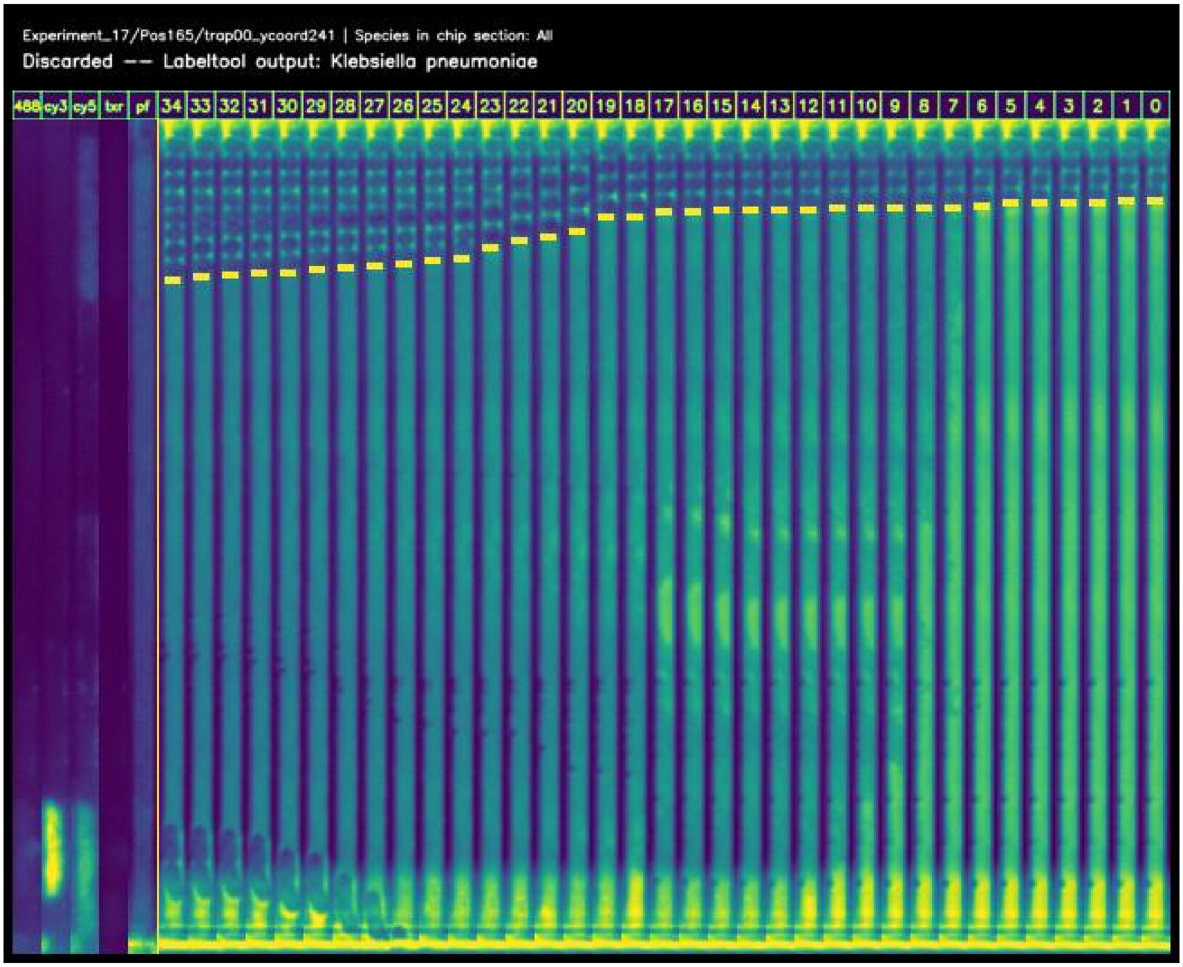
Experiment_17/Pos165/trap00_ycoord241 | Species in chip section: All
Discarded -- Labeltool output: Klebsiella pneumoniae
488 cy3 cy5 txr pf 34 33 32 31 30 29 28 27 26 25 24 23 22 21 20 19 18 17 16 15 14 13 12 11 10 9 8 7 6 5 4 3 2 1 0

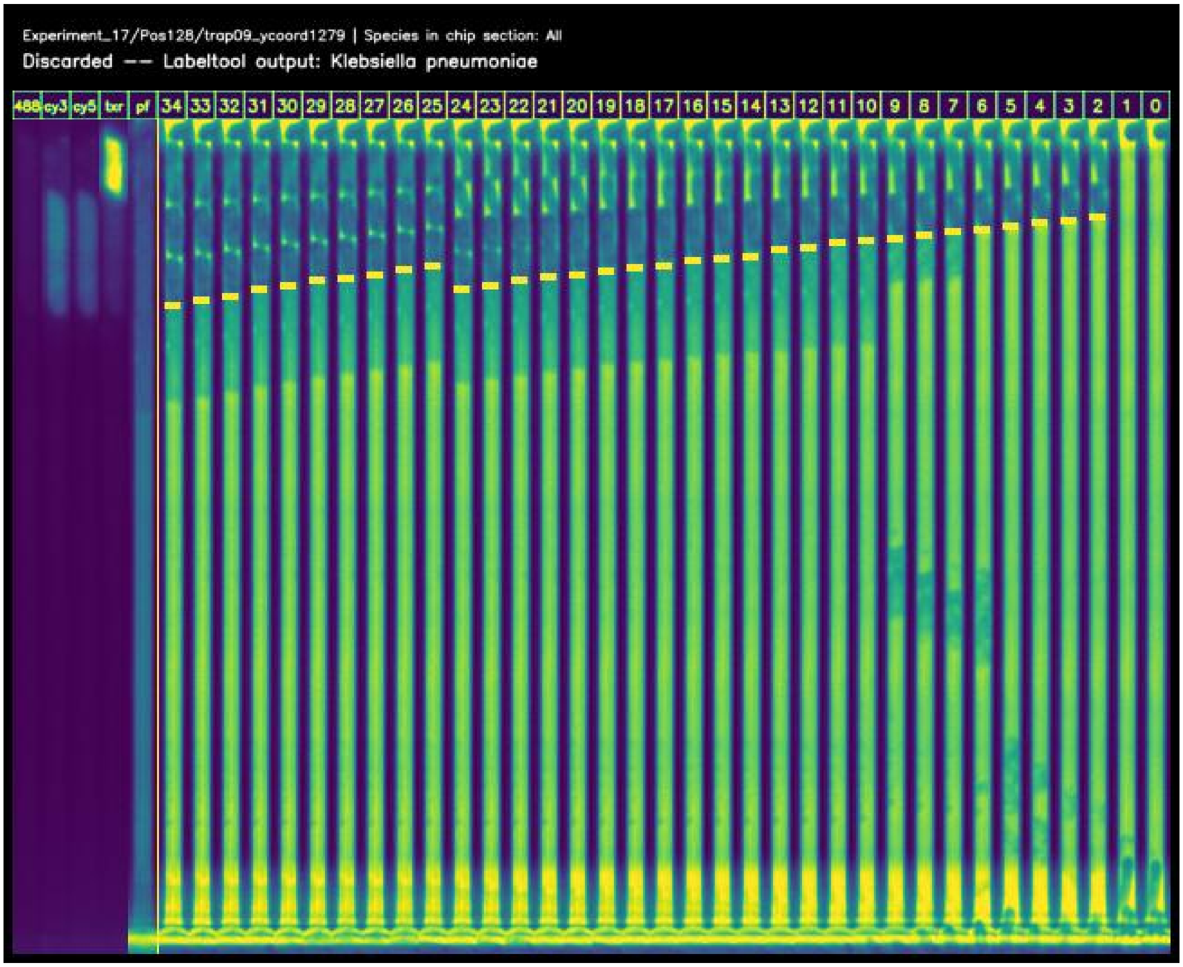
Experiment_17/Pos128/trap09_ycoord1279 | Species in chip section: All
Discarded -- Labeltool output: Klebsiella pneumoniae
488 cy3 cy5 txr pf 34 33 32 31 30 29 28 27 26 25 24 23 22 21 20 19 18 17 16 15 14 13 12 11 10 9 8 7 6 5 4 3 2 1 0

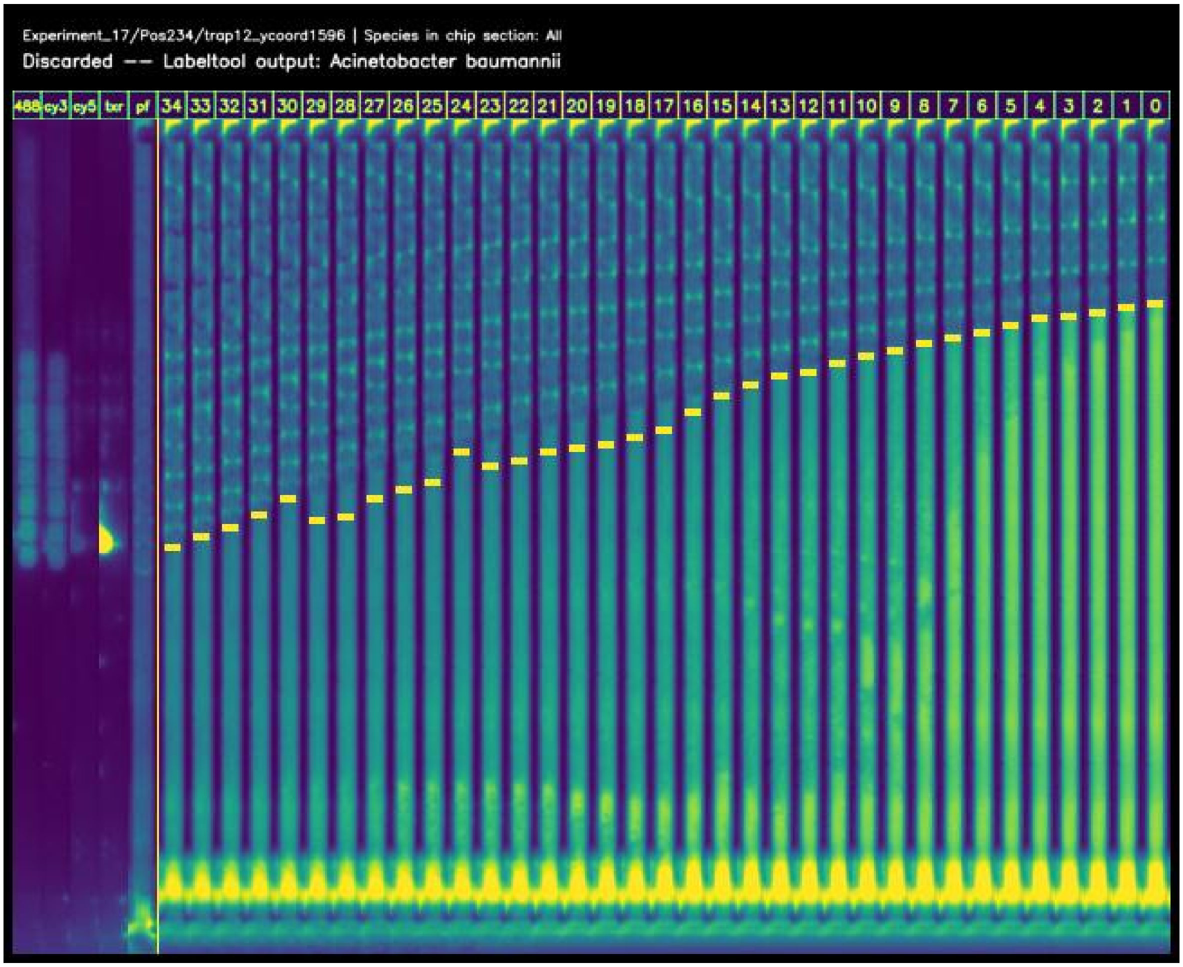
Experiment_17/Pos234/trap12_ycoord1596 | Species in chip section: All
Discarded -- Labeltool output: Acinetobacter baumannii
488 cy3 cy5 txr pf 34 33 32 31 30 29 28 27 26 25 24 23 22 21 20 19 18 17 16 15 14 13 12 11 10 9 8 7 6 5 4 3 2 1 0

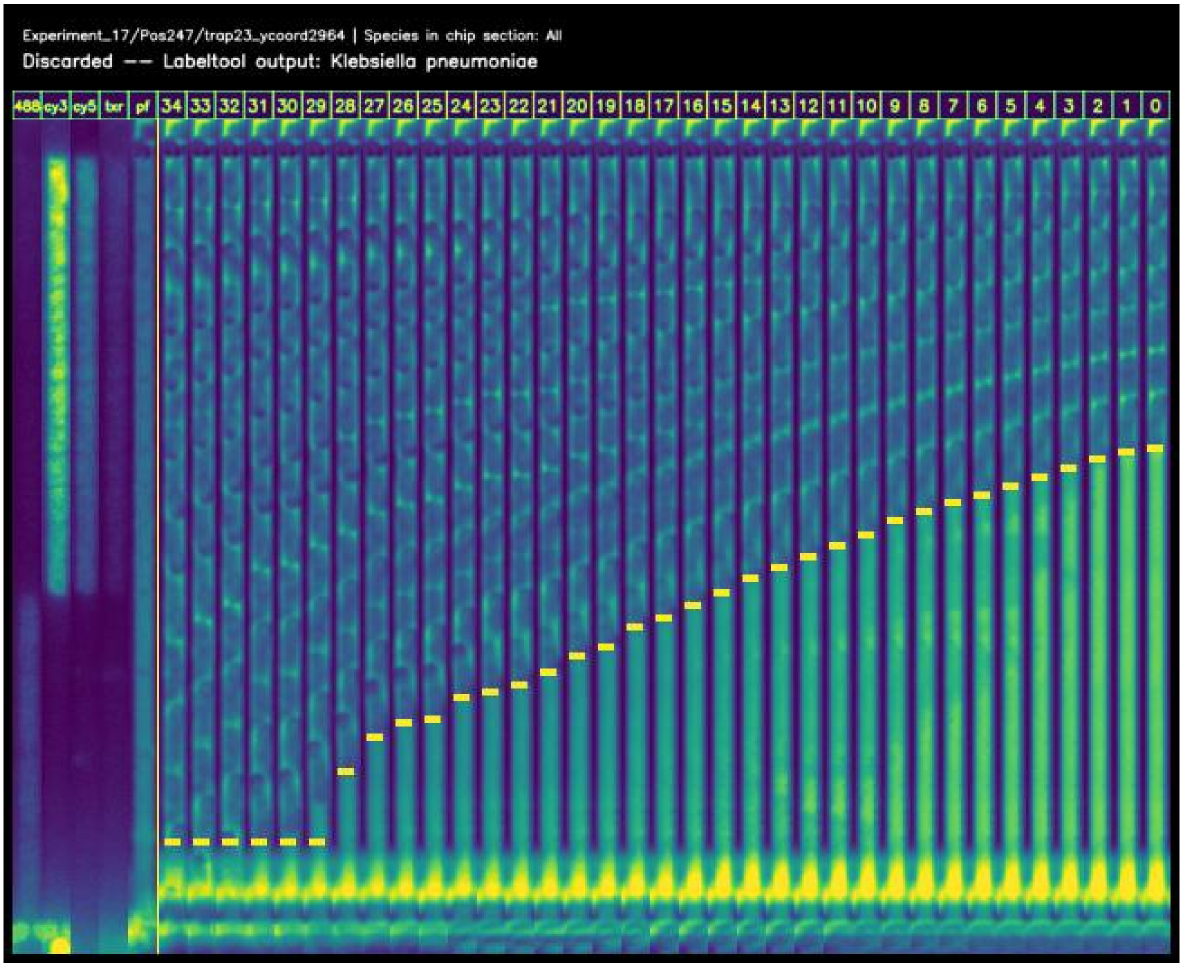
Experiment_17/Pos247/trap23_ycoord2964 | Species in chip section: All
Discarded -- Labeltool output: Klebsiella pneumoniae
488 cy3 cy5 txr pf 34 33 32 31 30 29 28 27 26 25 24 23 22 21 20 19 18 17 16 15 14 13 12 11 10 9 8 7 6 5 4 3 2 1 0

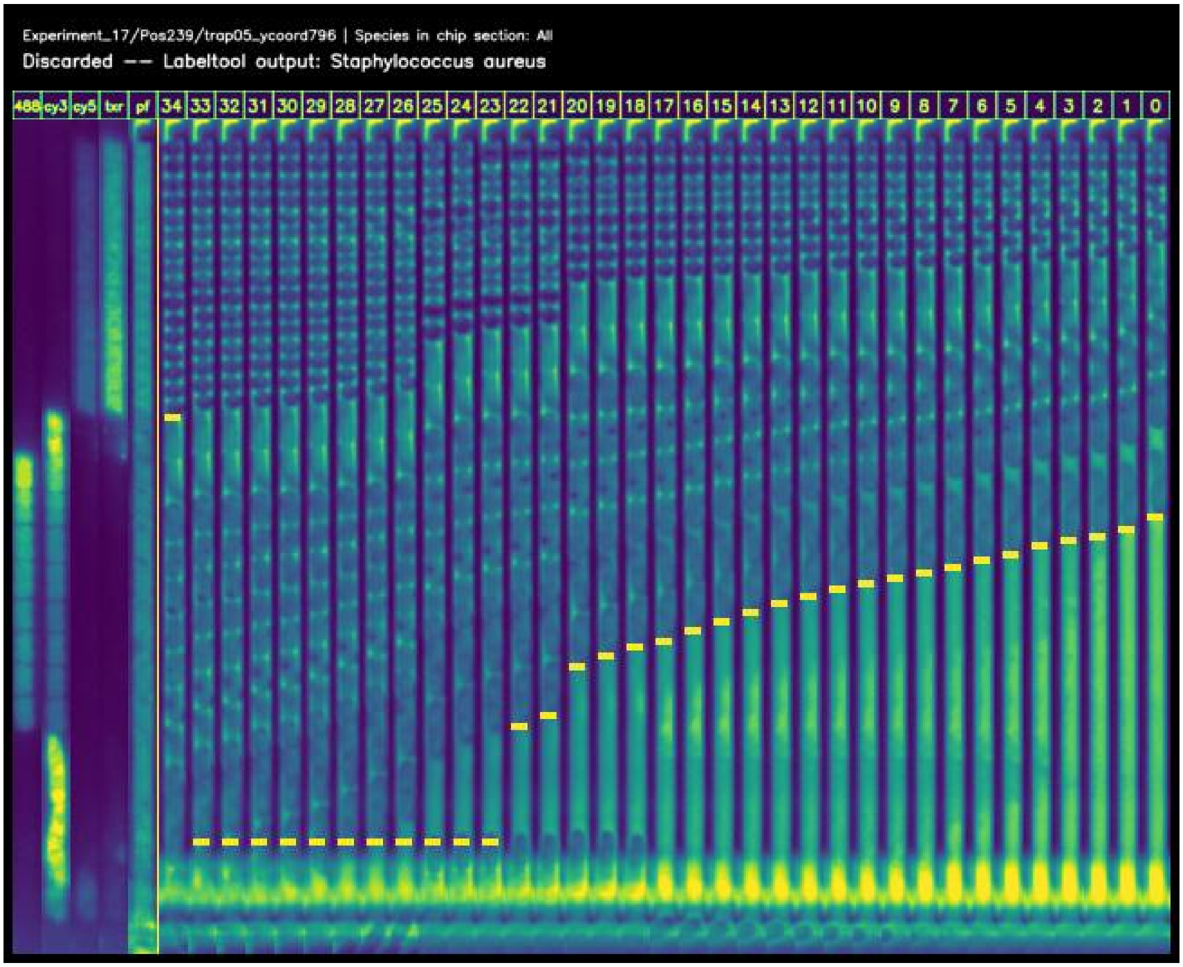
Experiment_17/Pos239/trap05_ycoord796 | Species in chip section: All
Discarded -- Labeltool output: Staphylococcus aureus
488 cy3 cy5 txr pf 34 33 32 31 30 29 28 27 26 25 24 23 22 21 20 19 18 17 16 15 14 13 12 11 10 9 8 7 6 5 4 3 2 1 0

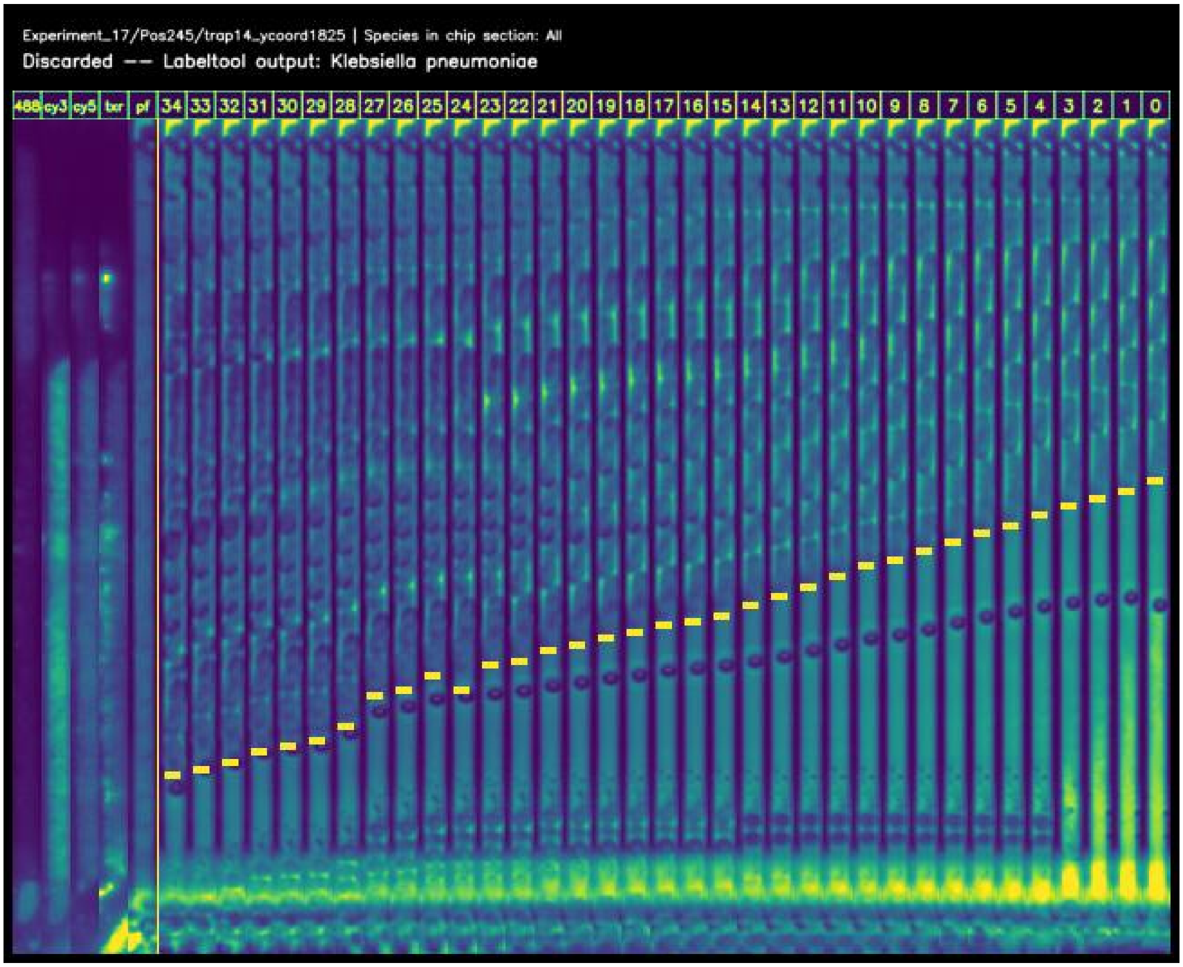
Experiment_17/Pos245/trap14_ycoord1825 | Species in chip section: All
Discarded -- Labeltool output: Klebsiella pneumoniae
488 cy3 cy5 txr pf 34 33 32 31 30 29 28 27 26 25 24 23 22 21 20 19 18 17 16 15 14 13 12 11 10 9 8 7 6 5 4 3 2 1 0

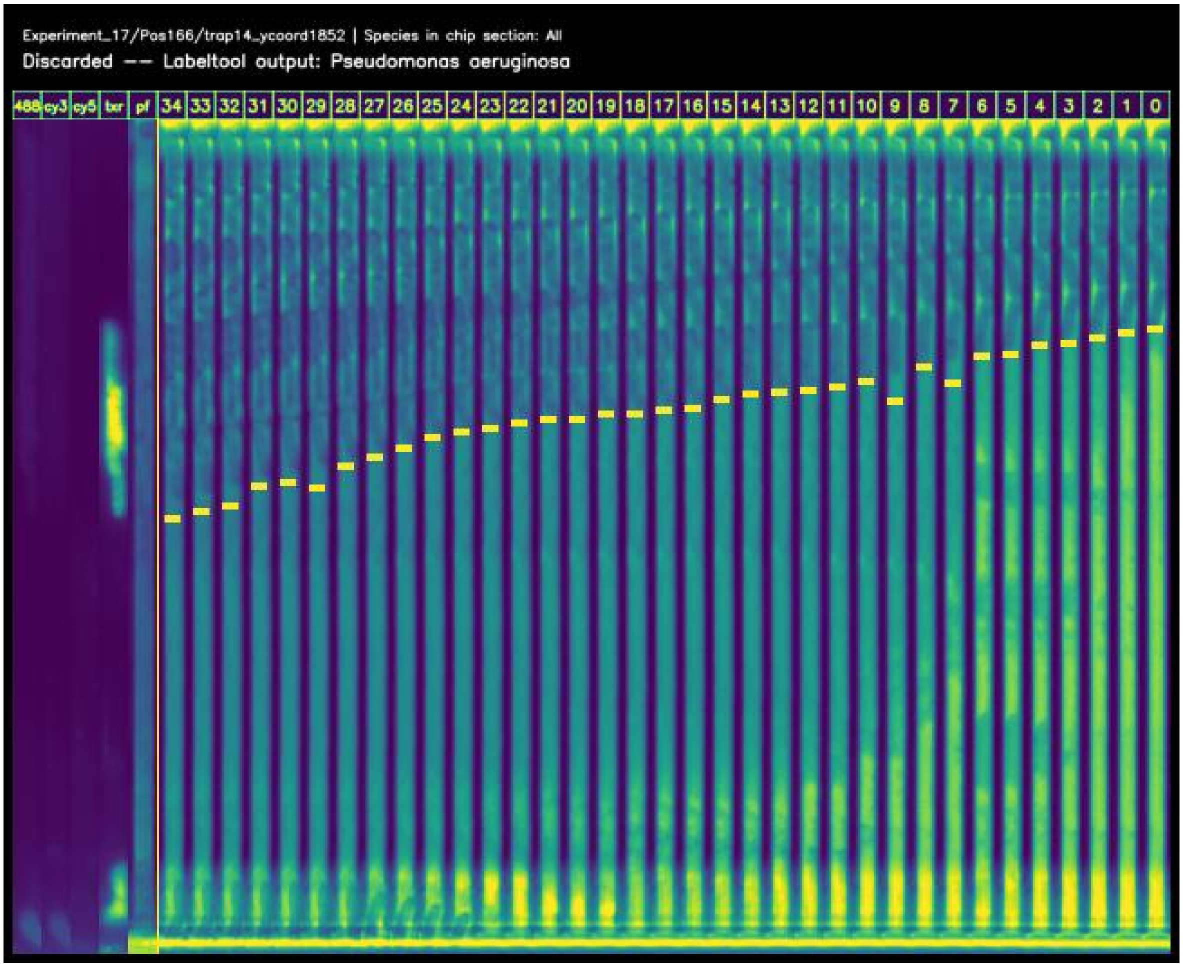
Experiment_17/Pos166/trap14_ycoord1852 | Species in chip section: All
Discarded -- Labeltool output: Pseudomonas aeruginosa
488 cy3 cy5 txr pf 34 33 32 31 30 29 28 27 26 25 24 23 22 21 20 19 18 17 16 15 14 13 12 11 10 9 8 7 6 5 4 3 2 1 0

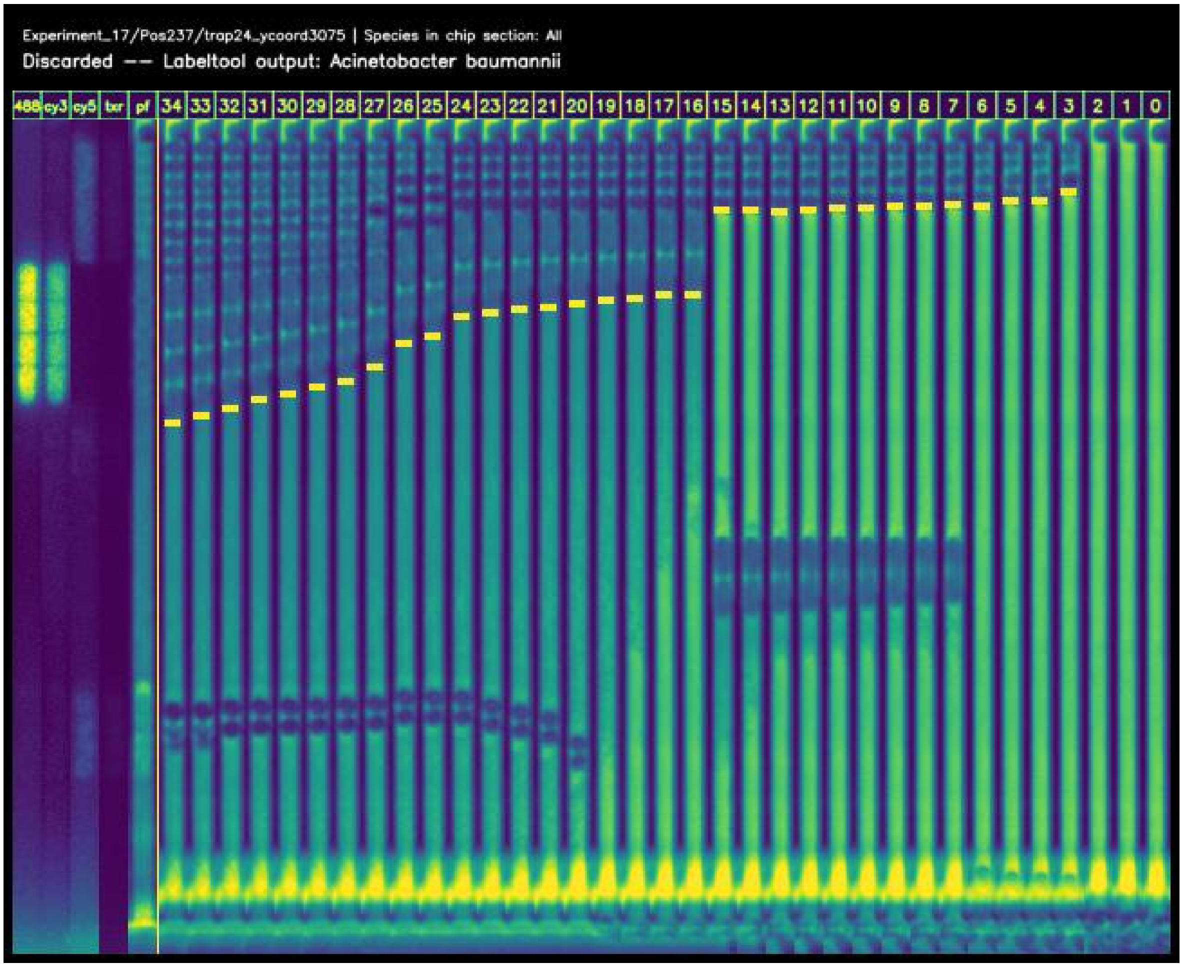
Experiment_17/Pos237/trap24_ycoord3075 | Species in chip section: All
Discarded -- Labeltool output: Acinetobacter baumannii
488 cy3 cy5 txr pf 34 33 32 31 30 29 28 27 26 25 24 23 22 21 20 19 18 17 16 15 14 13 12 11 10 9 8 7 6 5 4 3 2 1 0

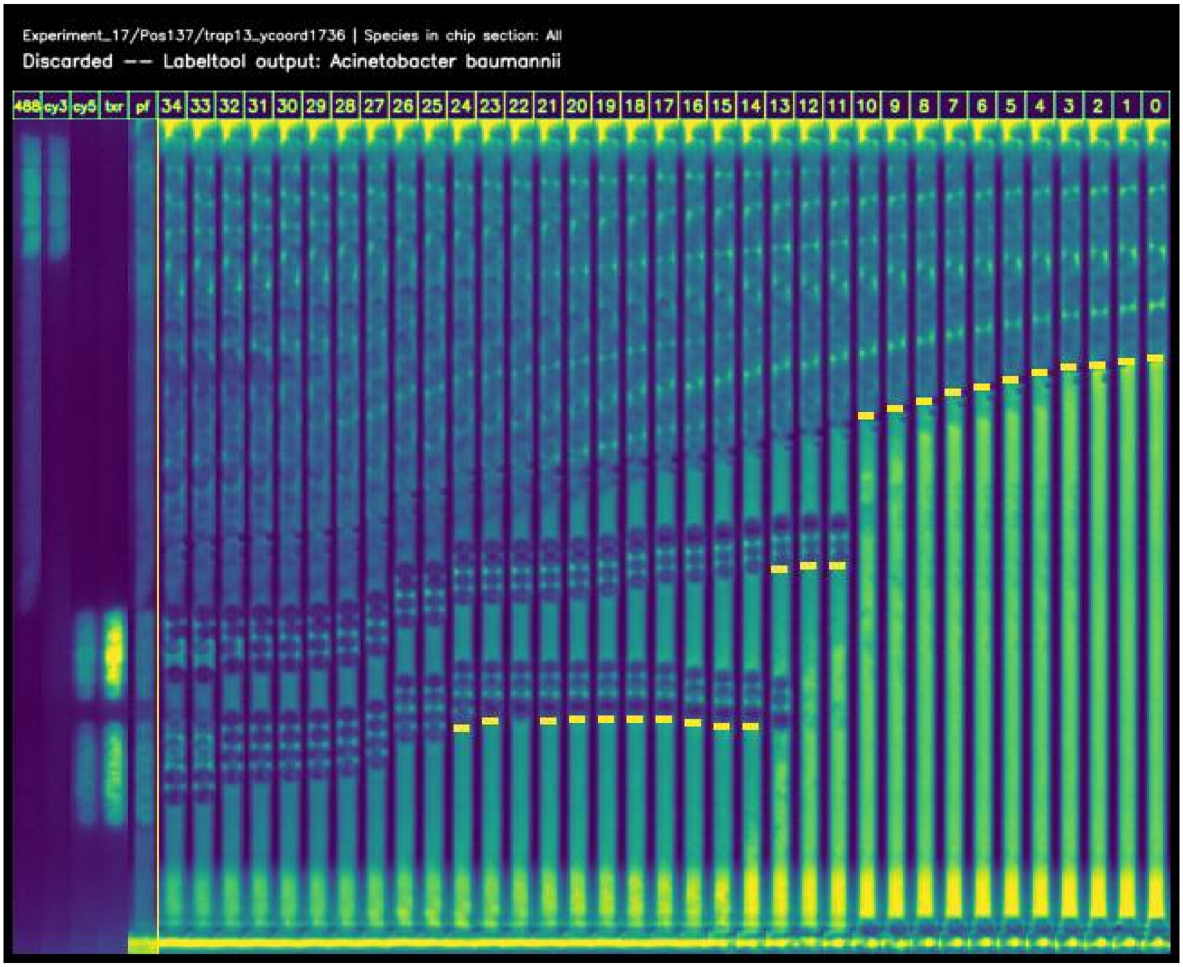
Experiment_17/Pos137/trap13_ycoord1736 | Species in chip section: All
Discarded -- Labeltool output: Acinetobacter baumannii
488 cy3 cy5 txr pf 34 33 32 31 30 29 28 27 26 25 24 23 22 21 20 19 18 17 16 15 14 13 12 11 10 9 8 7 6 5 4 3 2 1 0

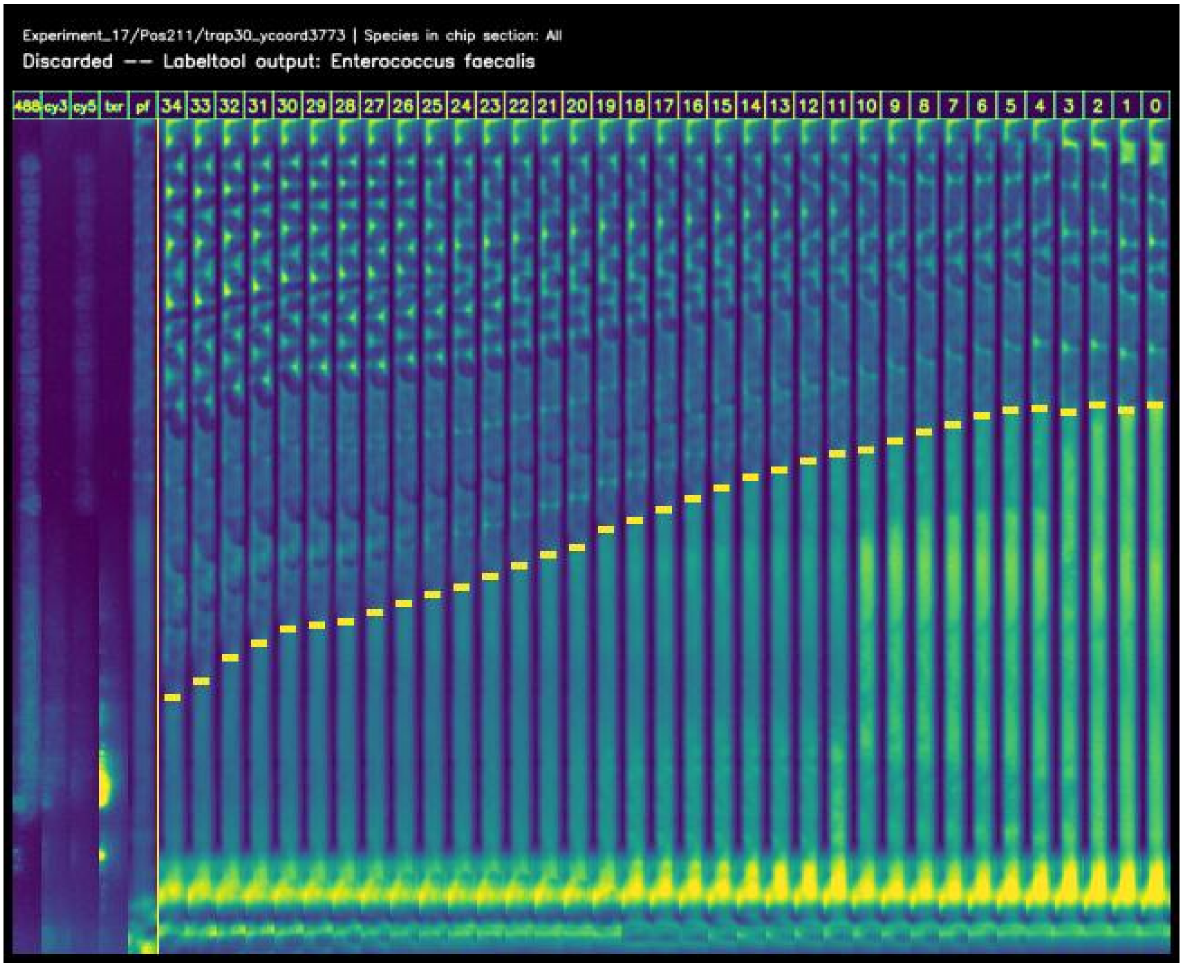
Experiment_17/Pos211/trap30_ycoord3773 | Species in chip section: All
Discarded -- Labeltool output: Enterococcus faecalis
488 cy3 cy5 txr pf 34 33 32 31 30 29 28 27 26 25 24 23 22 21 20 19 18 17 16 15 14 13 12 11 10 9 8 7 6 5 4 3 2 1 0

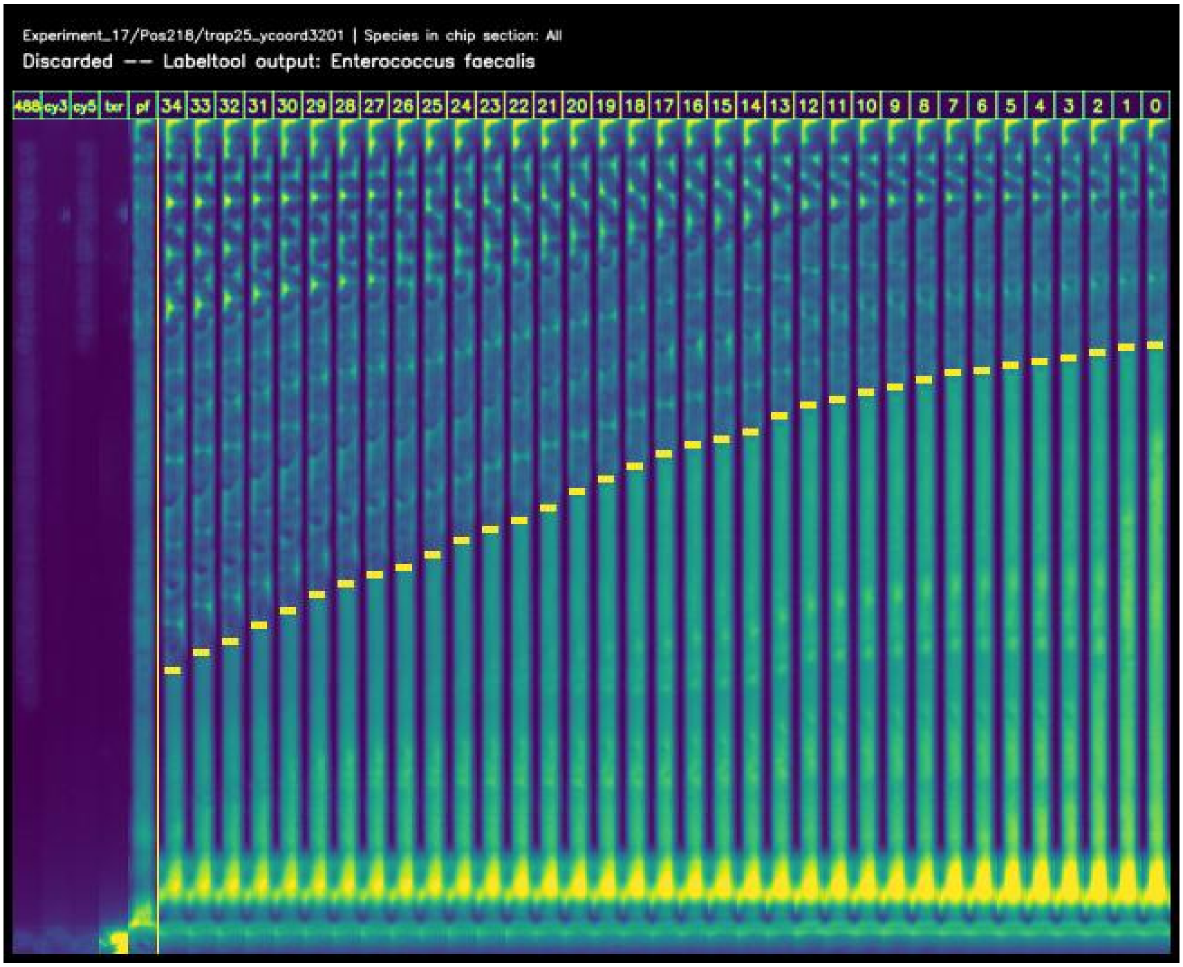
Experiment_17/Pos218/trap25_ycoord3201 | Species in chip section: All
Discarded -- Labeltool output: Enterococcus faecalis
488 cy3 cy5 txr pf 34 33 32 31 30 29 28 27 26 25 24 23 22 21 20 19 18 17 16 15 14 13 12 11 10 9 8 7 6 5 4 3 2 1 0

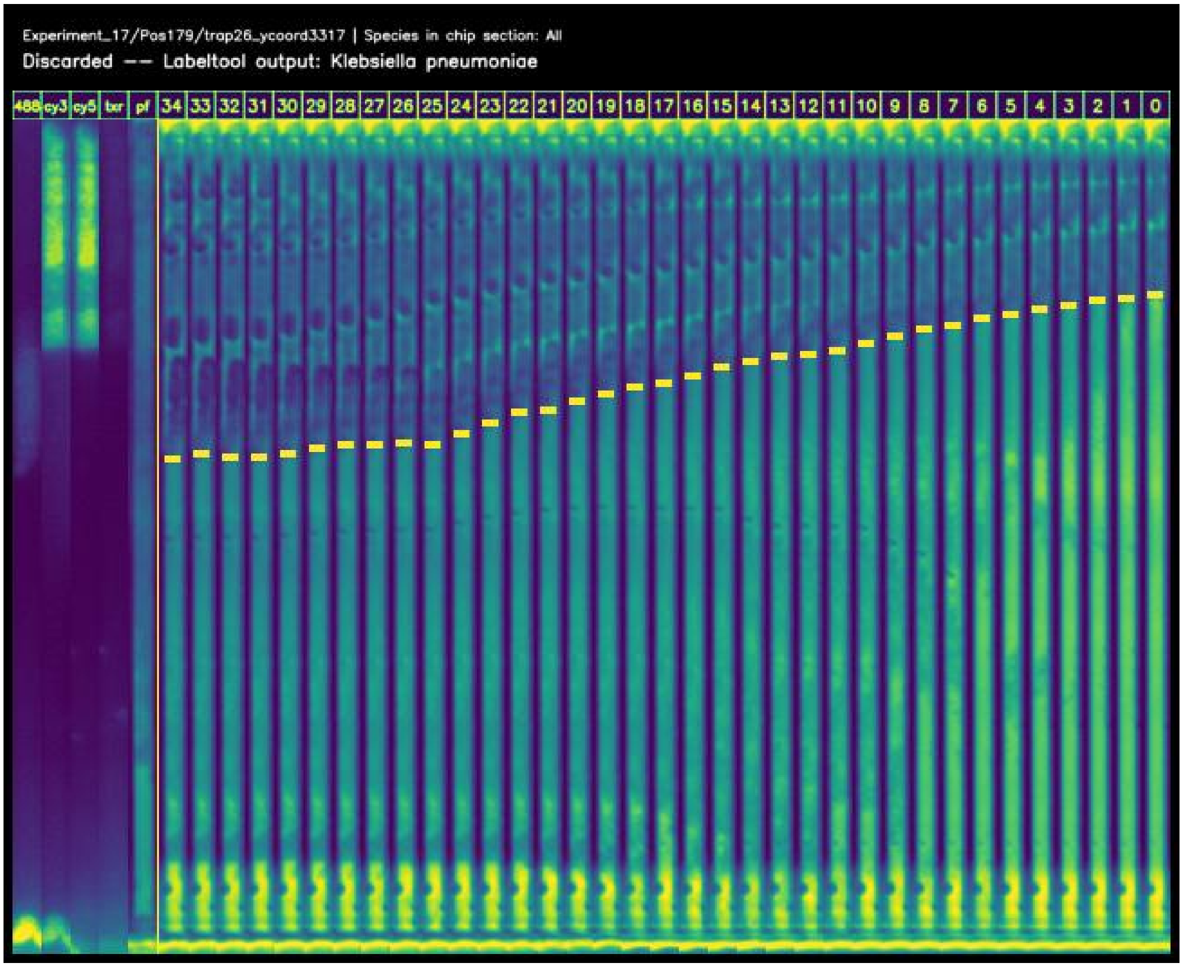
Experiment_17/Pos179/trap26_ycoord3317 | Species in chip section: All
Discarded -- Labeltool output: Klebsiella pneumoniae
488 cy3 cy5 txr pf 34 33 32 31 30 29 28 27 26 25 24 23 22 21 20 19 18 17 16 15 14 13 12 11 10 9 8 7 6 5 4 3 2 1 0

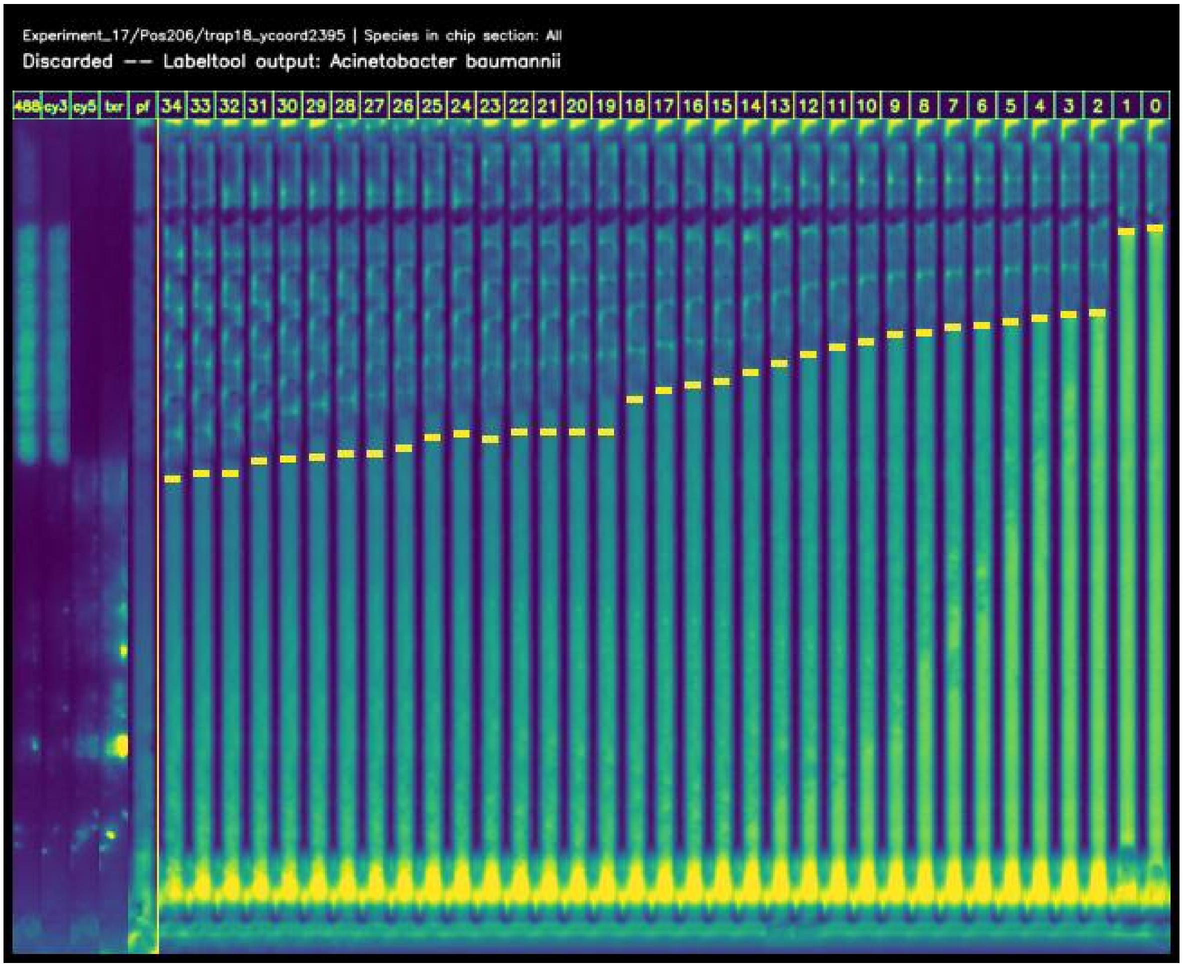
Experiment_17/Pos206/trap18_ycoord2395 | Species in chip section: All
Discarded -- Labeltool output: Acinetobacter baumannii
488 cy3 cy5 txr pf 34 33 32 31 30 29 28 27 26 25 24 23 22 21 20 19 18 17 16 15 14 13 12 11 10 9 8 7 6 5 4 3 2 1 0

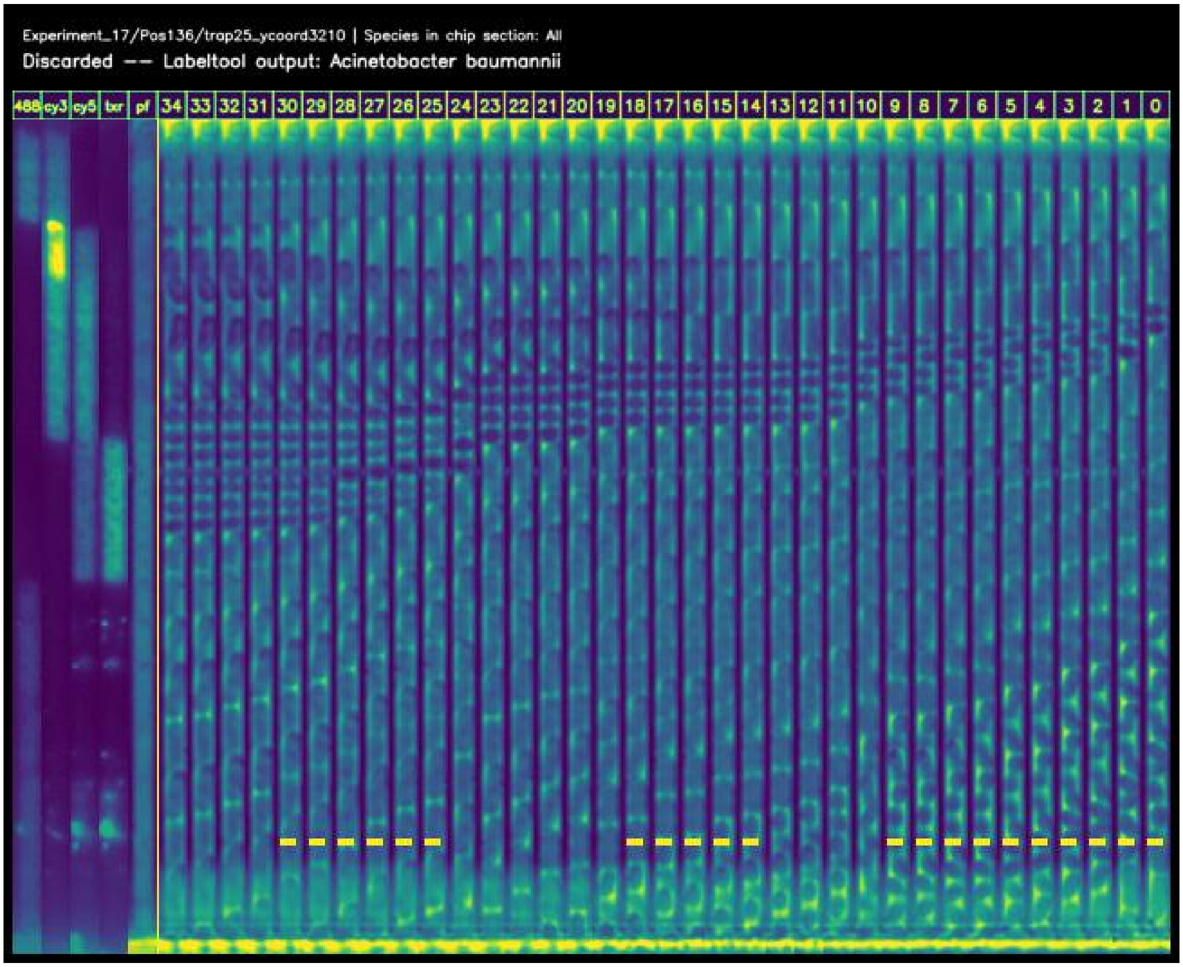
Experiment_17/Pos136/trap25_ycoord3210 | Species in chip section: All
Discarded -- Labeltool output: Acinetobacter baumannii
488 cy3 cy5 txr pf 34 33 32 31 30 29 28 27 26 25 24 23 22 21 20 19 18 17 16 15 14 13 12 11 10 9 8 7 6 5 4 3 2 1 0

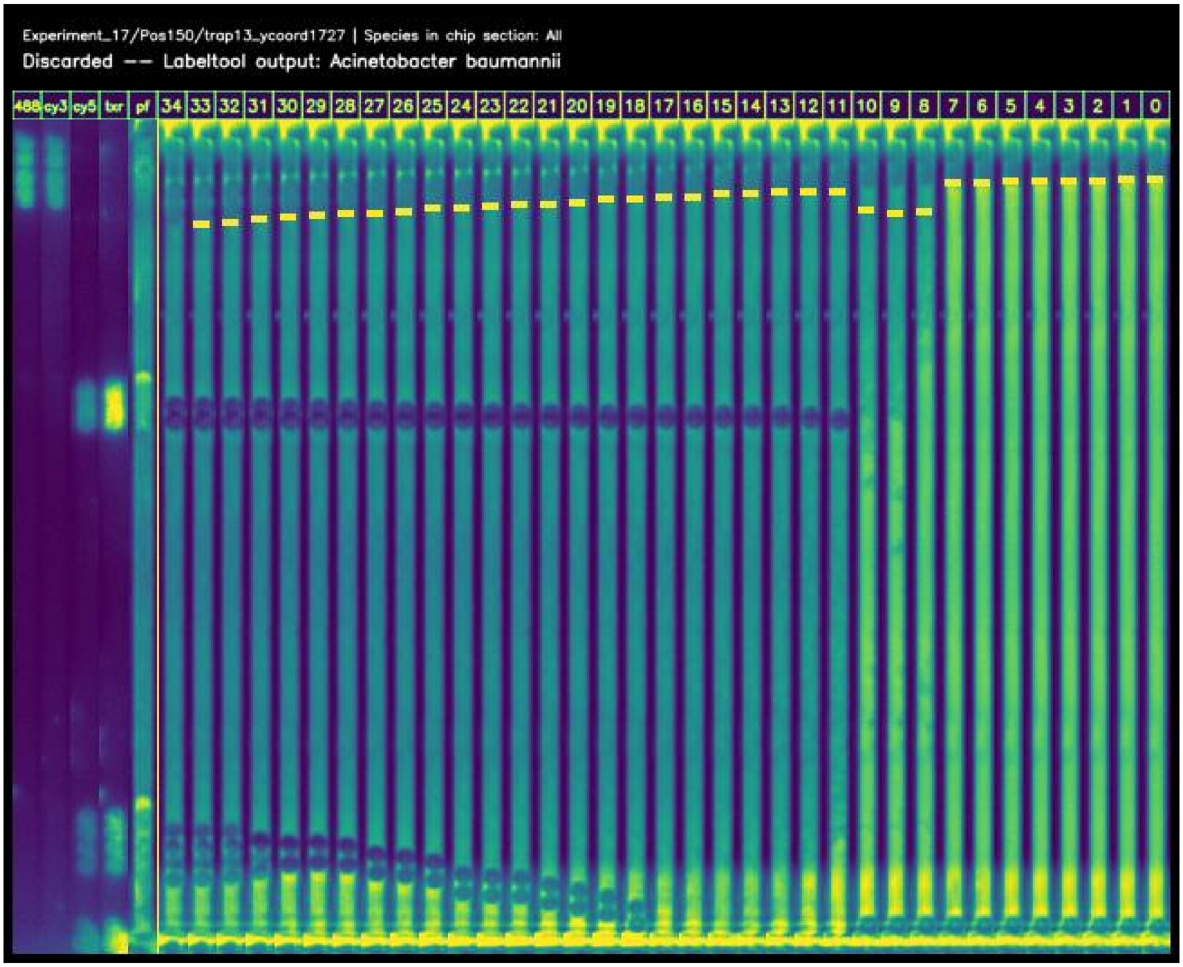
Experiment_17/Pos150/trap13_ycoord1727 | Species in chip section: All
Discarded -- Labeltool output: Acinetobacter baumannii
488 cy3 cy5 txr pf 34 33 32 31 30 29 28 27 26 25 24 23 22 21 20 19 18 17 16 15 14 13 12 11 10 9 8 7 6 5 4 3 2 1 0

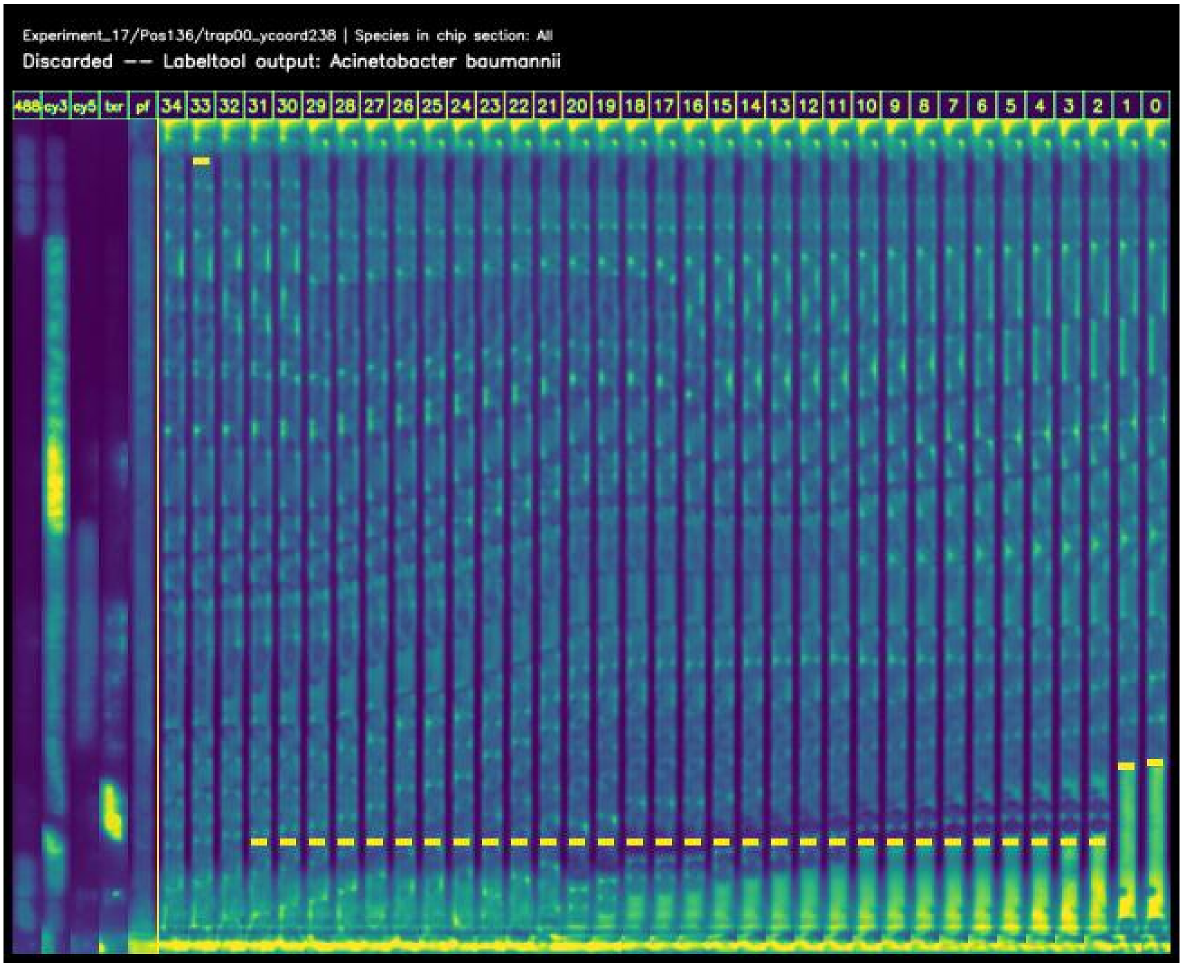
Experiment_17/Pos136/trap00_ycoord238 | Species in chip section: All
Discarded -- Labeltool output: Acinetobacter baumannii
488 cy3 cy5 txr pf 34 33 32 31 30 29 28 27 26 25 24 23 22 21 20 19 18 17 16 15 14 13 12 11 10 9 8 7 6 5 4 3 2 1 0

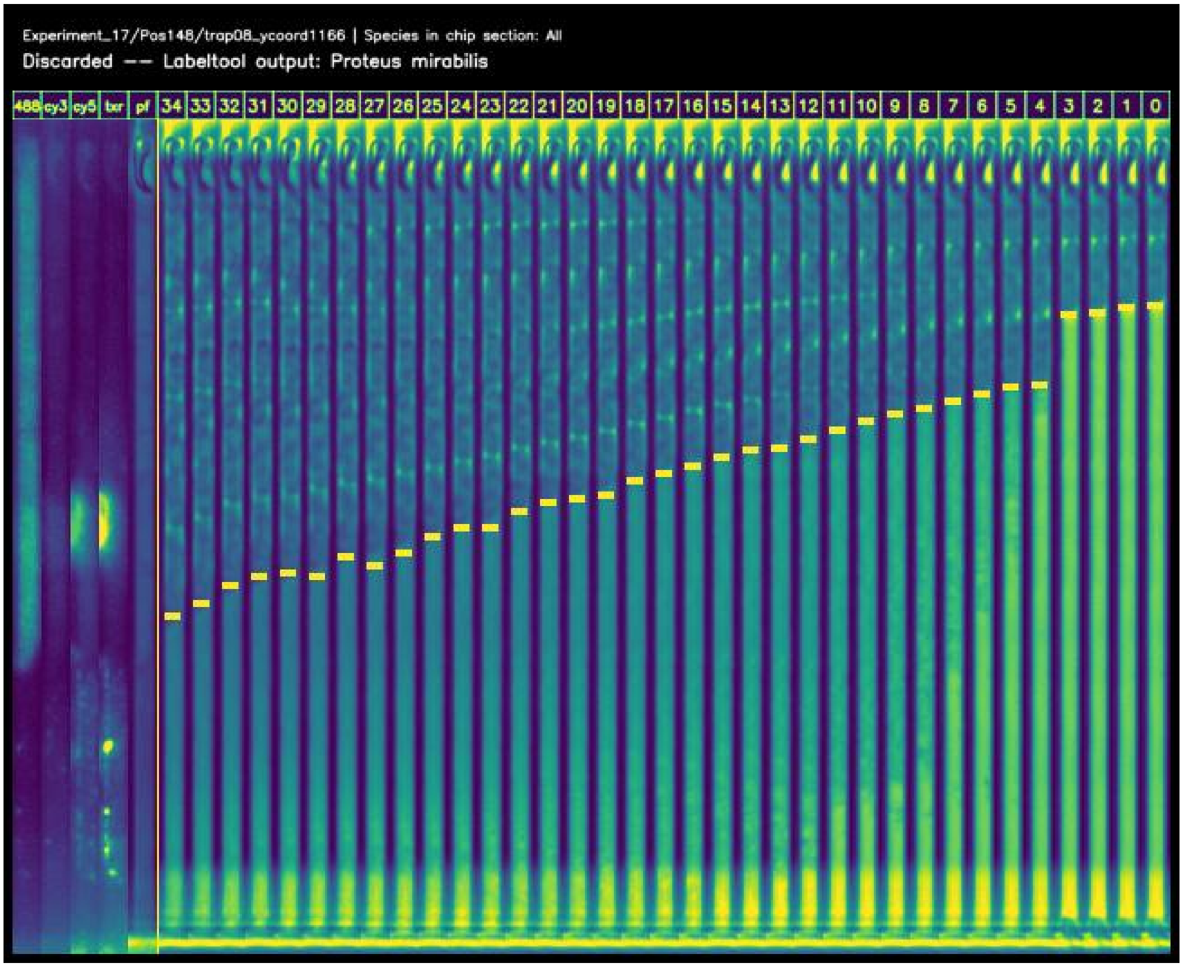
Experiment_17/Pos148/trap08_ycoord1166 | Species in chip section: All
Discarded -- Labeltool output: Proteus mirabilis
488 cy3 cy5 txr pf 34 33 32 31 30 29 28 27 26 25 24 23 22 21 20 19 18 17 16 15 14 13 12 11 10 9 8 7 6 5 4 3 2 1 0

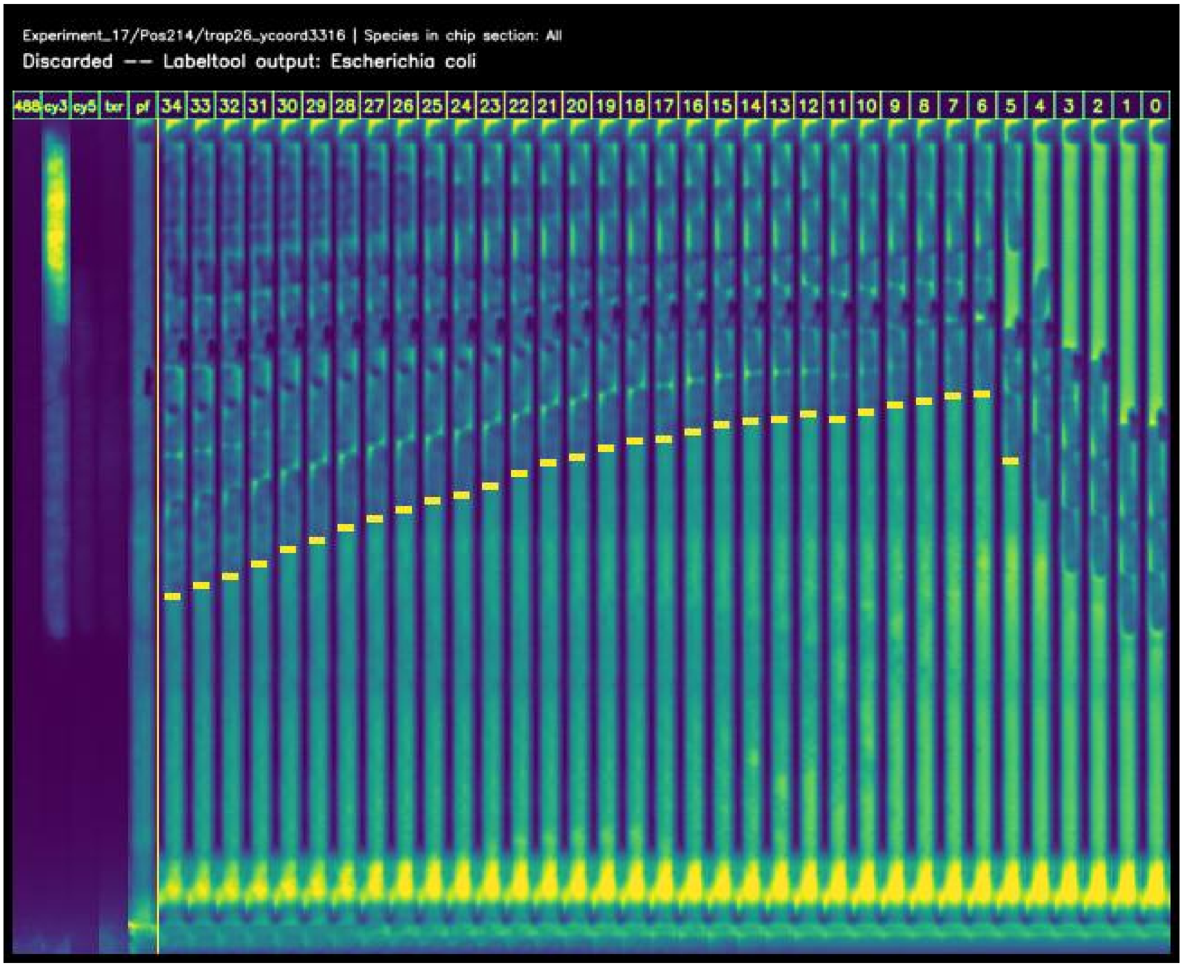
Experiment_17/Pos214/trap26_ycoord3316 | Species in chip section: All
Discarded -- Labeltool output: Escherichia coli
488 cy3 cy5 txr pf 34 33 32 31 30 29 28 27 26 25 24 23 22 21 20 19 18 17 16 15 14 13 12 11 10 9 8 7 6 5 4 3 2 1 0

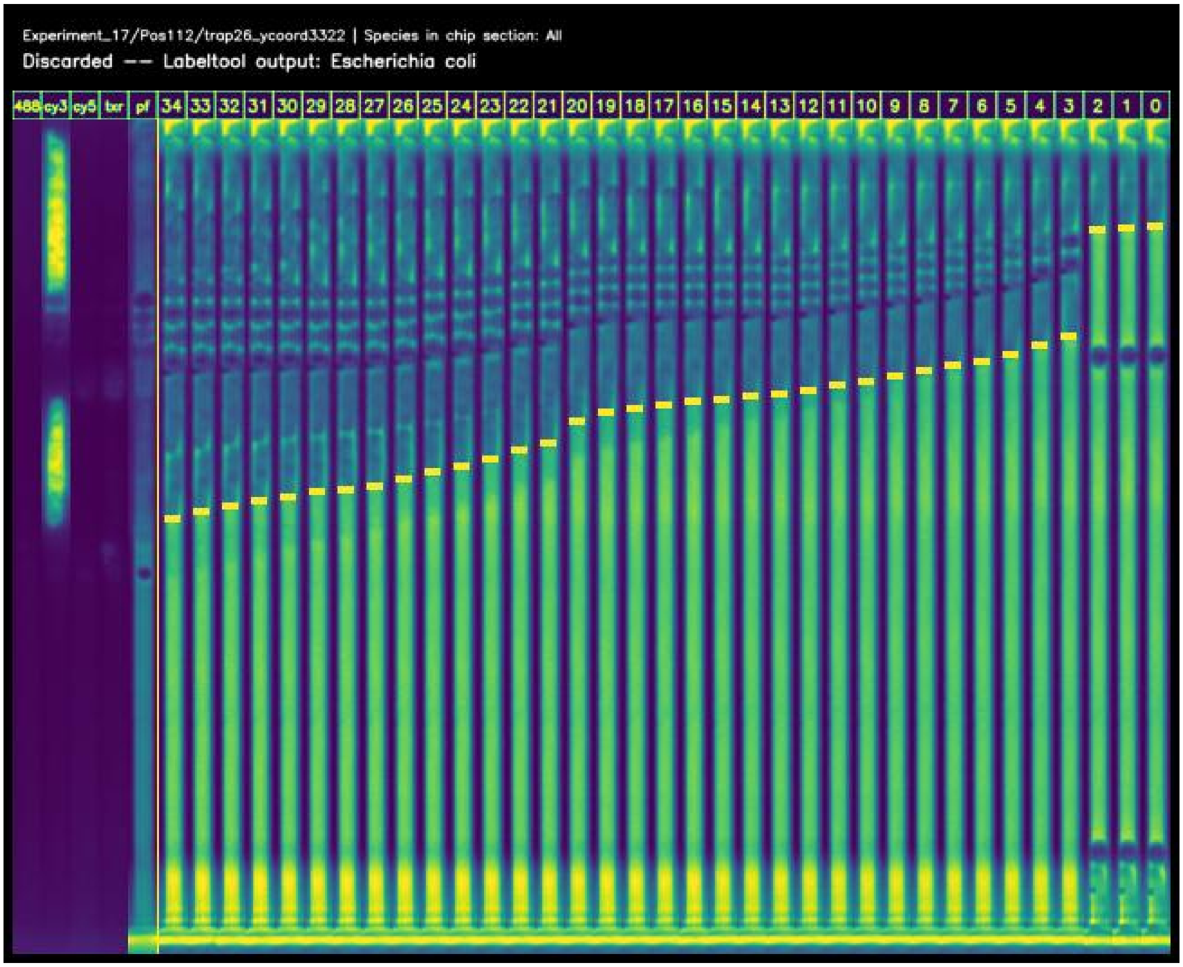
Experiment_17/Pos112/trap26_ycoord3322 | Species in chip section: All
Discarded -- Labeltool output: Escherichia coli
488 cy3 cy5 txr pf 34 33 32 31 30 29 28 27 26 25 24 23 22 21 20 19 18 17 16 15 14 13 12 11 10 9 8 7 6 5 4 3 2 1 0

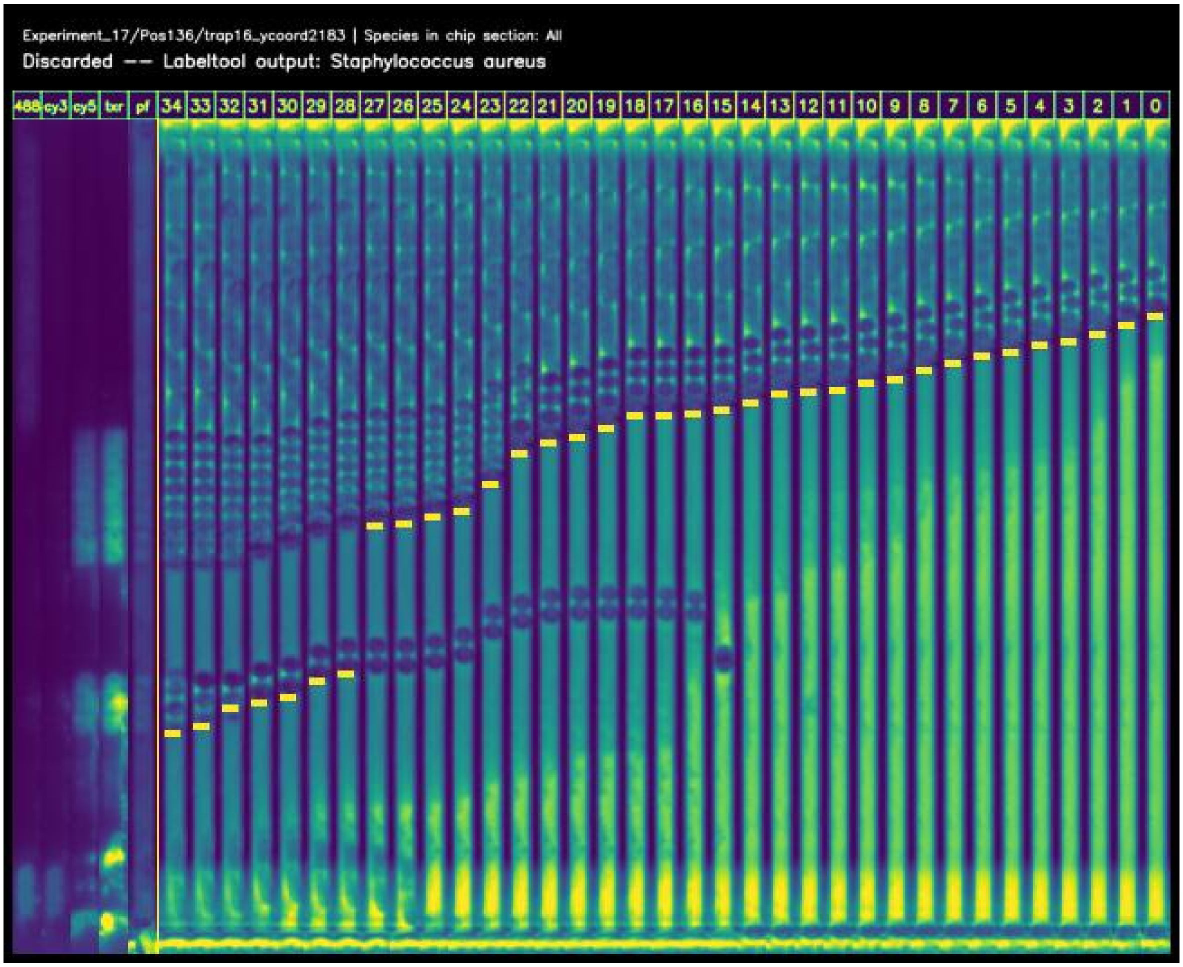
Experiment_17/Pos136/trap16_ycoord2183 | Species in chip section: All
Discarded -- Labeltool output: Staphylococcus aureus
488 cy3 cy5 txr pf 34 33 32 31 30 29 28 27 26 25 24 23 22 21 20 19 18 17 16 15 14 13 12 11 10 9 8 7 6 5 4 3 2 1 0

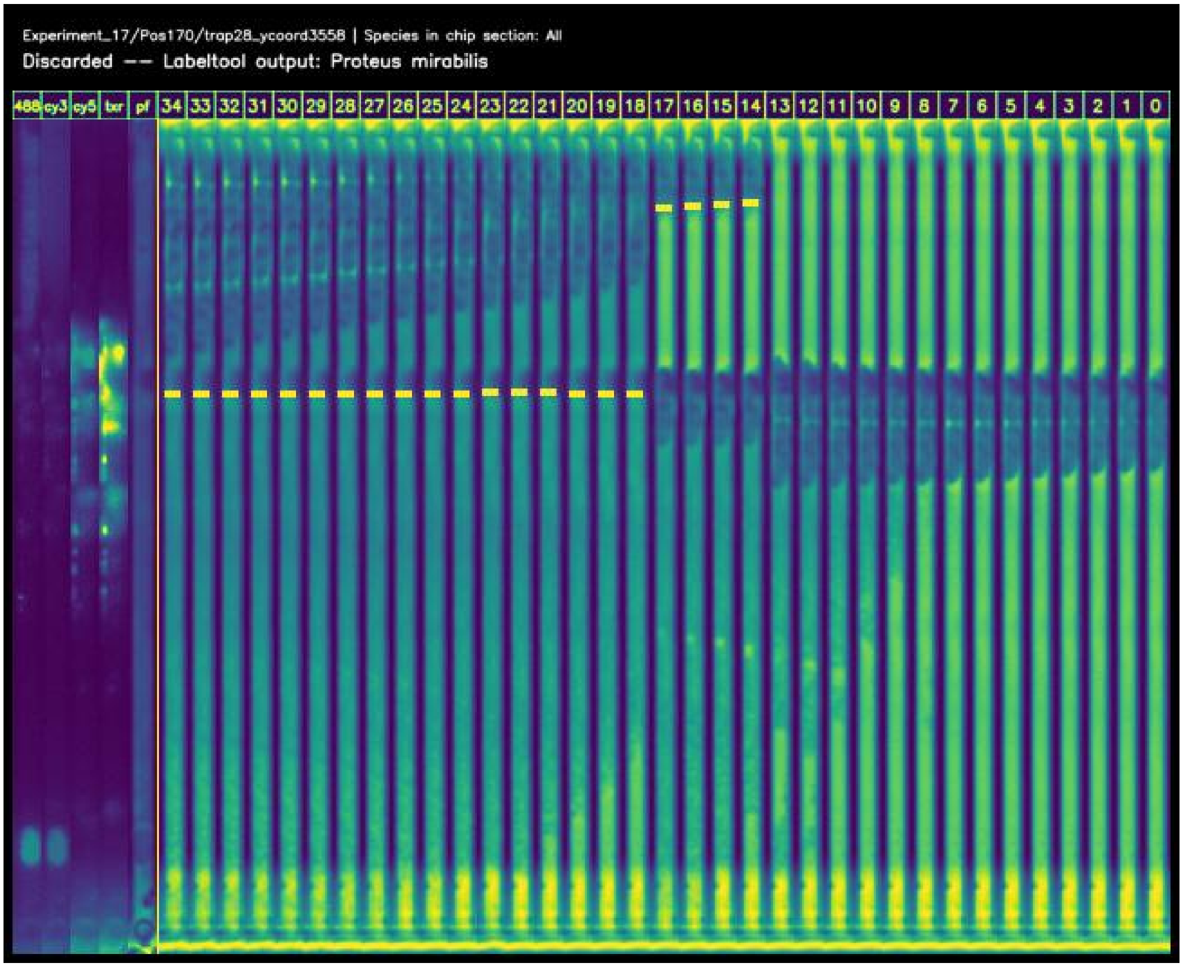
Experiment_17/Pos170/trap28_ycoord3558 | Species in chip section: All
Discarded -- Labeltool output: Proteus mirabilis
488 cy3 cy5 txr pf 34 33 32 31 30 29 28 27 26 25 24 23 22 21 20 19 18 17 16 15 14 13 12 11 10 9 8 7 6 5 4 3 2 1 0

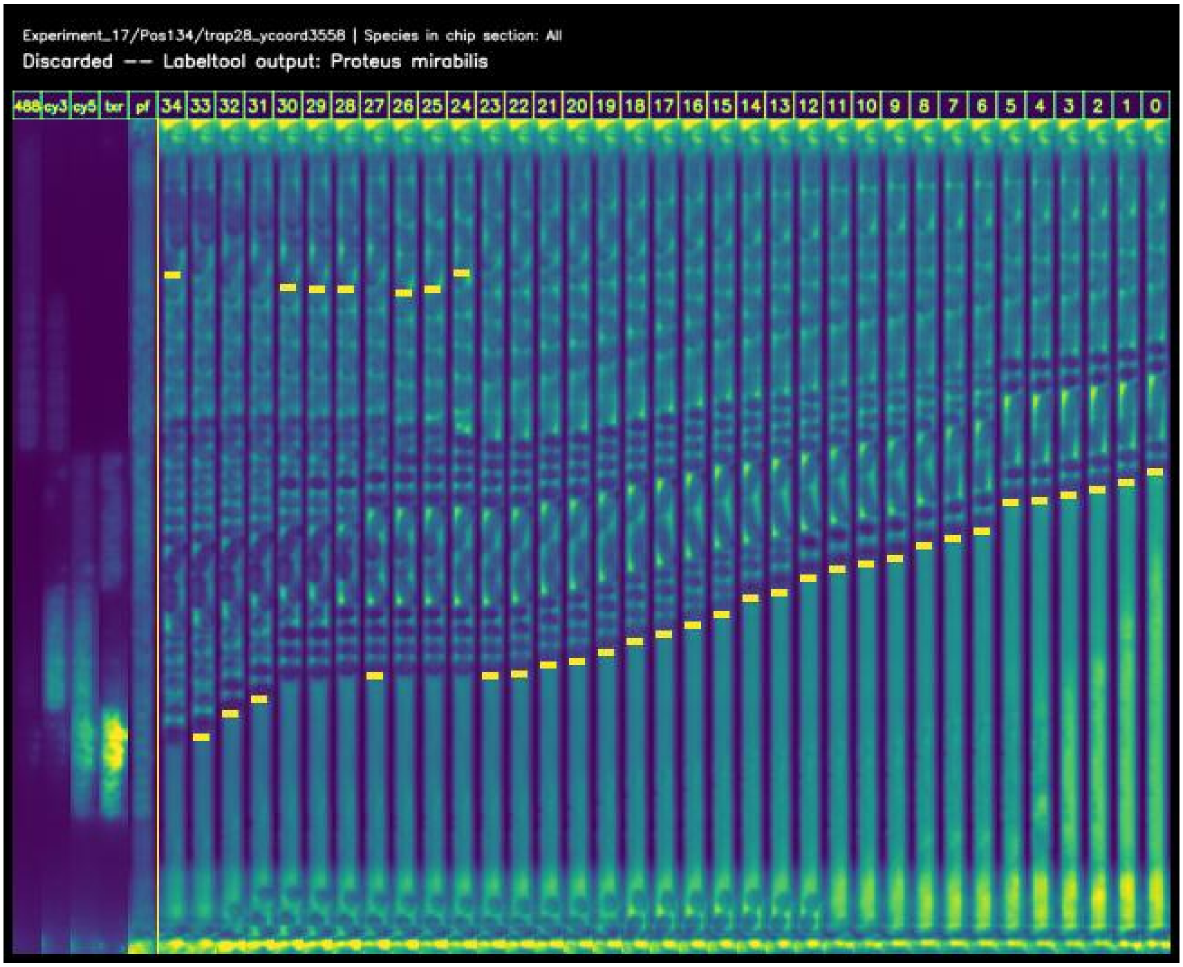
Experiment_17/Pos134/trap28_ycoord3558 | Species in chip section: All
Discarded -- Labeltool output: Proteus mirabilis
488 cy3 cy5 txr pf 34 33 32 31 30 29 28 27 26 25 24 23 22 21 20 19 18 17 16 15 14 13 12 11 10 9 8 7 6 5 4 3 2 1 0

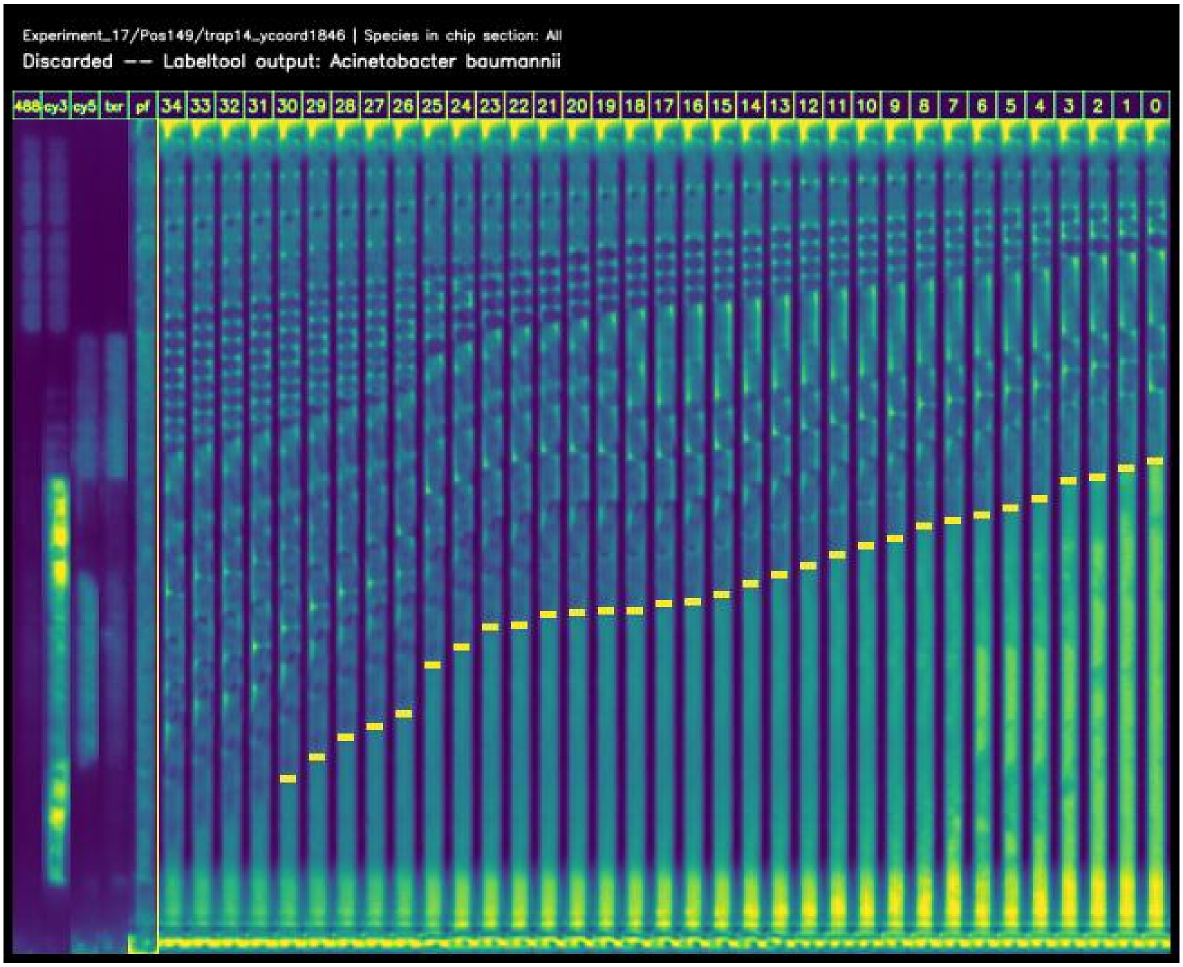
Experiment_17/Pos149/trap14_ycoord1846 | Species in chip section: All
Discarded -- Labeltool output: Acinetobacter baumannii
488 cy3 cy5 txr pf 34 33 32 31 30 29 28 27 26 25 24 23 22 21 20 19 18 17 16 15 14 13 12 11 10 9 8 7 6 5 4 3 2 1 0

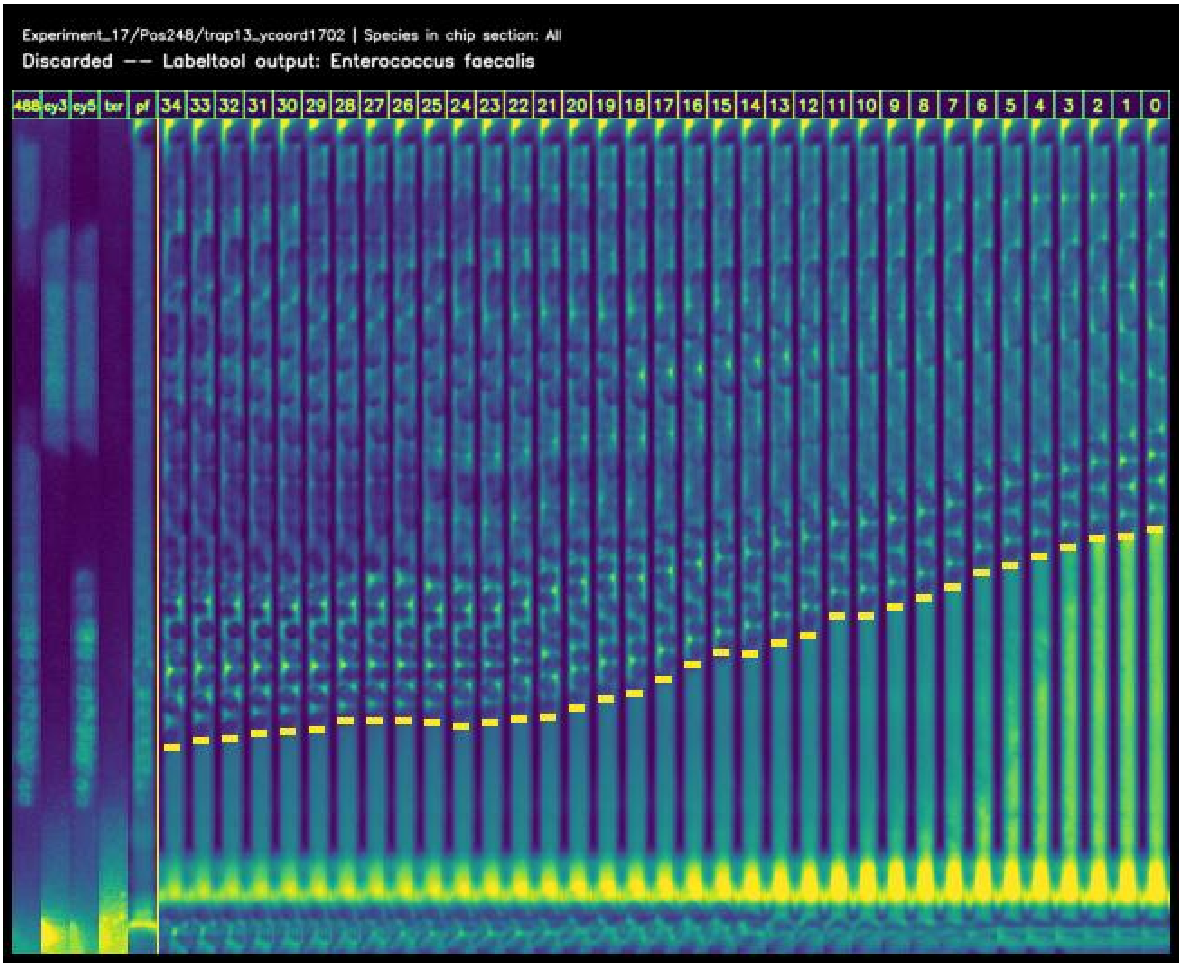
Experiment_17/Pos248/trap13_ycoord1702 | Species in chip section: All
Discarded -- Labeltool output: Enterococcus faecalis
488 cy3 cy5 txr pf 34 33 32 31 30 29 28 27 26 25 24 23 22 21 20 19 18 17 16 15 14 13 12 11 10 9 8 7 6 5 4 3 2 1 0

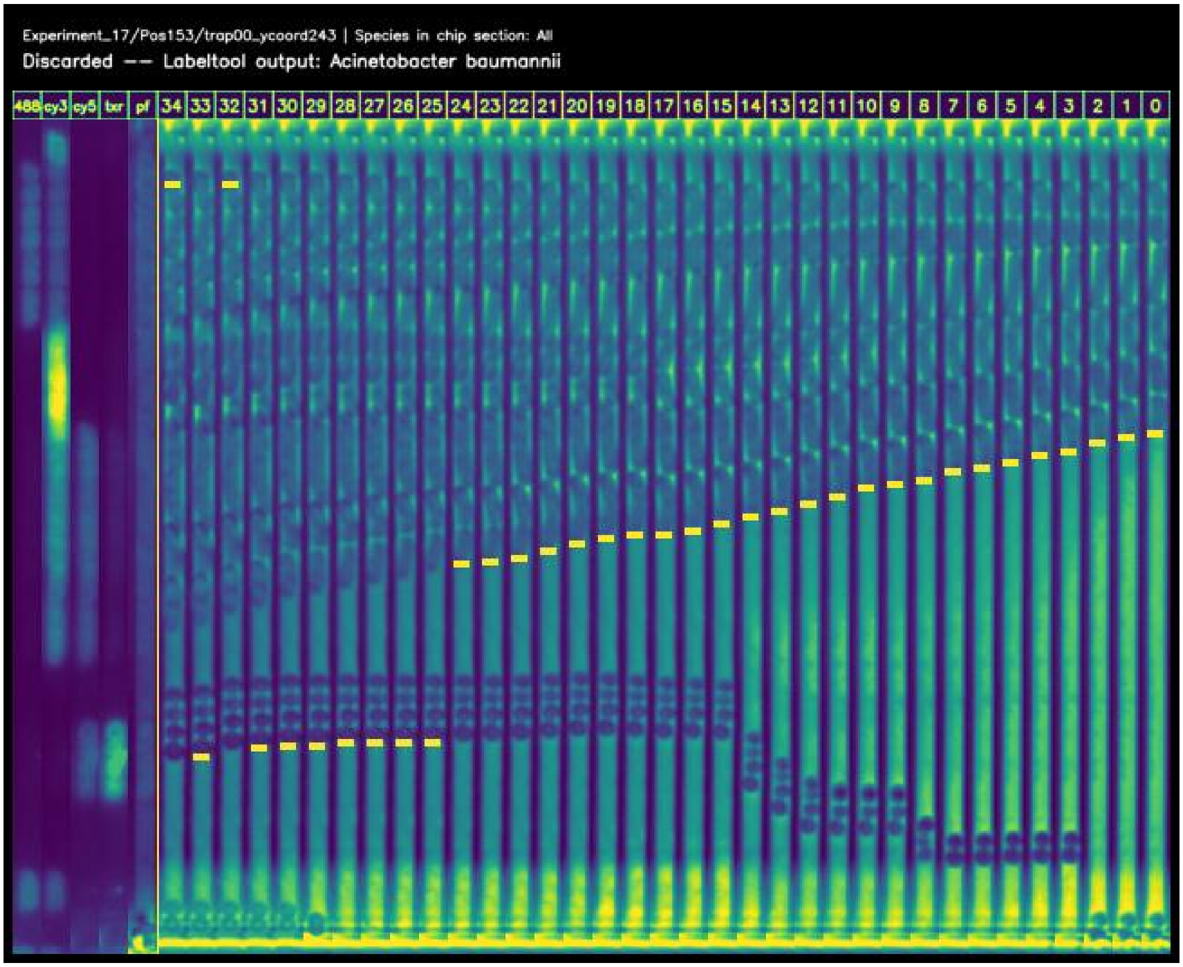
Experiment_17/Pos153/trap00_ycoord243 | Species in chip section: All
Discarded -- Labeltool output: Acinetobacter baumannii
488 cy3 cy5 txr pf 34 33 32 31 30 29 28 27 26 25 24 23 22 21 20 19 18 17 16 15 14 13 12 11 10 9 8 7 6 5 4 3 2 1 0

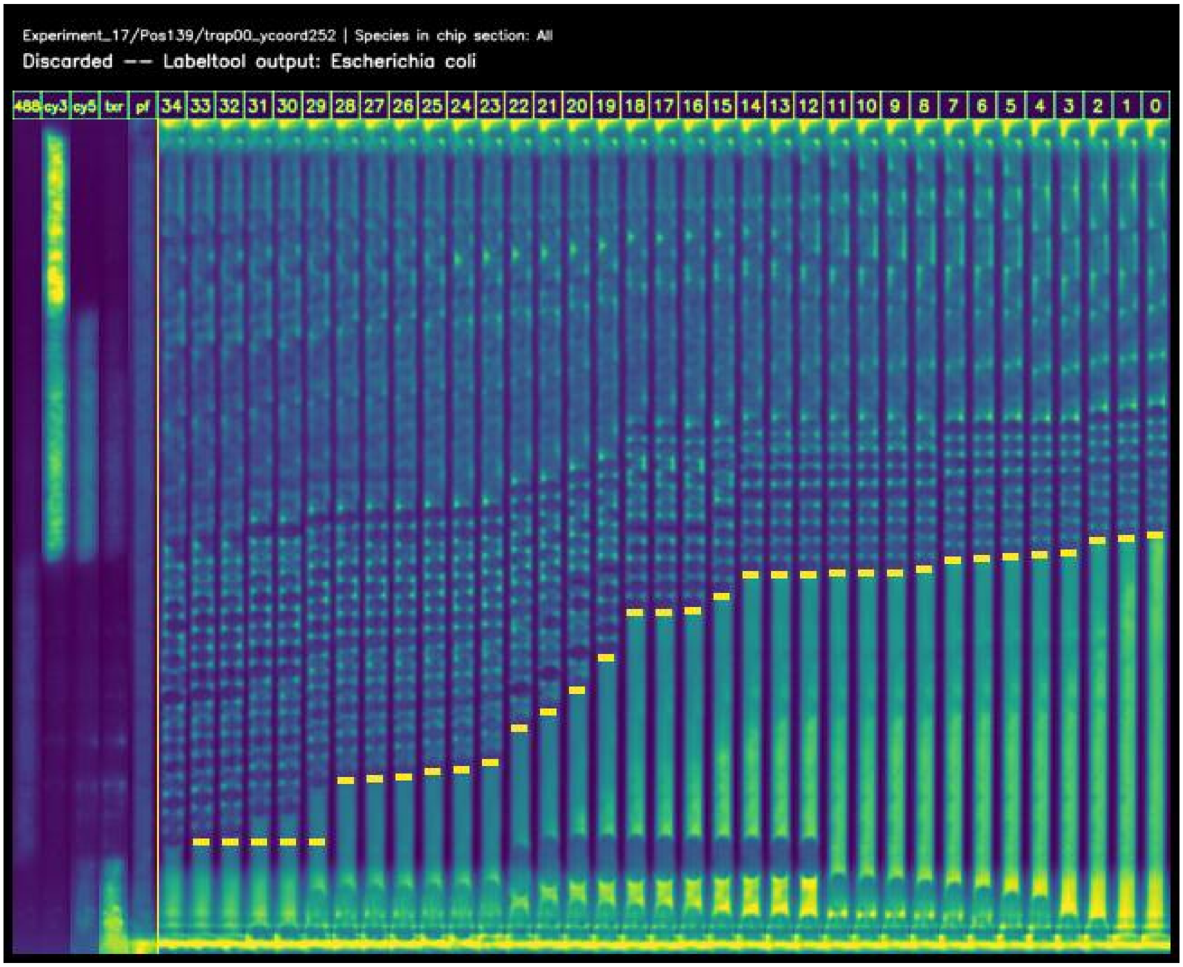
Experiment_17/Pos139/trap00_ycoord252 | Species in chip section: All
Discarded -- Labeltool output: Escherichia coli
488 cy3 cy5 txr pf 34 33 32 31 30 29 28 27 26 25 24 23 22 21 20 19 18 17 16 15 14 13 12 11 10 9 8 7 6 5 4 3 2 1 0

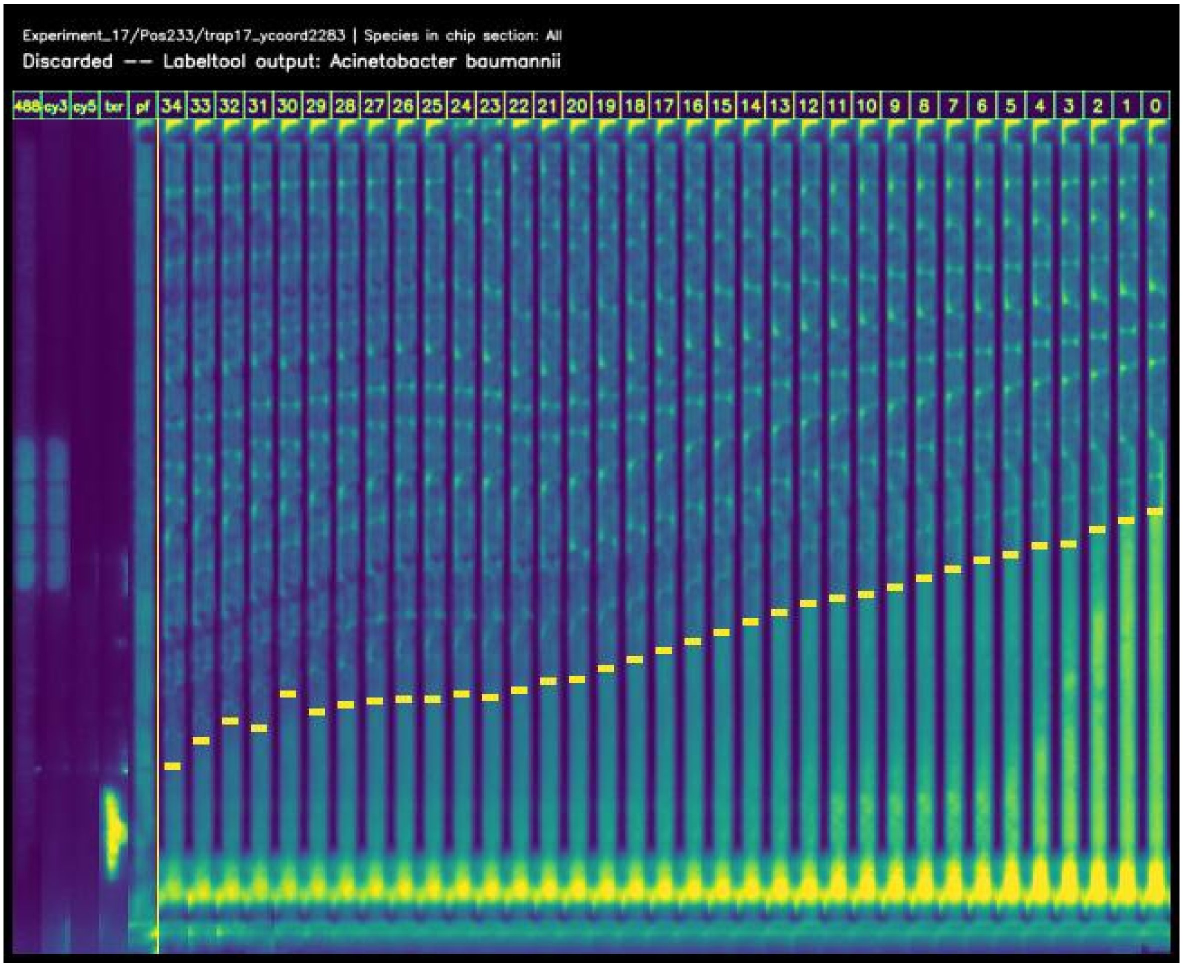
Experiment_17/Pos233/trap17_ycoord2283 | Species in chip section: All
Discarded -- Labeltool output: Acinetobacter baumannii
488 cy3 cy5 txr pf 34 33 32 31 30 29 28 27 26 25 24 23 22 21 20 19 18 17 16 15 14 13 12 11 10 9 8 7 6 5 4 3 2 1 0

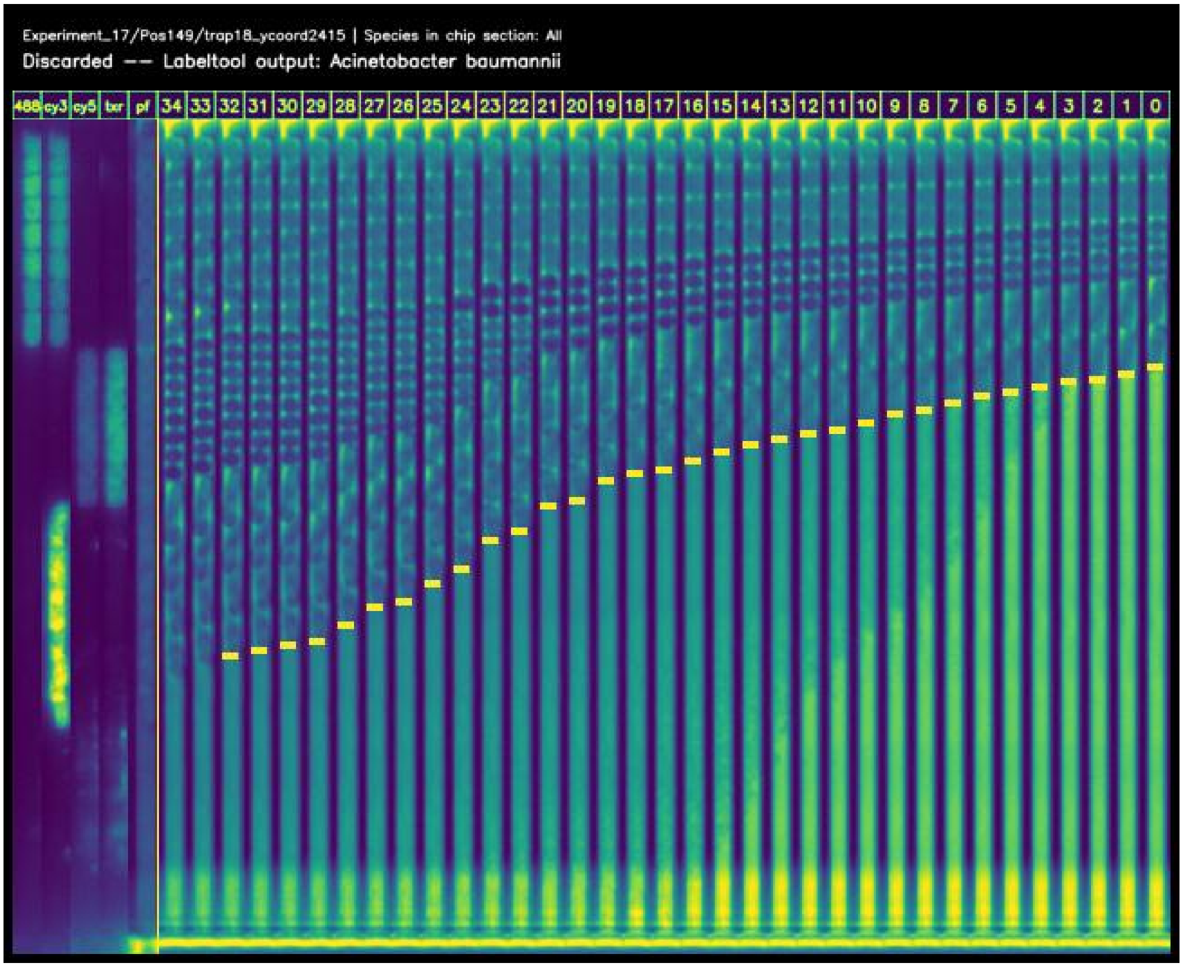
Experiment_17/Pos149/trap18_ycoord2415 | Species in chip section: All
Discarded -- Labeltool output: Acinetobacter baumannii
488 cy3 cy5 txr pf 34 33 32 31 30 29 28 27 26 25 24 23 22 21 20 19 18 17 16 15 14 13 12 11 10 9 8 7 6 5 4 3 2 1 0

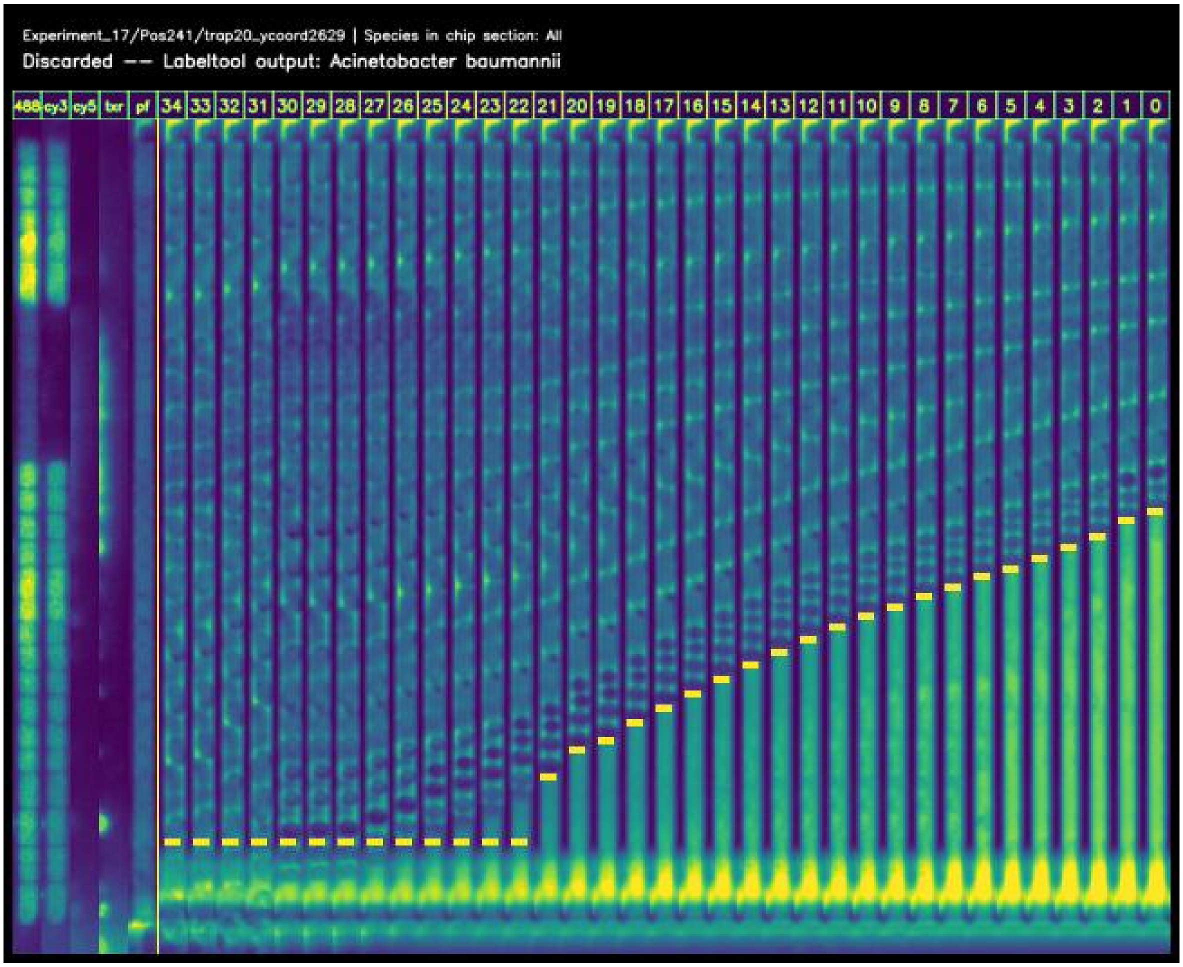
Experiment_17/Pos241/trap20_ycoord2629 | Species in chip section: All
Discarded -- Labeltool output: Acinetobacter baumannii
488 cy3 cy5 txr pf 34 33 32 31 30 29 28 27 26 25 24 23 22 21 20 19 18 17 16 15 14 13 12 11 10 9 8 7 6 5 4 3 2 1 0

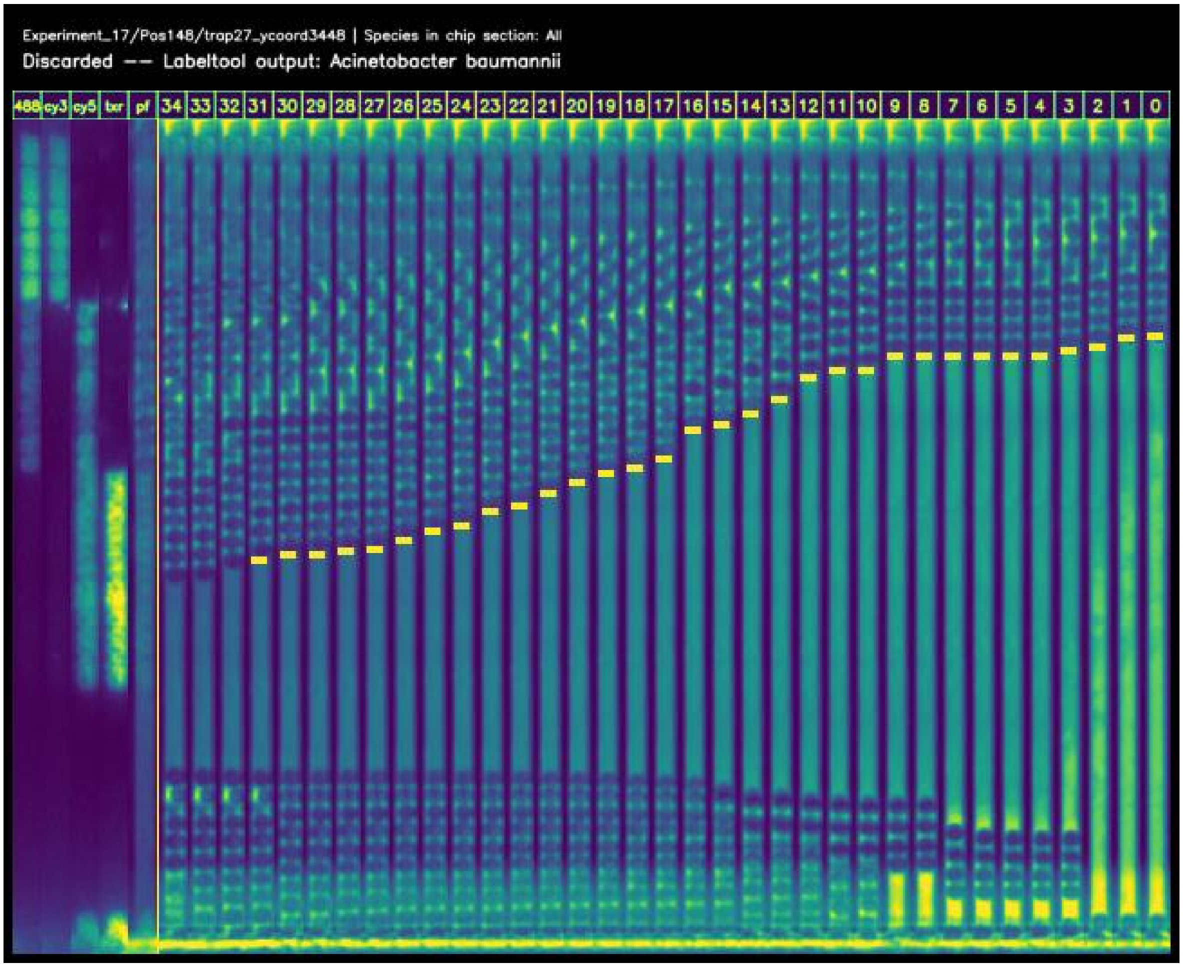
Experiment_17/Pos148/trap27_ycoord3448 | Species in chip section: All
Discarded -- Labeltool output: Acinetobacter baumannii
488 cy3 cy5 txr pf 34 33 32 31 30 29 28 27 26 25 24 23 22 21 20 19 18 17 16 15 14 13 12 11 10 9 8 7 6 5 4 3 2 1 0

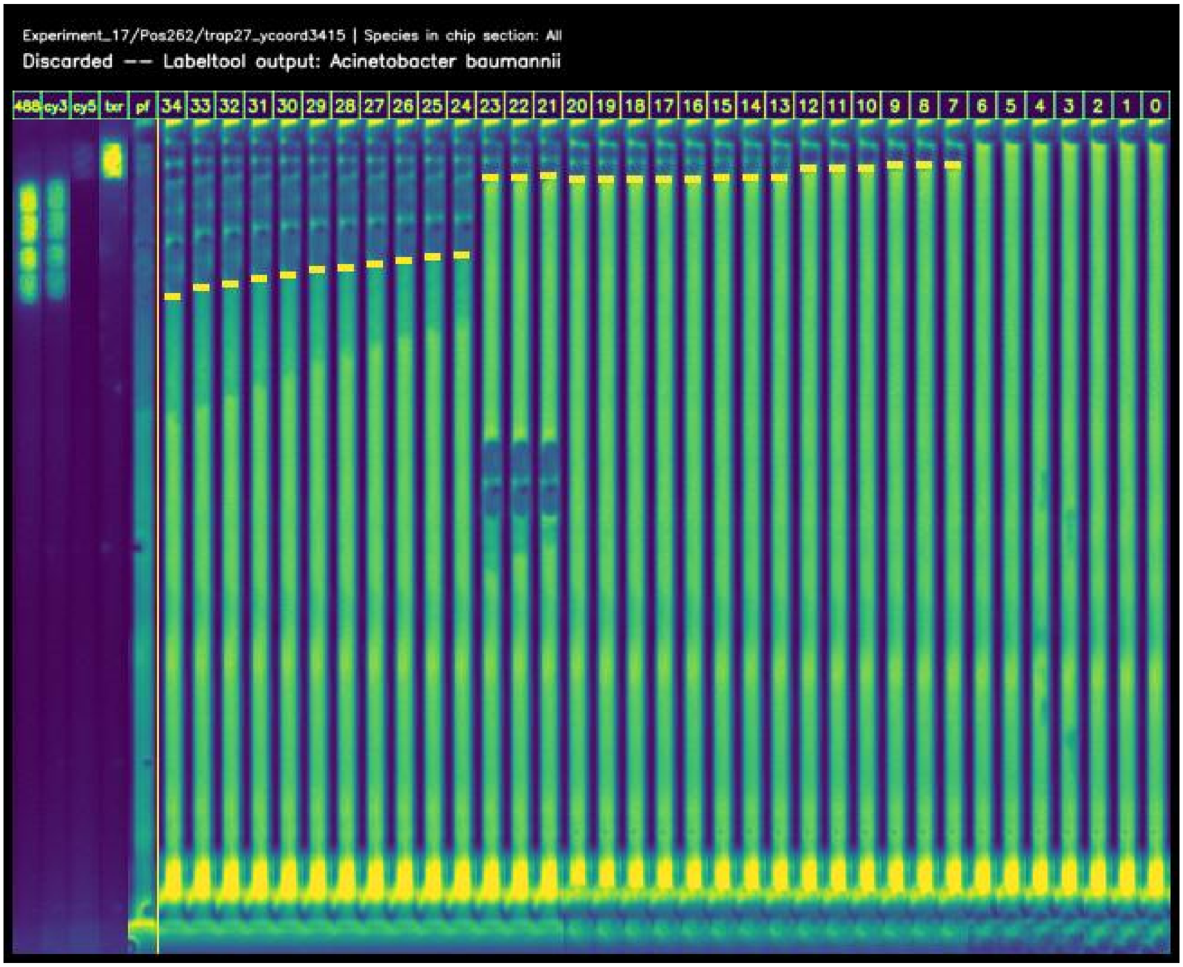
Experiment_17/Pos262/trap27_ycoord3415 | Species in chip section: All
Discarded -- Labeltool output: Acinetobacter baumannii
488 cy3 cy5 txr pf 34 33 32 31 30 29 28 27 26 25 24 23 22 21 20 19 18 17 16 15 14 13 12 11 10 9 8 7 6 5 4 3 2 1 0

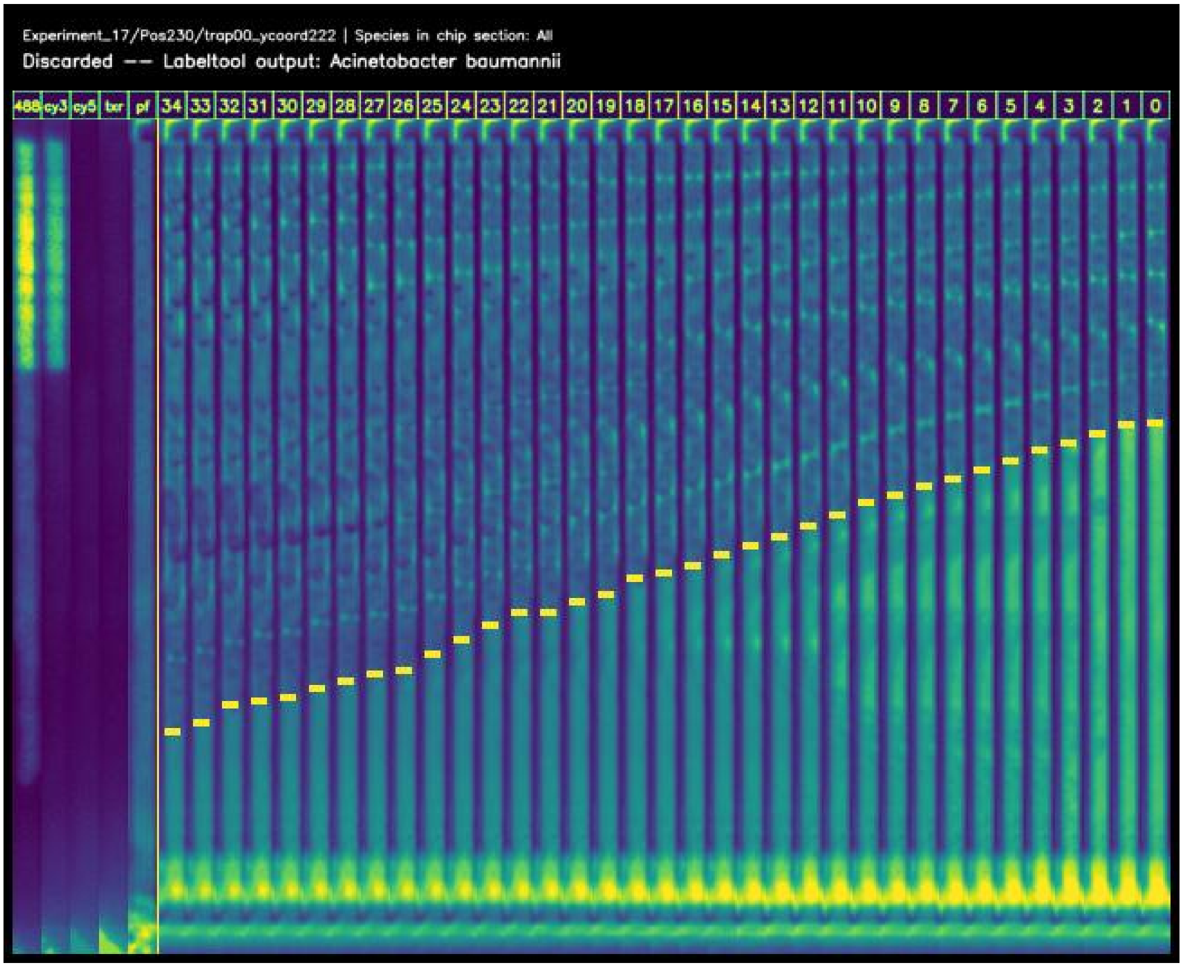
Experiment_17/Pos230/trap00_ycoord222 | Species in chip section: All
Discarded -- Labeltool output: Acinetobacter baumannii
488 cy3 cy5 txr pf 34 33 32 31 30 29 28 27 26 25 24 23 22 21 20 19 18 17 16 15 14 13 12 11 10 9 8 7 6 5 4 3 2 1 0

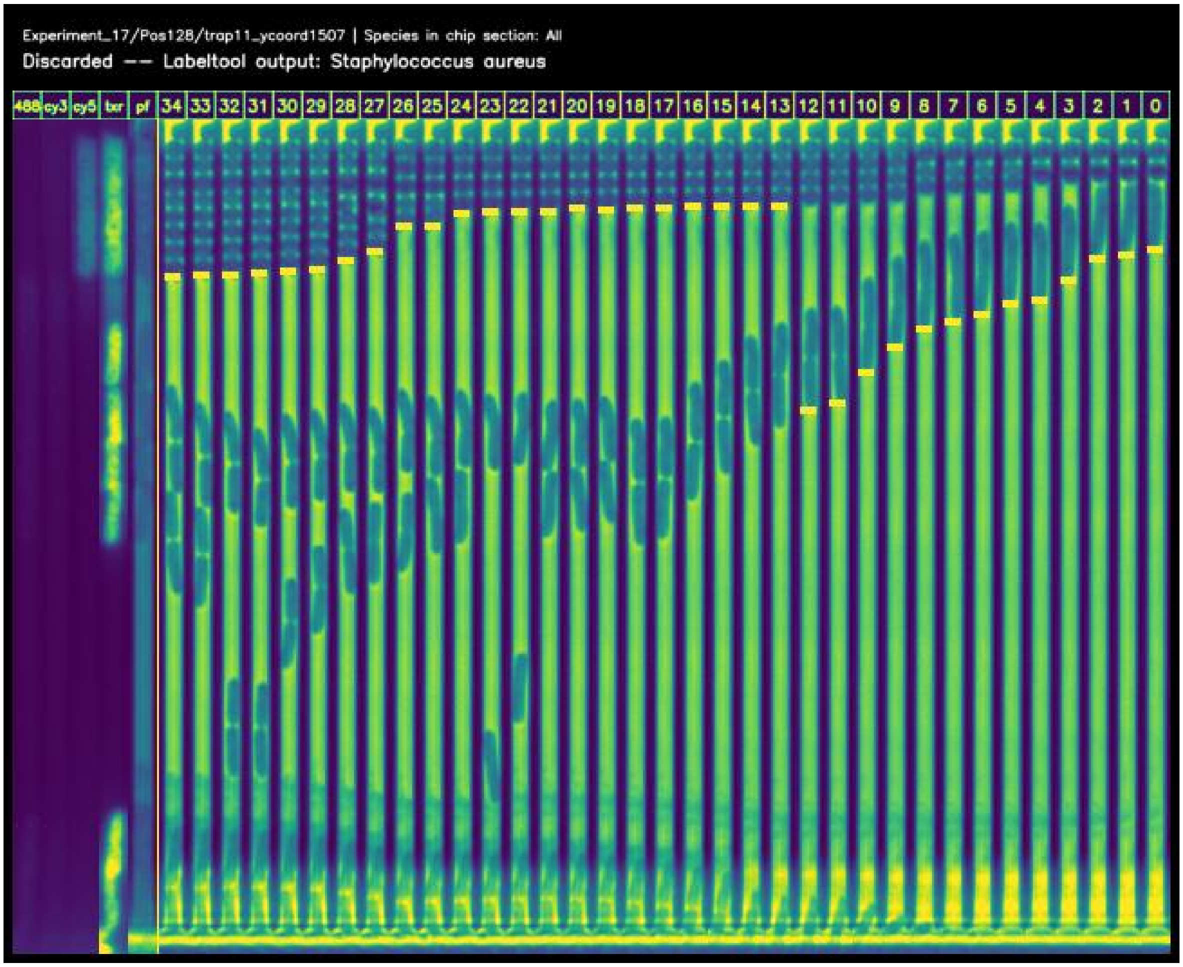
Experiment_17/Pos128/trap11_ycoord1507 | Species in chip section: All
Discarded -- Labeltool output: Staphylococcus aureus
488 cy3 cy5 txr pf 34 33 32 31 30 29 28 27 26 25 24 23 22 21 20 19 18 17 16 15 14 13 12 11 10 9 8 7 6 5 4 3 2 1 0

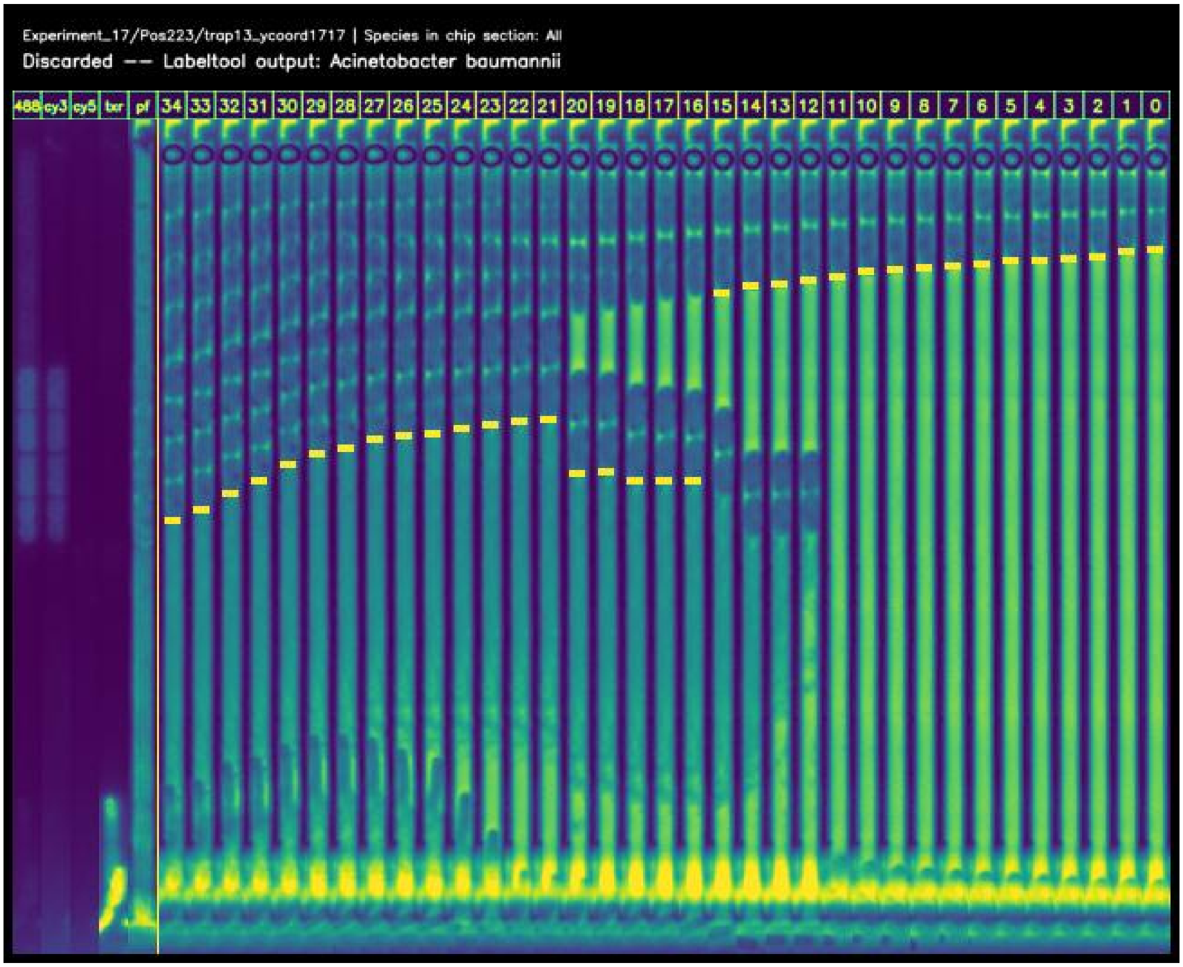
Experiment_17/Pos223/trap13_ycoord1717 | Species in chip section: All
Discarded -- Labeltool output: Acinetobacter baumannii
488 cy3 cy5 txr pf 34 33 32 31 30 29 28 27 26 25 24 23 22 21 20 19 18 17 16 15 14 13 12 11 10 9 8 7 6 5 4 3 2 1 0

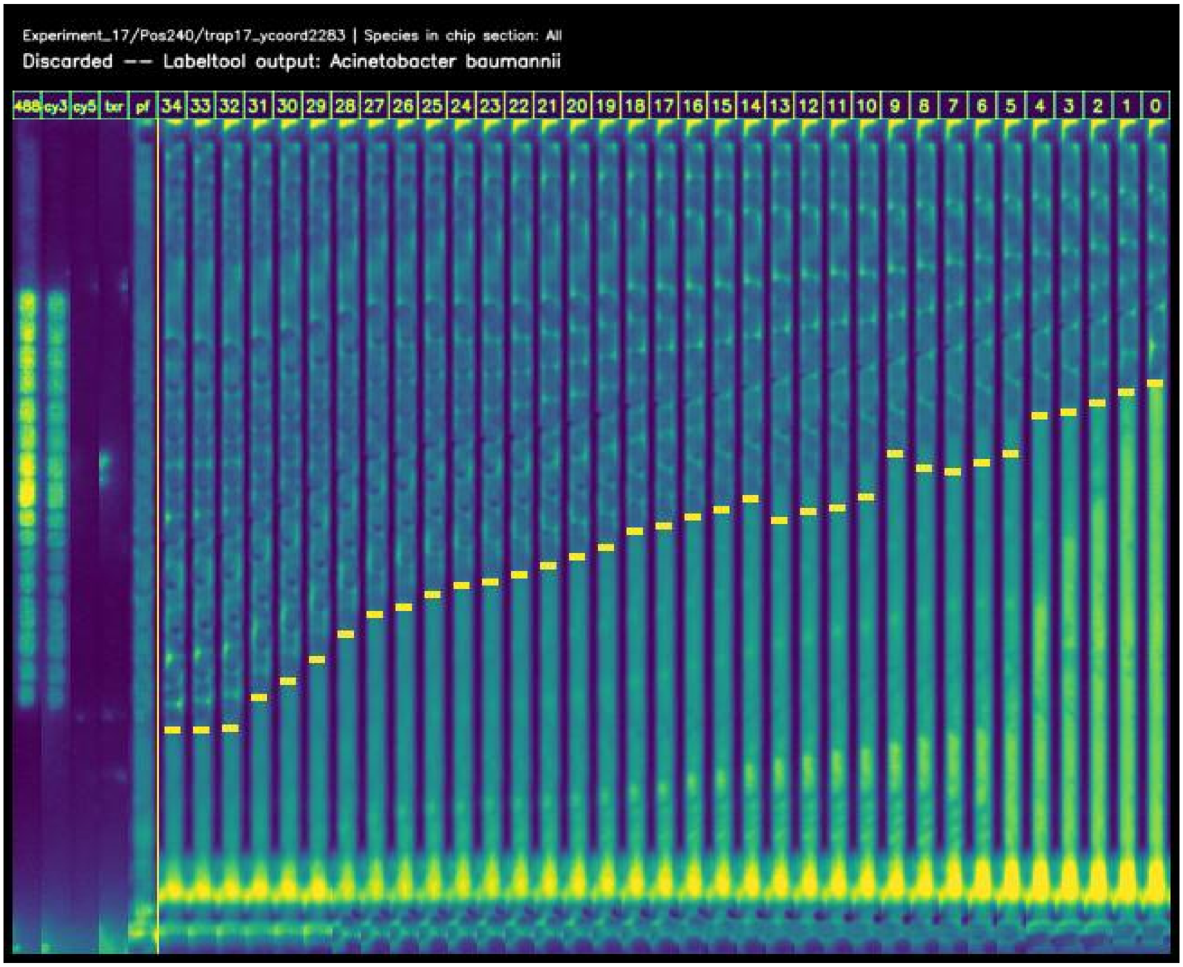
Experiment_17/Pos240/trap17_ycoord2283 | Species in chip section: All
Discarded -- Labeltool output: Acinetobacter baumannii
488 cy3 cy5 txr pf 34 33 32 31 30 29 28 27 26 25 24 23 22 21 20 19 18 17 16 15 14 13 12 11 10 9 8 7 6 5 4 3 2 1 0

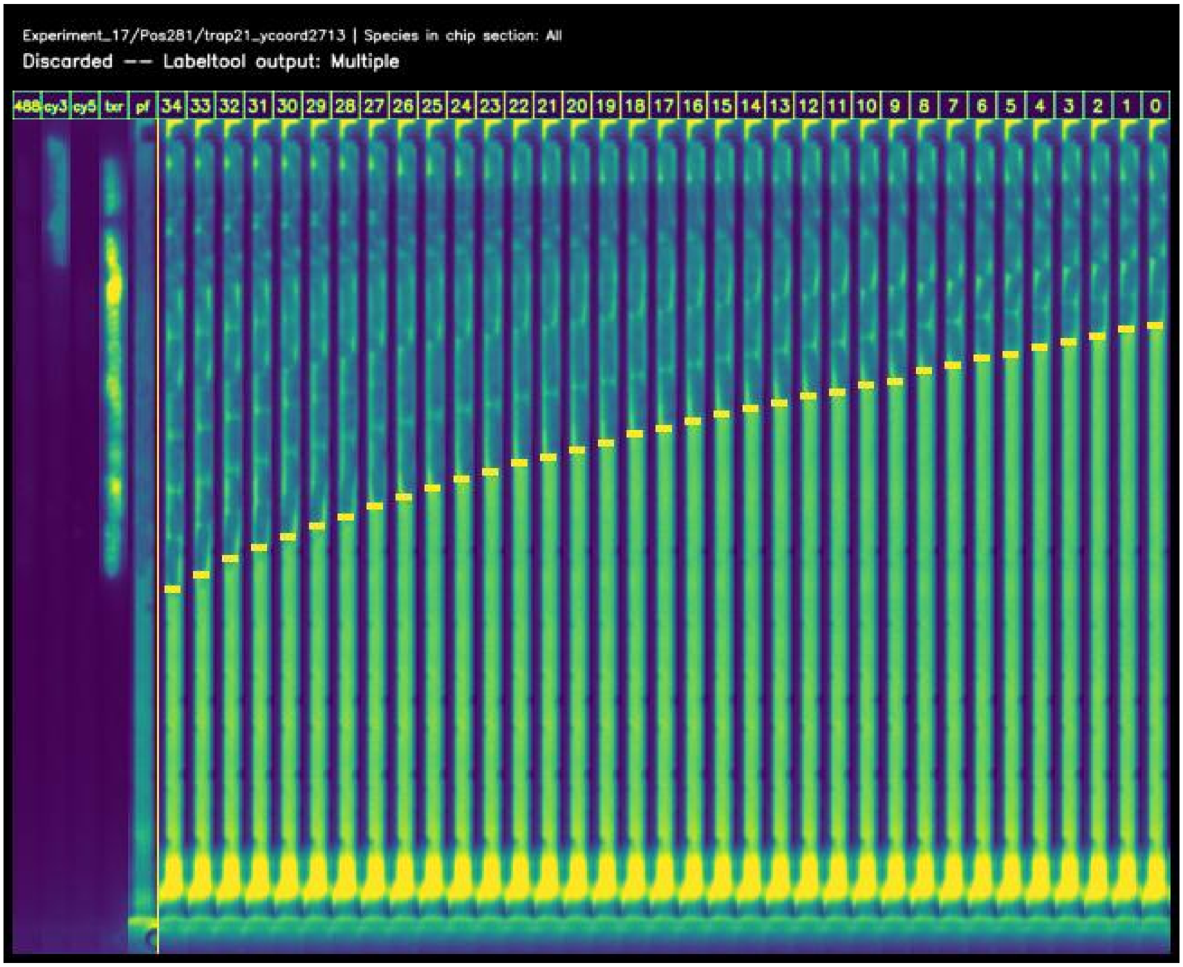
Experiment_17/Pos281/trap21_ycoord2713 | Species in chip section: All
Discarded -- Labeltool output: Multiple
488 cy3 cy5 txr pf 34 33 32 31 30 29 28 27 26 25 24 23 22 21 20 19 18 17 16 15 14 13 12 11 10 9 8 7 6 5 4 3 2 1 0

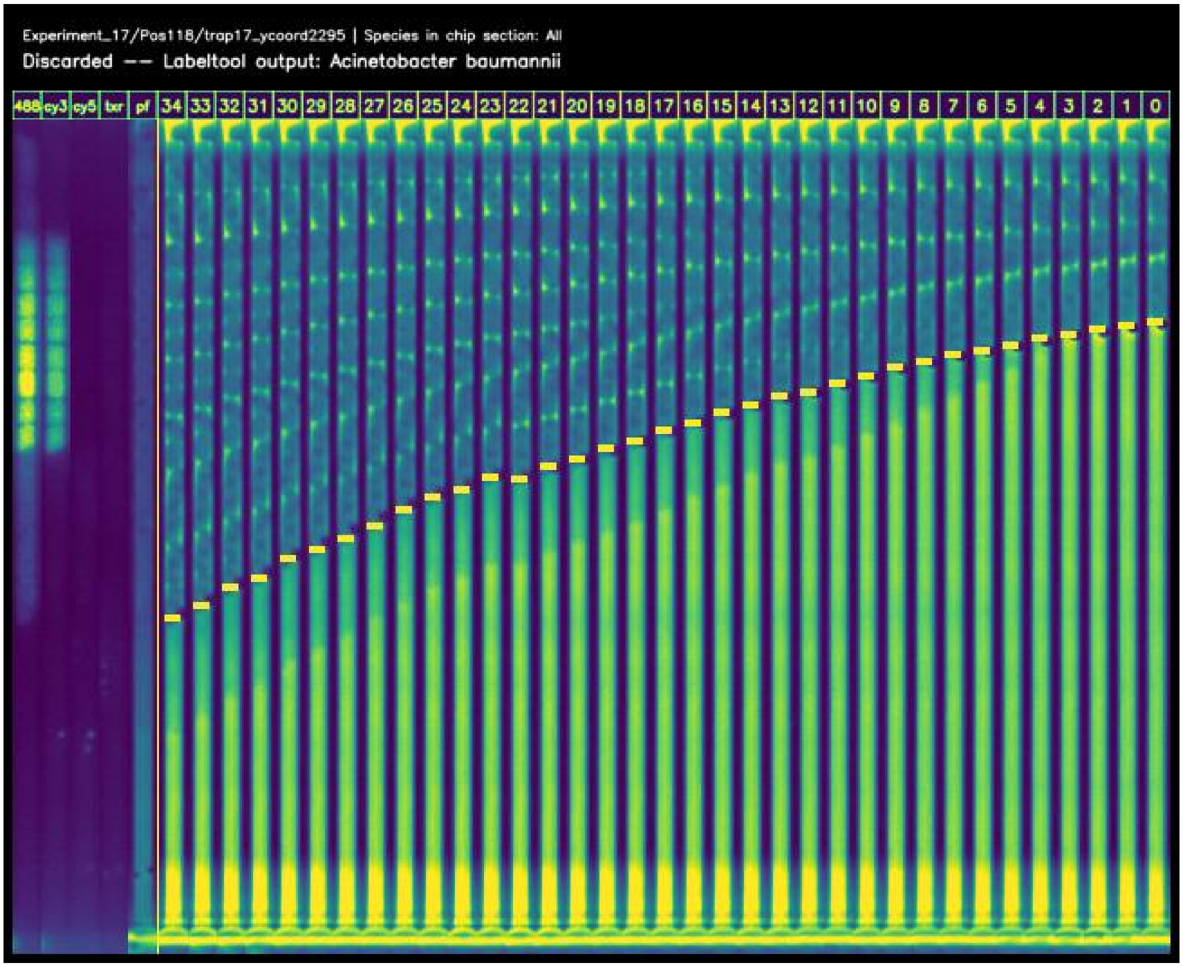
Experiment_17/Pos118/trap17_ycoord2295 | Species in chip section: All
Discarded -- Labeltool output: Acinetobacter baumannii
488 cy3 cy5 txr pf 34 33 32 31 30 29 28 27 26 25 24 23 22 21 20 19 18 17 16 15 14 13 12 11 10 9 8 7 6 5 4 3 2 1 0

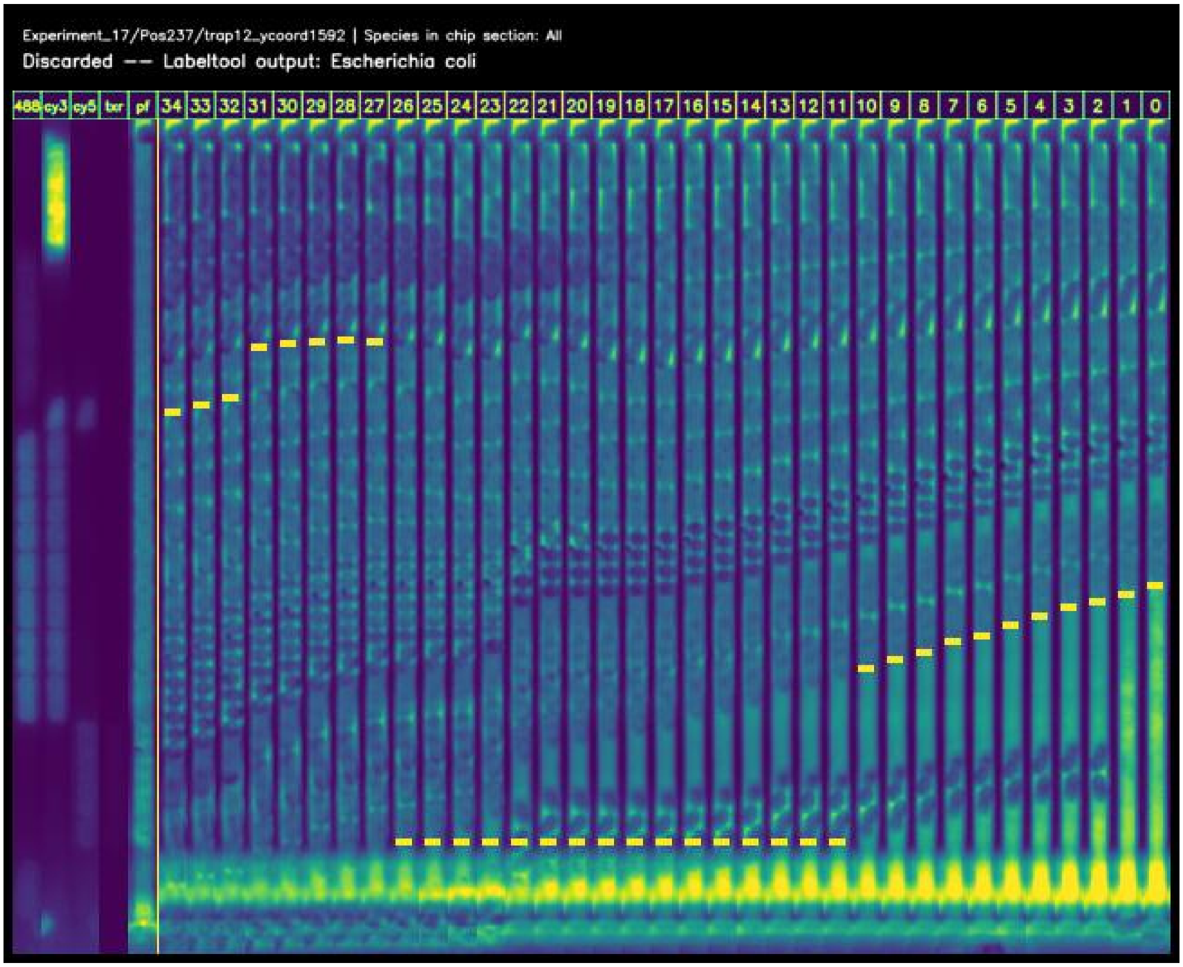
Experiment_17/Pos237/trap12_ycoord1592 | Species in chip section: All
Discarded -- Labeltool output: Escherichia coli
488 cy3 cy5 txr pf 34 33 32 31 30 29 28 27 26 25 24 23 22 21 20 19 18 17 16 15 14 13 12 11 10 9 8 7 6 5 4 3 2 1 0

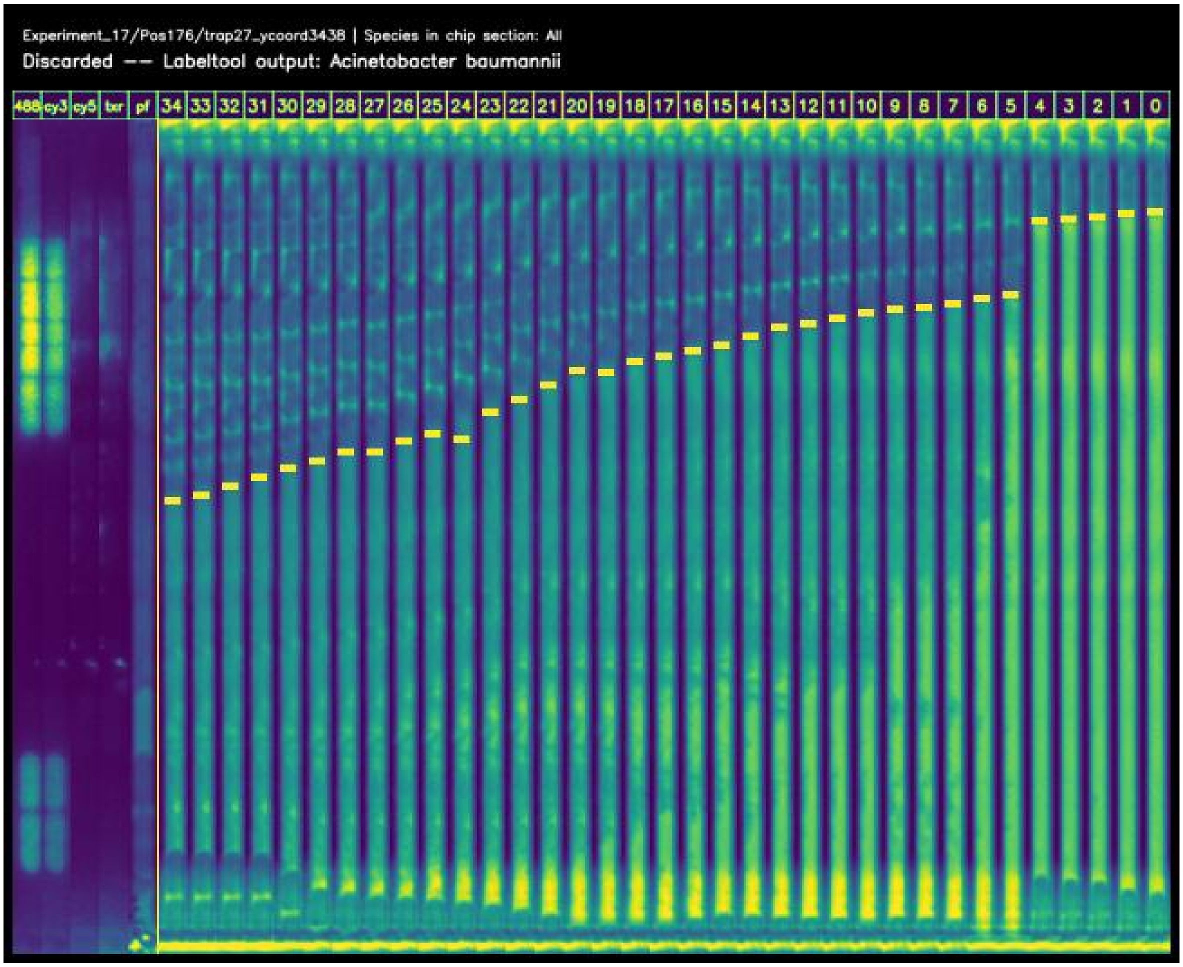
Experiment_17/Pos176/trap27_ycoord3438 | Species in chip section: All
Discarded -- Labeltool output: Acinetobacter baumannii
488 cy3 cy5 txr pf 34 33 32 31 30 29 28 27 26 25 24 23 22 21 20 19 18 17 16 15 14 13 12 11 10 9 8 7 6 5 4 3 2 1 0

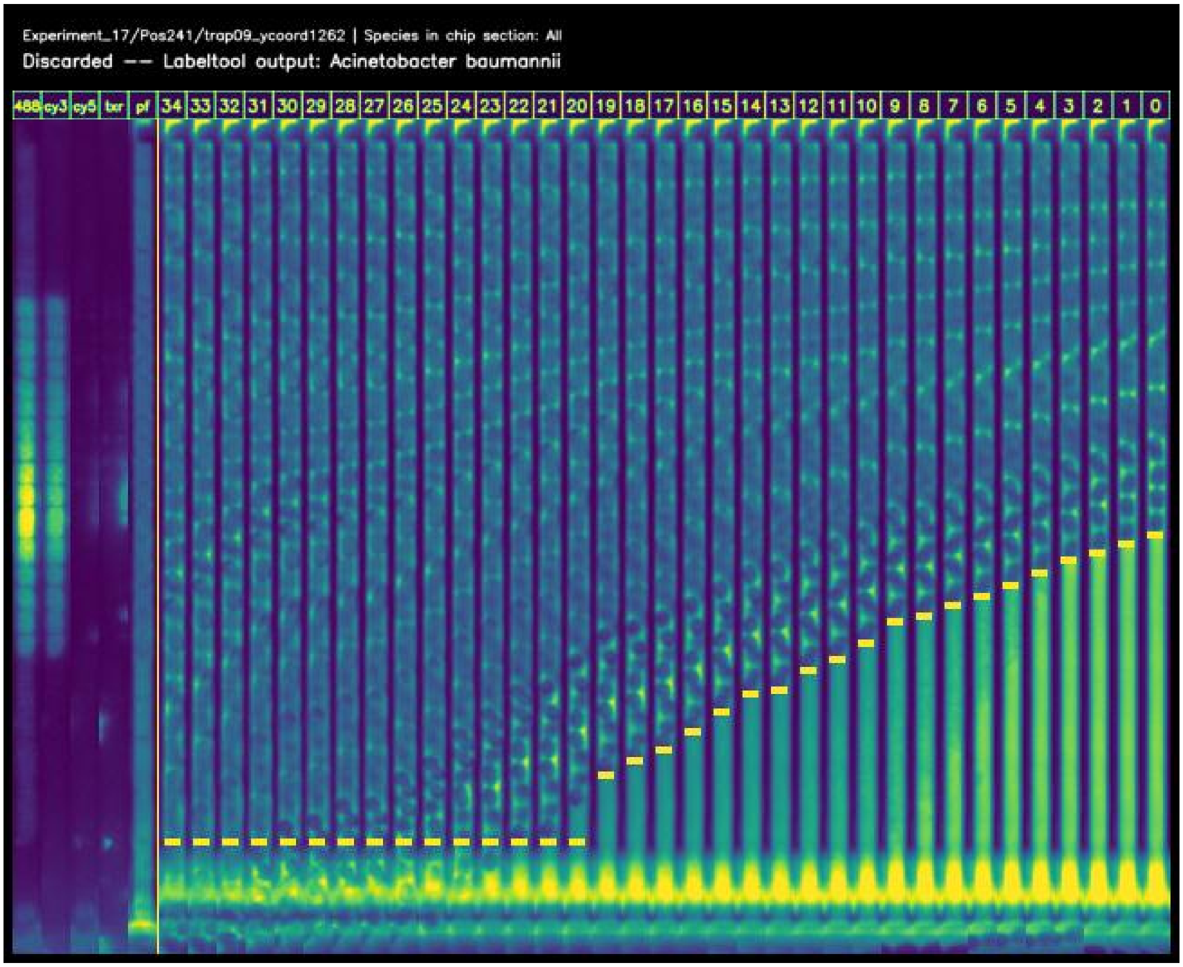
Experiment_17/Pos241/trap09_ycoord1262 | Species in chip section: All
Discarded -- Labeltool output: Acinetobacter baumannii
488 cy3 cy5 txr pf 34 33 32 31 30 29 28 27 26 25 24 23 22 21 20 19 18 17 16 15 14 13 12 11 10 9 8 7 6 5 4 3 2 1 0

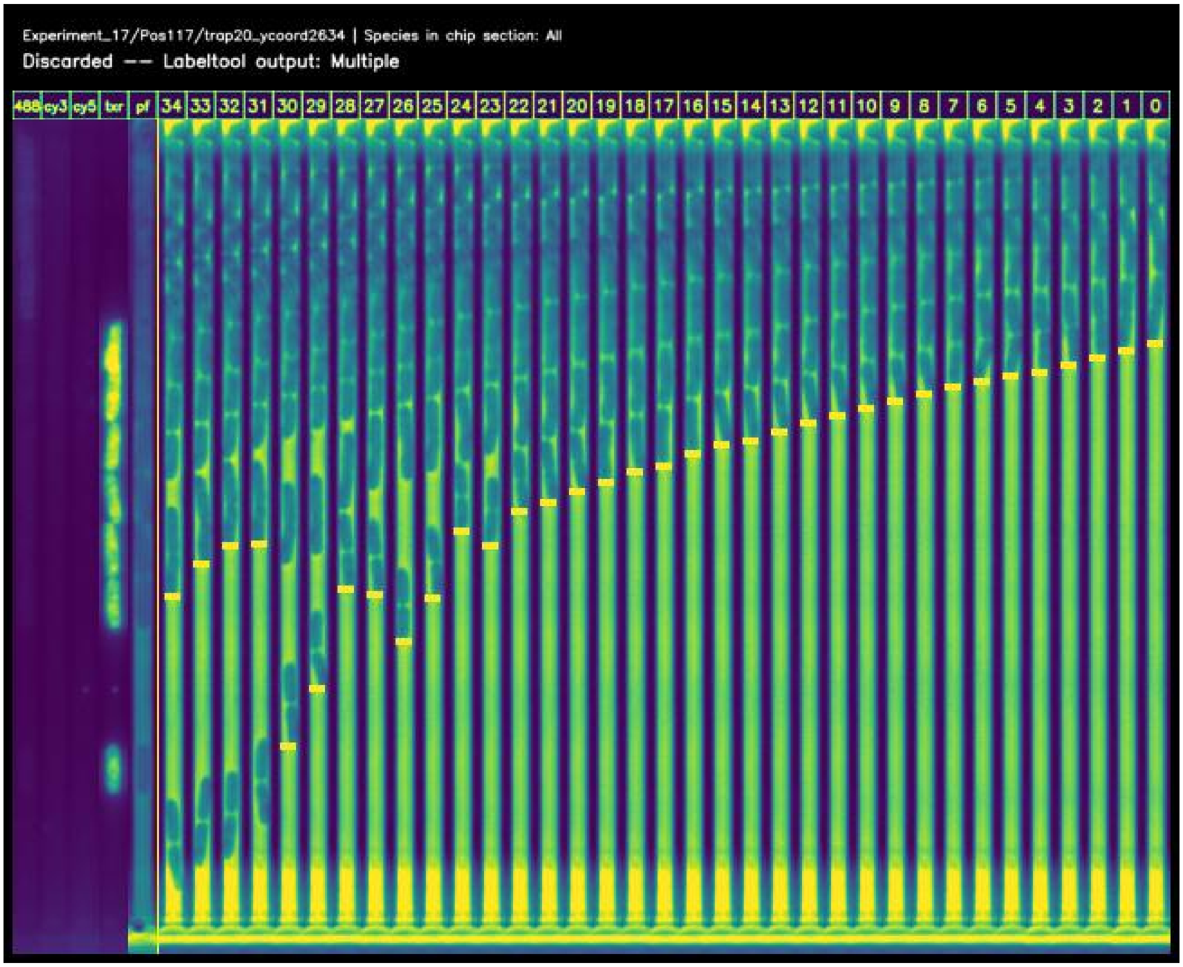
Experiment_17/Pos117/trap20_ycoord2634 | Species in chip section: All
Discarded -- Labeltool output: Multiple
488 cy3 cy5 txr pf 34 33 32 31 30 29 28 27 26 25 24 23 22 21 20 19 18 17 16 15 14 13 12 11 10 9 8 7 6 5 4 3 2 1 0

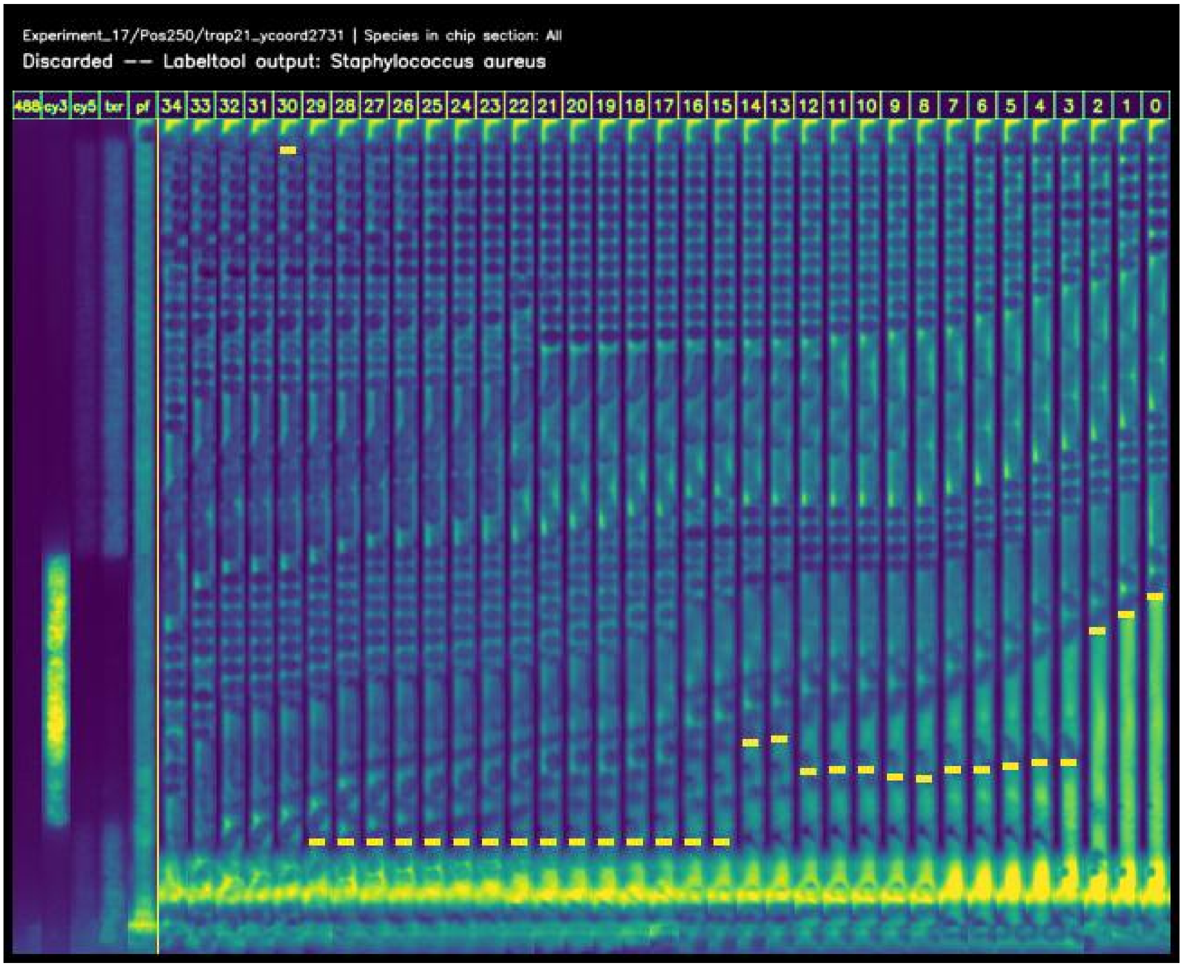
Experiment_17/Pos250/trap21_ycoord2731 | Species in chip section: All
Discarded -- Labeltool output: Staphylococcus aureus
488 cy3 cy5 txr pf 34 33 32 31 30 29 28 27 26 25 24 23 22 21 20 19 18 17 16 15 14 13 12 11 10 9 8 7 6 5 4 3 2 1 0

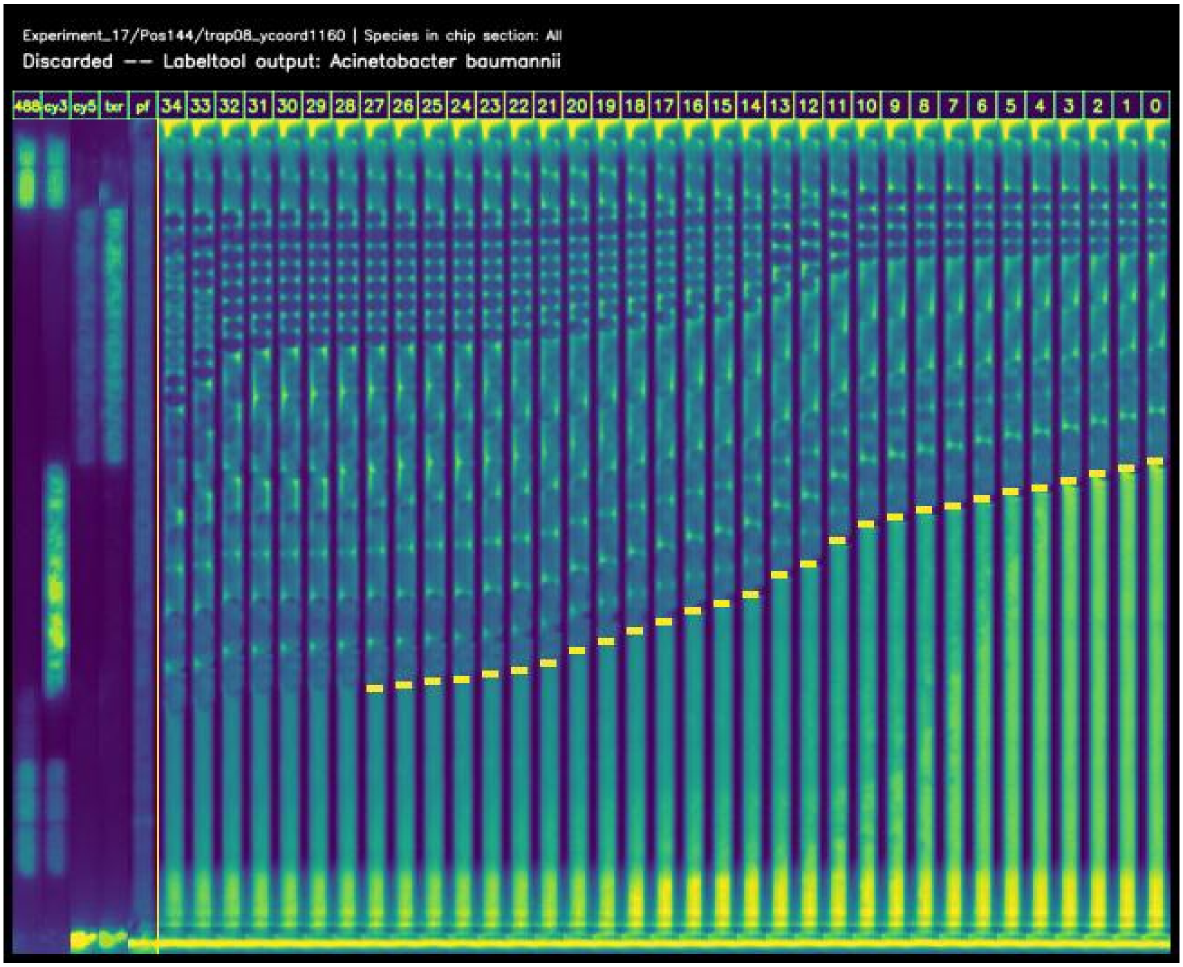
Experiment_17/Pos144/trap08_ycoord1160 | Species in chip section: All
Discarded -- Labeltool output: Acinetobacter baumannii
488 cy3 cy5 txr pf 34 33 32 31 30 29 28 27 26 25 24 23 22 21 20 19 18 17 16 15 14 13 12 11 10 9 8 7 6 5 4 3 2 1 0

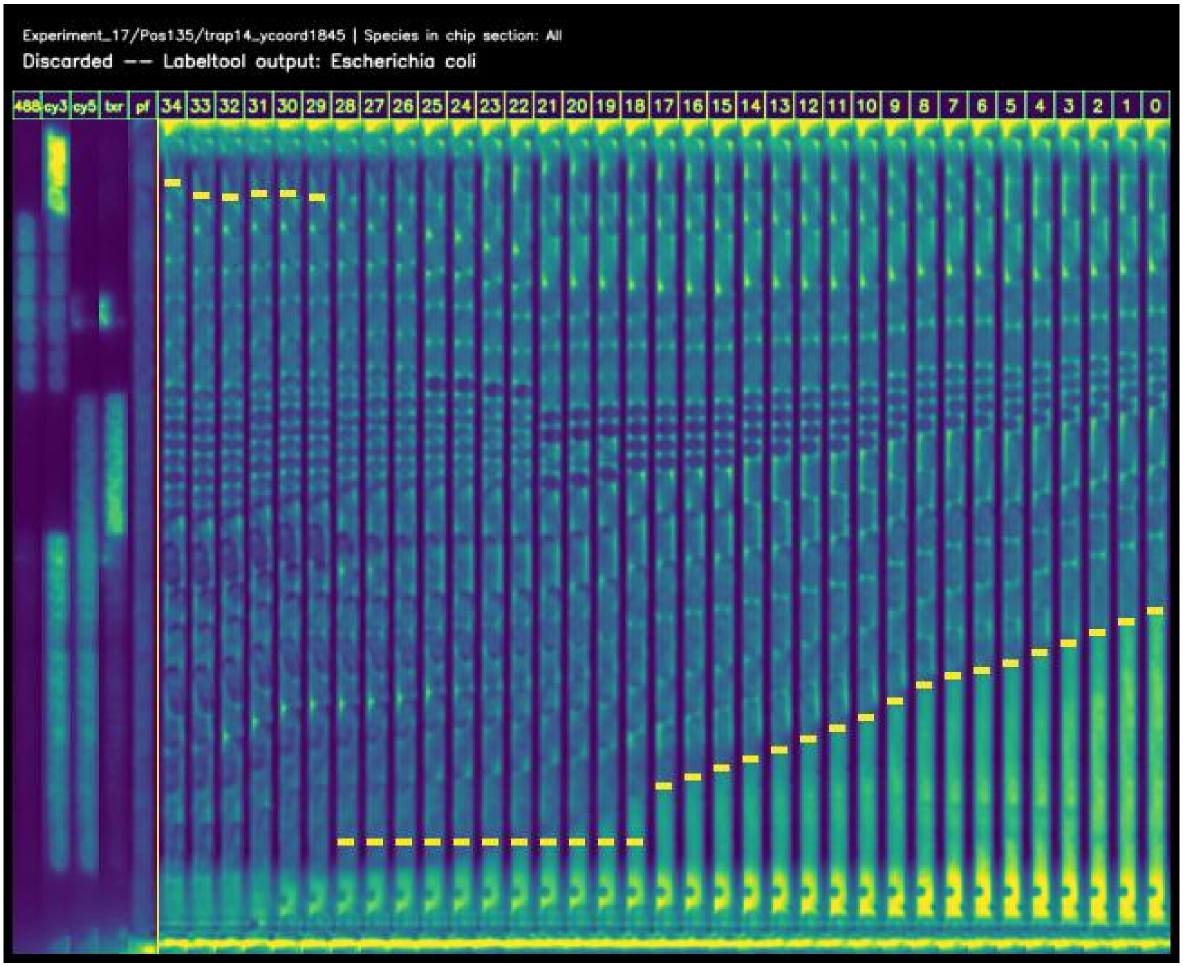
Experiment_17/Pos135/trap14_ycoord1845 | Species in chip section: All
Discarded -- Labeltool output: Escherichia coli
488 cy3 cy5 txr pf 34 33 32 31 30 29 28 27 26 25 24 23 22 21 20 19 18 17 16 15 14 13 12 11 10 9 8 7 6 5 4 3 2 1 0

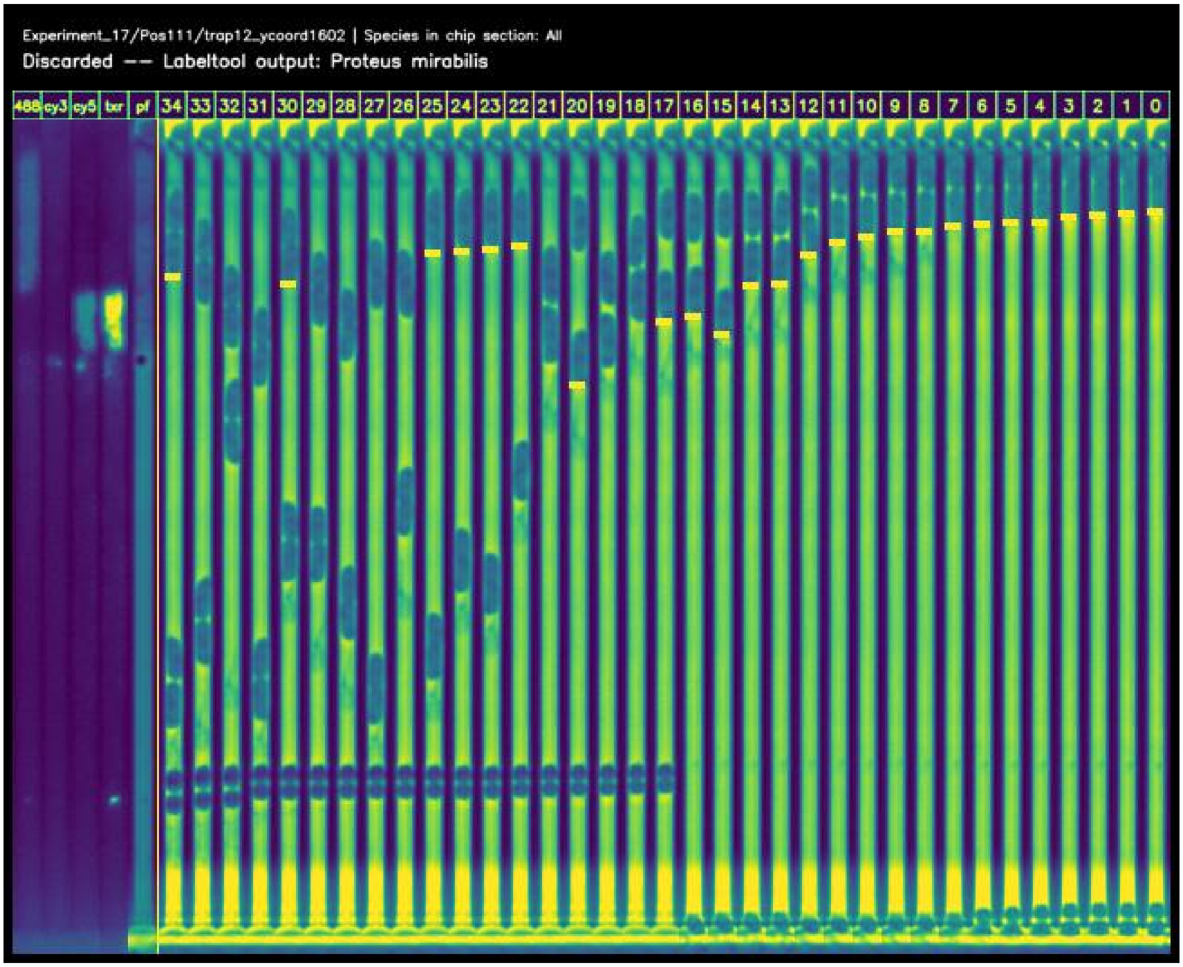
Experiment_17/Pos111/trap12_ycoord1602 | Species in chip section: All
Discarded -- Labeltool output: Proteus mirabilis
488 cy3 cy5 txr pf 34 33 32 31 30 29 28 27 26 25 24 23 22 21 20 19 18 17 16 15 14 13 12 11 10 9 8 7 6 5 4 3 2 1 0

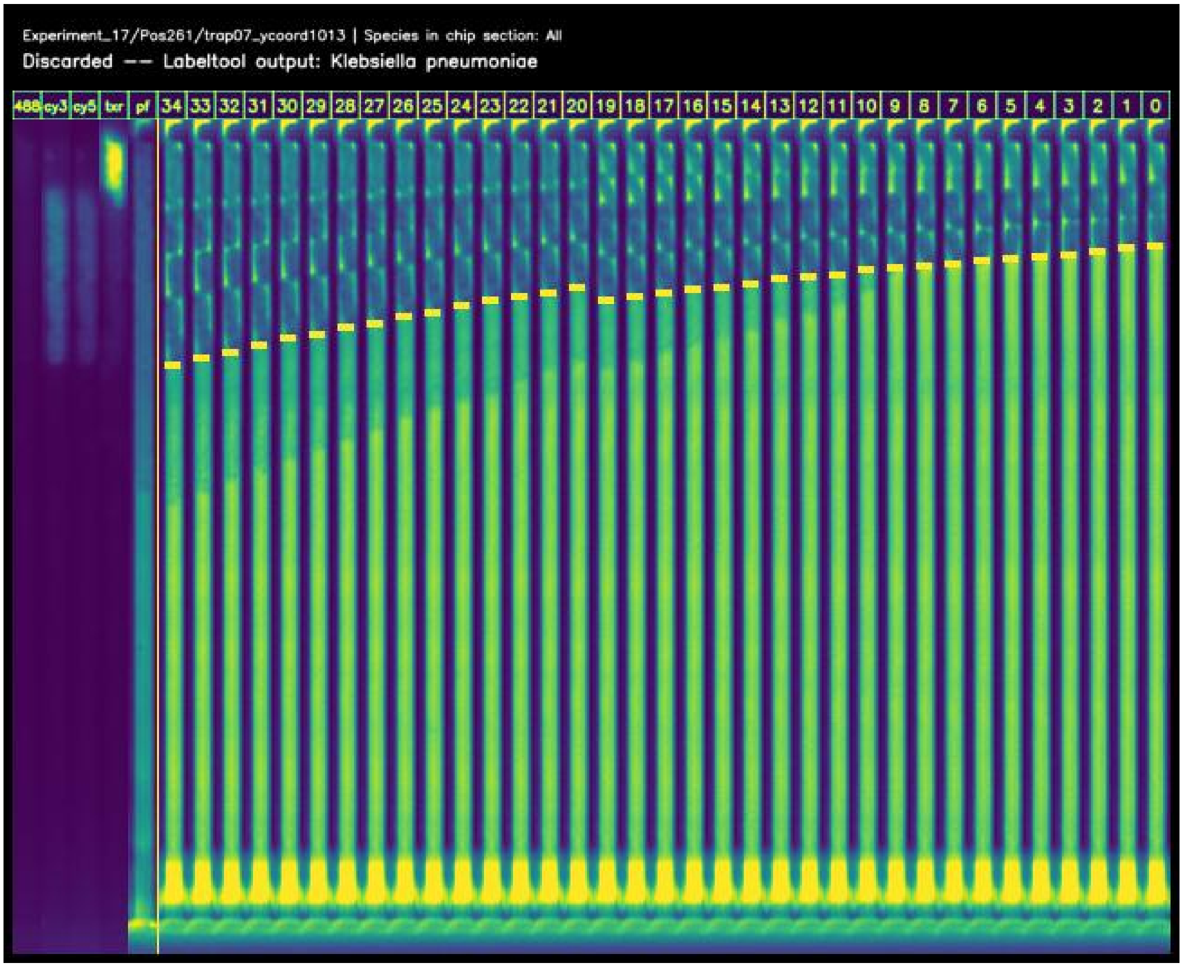
Experiment_17/Pos261/trap07_ycoord1013 | Species in chip section: All
Discarded -- Labeltool output: Klebsiella pneumoniae
488 cy3 cy5 txr pf 34 33 32 31 30 29 28 27 26 25 24 23 22 21 20 19 18 17 16 15 14 13 12 11 10 9 8 7 6 5 4 3 2 1 0

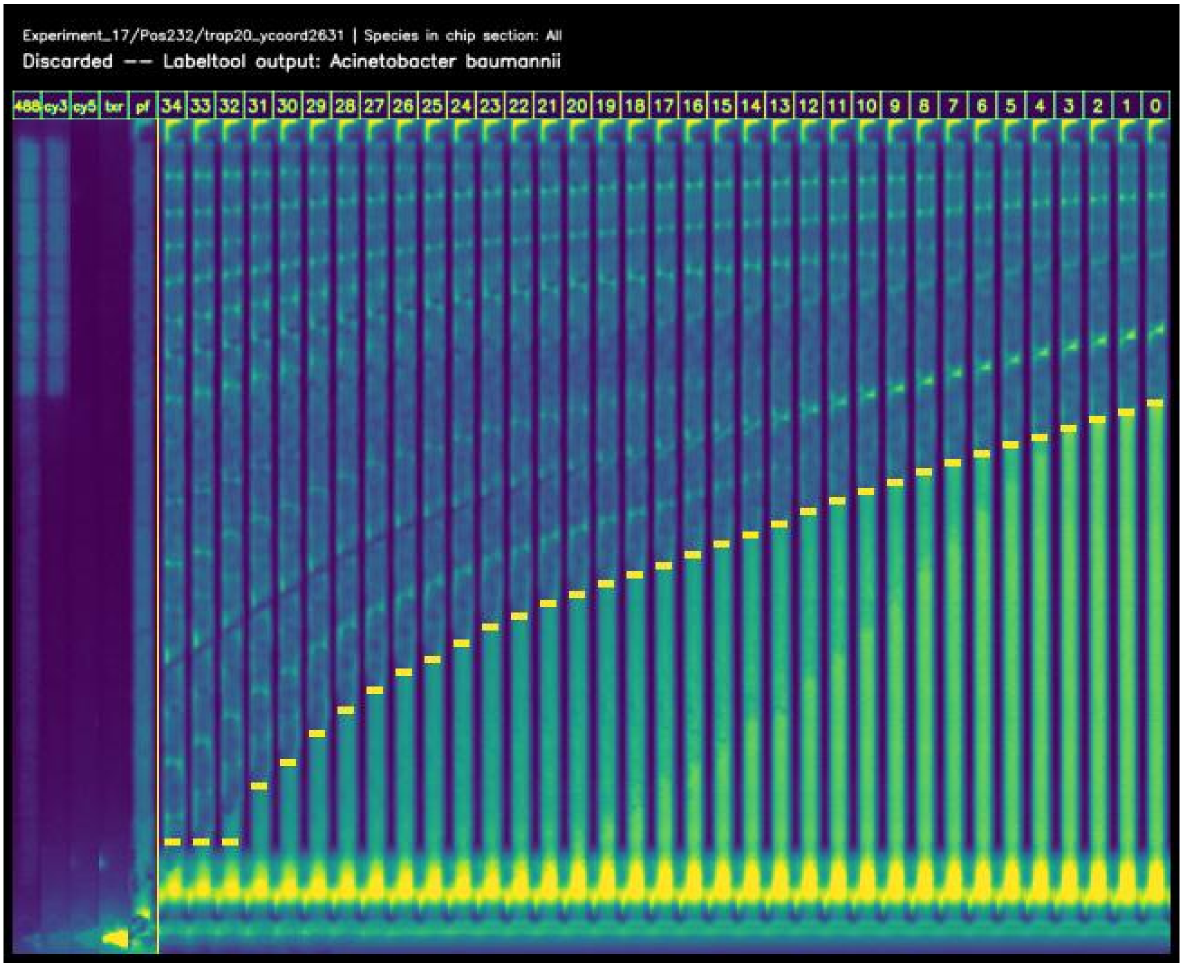
Experiment_17/Pos232/trap20_ycoord2631 | Species in chip section: All
Discarded -- Labeltool output: Acinetobacter baumannii
488 cy3 cy5 txr pf 34 33 32 31 30 29 28 27 26 25 24 23 22 21 20 19 18 17 16 15 14 13 12 11 10 9 8 7 6 5 4 3 2 1 0

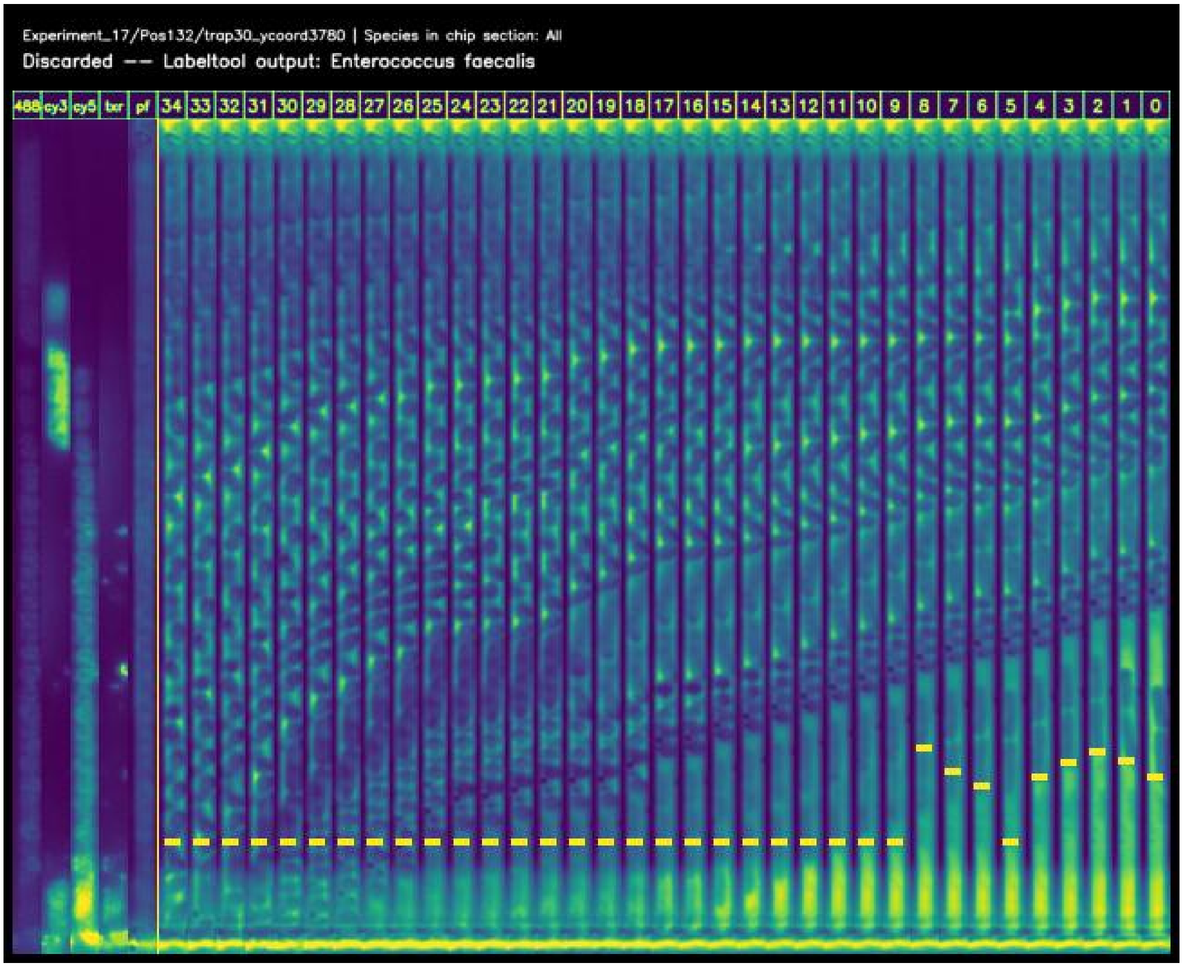
Experiment_17/Pos132/trap30_ycoord3780 | Species in chip section: All
Discarded -- Labeltool output: Enterococcus faecalis
488 cy3 cy5 txr pf 34 33 32 31 30 29 28 27 26 25 24 23 22 21 20 19 18 17 16 15 14 13 12 11 10 9 8 7 6 5 4 3 2 1 0

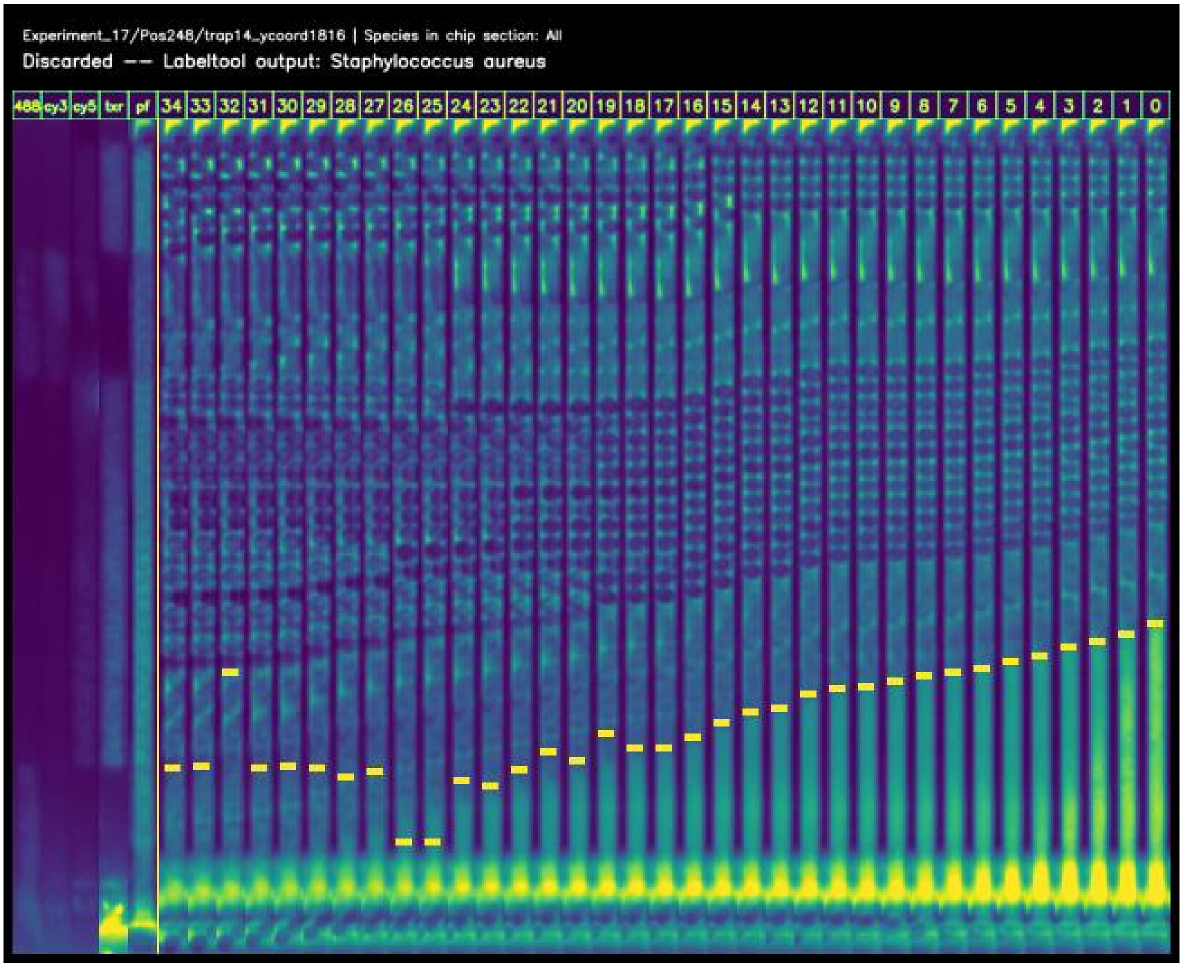
Experiment_17/Pos248/trap14_ycoord1816 | Species in chip section: All
Discarded -- Labeltool output: Staphylococcus aureus
488 cy3 cy5 txr pf 34 33 32 31 30 29 28 27 26 25 24 23 22 21 20 19 18 17 16 15 14 13 12 11 10 9 8 7 6 5 4 3 2 1 0

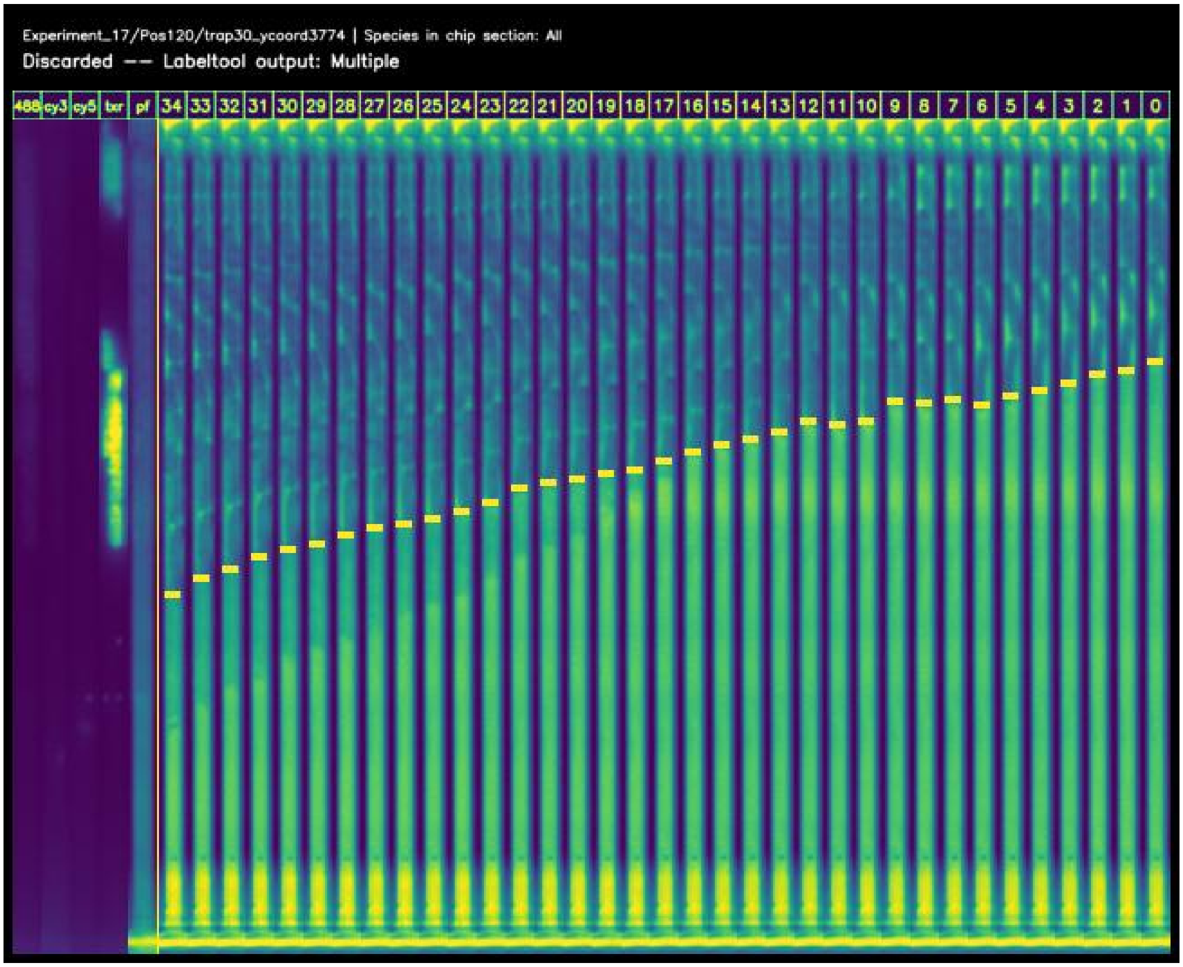
Experiment_17/Pos120/trap30_ycoord3774 | Species in chip section: All
Discarded -- Labeltool output: Multiple
488 cy3 cy5 txr pf 34 33 32 31 30 29 28 27 26 25 24 23 22 21 20 19 18 17 16 15 14 13 12 11 10 9 8 7 6 5 4 3 2 1 0

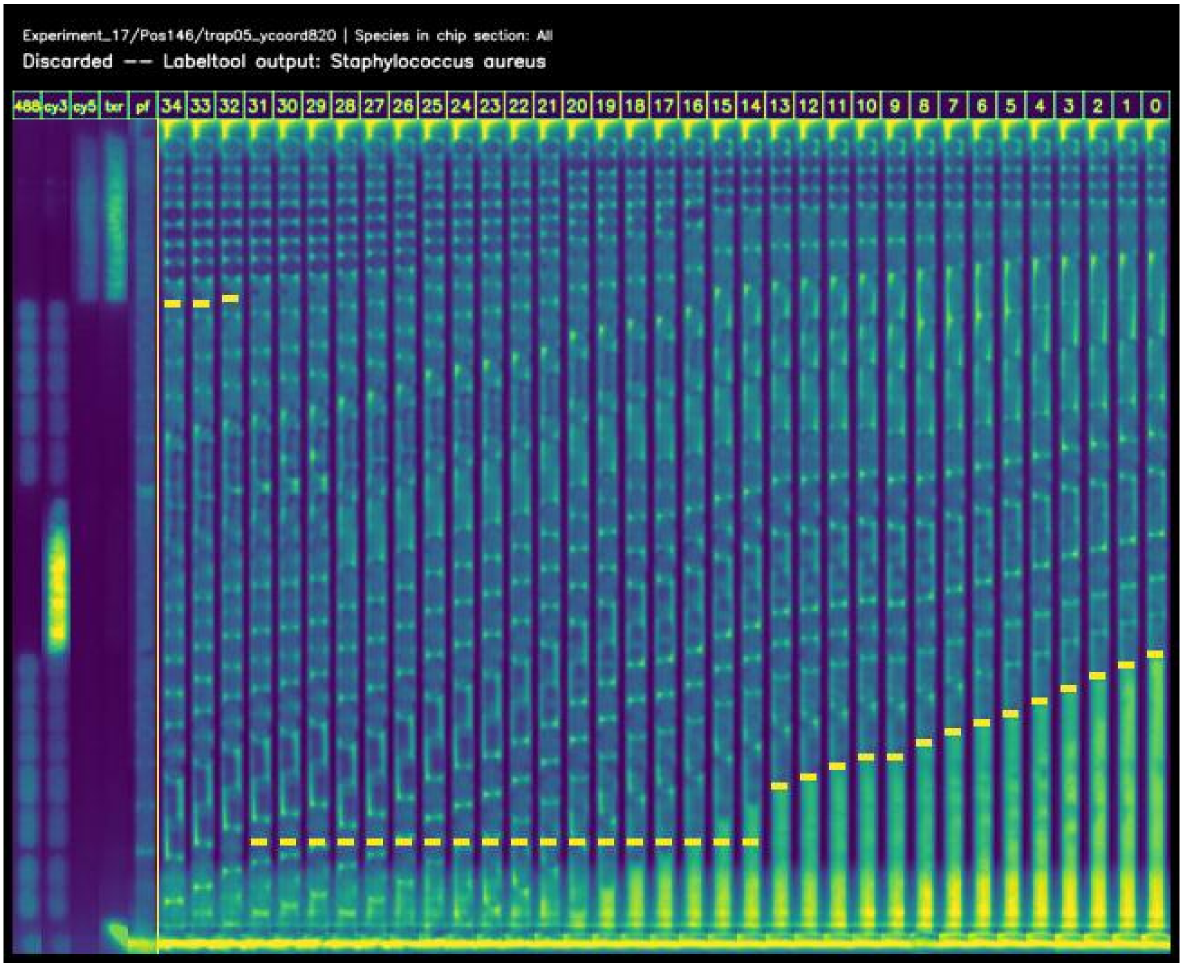
Experiment_17/Pos146/trap05_ycoord820 | Species in chip section: All
Discarded -- Labeltool output: Staphylococcus aureus
488 cy3 cy5 txr pf 34 33 32 31 30 29 28 27 26 25 24 23 22 21 20 19 18 17 16 15 14 13 12 11 10 9 8 7 6 5 4 3 2 1 0

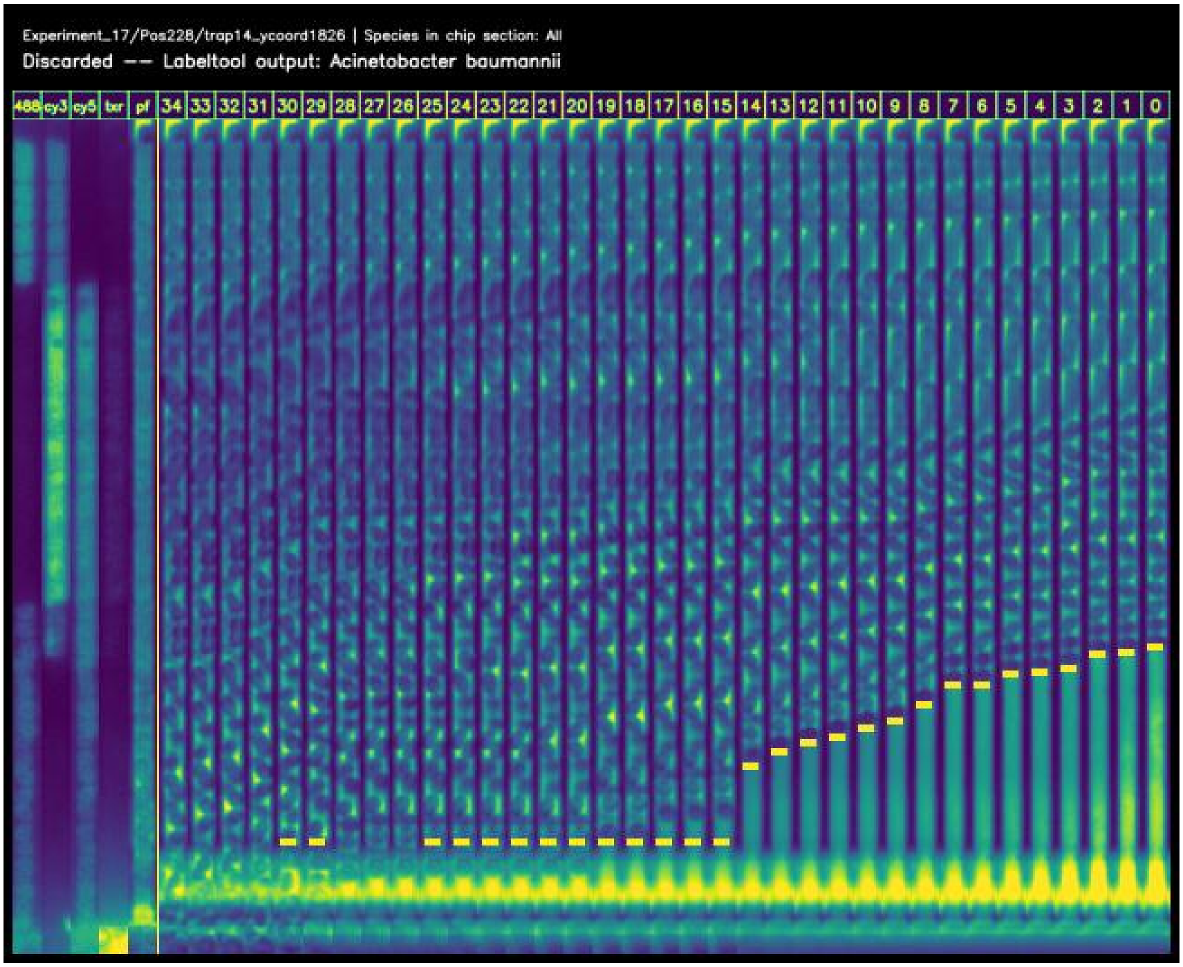
Experiment_17/Pos228/trap14_ycoord1826 | Species in chip section: All
Discarded -- Labeltool output: Acinetobacter baumannii
488 cy3 cy5 txr pf 34 33 32 31 30 29 28 27 26 25 24 23 22 21 20 19 18 17 16 15 14 13 12 11 10 9 8 7 6 5 4 3 2 1 0

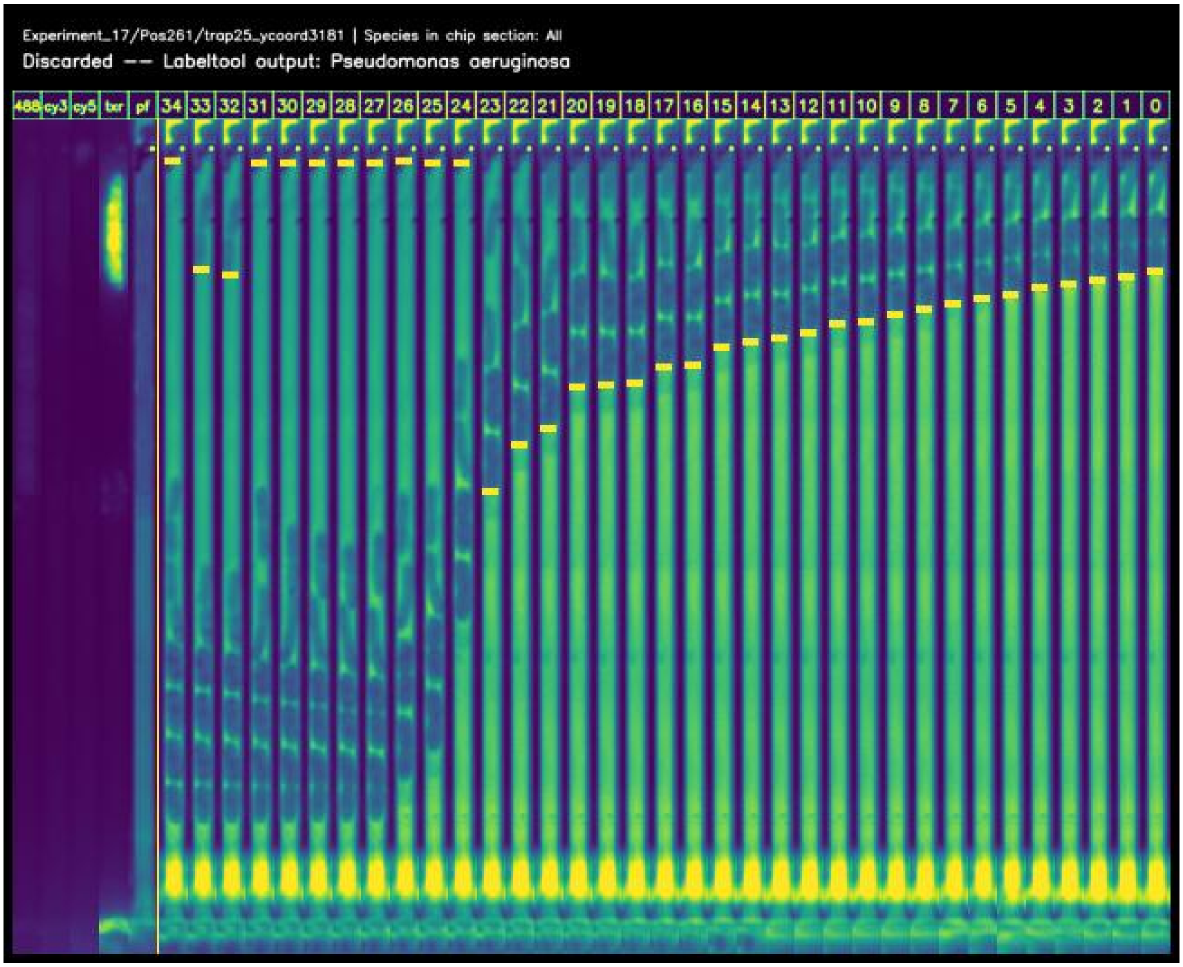
Experiment_17/Pos261/trap25_ycoord3181 | Species in chip section: All
Discarded -- Labeltool output: Pseudomonas aeruginosa
488 cy3 cy5 txr pf 34 33 32 31 30 29 28 27 26 25 24 23 22 21 20 19 18 17 16 15 14 13 12 11 10 9 8 7 6 5 4 3 2 1 0

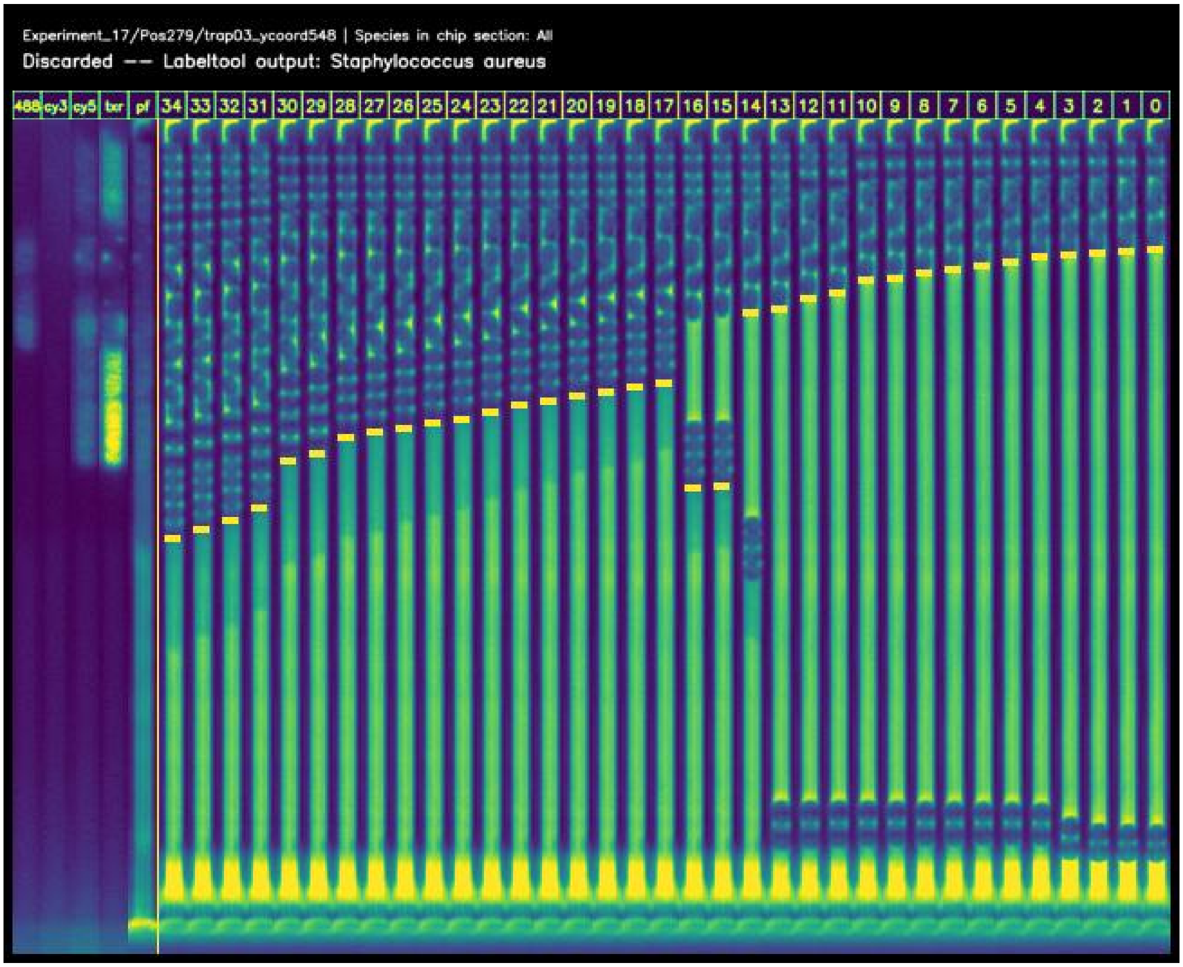

Experiment_17/Pos279/trap03_ycoord548 | Species in chip section: All
Discarded -- Labeltool output: Staphylococcus aureus
488 cy3 cy5 txr pf 34 33 32 31 30 29 28 27 26 25 24 23 22 21 20 19 18 17 16 15 14 13 12 11 10 9 8 7 6 5 4 3 2 1 0

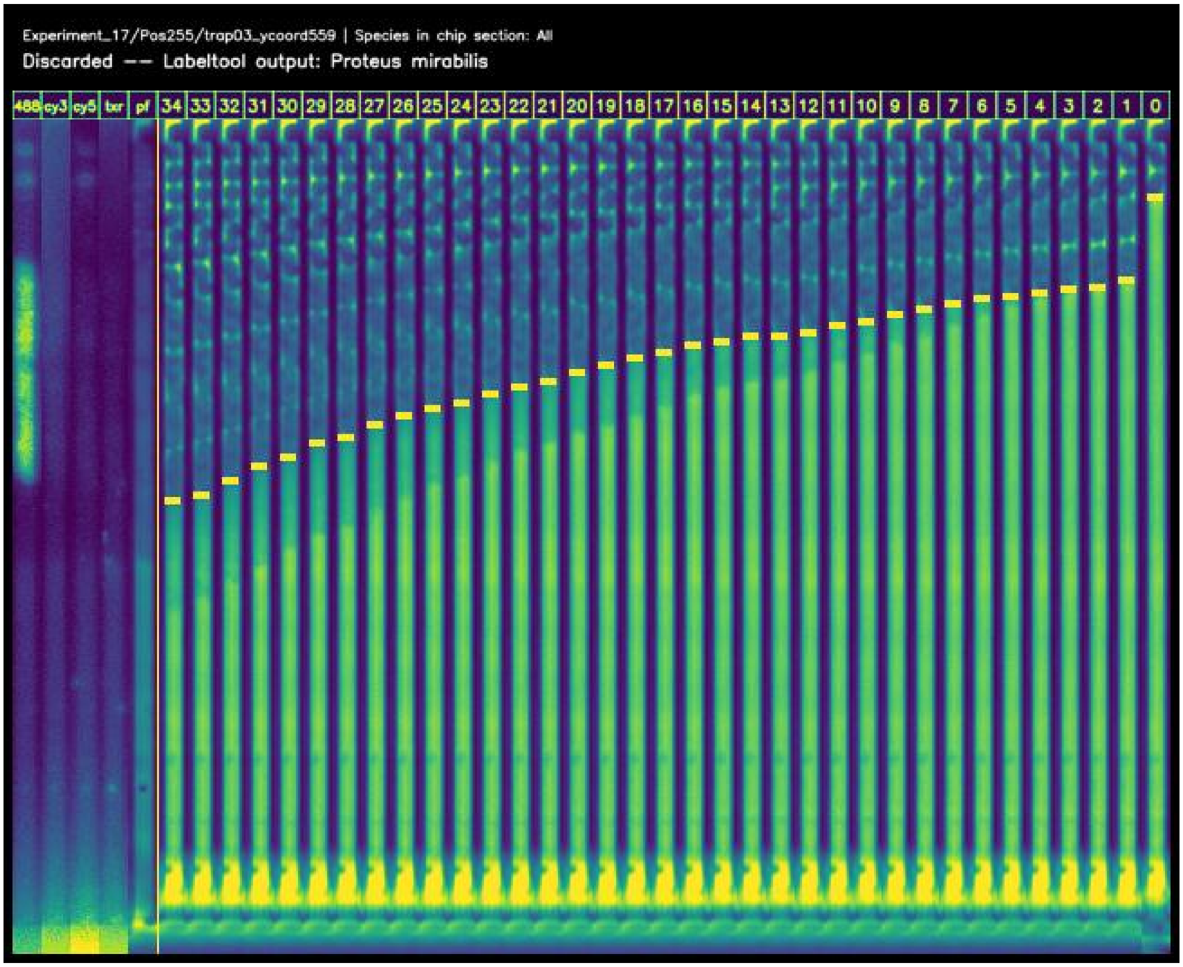

Experiment_17/Pos255/trap03_ycoord559 | Species in chip section: All
Discarded -- Labeltool output: Proteus mirabilis
488 cy3 cy5 txr pf 34 33 32 31 30 29 28 27 26 25 24 23 22 21 20 19 18 17 16 15 14 13 12 11 10 9 8 7 6 5 4 3 2 1 0

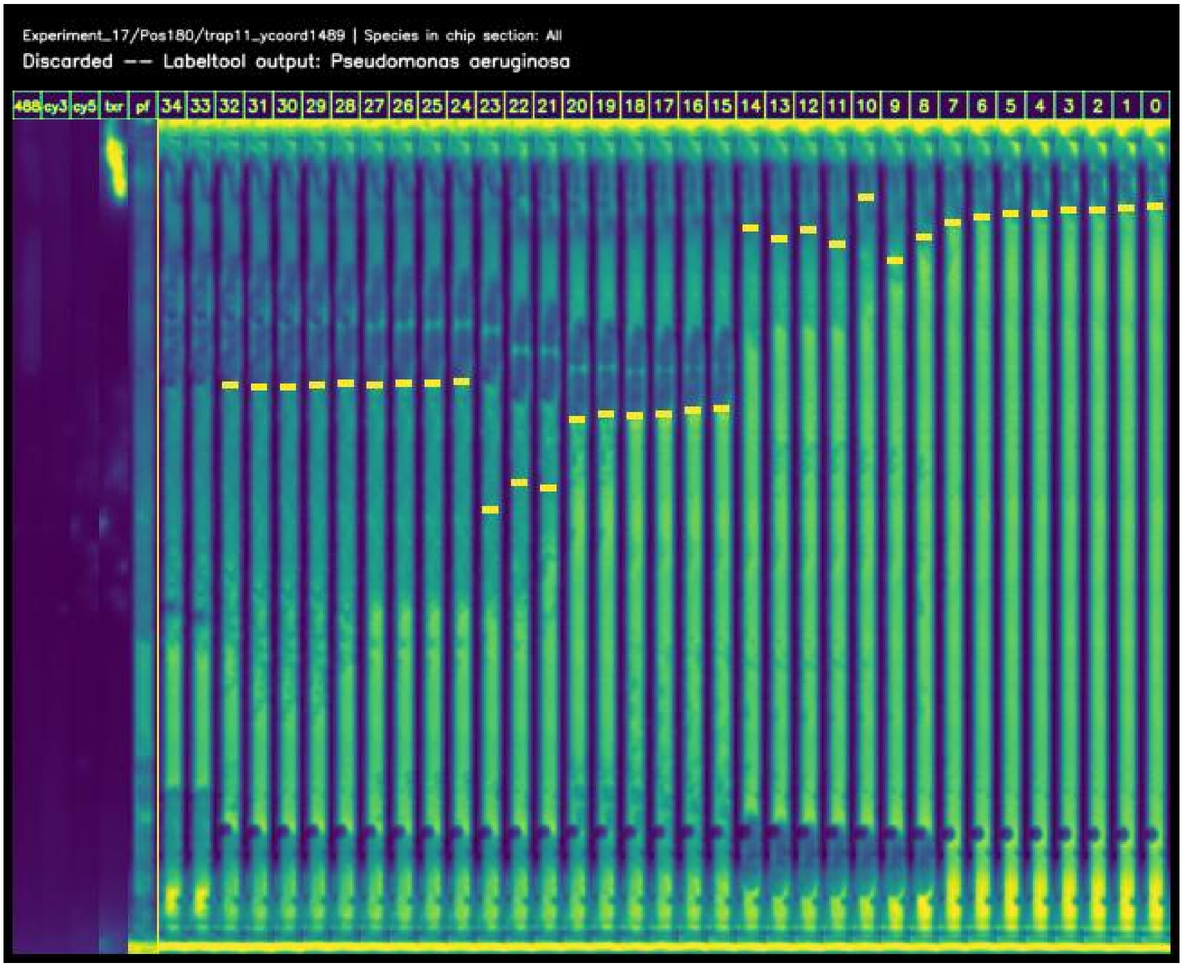
Experiment_17/Pos180/trap11_ycoord1489 | Species in chip section: All
Discarded -- Labeltool output: Pseudomonas aeruginosa
488 cy3 cy5 txr pf 34 33 32 31 30 29 28 27 26 25 24 23 22 21 20 19 18 17 16 15 14 13 12 11 10 9 8 7 6 5 4 3 2 1 0

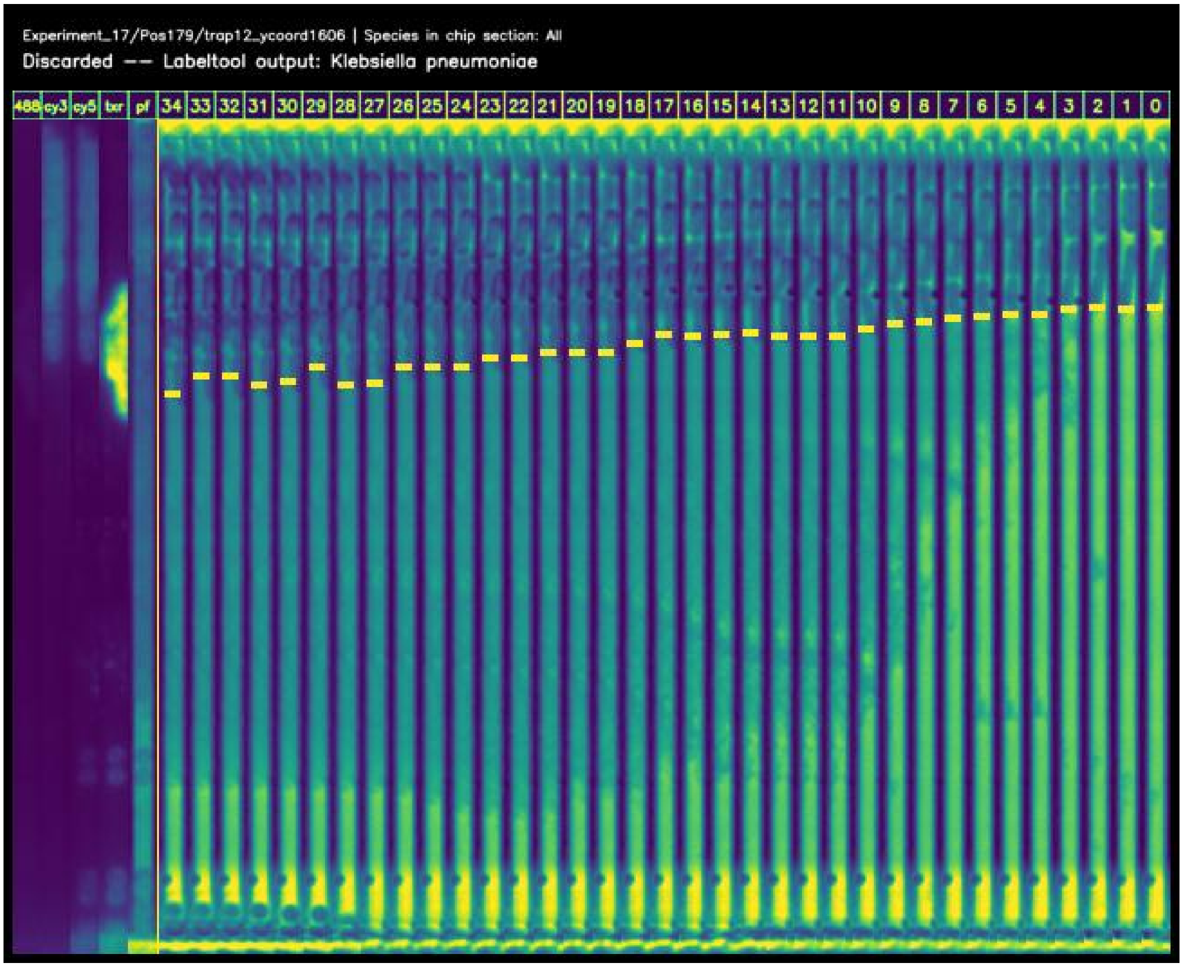
Experiment_17/Pos179/trap12_ycoord1606 | Species in chip section: All
Discarded -- Labeltool output: Klebsiella pneumoniae
488 cy3 cy5 txr pf 34 33 32 31 30 29 28 27 26 25 24 23 22 21 20 19 18 17 16 15 14 13 12 11 10 9 8 7 6 5 4 3 2 1 0

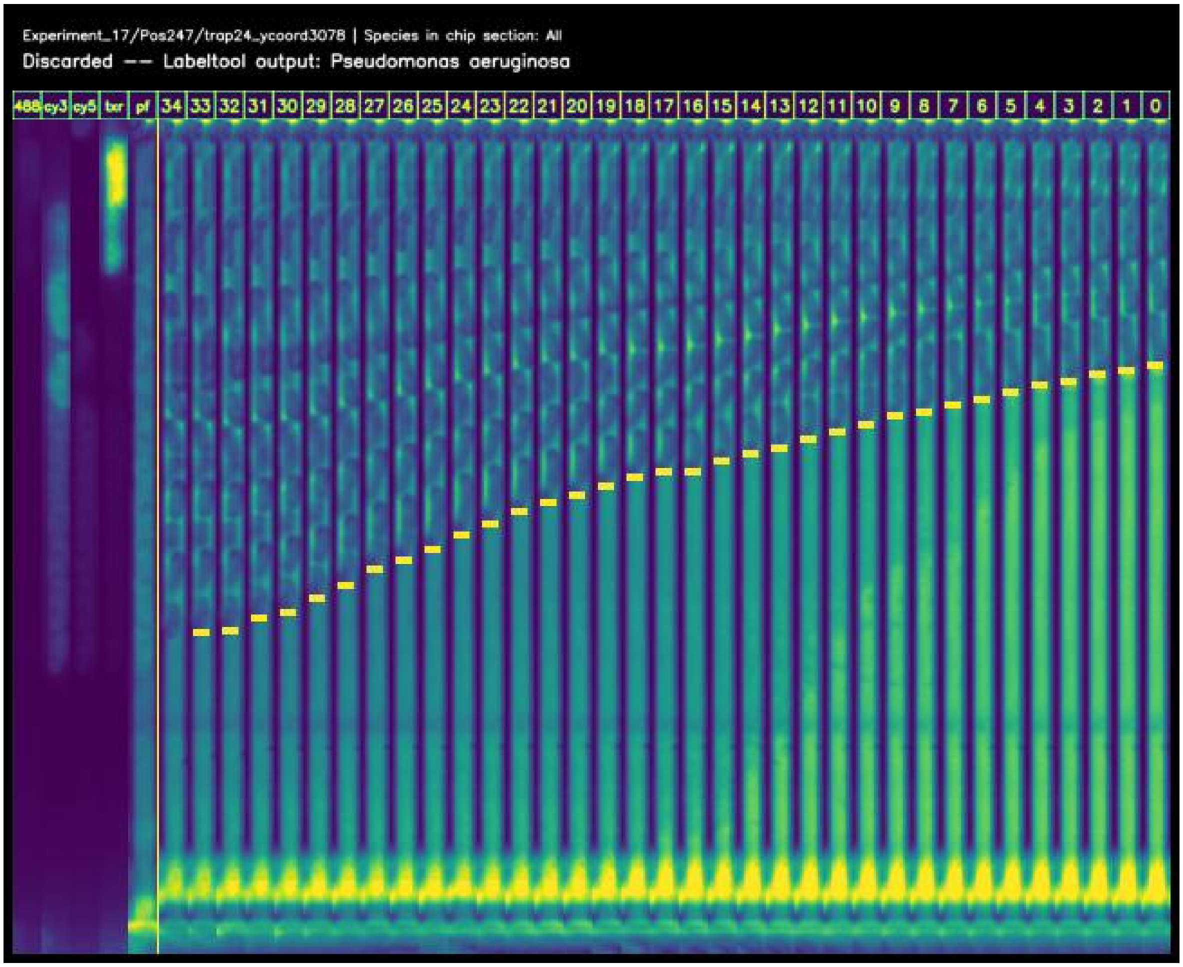
Experiment_17/Pos247/trap24_ycoord3078 | Species in chip section: All
Discarded -- Labeltool output: Pseudomonas aeruginosa
488 cy3 cy5 txr pf 34 33 32 31 30 29 28 27 26 25 24 23 22 21 20 19 18 17 16 15 14 13 12 11 10 9 8 7 6 5 4 3 2 1 0

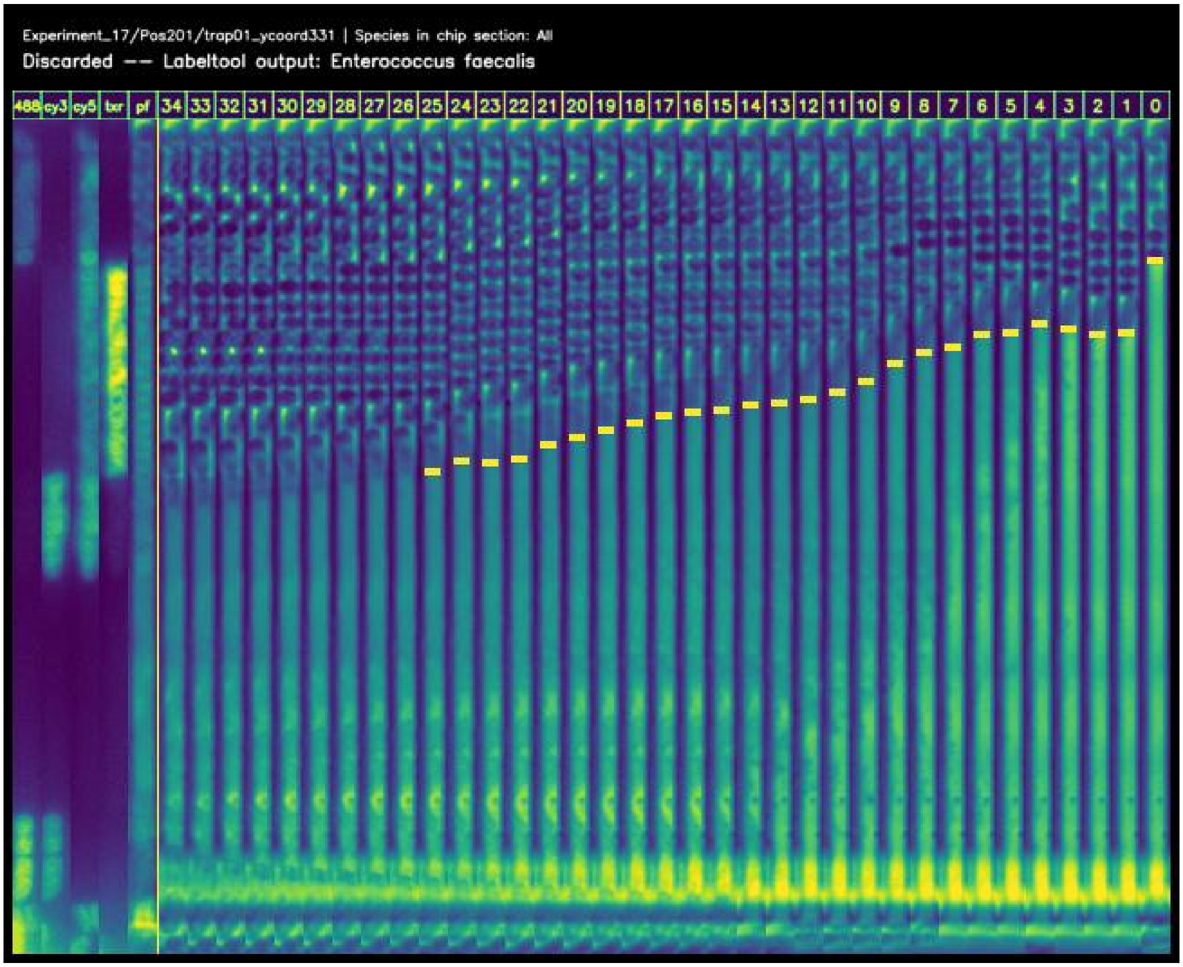
Experiment_17/Pos201/trap01_ycoord331 | Species in chip section: All
Discarded -- Labeltool output: Enterococcus faecalis
488 cy3 cy5 txr pf 34 33 32 31 30 29 28 27 26 25 24 23 22 21 20 19 18 17 16 15 14 13 12 11 10 9 8 7 6 5 4 3 2 1 0

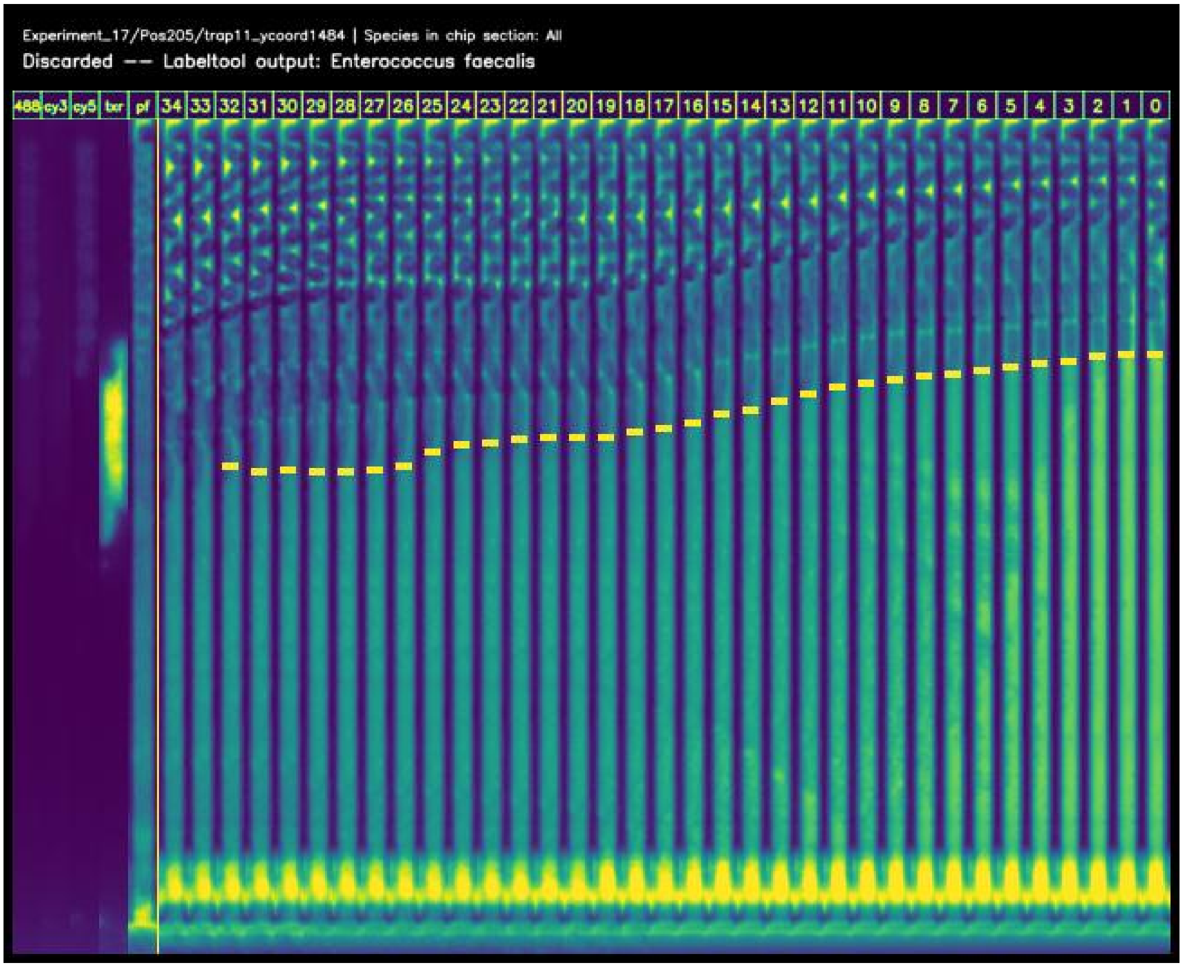
Experiment_17/Pos205/trap11_ycoord1484 | Species in chip section: All
Discarded -- Labeltool output: Enterococcus faecalis
488 cy3 cy5 txr pf 34 33 32 31 30 29 28 27 26 25 24 23 22 21 20 19 18 17 16 15 14 13 12 11 10 9 8 7 6 5 4 3 2 1 0

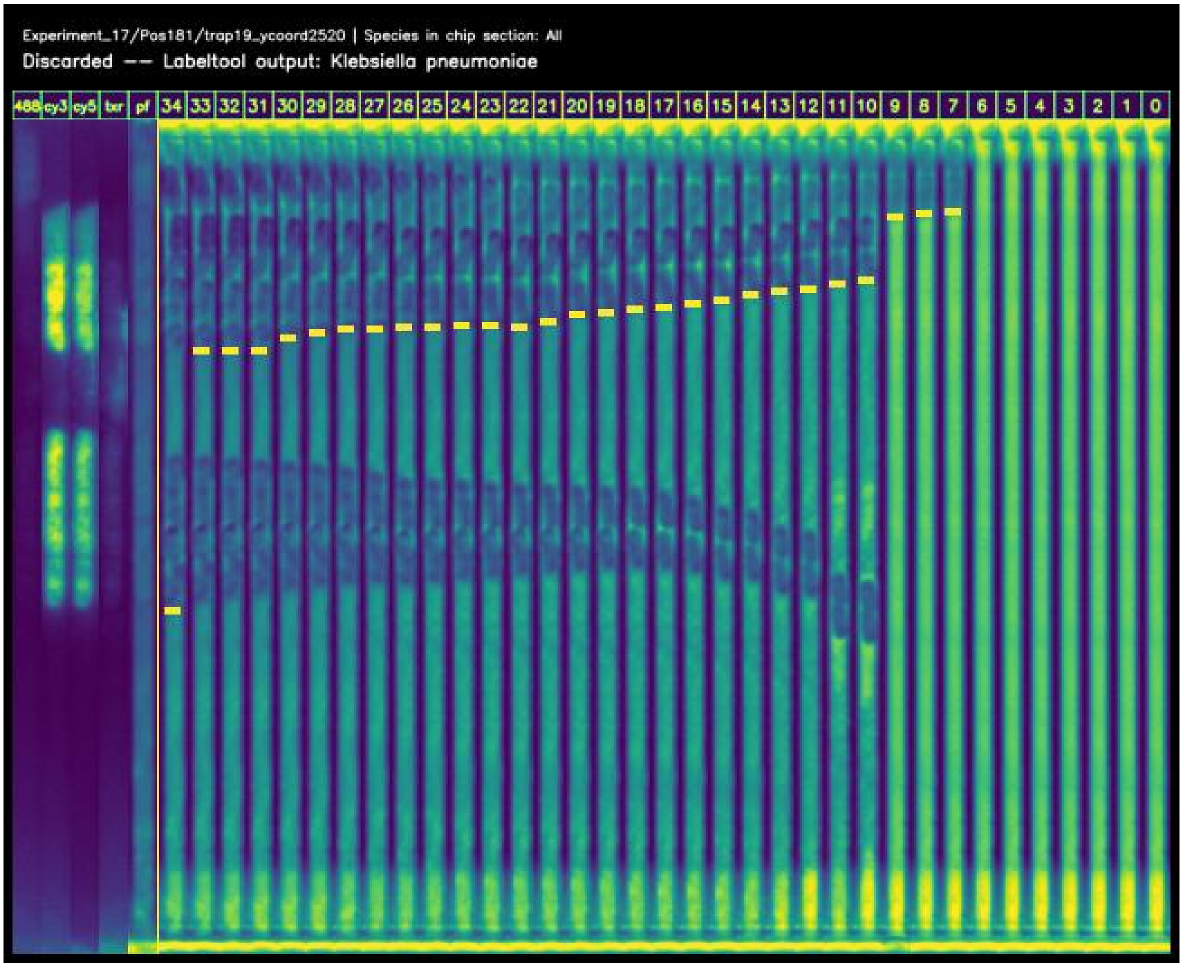
Experiment_17/Pos181/trap19_ycoord2520 | Species in chip section: All
Discarded -- Labeltool output: Klebsiella pneumoniae
488 cy3 cy5 txr pf 34 33 32 31 30 29 28 27 26 25 24 23 22 21 20 19 18 17 16 15 14 13 12 11 10 9 8 7 6 5 4 3 2 1 0

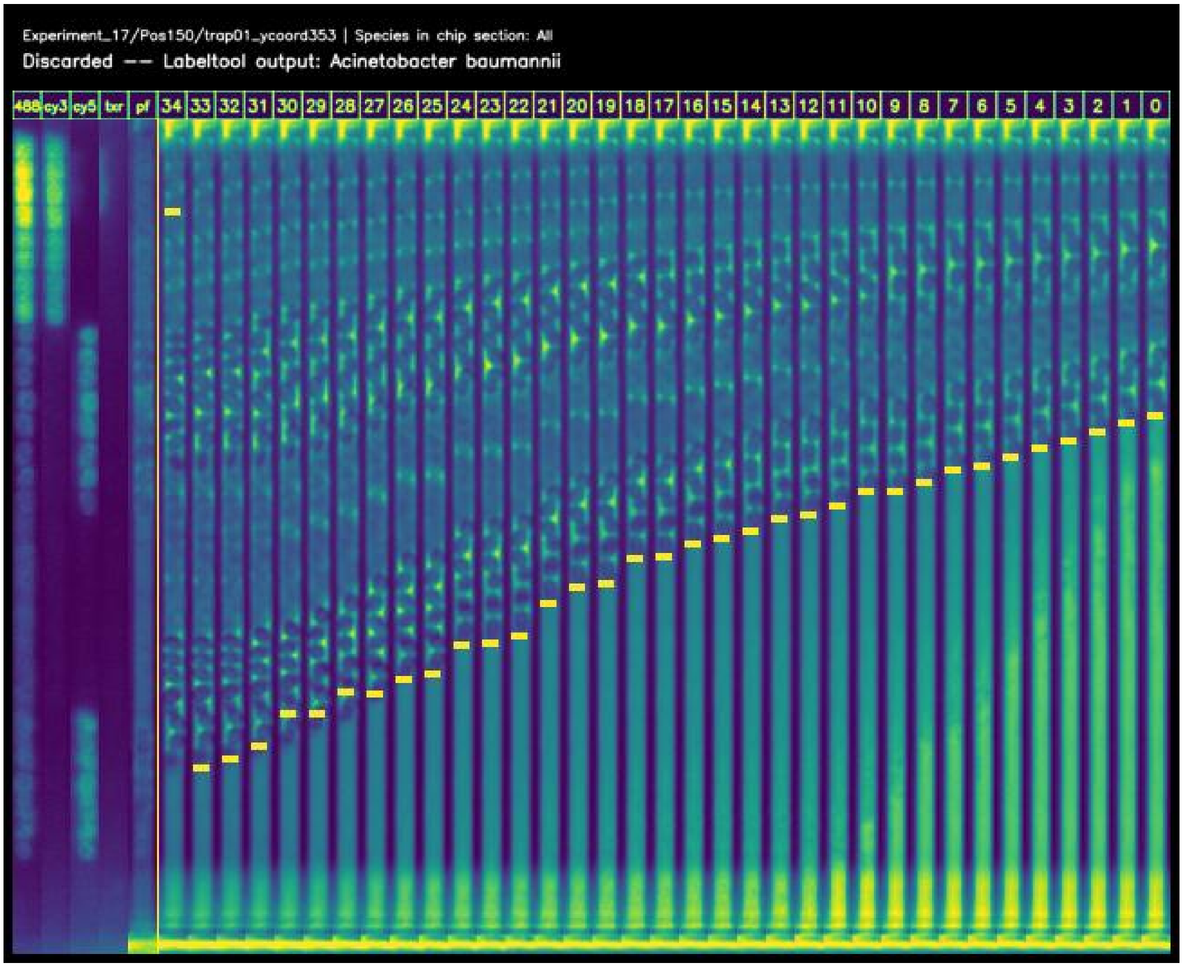
Experiment_17/Pos150/trap01_ycoord353 | Species in chip section: All
Discarded -- Labeltool output: Acinetobacter baumannii
488 cy3 cy5 txr pf 34 33 32 31 30 29 28 27 26 25 24 23 22 21 20 19 18 17 16 15 14 13 12 11 10 9 8 7 6 5 4 3 2 1 0

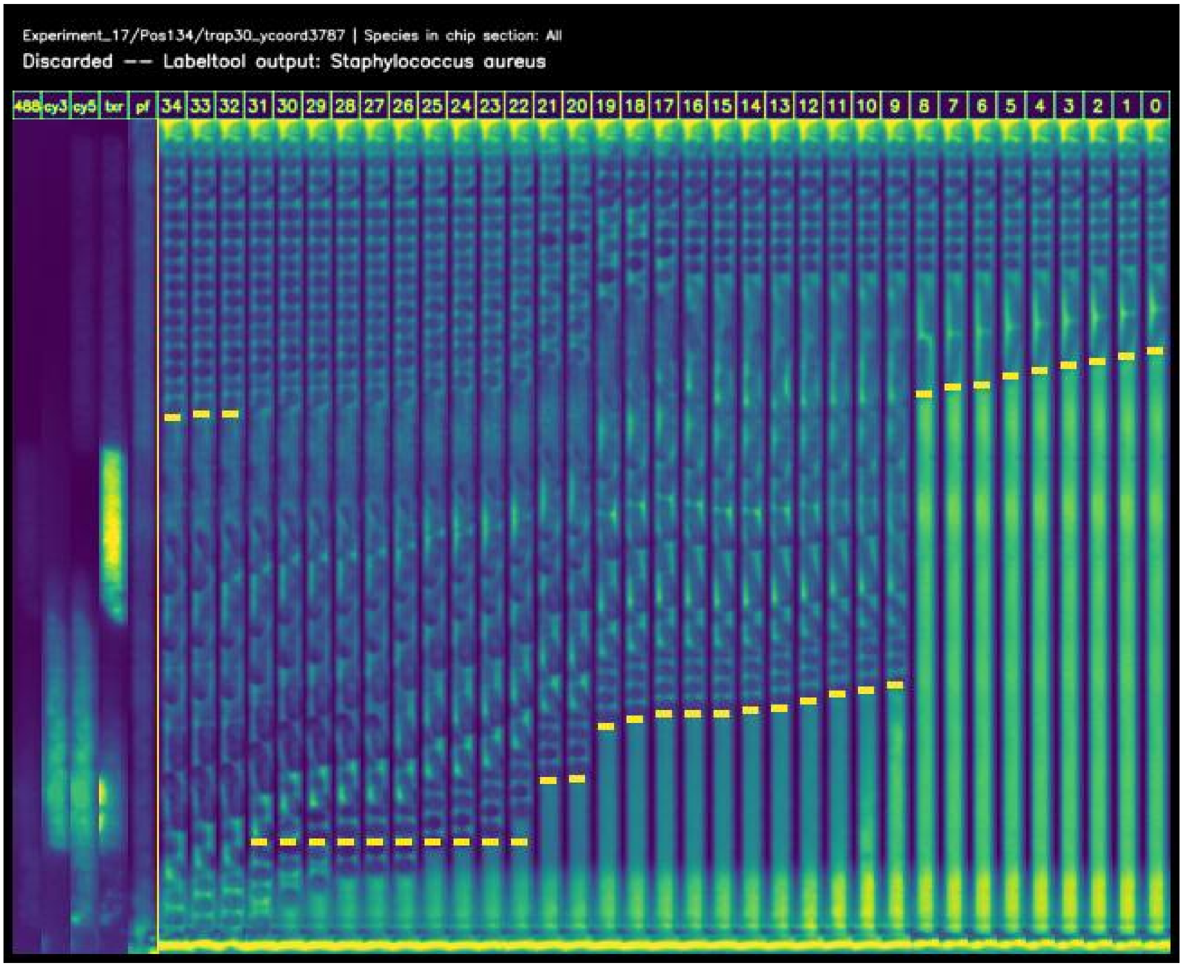
Experiment_17/Pos134/trap30_ycoord3787 | Species in chip section: All
Discarded -- Labeltool output: Staphylococcus aureus
488 cy3 cy5 txr pf 34 33 32 31 30 29 28 27 26 25 24 23 22 21 20 19 18 17 16 15 14 13 12 11 10 9 8 7 6 5 4 3 2 1 0

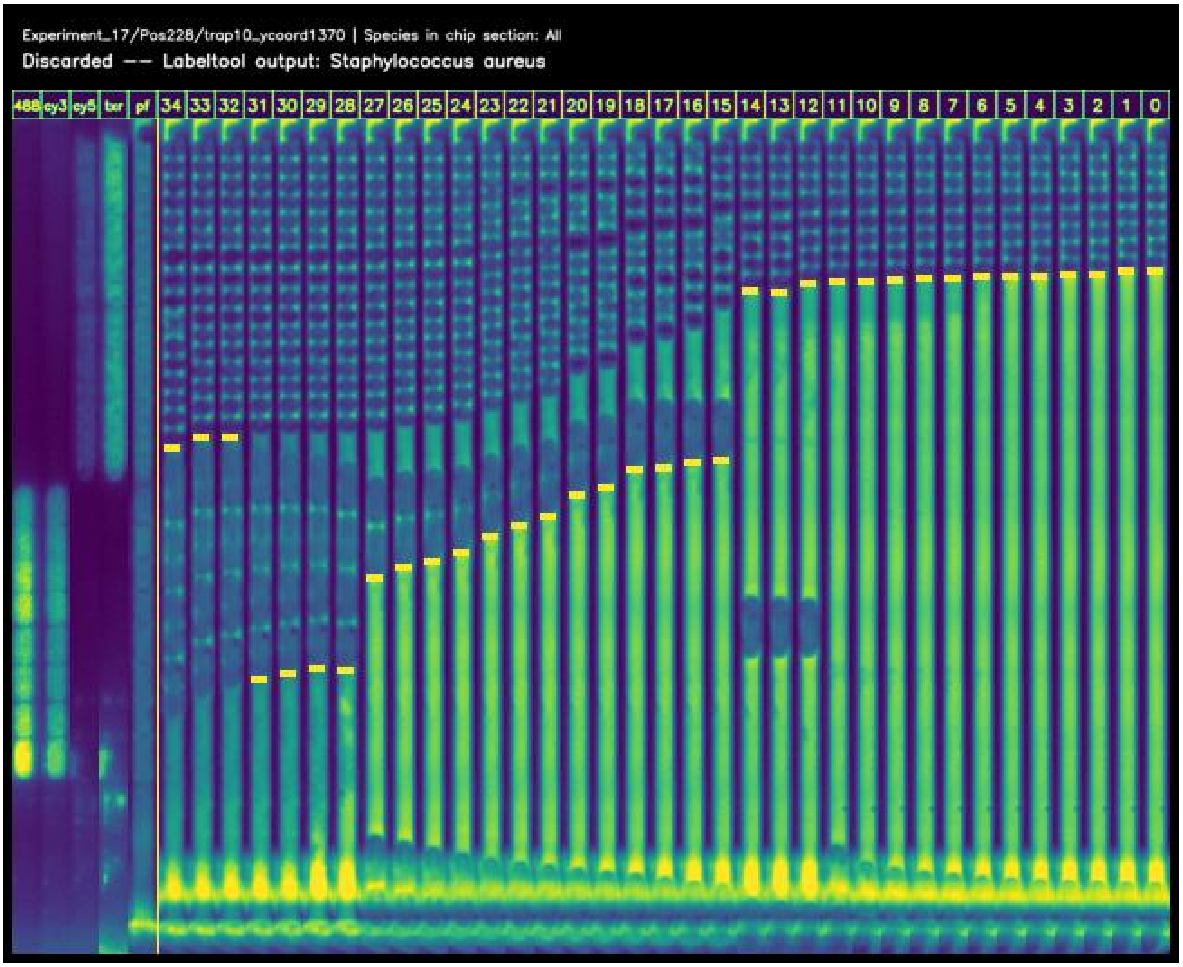
Experiment_17/Pos228/trap10_ycoord1370 | Species in chip section: All
Discarded -- Labeltool output: Staphylococcus aureus
488 cy3 cy5 txr pf 34 33 32 31 30 29 28 27 26 25 24 23 22 21 20 19 18 17 16 15 14 13 12 11 10 9 8 7 6 5 4 3 2 1 0

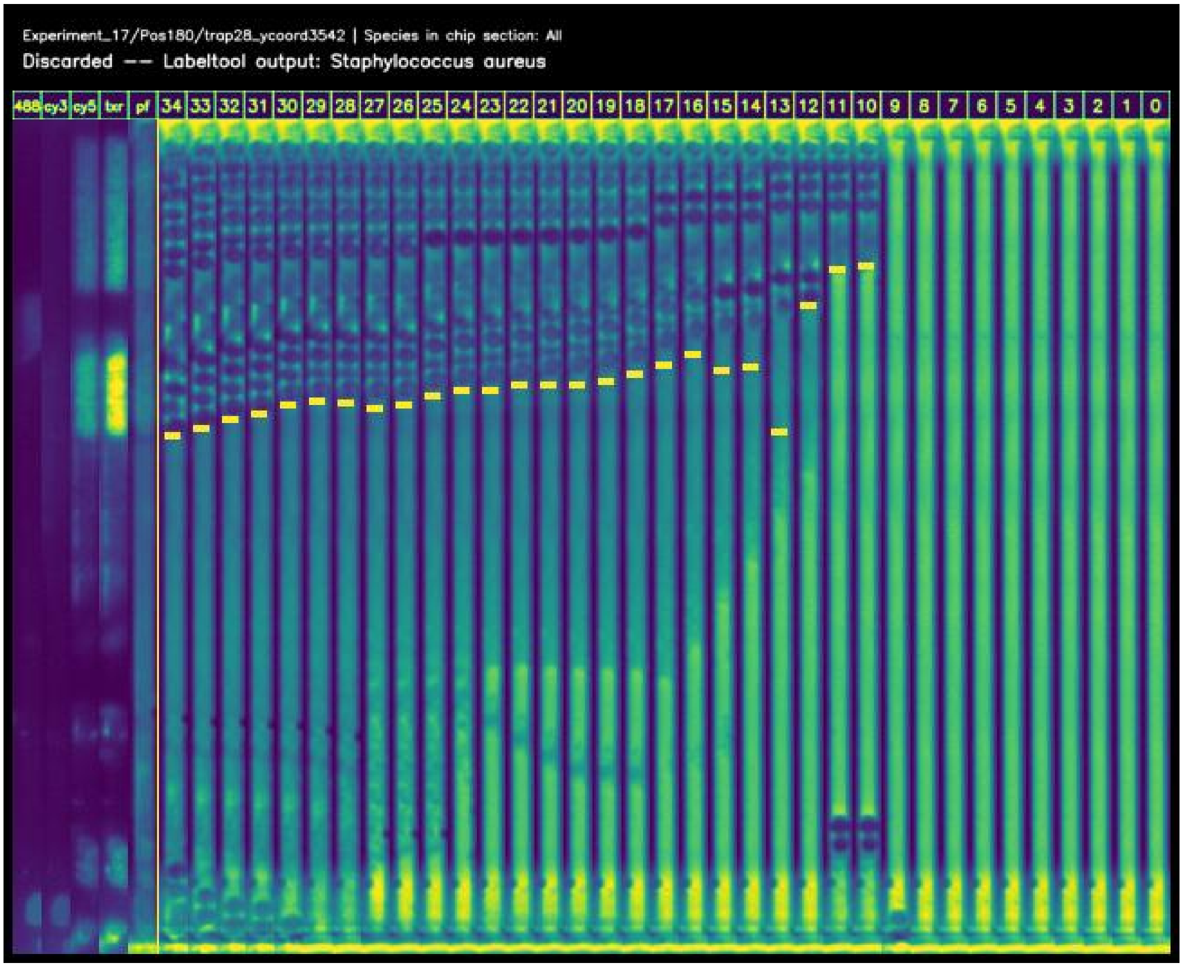
Experiment_17/Pos180/trap28_ycoord3542 | Species in chip section: All
Discarded -- Labeltool output: Staphylococcus aureus
488 cy3 cy5 txr pf 34 33 32 31 30 29 28 27 26 25 24 23 22 21 20 19 18 17 16 15 14 13 12 11 10 9 8 7 6 5 4 3 2 1 0

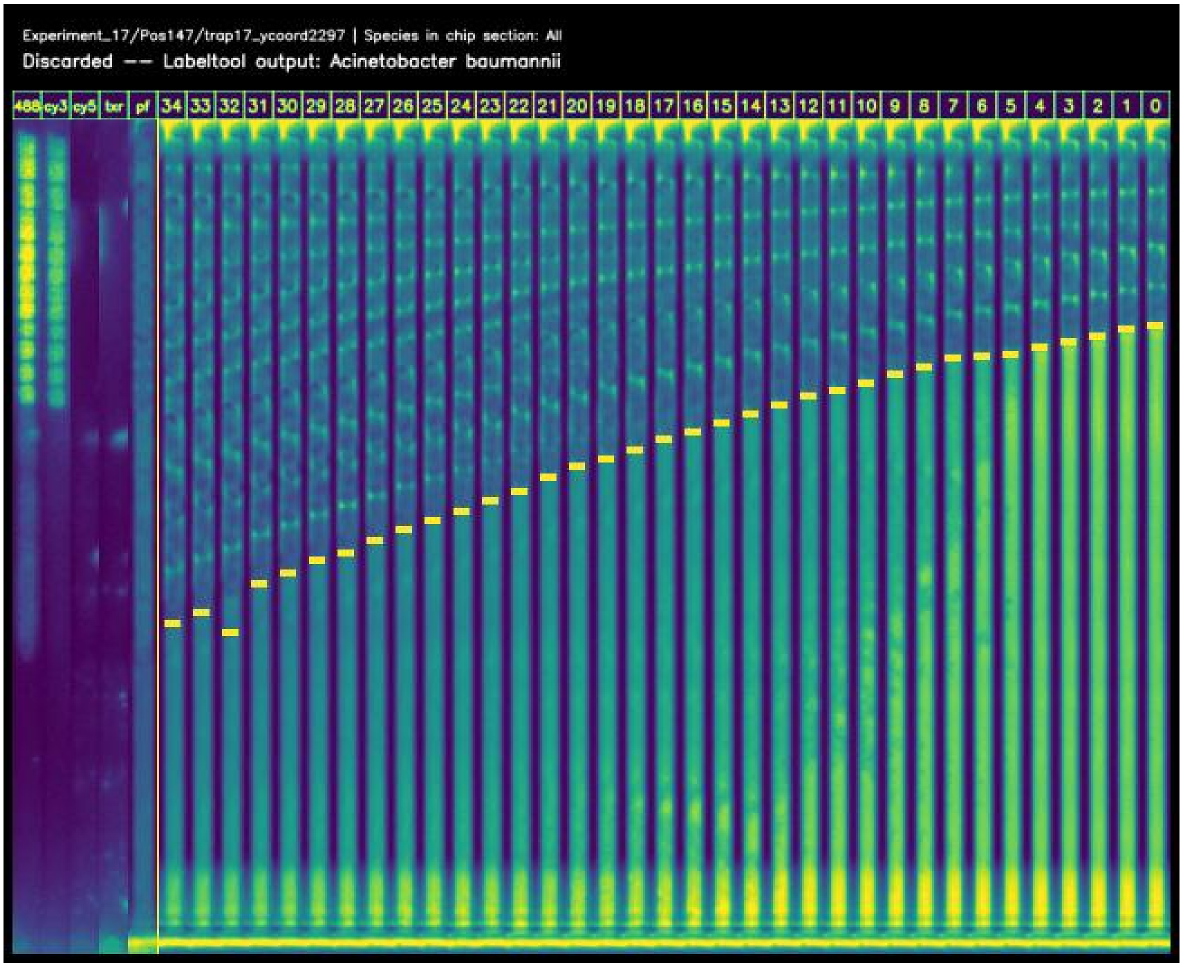
Experiment_17/Pos147/trap17_ycoord2297 | Species in chip section: All
Discarded -- Labeltool output: Acinetobacter baumannii
488 cy3 cy5 txr pf 34 33 32 31 30 29 28 27 26 25 24 23 22 21 20 19 18 17 16 15 14 13 12 11 10 9 8 7 6 5 4 3 2 1 0

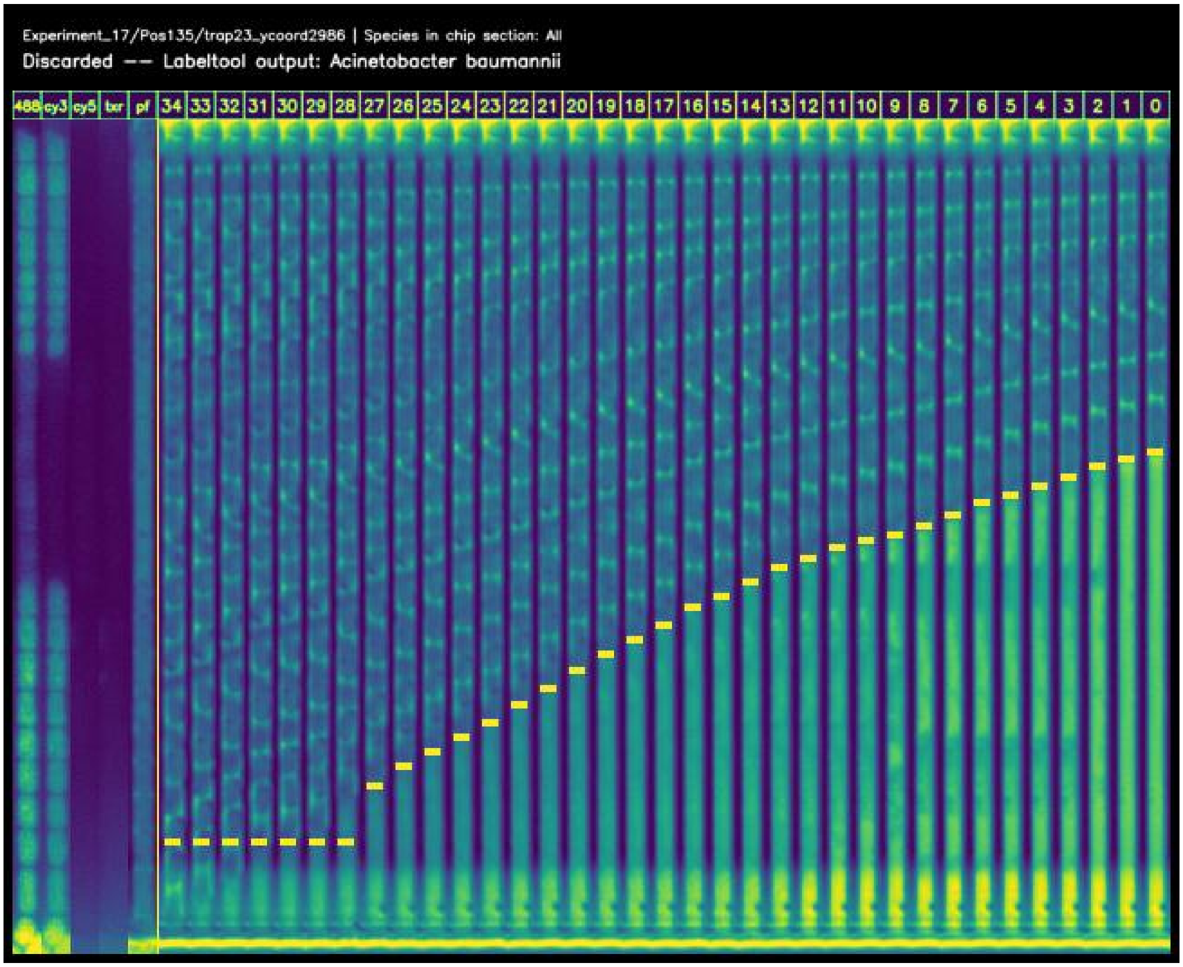
Experiment_17/Pos135/trap23_ycoord2986 | Species in chip section: All
Discarded -- Labeltool output: Acinetobacter baumannii
488 cy3 cy5 txr pf 34 33 32 31 30 29 28 27 26 25 24 23 22 21 20 19 18 17 16 15 14 13 12 11 10 9 8 7 6 5 4 3 2 1 0

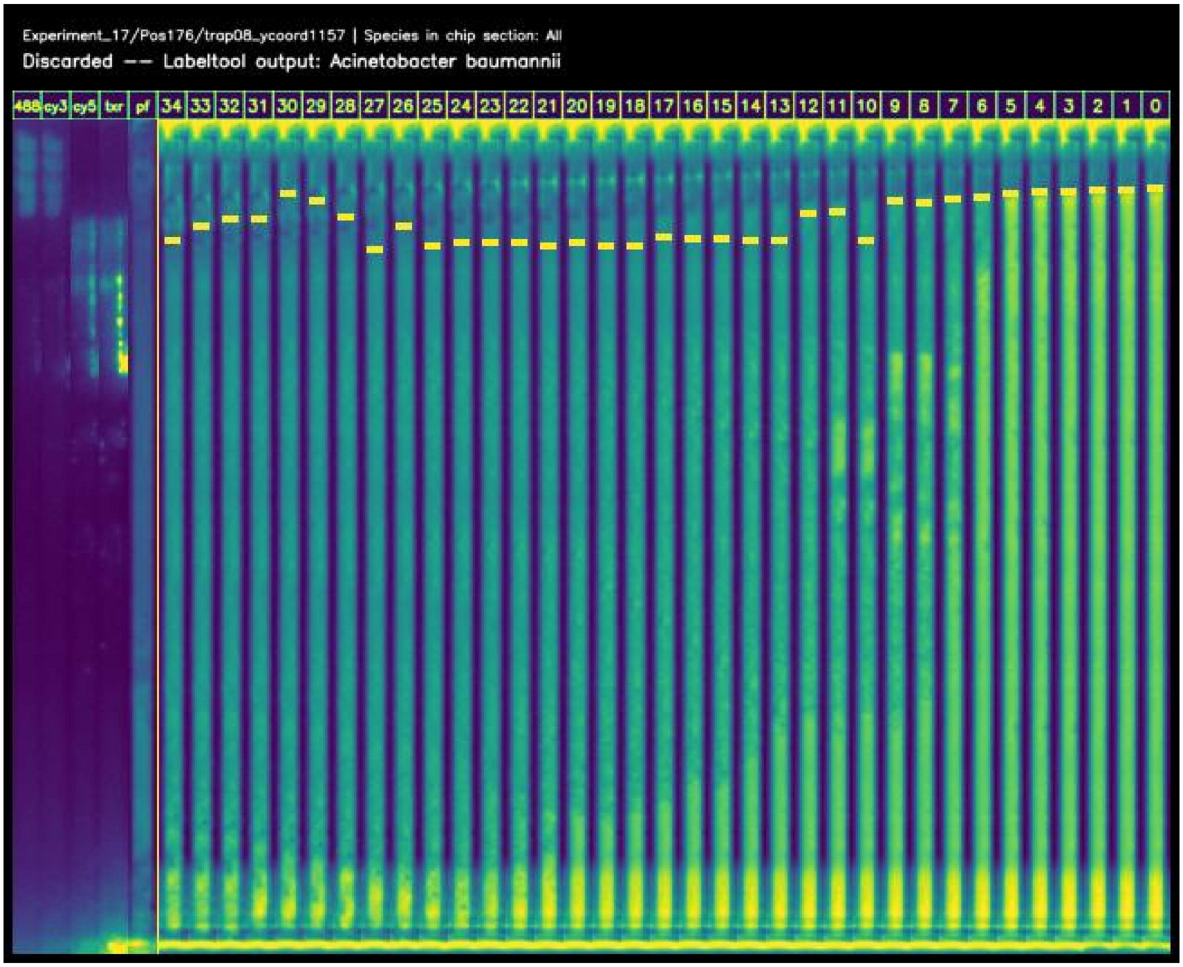

Experiment_17/Pos176/trap08_ycoord1157 | Species in chip section: All
Discarded -- Labeltool output: Acinetobacter baumannii
488 cy3 cy5 txr pf 34 33 32 31 30 29 28 27 26 25 24 23 22 21 20 19 18 17 16 15 14 13 12 11 10 9 8 7 6 5 4 3 2 1 0

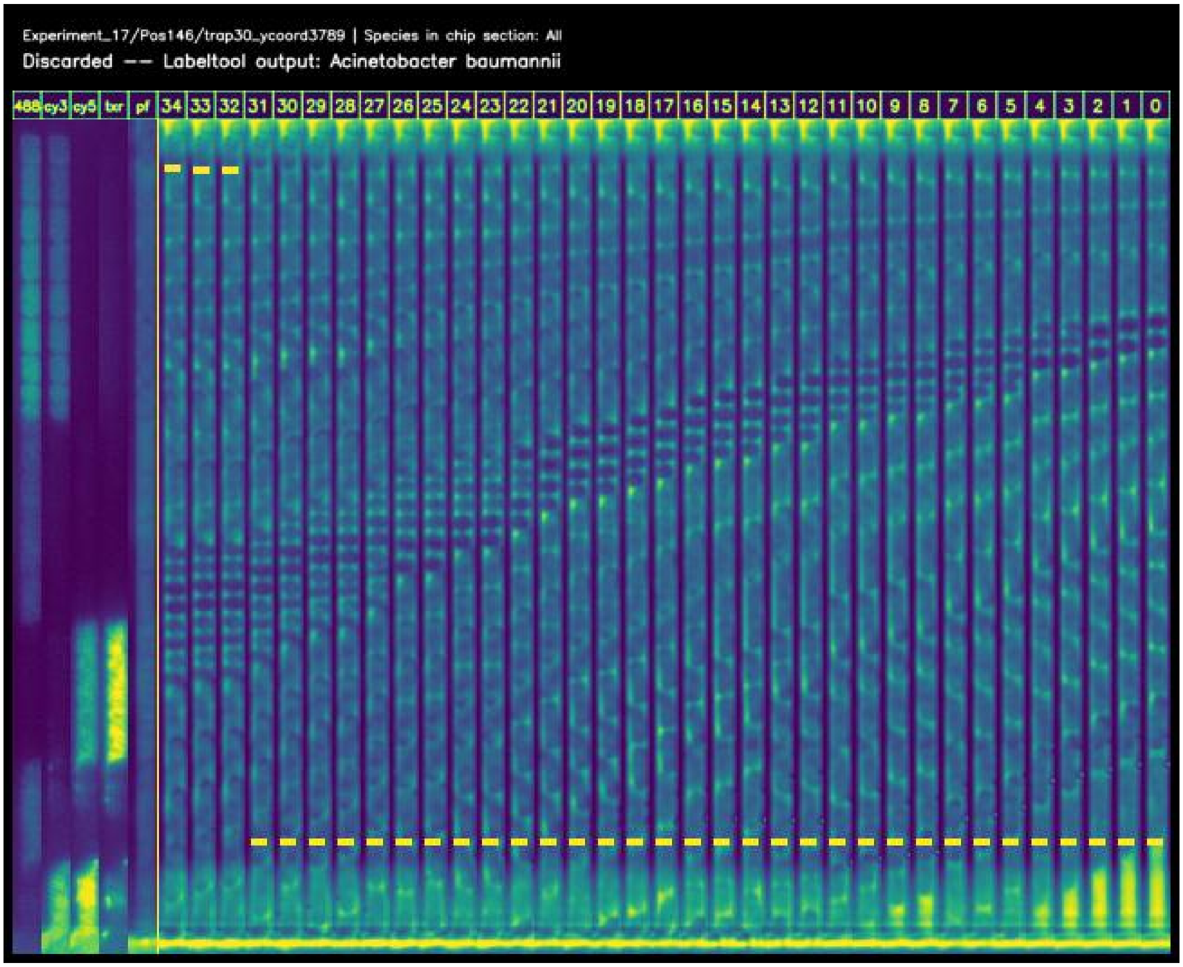

Experiment_17/Pos146/trap30_ycoord3789 | Species in chip section: All
Discarded -- Labeltool output: Acinetobacter baumannii
488 cy3 cy5 txr pf 34 33 32 31 30 29 28 27 26 25 24 23 22 21 20 19 18 17 16 15 14 13 12 11 10 9 8 7 6 5 4 3 2 1 0

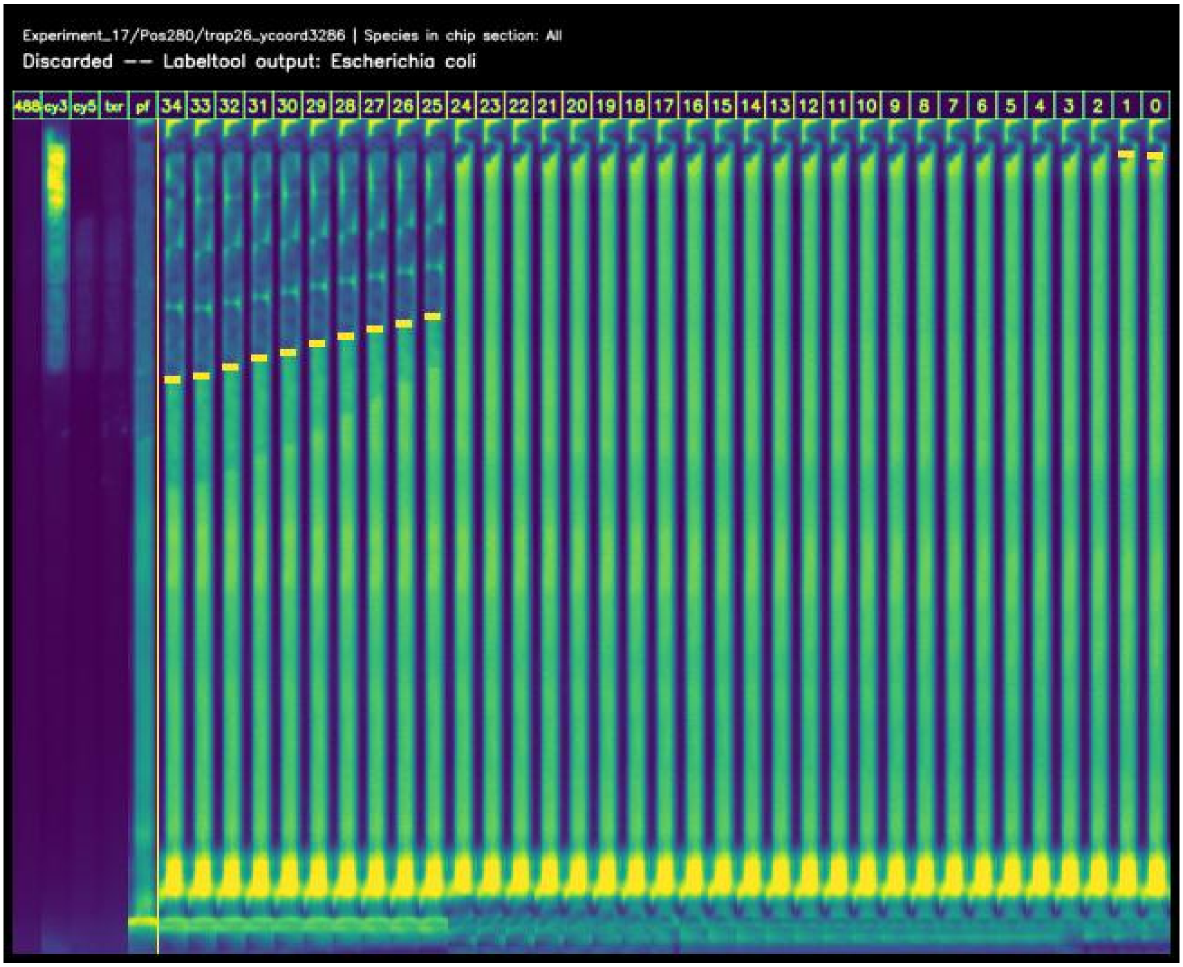
Experiment_17/Pos280/trap26_ycoord3286 | Species in chip section: All
Discarded -- Labeltool output: Escherichia coli
488 cy3 cy5 txr pf 34 33 32 31 30 29 28 27 26 25 24 23 22 21 20 19 18 17 16 15 14 13 12 11 10 9 8 7 6 5 4 3 2 1 0

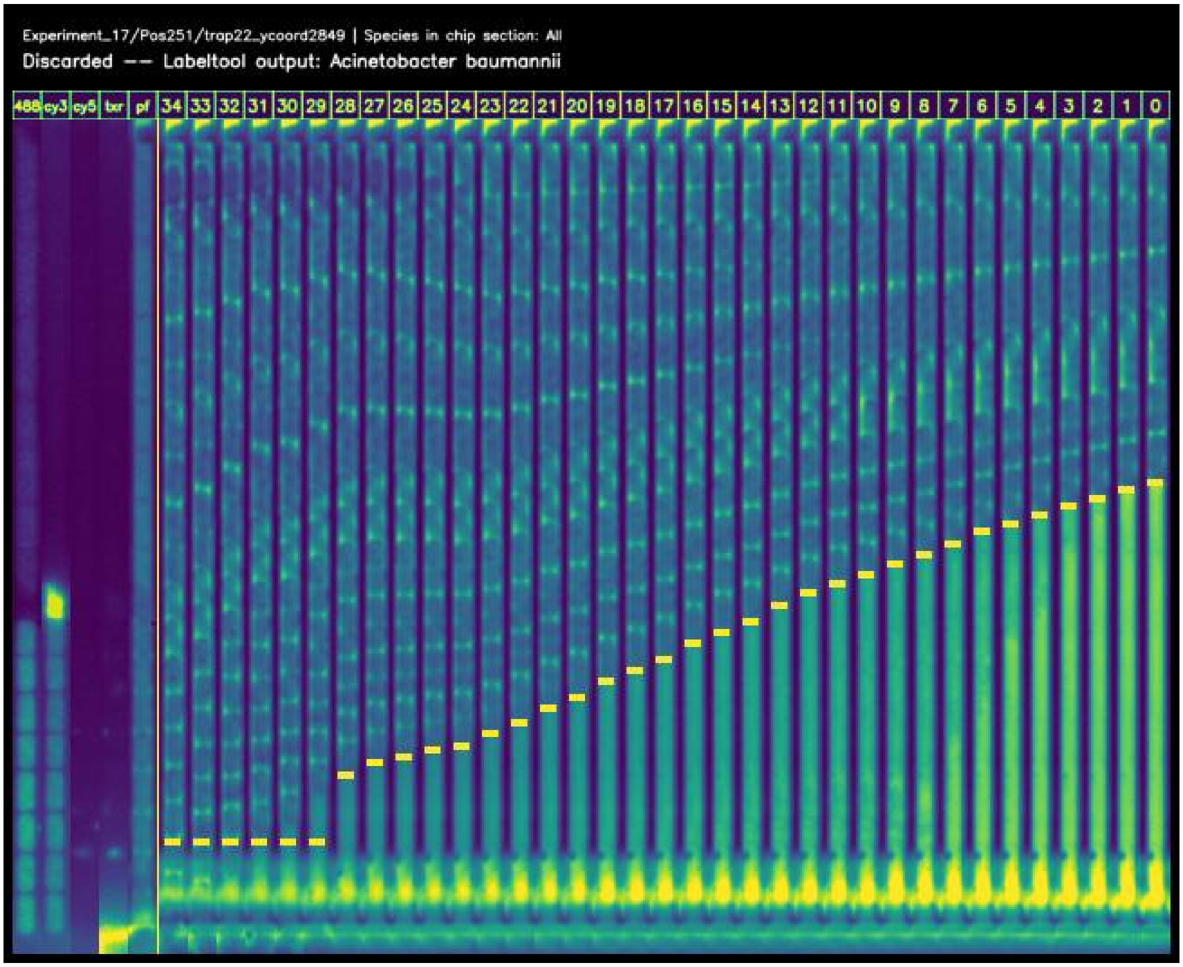
Experiment_17/Pos251/trap22_ycoord2849 | Species in chip section: All
Discarded -- Labeltool output: Acinetobacter baumannii
488 cy3 cy5 txr pf 34 33 32 31 30 29 28 27 26 25 24 23 22 21 20 19 18 17 16 15 14 13 12 11 10 9 8 7 6 5 4 3 2 1 0
