# Supplementary figures and images for: Rapid label-free identification of seven bacterial species using microfluidics, single-cell time-lapse phase-contrast microscopy, and deep learning-based image and video classification
Source: PLoS One. 2025 Sep 8;20(9):e0330265. doi: 10.1371/journal.pone.0330265 (PMC12416834; doi:10.1371/journal.pone.0330265)

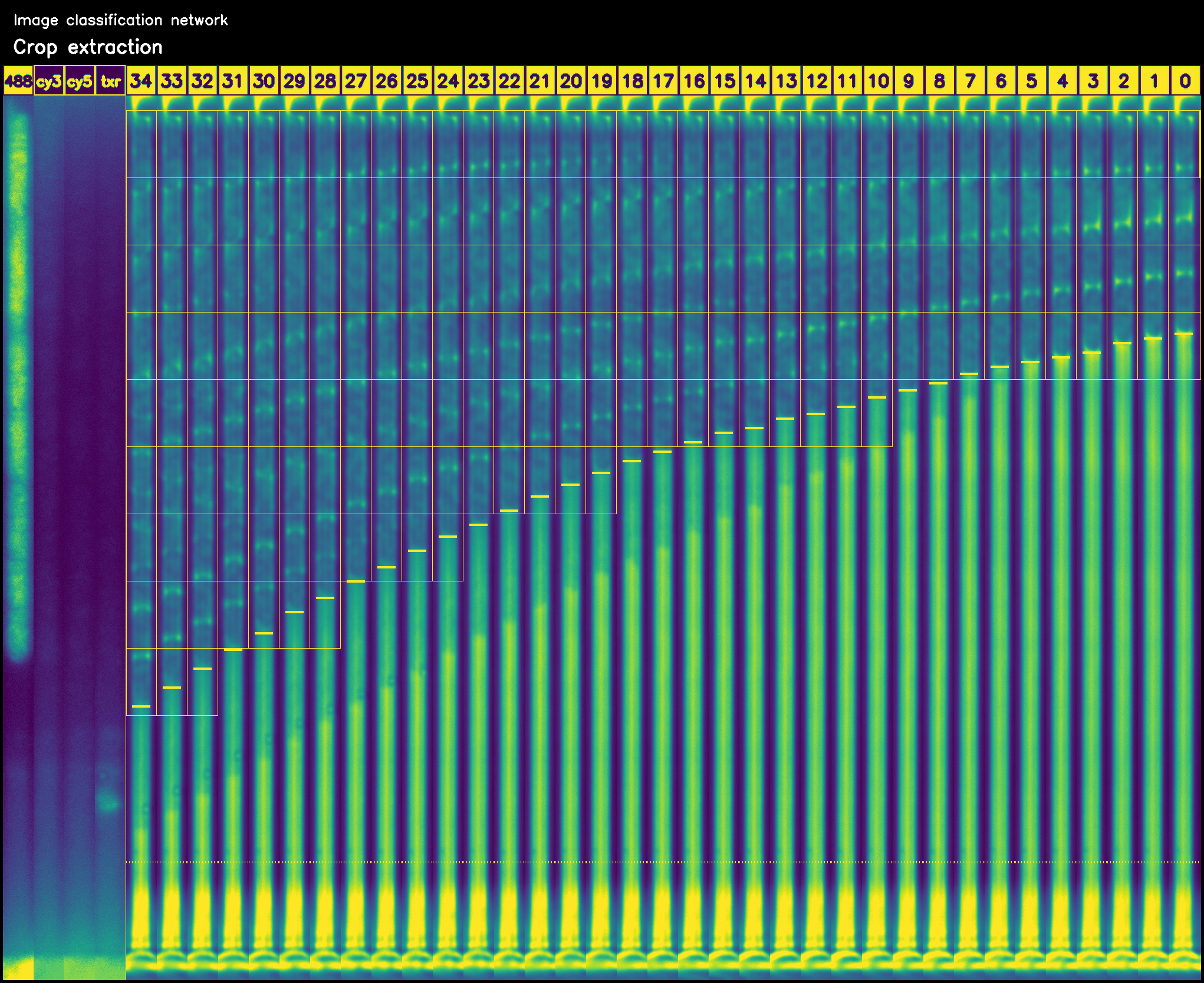

Supplement: S1 Fig — The vertical growth extent of the bacteria in each trap was measured using the segmentation model, indicated by the horizontal markings on the tip of the lowest cell in each trap. Crops were extracted in a tiling-window fashion, shown by outlined yellow squares. In total, 202 52x114 pixel crops were extracted from this trap. The criteria for including a crop is that at least 25% of its vertical extent contains bacteria. (PNG) [file pone.0330265.s002.png]

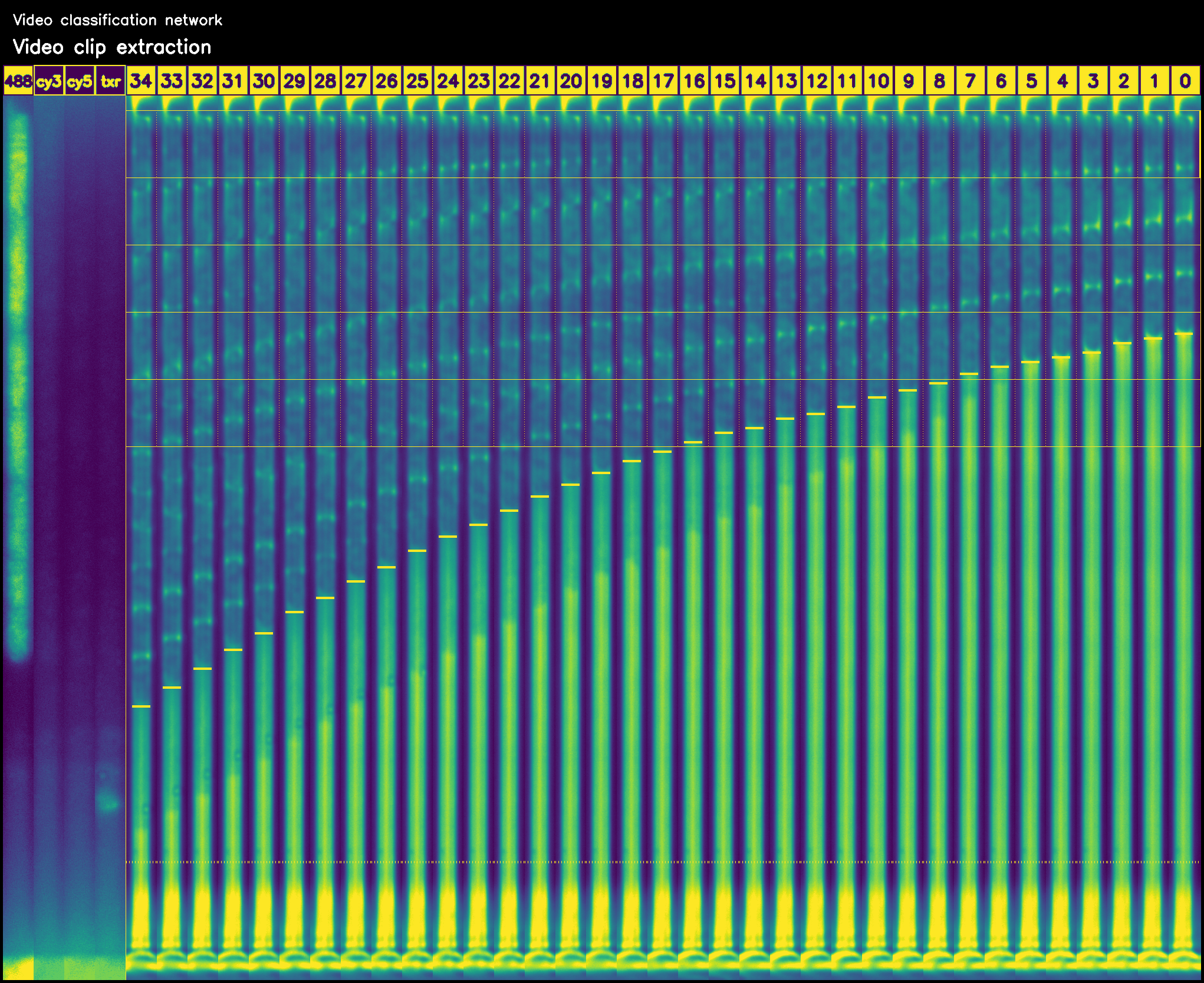

Supplement: S2 Fig — The vertical growth extent of the bacteria in each trap was measured using the segmentation model, indicated by the horizontal markings on the tip of the lowest cell in each trap. Each video crop is outlined with a yellow square. The vertical dotted lines between adjacent frames indicate they are part of the same video crop. In total, five 52x114x35 pixel video crops were extracted from this trap. The criteria for including a video crop is that at least 75% of the frames contain bacterial content. (PNG) [file pone.0330265.s003.png]

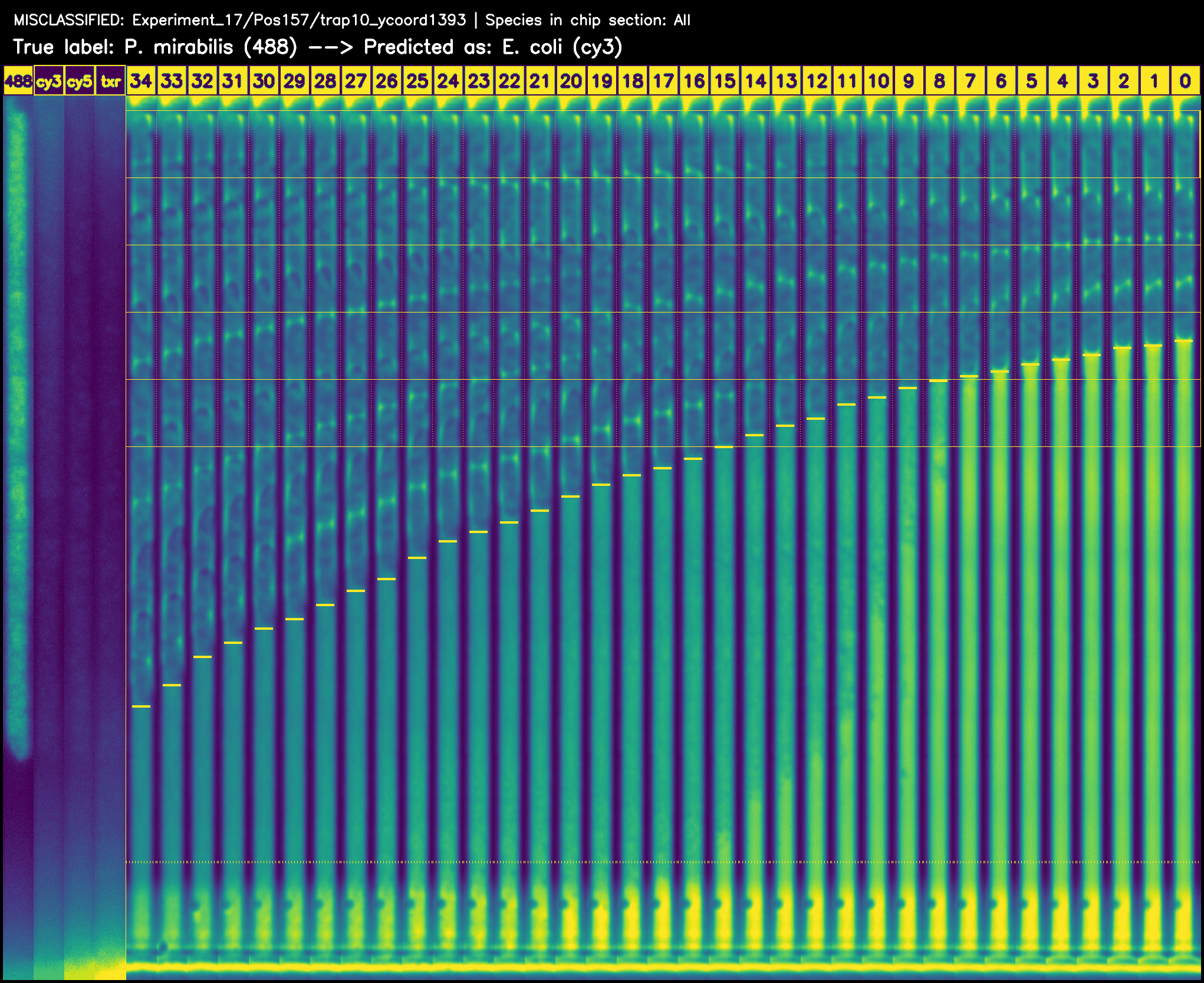

Supplement: S3 Fig — E. coli and P. mirabilis are visually very similar in shape, both being rods. There is some overcrowding in the trap with overlapping cells. (PNG) [file pone.0330265.s004.png]

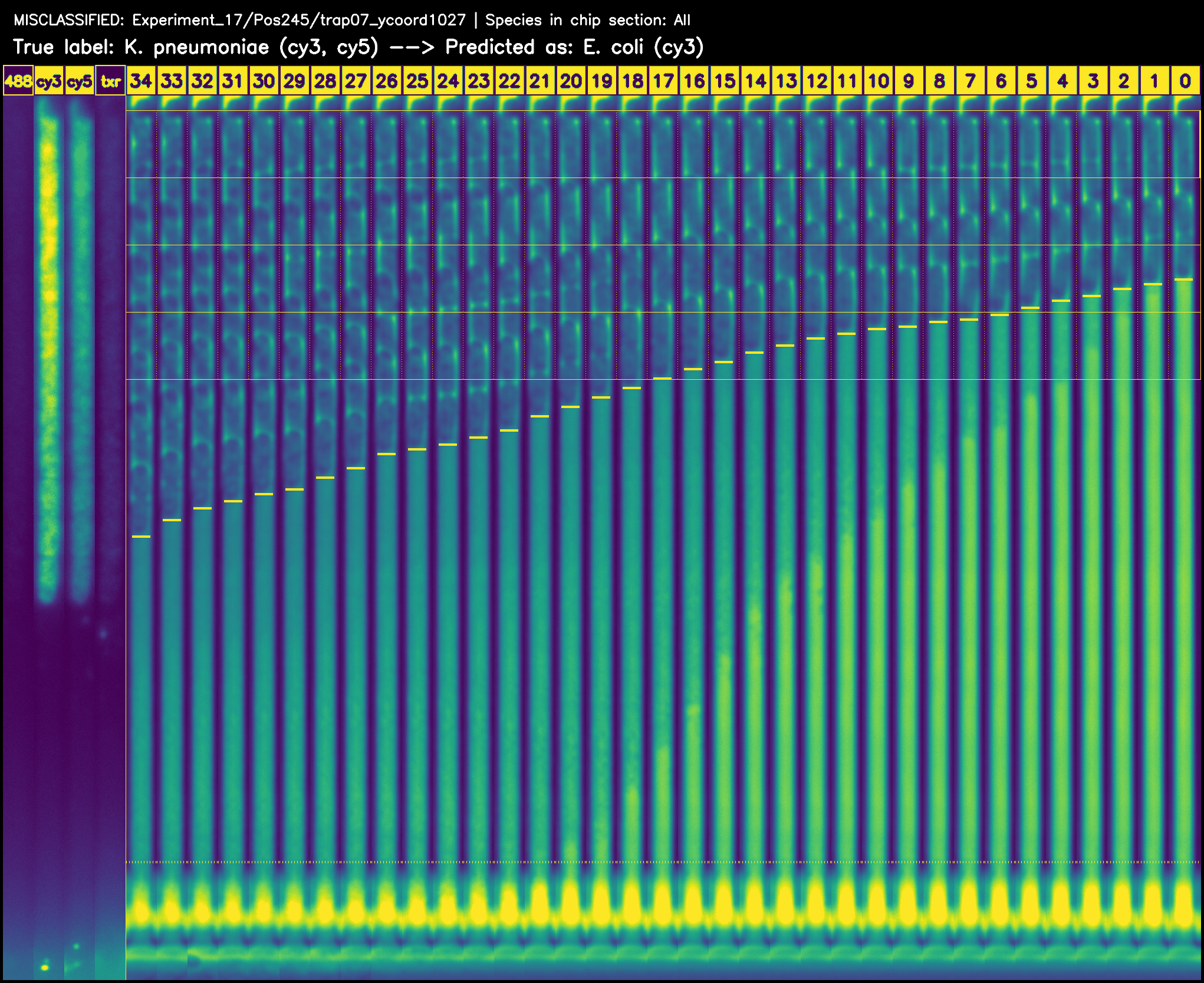

Supplement: S4 Fig — E. coli and K. pneumoniae are visually very similar in shape, both being rods. There is some overcrowding in the trap with overlapping cells at the end of the time-lapse. (PNG) [file pone.0330265.s005.png]

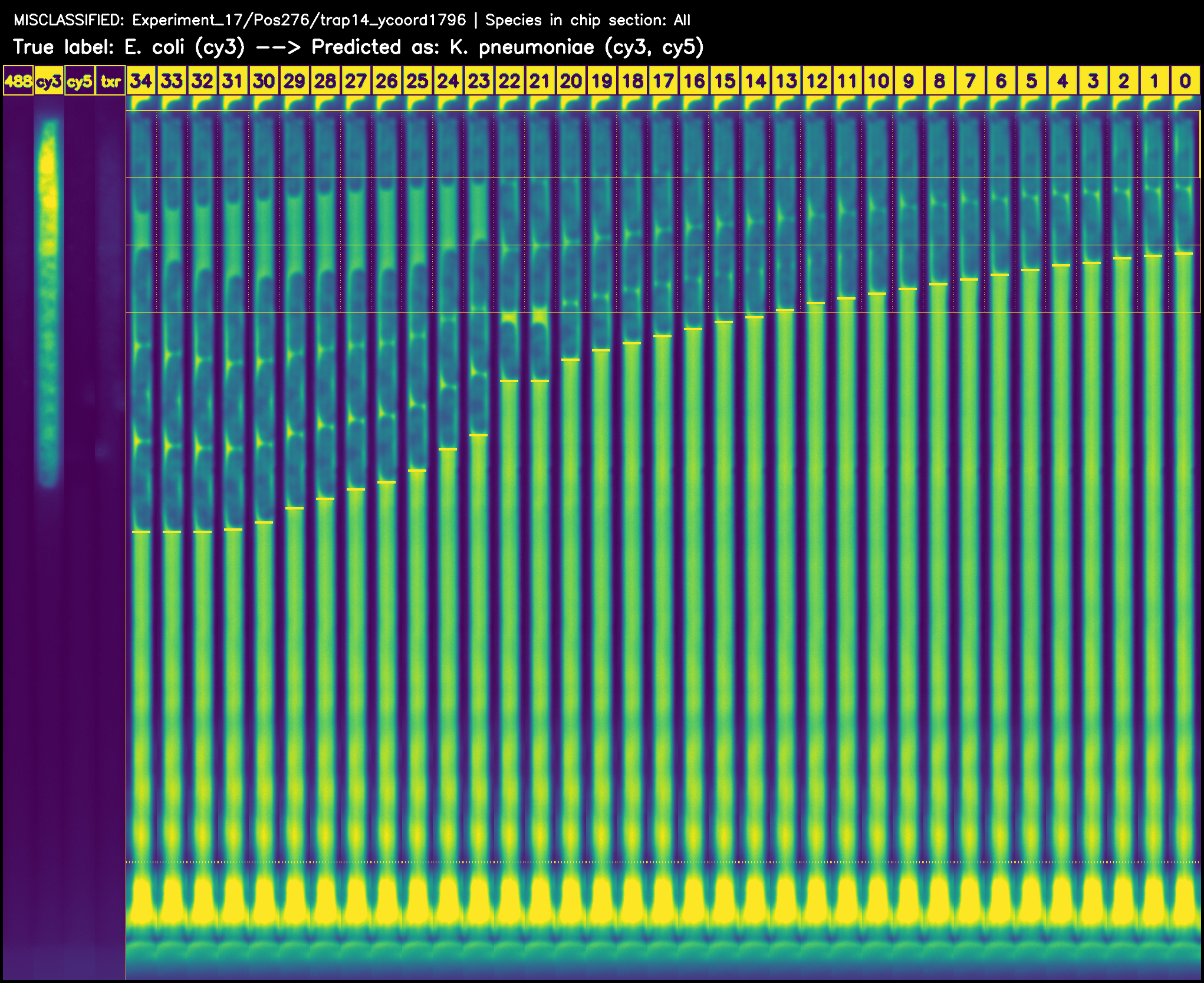

Supplement: S5 Fig — K. penumaniae and E. coli are visually very similar in shape, both being rods. There is some dislocation in the trap at the end of the time-lapse, causing one of the video-crops to have empty frames. (PNG) [file pone.0330265.s006.png]

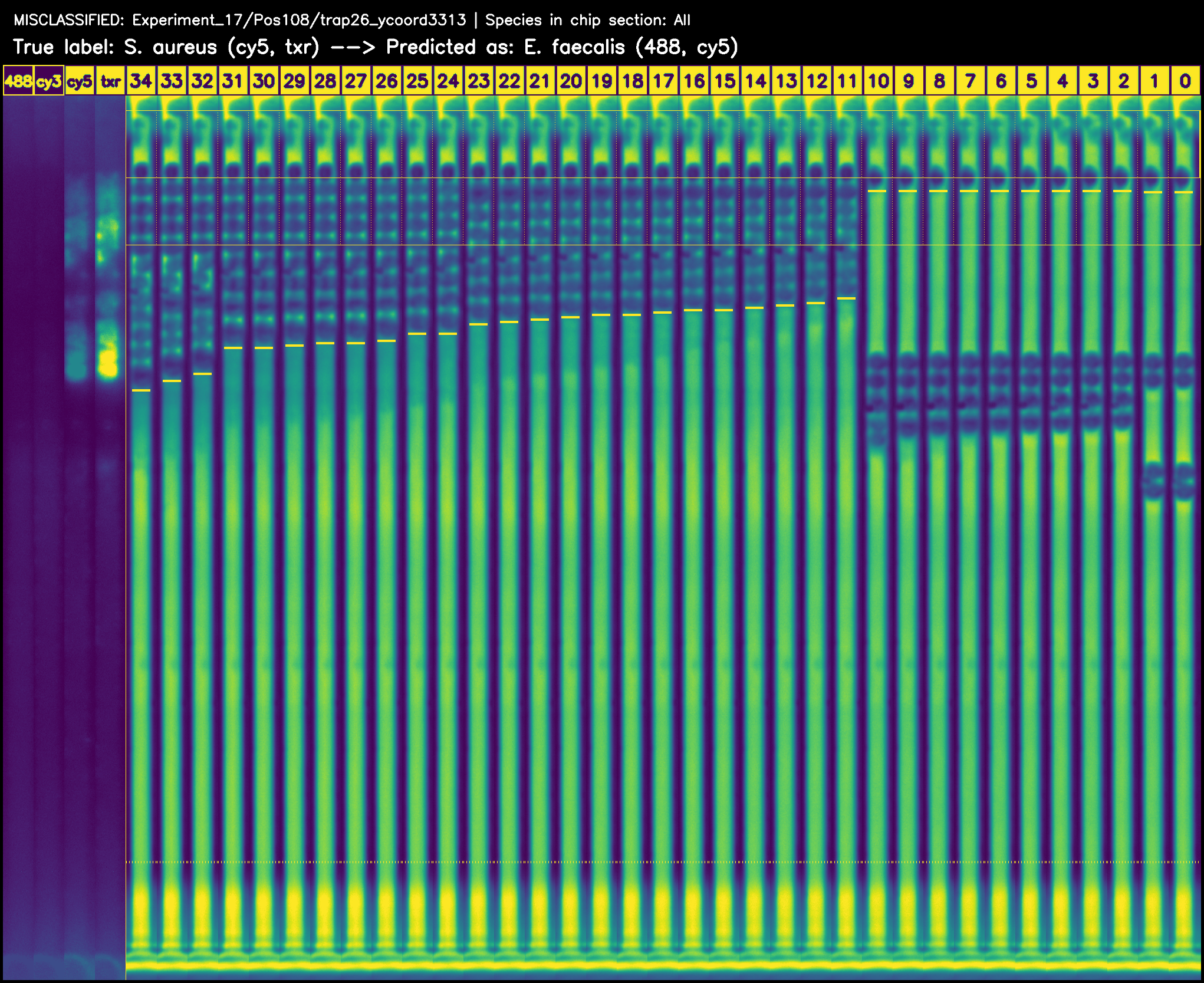

Supplement: S6 Fig — S. aureus and E. faecalis are visually very similar in shape, both being cocci. There is a dislocation in the top, one of the video crops has only empty frames. This trap should have been discarded according to the discarding criteria (no dislocation from the top of the trap), but it was missed during the test set inspection. (PNG) [file pone.0330265.s007.png]

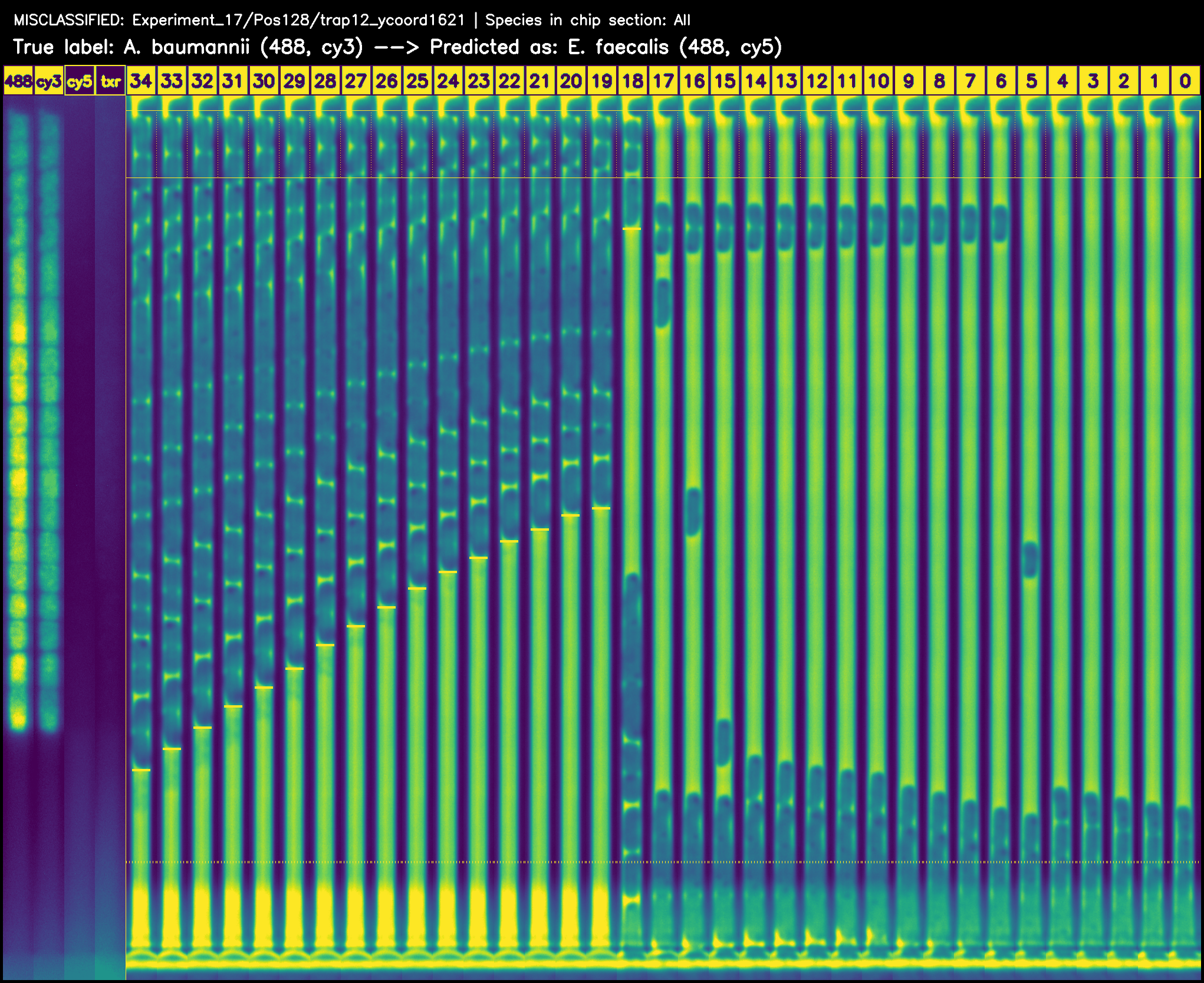

Supplement: S7 Fig — The model mistakes the circular physical stop at the top of the trap for E. faecalis. This trap should have been discarded according to the discarding criteria since the trap is empty up to frame 15 (cells need to be loaded within 30 minutes), but it was missed during the test set inspection. (PNG) [file pone.0330265.s008.png]

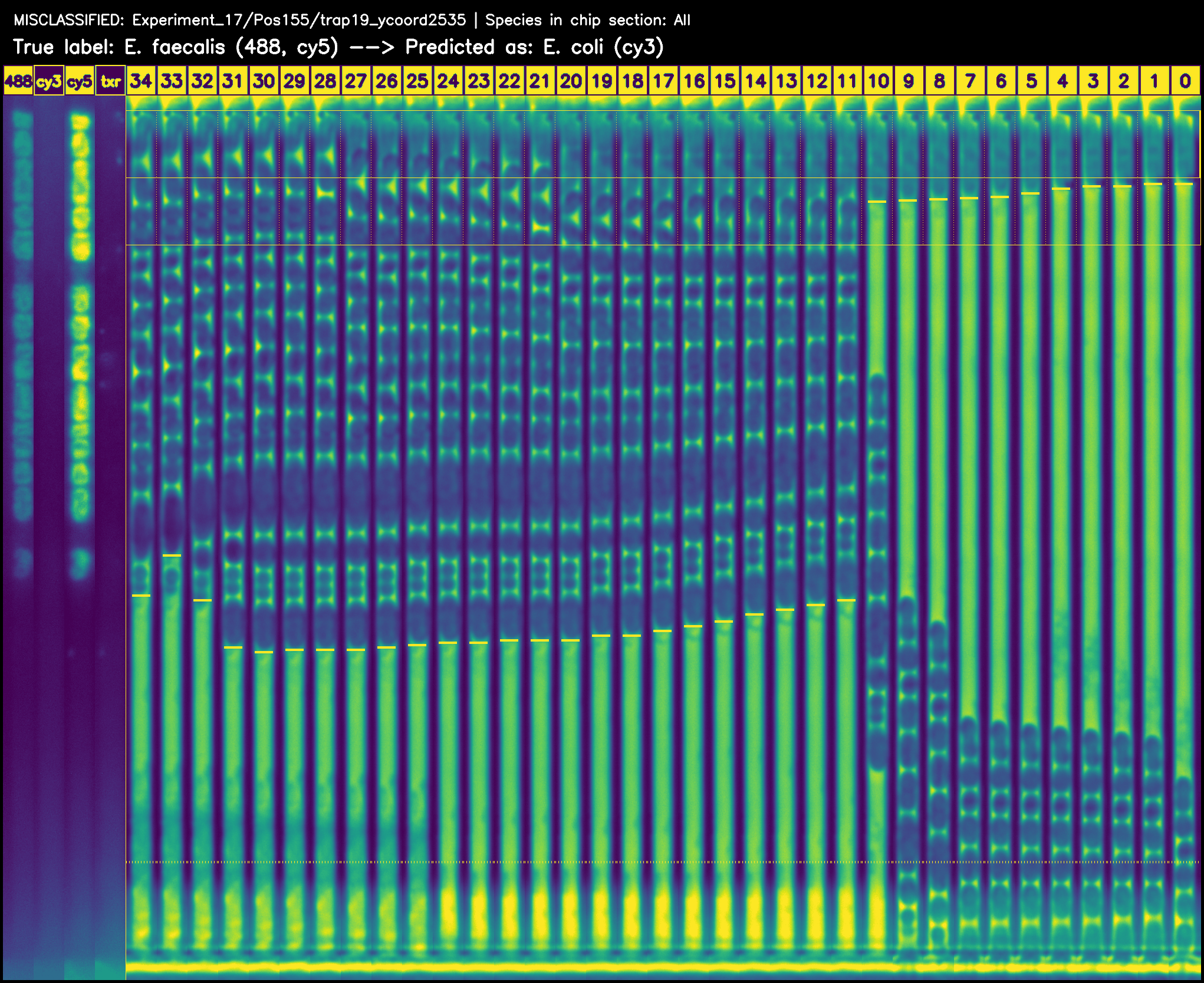

Supplement: S8 Fig — A rod cell is present at the beginning of the time-lapse, which then dissolves and is not visible in the fluorescence images. (PNG) [file pone.0330265.s009.png]

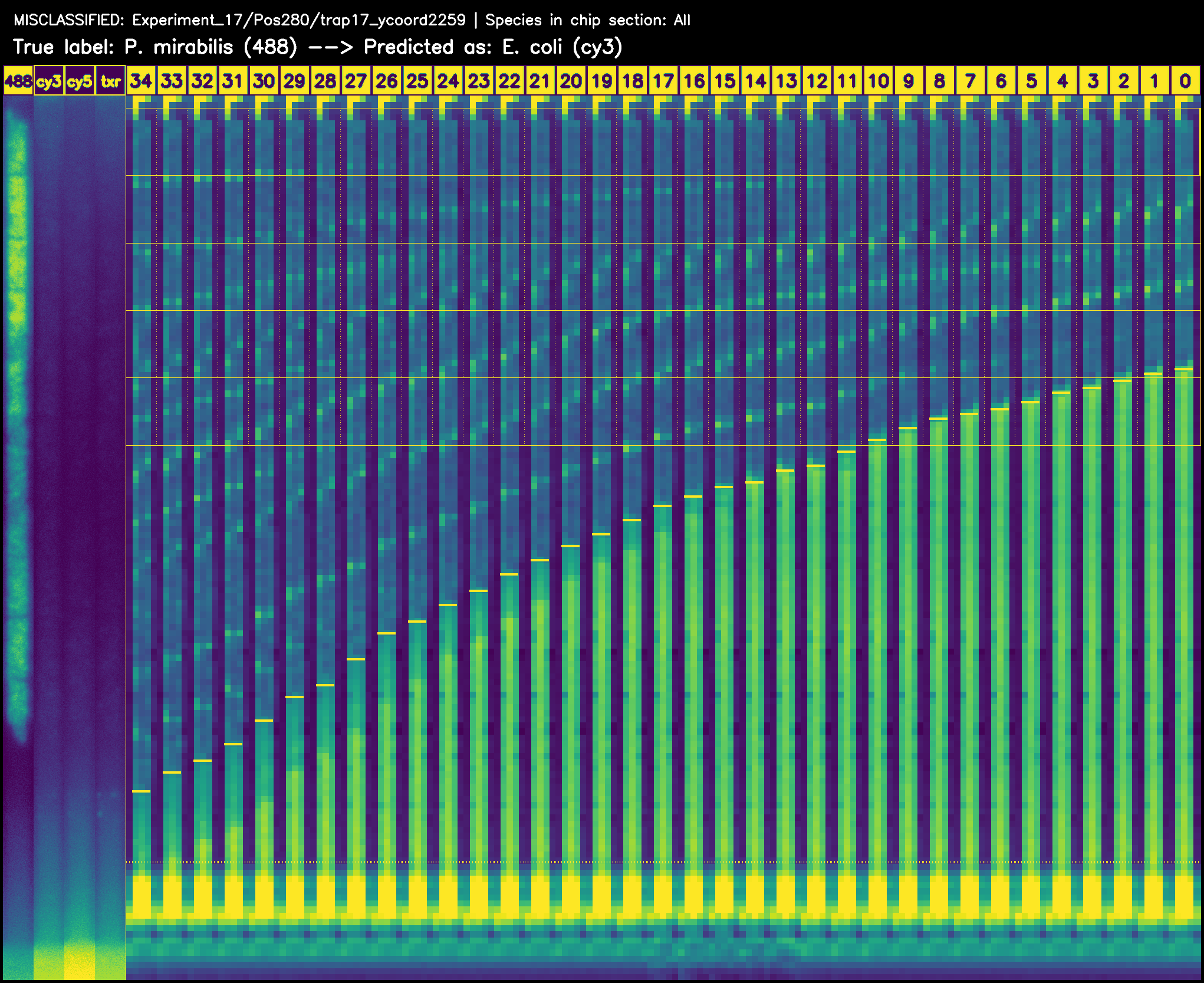

Supplement: S9 Fig — P. mirabilis and E. coli are visually very similar in shape, both being rods. Classifying at very low resolution is significantly more challenging. (PNG) [file pone.0330265.s010.png]

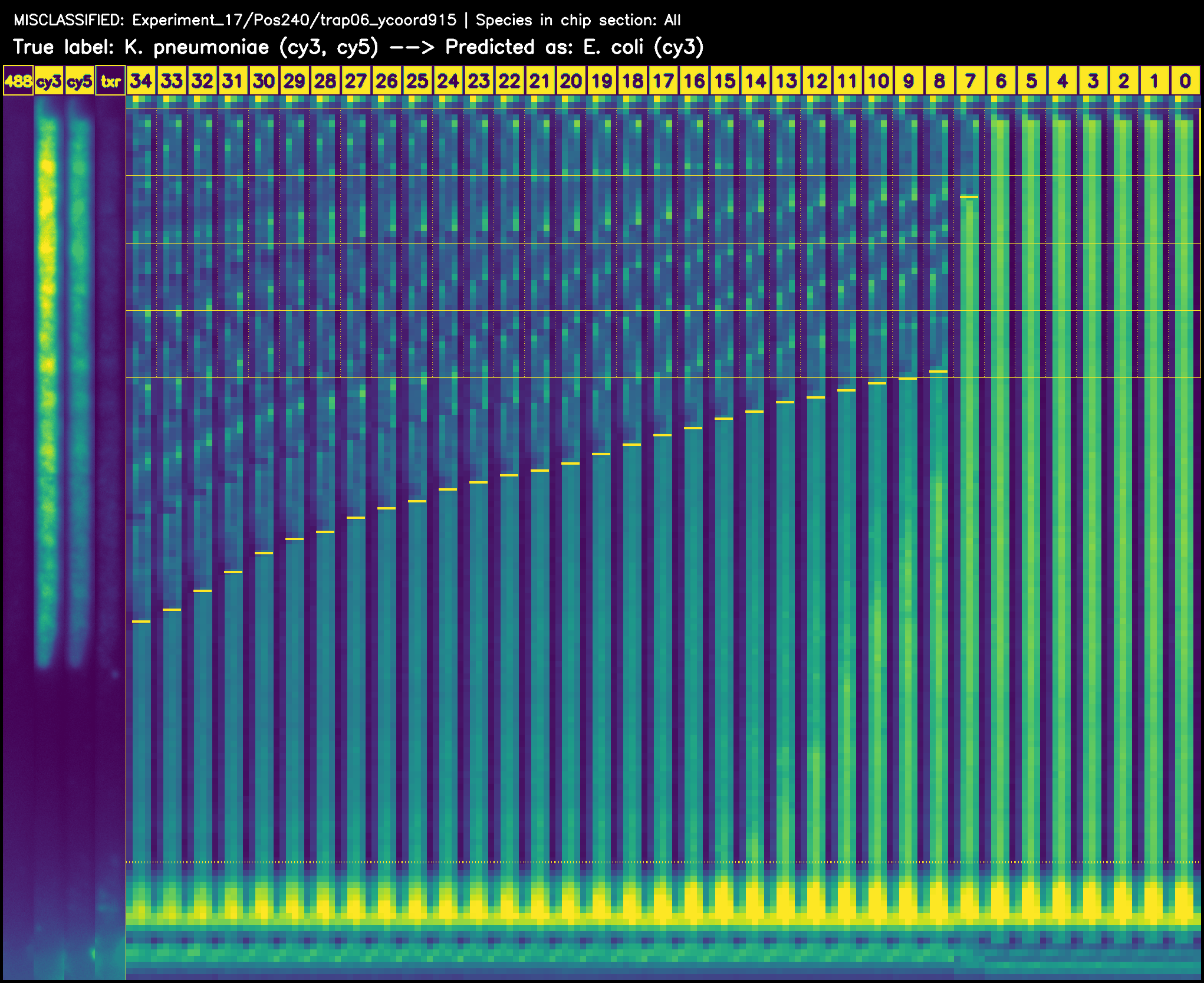

Supplement: S10 Fig — K. pneumoniae and E. coli are visually very similar in shape, both being rods. Classifying at very low resolution is significantly more challenging. (PNG) [file pone.0330265.s011.png]

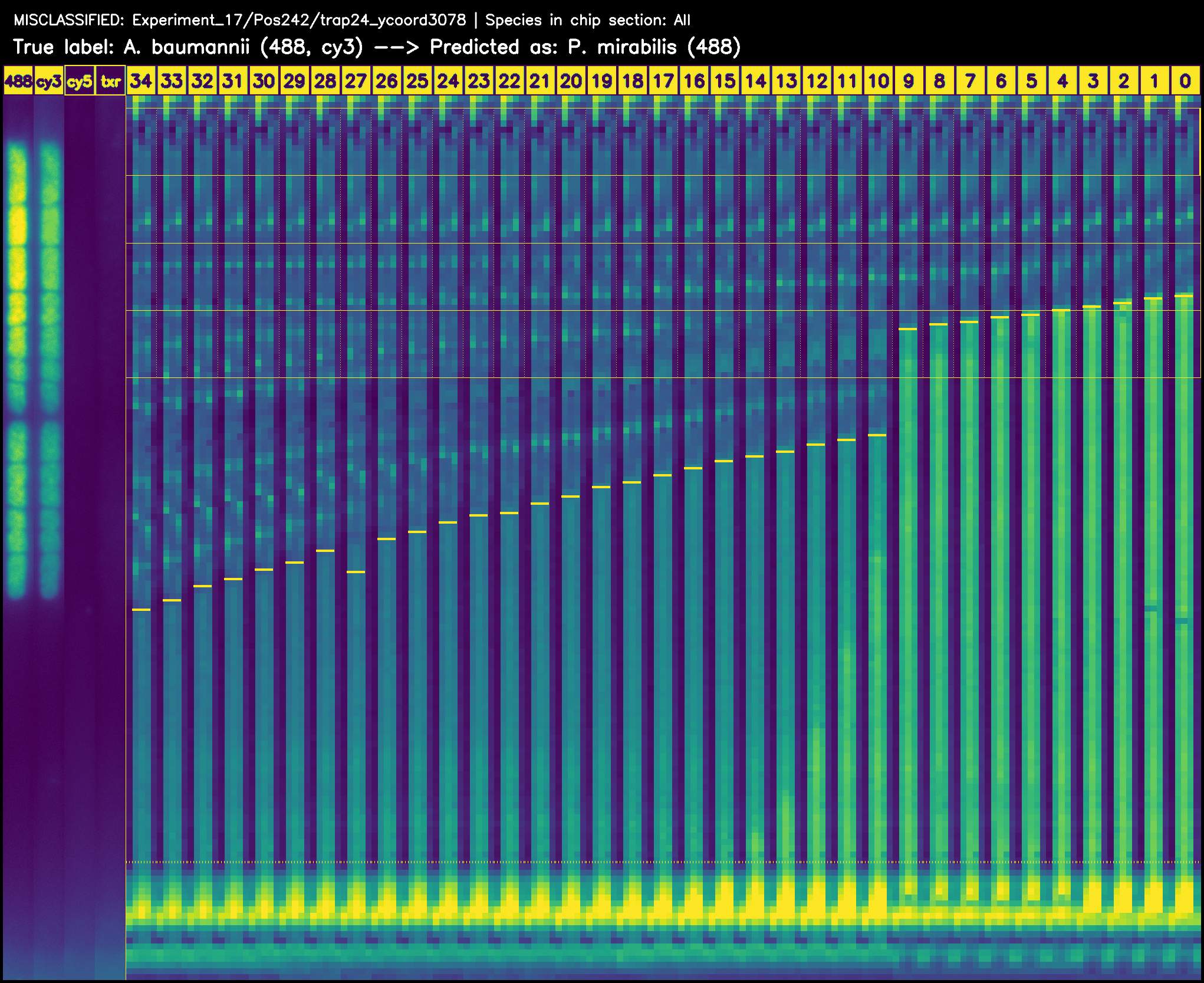

Supplement: S11 Fig — A. baumannii and E. coli are visually very similar in shape, both being rods. Classifying at very low resolution is significantly more challenging. (PNG) [file pone.0330265.s012.png]

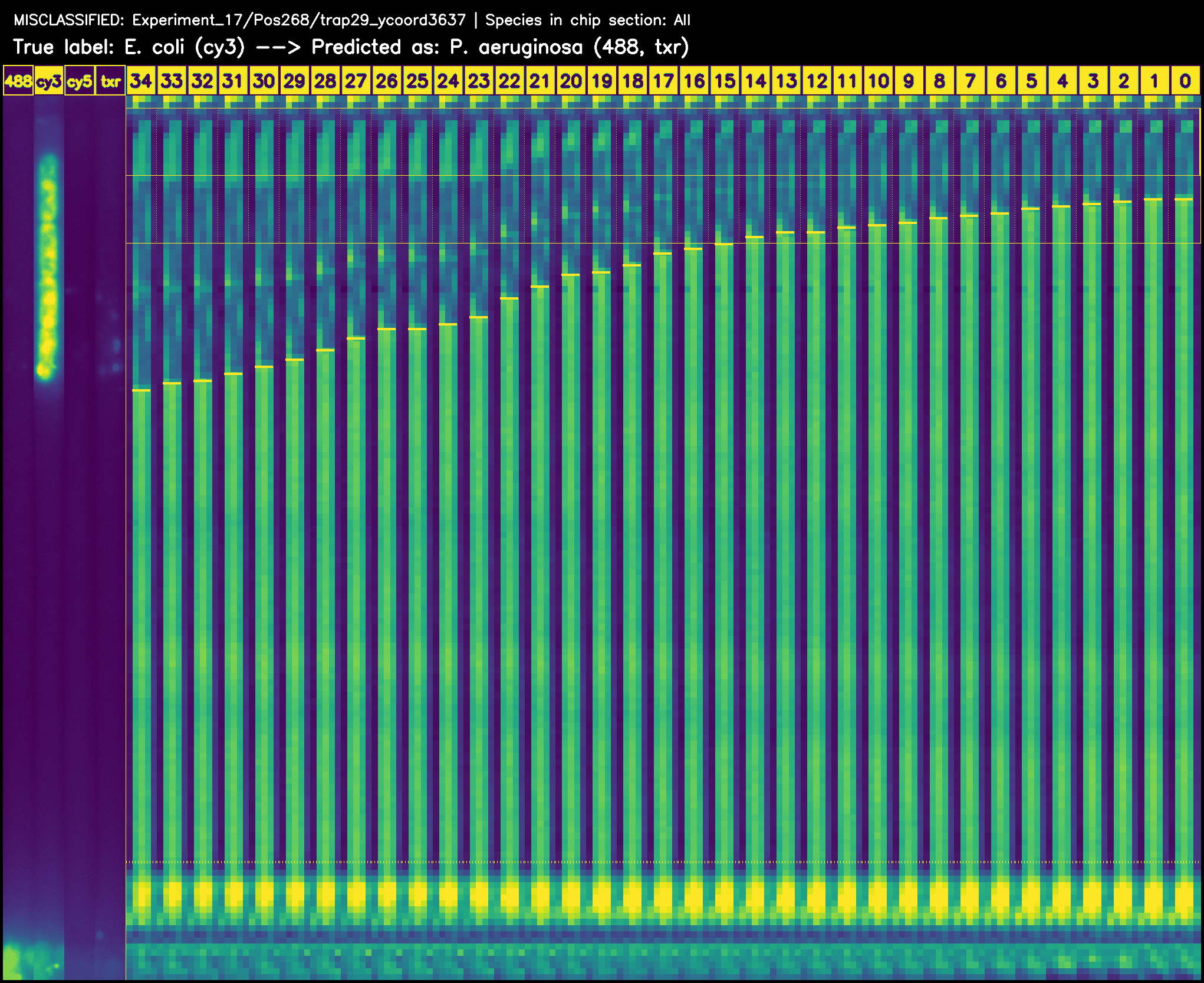

Supplement: S12 Fig — This trap was also misclassified by Video ResNet in the full resolution. E. coli and P. aeruginosa are visually very similar in shape, both being rods. Classifying at very low resolution is significantly more challenging. (PNG) [file pone.0330265.s013.png]

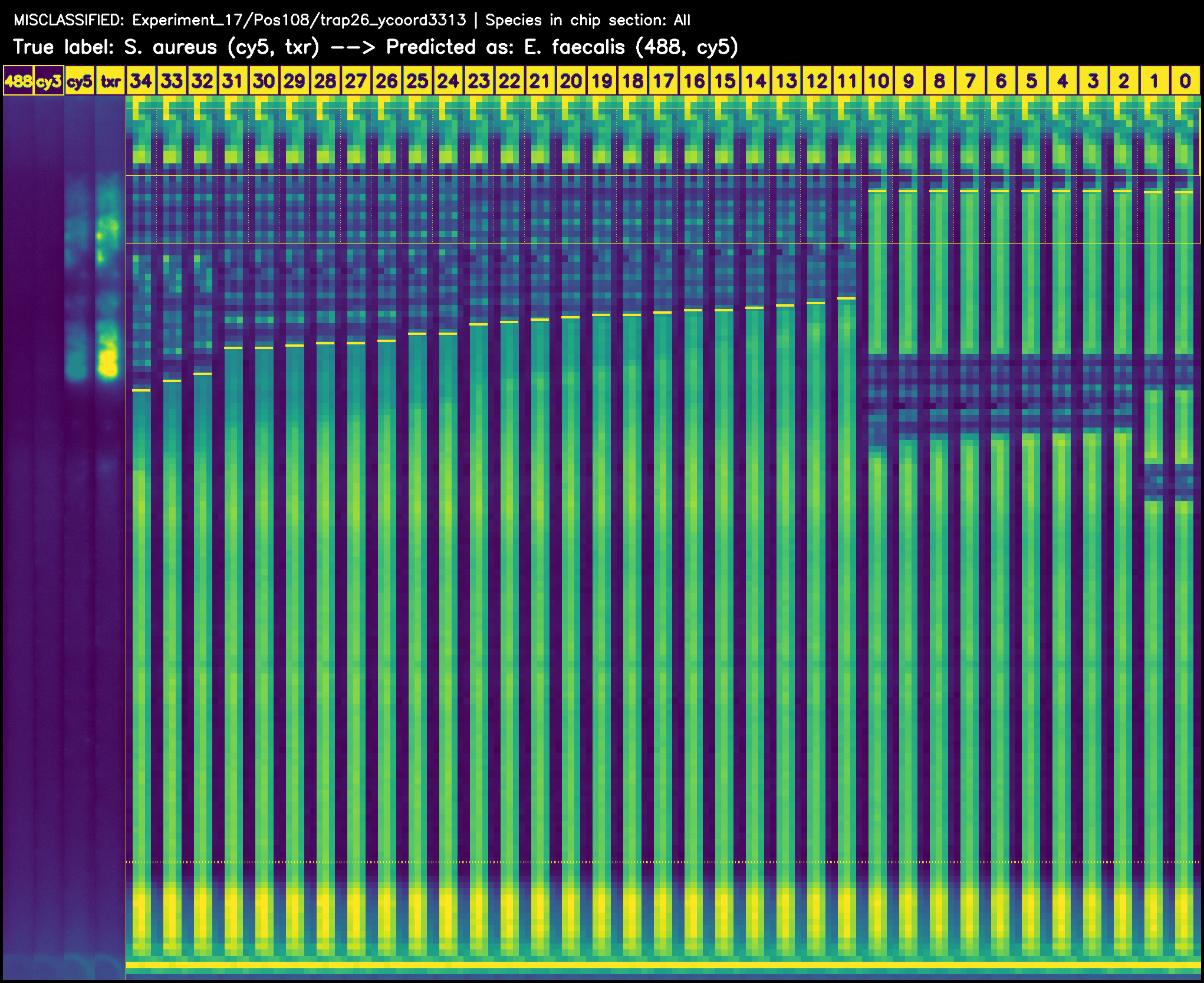

Supplement: S13 Fig — This trap was also misclassified by Video ResNet in the full resolution. S. aureus and E. faecalis are visually very similar in shape, both being cocci. There is a dislocation in the top, one of the video crops has only empty frames. This trap should have been discarded according to the discarding criteria (no dislocation from the top of the trap), but it was missed during the test set inspection. Classifying at very low resolution is significantly more challenging. (PNG) [file pone.0330265.s014.png]

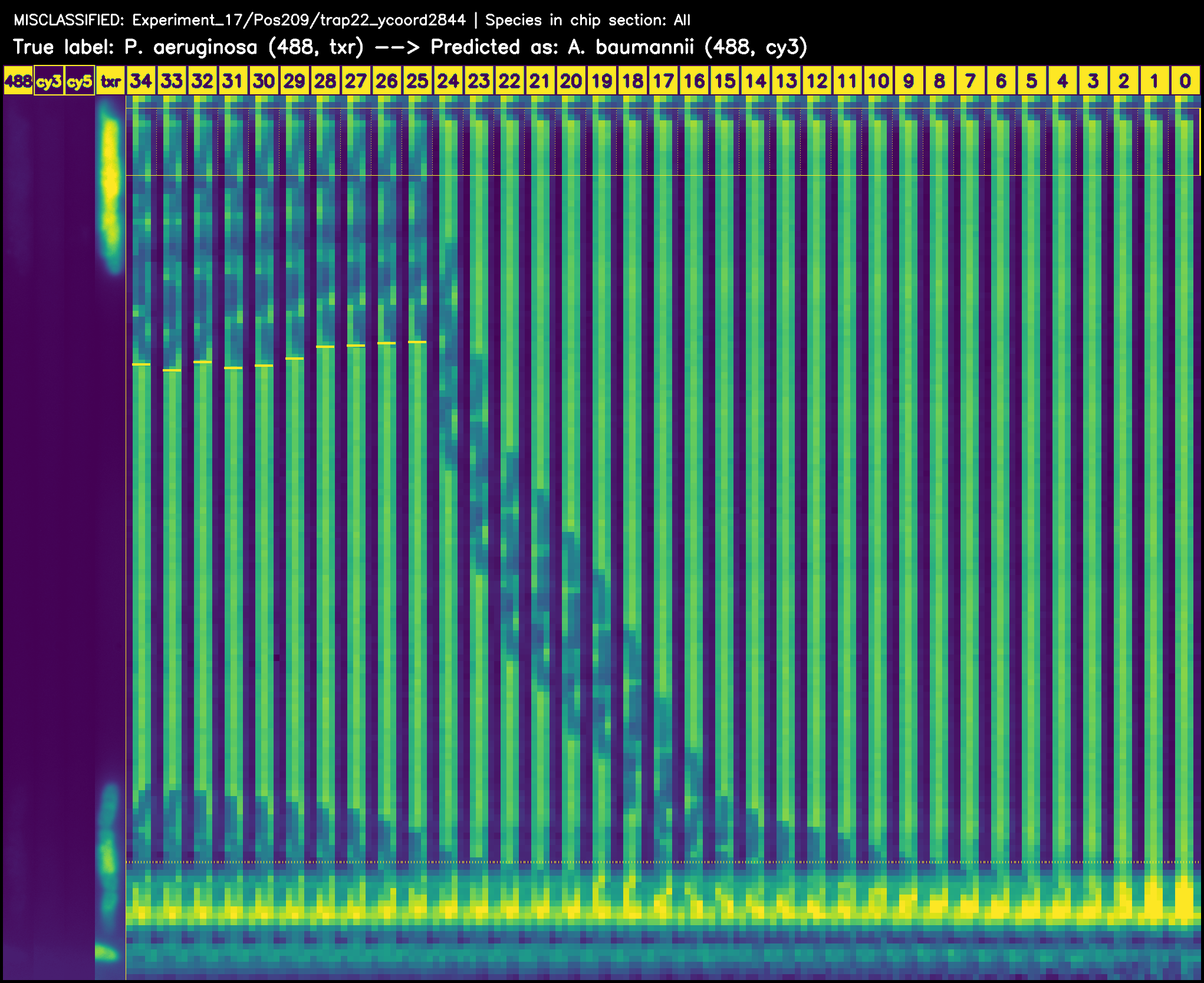

Supplement: S14 Fig — P. aeruginosa and A. baumannii are visually very similar in shape, both being rods. This trap should have been discarded according to the discarding criteria as it is empty until frame 25 (cells must be loaded within 30 minutes), but it was missed during the test set inspection. Classifying at very low resolution is significantly more challenging. (PNG) [file pone.0330265.s015.png]

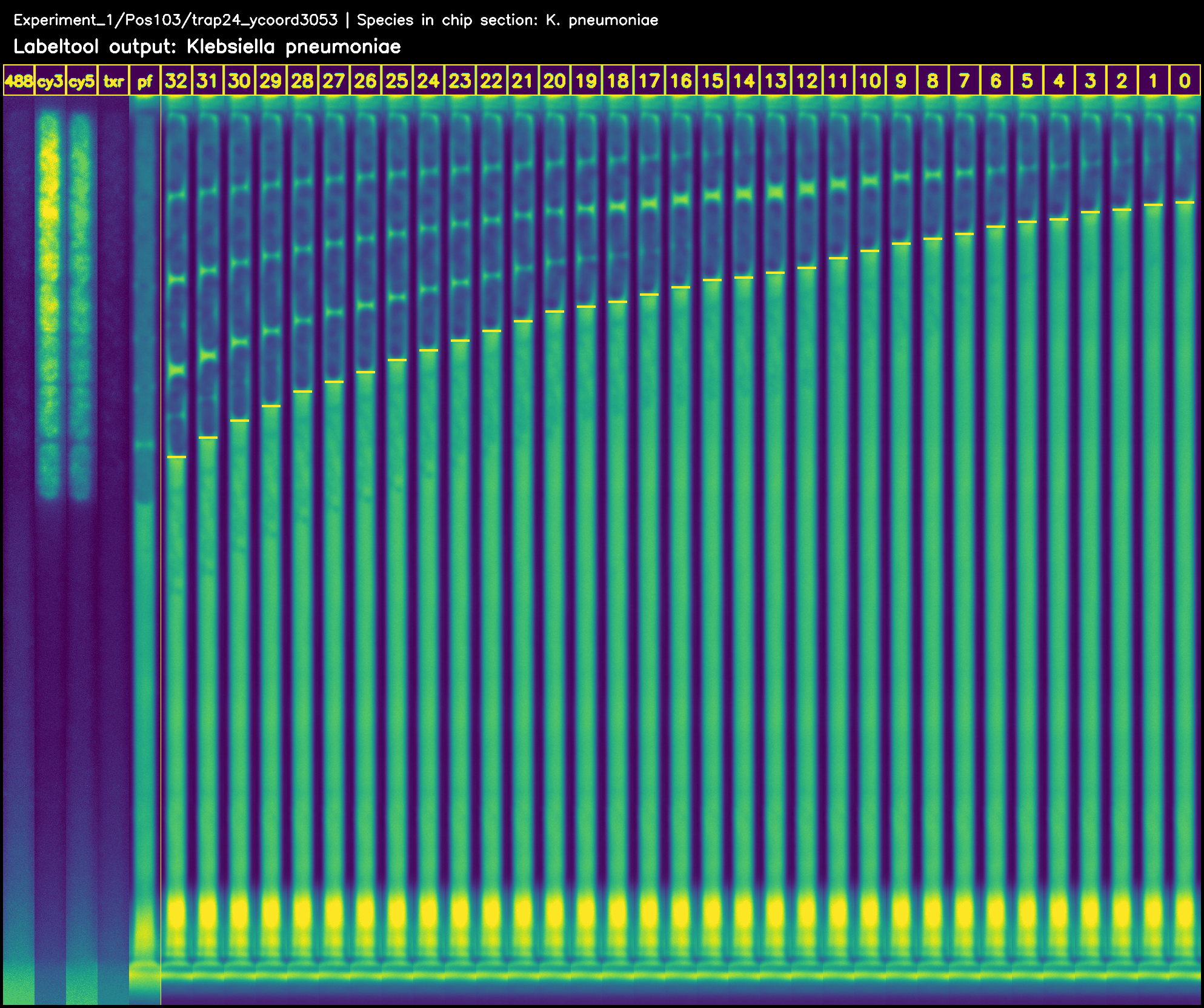

Supplement: S15 Fig — For K. pneumonie the cells are visible in Cy3 and Cy5 in the fluorescent staining. (PNG) [file pone.0330265.s016.png]

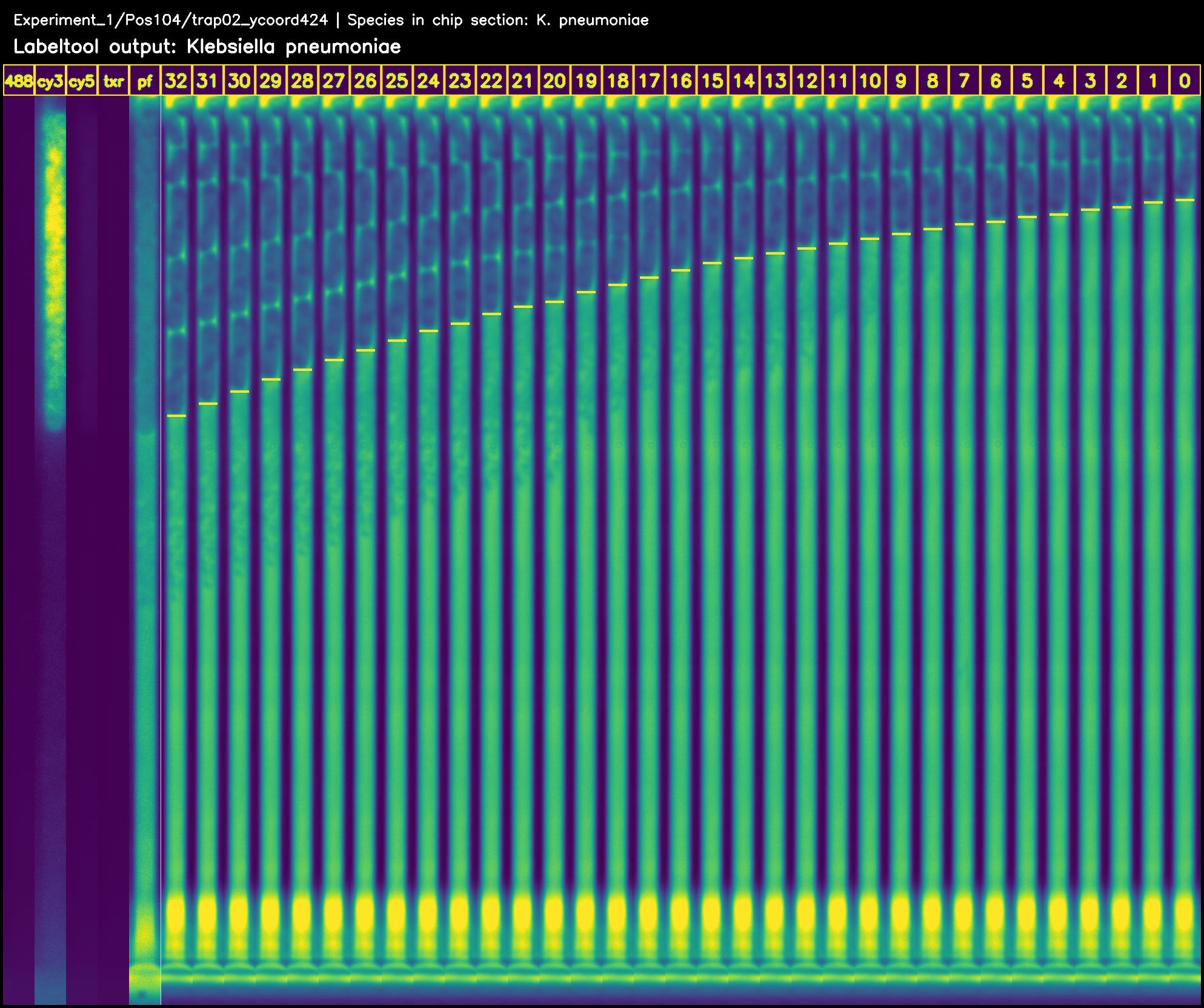

Supplement: S16 Fig — The cells should be visible in Cy3 and Cy5, but there was almost no absorption of Cy5 fluorophores. The trap originated from the same experiment and chip section as S15 Fig, which contained only K. pneumoniae, allowing the labeling tool to infer the correct label. (PNG) [file pone.0330265.s017.png]

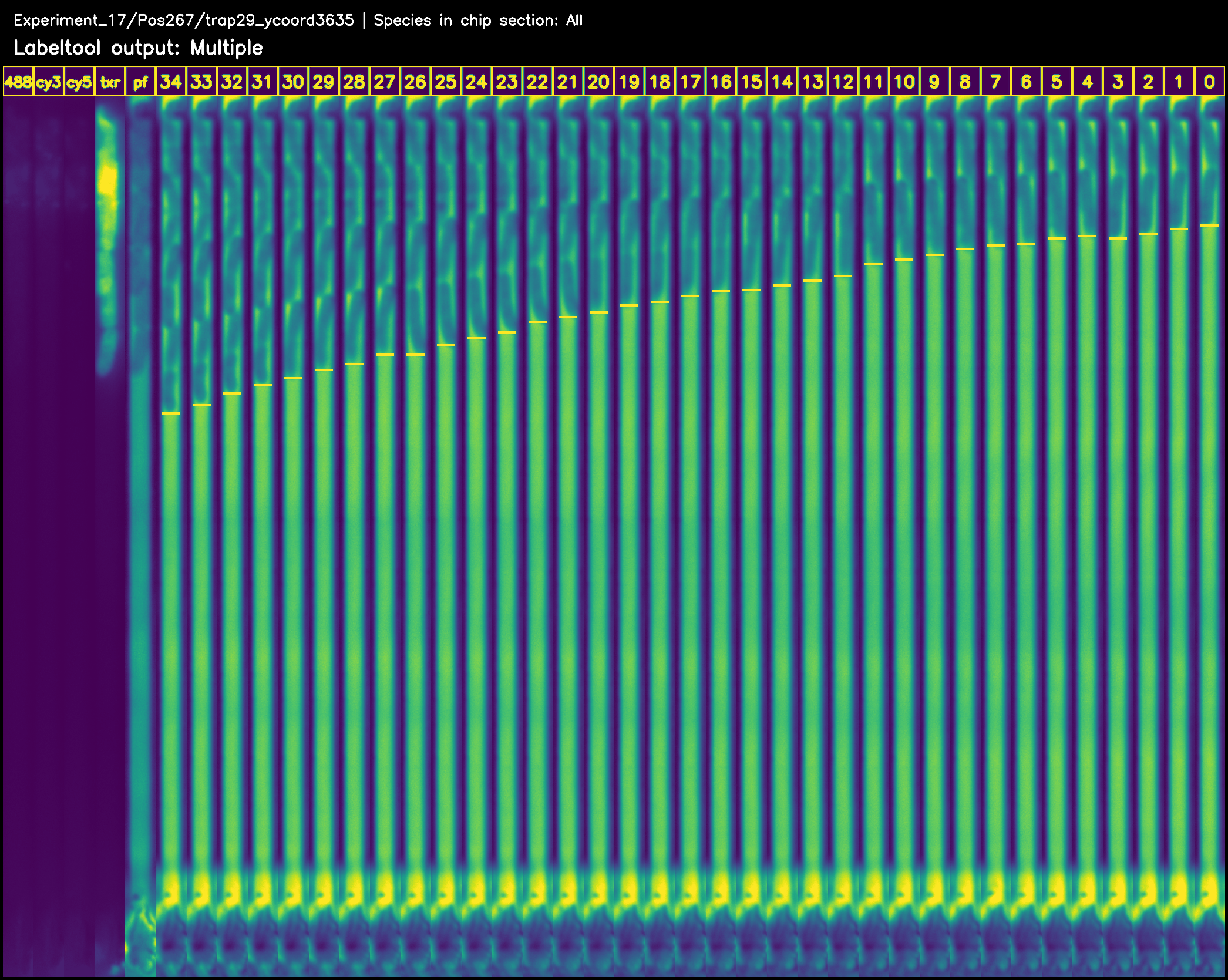

Supplement: S17 Fig — The species should be visible in Alexa488 and TxR, but there was no absorption of Alexa488 fluorophores. It can not be S. aureus (Cy5, TxR) being a cocci; hence, the label was manually assigned. (PNG) [file pone.0330265.s018.png]

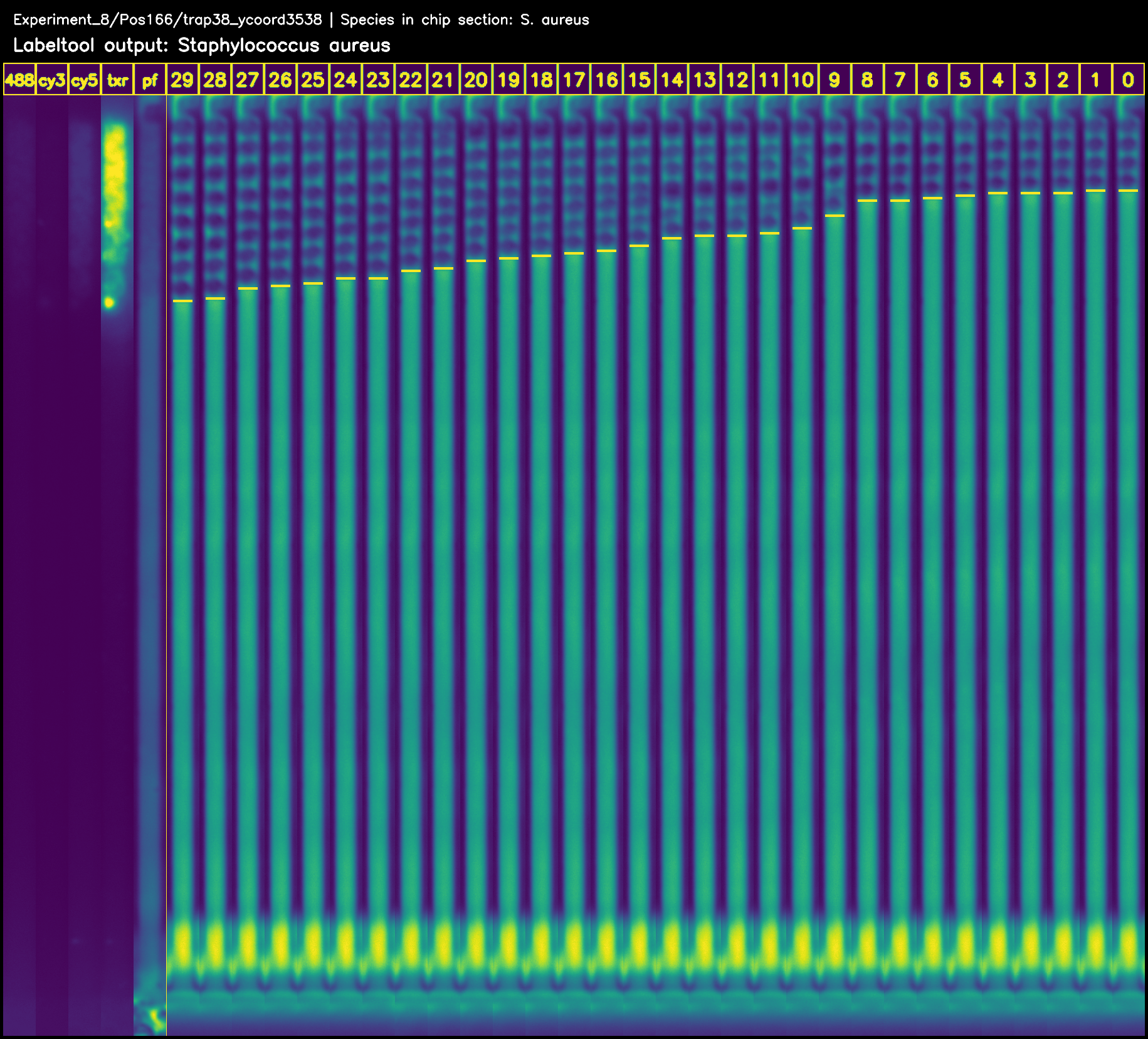

Supplement: S18 Fig — The species should be visible in Cy5 and TxR, but there was no absorption of Cy5 fluorophores. The chip section contained only S. aureus, allowing the labeling tool to infer the correct label. (PNG) [file pone.0330265.s019.png]
