# Supplementary material for: Rapid label-free identification of seven bacterial species using microfluidics, single-cell time-lapse phase-contrast microscopy, and deep learning-based image and video classification
Source: PLoS One. 2025 Sep 8;20(9):e0330265. doi: 10.1371/journal.pone.0330265 (PMC12416834; doi:10.1371/journal.pone.0330265)
Supplement: Attachment — Submitted filename: ResponseLetter.pdf [file pone.0330265.s029.pdf]

Response to the reviewer’s letter

The the reviewer’s comments are cursive in dark blue, our response right below in black.

Good morning -

Thank you for an interesting paper on an ingenious approach to rapid identification of bacteria. I regret that I recommended a "Reject". This was based largely on the detailed and thoughtful comments of Reviewer 1. The comments of Reviewer 2 were sparse (perhaps partly because they found the paper's organization a bit difficult to work with) and so carried less weight.

The article has been split up into subsections for better readability.

Line 48 - 49: Like Reviewer 2, I found the description of the ML a bit confusing. Perhaps you can distinguish the training phase from the intended use case workflow (when a trained model is applied to new samples), and the related train-val-test sets. Verb tense might help with this.

We have clarified the training phase and the use case workflow in lines 64-65:
“...Using this data, we trained deep learning video or image classification models to classify the species growing inside each trap using only the phase-contrast data as input.”

Multi-class ROC figure: It might give helpful context to include two ROCs: the standard (x range = y range = [0, 1]) and the zoomed-in portion. Showing only the zoomed-in portion makes results look worse than they are.

This has been clarified in the figure text in Fig 4 and 6: “...*Note that the plot has been zoomed in for visibility.”*

3. Have the authors made all data underlying the findings in their manuscript fully available
Reviewer #1: NoThe standards of open science are fully respected as far as data analysis is concerned. On the contrary, as far as microbiology is concerned, these standards are not respected : there is no information at all about the strains used. The authors refer to previous papers in PLOS Comput. Bio. and bioRxiv but none of them deals with P. mirabilis, S. aureus or A. baumannii.
The dataset was obtained from a separate publication containing information about the strains and microfluidics. This information has been restated from line 227 in Materials and Methods.

Methodology
When working on a phenotypic identification method, it is important to address both instrumental and biological variabilities. The sources of instrumental variabilities are

correctly addressed but there the biological variability is not discussed. In particular, there should be data from different strains for every species of the database. Some other parameters such as the culture medium should then also be studied as they may have an impact on optical phenotype.

We agree but were not able to expand the study given the scope and our resources. These concerns have been added to “Limitations” in the Discussion.

Substantial evidence for its conclusions

The conclusions rely on substantial evidence except on a specific item of the « Discussion » chapter. The authors wrote (line 187) « the methodology can be applied prior to or concurrently with phenotypic AST testing and give an early warning before results of other approaches to species identification, such as MALDI-TOF ».

There is an obvious lack of evidence to support this conclusion since the authors did not discuss about how to insert a complex sample into a microfluidic chip without an isolation culture on agar medium. How can they fill the micrometric traps of the mother machine device when bacterial cells are dispersed in a complex sample, where much larger human cells are predominant ?

Furthermore, the methodology requires a high concentration of bacterial cells, i.e. 1E5 cfu/mL, to fill the microfluidic device. Once again, the authors did not discuss how they could concentrate the bacterial cells to achieve such a concentration.

This is a good comment, there are parallel studies developing methods to isolate bacteria from blood samples, referenced in line 256-257. The main focus of the study was investigating image processing and how to train neural networks for the task. It has been clarified under limitation in the discussion that this was a proof of principle study.

Accessibility for the broader community

The article is well-structured and includes many bibliographic references for readers who would like to learn about some of the tools used. The article is partly accessible to biologists interested in image analysis, such as the end-users of digital health, but I do not think it is accessible to the broader community of clinical bacteriology.

We agree, but the purpose of the study was mainly to investigate deep learning model training and performance.

Practice of open science

The standards of open science are fully respected as far as data analysis is concerned. On the contrary, as far as microbiology is concerned, these standards are not respected : there is no information at all about the strains used. The authors refer to previous papers in PLOS Comput. Bio. and bioRxiv but none of them deals with P. mirabilis, S. aureus or A. baumannii.

The data was obtained from another study that contains information about microbiology and bacterial strains. This information has been restated for convenience in the “Materials and Methods section”

Originality

All the methodology, whether it deals with microfluidics, optics or data analysis, has already been tested and described by the same authors, in a previous article of PLOS Computational Biology : the trapping of cells in a mother machine, data labelling with FISH (fluorescence in situ hybridization), the spatiotemporal analysis (video and classification). There is nothing new in the article, except that the database includes 7 species this time, instead of 4, and that some minor improvements have been achieved in the image processing. There is no breakthrough in comparison to their previous article.

We agree that there are no major "breakthroughs", however, we believe useful and novel results are obtained, by studying real-time performance as additional frames are captured during testing, and investigating the role of training set size, data quality, and data augmentation, as well as the contribution of texture and morphology to performance.

Comments

Author summary : « Labelling cells with fluorescence in-situ hybridization, artificial neural networks models are then trained to identify… » should be written, instead of « artificial neural networks models are then trained to identify… »

The author summary is not needed for PLOS One and was removed.

Line 20 : A reference should be also made on multiplex-qPCR as its use increases a lot in clinical microbiology

Added in lines 22-23

Line 21 : The authors talk about « genotyping by fluorescence microscopy ». The technique should be called by its common name, i.e. FISH (fluorescence in situ hybridisation)

Changed in line 25

Line 23 : There should be a conclusive sentence after presenting the genomic methods, at the end of this paragraph: why genomic and spectroscopic methods are not satisfying ?

Added in lines 28-32

Line 24 : There should be a clearer transition between the previous paragraph and the microfluidic methods for single-cell characterization: why did you chose to focus on methods for single-cells?

Discussed in lines 31-36

Line 32 : References are given to get details about the microfluidics but a minimum of information should be given in the article itself, at least an order of magnitude of the pressure and flow rates, as well as the time required for filling the device.

Added in lines 47-49.

Capture of Figure 1 : When working on a phenotypic identification method, even for a proof-of-concept, it is important to start with a database that covers the four major categories of pathogens : Gram+ and Gram- cocci and rod-shaped bacteria. However, the authors chose not to cover Gram-negative cocci and Gram-positive rod-shaped cells. Furthermore, they did not discuss at all their choices for the database.

We were unable to obtain this amount species given the scope of the study, this has been elaborated on in “Limitations” in the Discussion.

Line 67 : The methodology for validation/cross-validation is not sufficiently explained, especially for non-specialists. To make the article more accessible to a wider audience, this is an aspect to improve.

The training and test sets were clarified in lines 85-87, detailed information is in Table 1.

Line 75 : The number of strains per learning class, i.e. per species, is not given.
The bacteria were laboratory strains, however, still challenging when the rods almost looked identical.

Line 86 : « summing the output logits » should be explained.
“…The final output for a trap was obtained by summing the output logits (unnormalized network output) from each crop (image or video clip).”

Line 91 : The cross-validation was seemingly done in « one vs. rest » (written in the caption of the Figure 4). It should be precised in the text here.

No cross-validation was used, the training and test sets (experiments) were fixed among all retrainings. Each network was retrained multiple times with identical hyperparameters.

Lines 96-97 : Poorly accessible to a broader community.

Information about visualization (Strip plot and Beeswarm plot) was moved to the materials and methods.

Line 115 : « Lancoz » is uncorrectly spelled : "Lánczos" is the correct spelling.

The spelling was corrected.

Line 135 : « Albumetation library » is uncorrectly spelled : "Albumentation" is the correct spelling
The spelling was corrected.

Line 141 : « Finally, one experiment was conducted removing all train augmentations. » should be replaced by « Finally, one experiment was conducted removing all training augmentations. »
The text was corrected.

Line 143 : The reference 37 is not really sufficient to understand the concept of « label smoothing » and how the authors performed it. « Label smoothing » should be explained, at least in the « Materials and methods » part.
Label smoothing was explained in line 512.

Line 180 : The authors comment the similarities of morphotypes between rod-shaped species (« two new rod species, P. mirabilis, and A. baumannii, which were visually almost identical to E. coli and K. pneumoniae »), but do not comment the case of Gram-positive cocci (E. faecalis & S. aureus).

This is comment on in Fig 13.

Lines 194-198 : For ease of reading, the authors should recall each time which figure is related to the stated outcome.
This has been added in the Discussion, each figure is referenced to each stated outcome.

Line 217 : « Instead of days to obtain positive cultures, typing, and phenotypic drug sensitivity testing results, these new methods decrease the waiting time to hours or even minutes. » With this sentence, the authors state that their methodology can be applied directly to samples, without any prior isolation culture step. But they do not discuss at all the ways to achieve such an ambitious goal : how can they fill the microfluidic device with a raw sample, or how can the sample be processed prior entering the microfluidic channels ?

This has been elaborated on in the Discussion section, lines 255-257.

Line 229 : The authors refer to a bibliographic reference [16] for details on biological experiments, but this article does not mention the references of the used strains. Are they clinical isolates ? Are they reference strains ? What are their collection numbers ?
Information about the strains have been added in the Materials and Methods section, lines 288-291.

Reviewer #2: Few suggestions, for ease of reviewing:

1. Ordering of the material - interms of flow of the paper starting from introduction, results, discussion, methods; makes the paper very challenging to review.

The paper has been split up into subsections with research questions clearly stated to become easier to read.

2. In the results as well as methods section - suggestion to use language in past tense, as this is about the data which had been taken from the works of Kandavalli et al.

The tense has been changed to past tense in the paper.

3. To avoid descriptions of the methods in the results section, and add it to the method section.

The methods were only stated in the Results section and elaborated in the Materials and Methods.

4. The titles of the pictures and graphs - were not in sync in the pdf downloaded, hence seems a bit challenging to comment on.

Also, next to the title, a suggestion to include description as notes in the title explaining the graph/pictures and its contents for ease of understanding and appreciating the work.

We are not sure how to address these concerns.

Best regards,

Erik Hallström and Co-authors.
