# Supplementary material for: Rapid label-free identification of seven bacterial species using microfluidics, single-cell time-lapse phase-contrast microscopy, and deep learning-based image and video classification
Source: PLoS One. 2025 Sep 8;20(9):e0330265. doi: 10.1371/journal.pone.0330265 (PMC12416834; doi:10.1371/journal.pone.0330265)
Supplement: Attachment — Submitted filename: Response_to_the reviewers_letter.pdf [file pone.0330265.s030.pdf]

Response to the reviewer’s letter

Dear reviewers,

Thank you for your comments and valuable feedback on our study - the reviewers’ feedback has helped us improve the paper. The reviewers’ comments are cursive in dark blue, our response right below in black.

Reviewer #1:

The manuscript presents a novel approach for bacterial species identification using microfluidics, time-lapse microscopy and deep learning. While the methodology is technically sound, several critical issues must be addressed before publication can be considered. The authors emphasize their "label-free rapid identification" as a key innovation. However, the previous work already demonstrated this approach for 4 species. Simply extending to 7 species without significant methodological advancement is insufficient.

A number of improvements have been made as compared to our previous publications on classifying four species, such as a new pipeline to align, register, and crop out traps from the raw images (line 379), using tile aggregation for both image- and video classification to utilize all image data containing cells (line 144), using more augmentation methods (line 519) and label smoothing (line 554) to prevent overfitting.

Furthermore, the study extends our previous work by evaluating real-time classifier performance as a function of the number of observed time-lapse frames, and by investigating the effects of training set size, data quality, augmentations, and testing how texture and morphology contribute to classifier decision (paraphrasing line 225).

No clinical isolates were tested, only lab strains. The claimed clinical applicability remains unproven.

As the reviewer points out, the clinical applicability remains unproven, but the clinical application is always referred to as “potential” (line 131, 257, etc), and we emphasize that the study is a further improvement that builds upon the previous study, and is still at the proof-of-principle stage (stated in the Abstract, line 284). Future work must validate the method on more species and clinical isolates (stated in line 287-288). Additionally, we believe the computerized image processing/deep learning experiments are of value for the research community aiming to identify species using only video information of single-cell divisions.

In the PLOS One journal information in *“What we publish”* it is stated that: *“In keeping with our mission to publish all methodologically and ethically rigorous research, we will consider Research Articles reporting negative and null results.”* https://journals.plos.org/plosone/s/what-we-publish. Furthermore it is stated in the journal information that *“replication studies, negative and null results are all in scope”*

https://journals.plos.org/plosone/s/journal-information Also, in *"An Editor's Guide to the PLOS One Article Types"* in the gray box on the bottom of the first page it is stated: *"Do not consider novelty. PLOS One welcomes all well-conducted valid scientific research, including negative, null and replication studies."*
https://genweb.plos.org/RR/EditorResources_PLOS-ONE-Article-Types.pdf

This is in line with the journal's mission to advance science, mitigate publication bias, and address the replication crisis. Hence, the "key innovation" and "significant methodological advancement" outlined by the reviewer is not a requirement for publication in the journal. Regarding "significant methodological advancement" as outlined by the reviewer, we actually envision the methodology to be the same regardless of using laboratory strains or clinical isolates, collecting more diverse high quality data and retraining the networks.

In conclusion, results from the previous four species article are replicated and extended to seven species testing on a separate experiment. Additionally the computerized image processing/deep learning methods are investigated. We believe the current version of the article is publishable in the journal according to the guidelines.

The "one-hour identification" claim lacks proper context.
The one hour identification is conducted in a laboratory setting, with the stated frame rate (line 158). We observed that the models become more accurate when accessing more frames, thus using spatiotemporal features. This was especially true for visually similar species (*K. pneumoniae*, *E. coli*) as visualized in Fig 3. We believe this is an interesting result, and may extend to visually similar strains in a clinical setting which future work must demonstrate.

No comparison with standard methods, such as MALDI-TOF or qPCR in terms of total processing time.
In the MALDI-TOF machines used clinically today typically require pre-cultivation on agar plates for colonies to form, requiring 18-24 hours (stated in line 48). The cited qPCR method for bacteria identification has a 6-hour turn-around time (added on line 56), and the applications using microfluidic mother machines typically require a total time of 1-2 hours including isolation (added on line 69).

No cost analysis provided despite emphasizing "low-resource settings".
The "potential low-resource setting" (stated on line 131) is referring to the experiment results where high accuracy was retained training and testing the networks on spatially downsampled images, which requires significantly lower computational hardware for inference. Exact cost analysis is hard to make, as the technologies are constantly evolving, however the components of a microfluidic system are typically less expensive (microfluidic chip and a phase-contrast microscope) than the MALDI-TOF, but the main advantage of the proposed method is speed.

The microfluidic system requires high bacterial concentrations. No solution is provided for processing complex clinical samples.
Recent developments of quickly loading the microfluidic system are briefly covered in "Microfluidics" in the Introduction (line 74-87). With regards to complex clinical samples, one approach for loading the microfluidic device is mentioned and cited in line 300 (Henar et al.).

Furthermore Sysmex-Astrego currently has the PA-100 microfluidic system on the market for urine tract antibiotic susceptibility testing, mentioned in line 86 and 292.

Only single strains per species were tested. No evaluation of medium effects on optical phenotypes. Gram-positive rods and Gram-negative cocci completely omitted.
This is mentioned in the Abstract, and in Limitations and future works on lines 281-286. However, we would like to emphasize that the problem is not trivial despite using laboratory isolates. It is very challenging, even for a human eye to distinguish between the different species of rod-shaped bacteria, as stated on line 252-254 and in Fig 13.

It is recommended to clearly differentiating from previous work, demonstrating clinical isolate testing, and adding comparison tables.
The developments as compared to previous work is discussed in the first paragraph in the discussion, line 246-266.

Reviewer #2:

This study presents a rapid diagnostic approach for identifying bacterial pathogens within one hour using phase-contrast time-lapse imaging of single bacterial cells trapped in a microfluidic "mother machine". By training deep learning models —specifically Convolutional Neural Networks (CNNs) and Vision Transformers (ViTs) — on these videos, the authors showed that they can accurately classify seven common infection-causing species.

The method achieves high performance and leverages spatiotemporal features such as cell texture and morphology. It also supports real-time evaluation as new frames are captured and can be integrated with phenotypic antibiotic susceptibility testing (AST). Despite its promise, practical challenges like isolating bacteria directly from blood and validating against clinical strains remain. This proof-of-concept marks a step toward real-time diagnostics for acute infections.

I consider this study being publishable in PLOS ONE, as this jounrnal publishes solid, reproducible science — regardless of novelty or expected impact. I am saying this because this study is a follow-up study of an earlier study by these authors published in PLOS Computational Biology (Reference 21). What makes this study different from their previous work is that they (i) study real-time performance by capturing additional frames during testing, (ii) investigate the role of training set size, (iii) data quality, and (iv) data augmentation, as well as (v) the contribution of texture and morphology to performance.

The manuscript is written fairly technically and recent biological advances are missing from the manuscript.

We agree, and the reason for this is that the focus of the article is on computerized image processing/deep learning to aid future research of learning spatiotemporal features from single cell division or potentially enabling visual identification of other pathogens or organisms.

I ask the authors to include and discuss recent advances on antibiotic susceptibility testing into their manuscript. In fact, looking at pubmed (https://pubmed.ncbi.nlm.nih.gov/?term=antibiotic+susceptibility+testing&sort=pubdate), work in this area is exponentially increasing during the last years, while the authors do not cite any of those studies after the year 2022. This should be improved by discussing their own approach in the context of other approaches (for example, see this recent work: https://doi.org/10.1016/j.snb.2024.136866 and the references discussed therein).

I recommend a minor revision for improving the missing references to recent work being implemented in the Introduction or Discussion section.

The main focus of the paper is species identification using image processing, not AST. However, obtaining species information in parallel with, or even before performing AST provides early clues on optimal patient treatment, and one could envision systems performing both tasks. In line with the suggestion of the reviewer, we have added a section about AST to the Introduction on lines 14-41, including the paper suggested by the reviewer. Additionally, we added a paragraph to the discussion on line 291.

Reviewer #3:

Hallström et al. present a thorough work on bacterial species classification using Deep learning in a Mother Machine. I find it especially interesting that they investigate the possibility of just using masks do do the classification with high accuracy. This suggests that the method should work well under different conditions as long as good segmentation is possible. Maybe this should be emphasized a bit more in the Discussion, but i leave it up to the authors if they want to do that.

We would rather say that the results suggest that the deep learning models are using both morphology, texture and spatiotemporal features for the decision (as one would expect in a DL model, many small cues). However, for these species accurate results can be obtained using only the cell shapes. In future work with clinical isolates, morphology alone may not be sufficient.

My main issue is that it is not clearly stated how and where the Code and Data can be obtained without restrictions. In the Data Availability section the authors have just stated that "Yes - all data are fully available without restriction". It's even mentioned by PLOS that "'data available on request from the author' is not sufficient". I saw that the dataset is from a different publication where the data availability was "data is available upon reasonable request from the author', but as there is at least one co-author on both publications so please make an effort to make both Code and Data available. I would personally suggest https://bioimage.io/#/, but if the authors want to use other repositories that is also fine.

The data is fully available on Zenodo, this is filled in "Describe where the data may be found in full sentences" in the submission system:

""The authors confirm that all data underlying the findings are fully available without restriction. A replication package is available at https://doi.org/10.5281/zenodo.13321089 containing all

image data and software to reproduce the experiments, generate output metrics and build the graphs in the article, in addition to pre-trained models.""

Also the data/code citation is in the paper [51], stated on line 321 in the first paragraph in Materials and Methods. We added "image data" to the sentence for clarification:

""The following sections outline the main components of the software and data. However, for full implementation details, refer to the released replication package [51], which contains documentation, software and all image data to reproduce the experiments, as well as pre-trained models.""

For the species detection based purely on segmentation masks, it would be interesting to see how well Linear Discriminant Analysis would perform just based on standard shape characteristics that you would get from ImageJs "Analyze Particles" or similar methods.

This could be interesting to try, however current model performance improves when the model can access all information, not only the shape. If future species or isolates are to be classified, shape information may not be sufficient, it would be better to collect a high quality dataset and retrain the DL models.

Best regards,

Erik Hallström and the co-authors
